# N–O Dual Functional Group Transposition via Energy Transfer Photocatalysis

**DOI:** 10.1002/advs.76772

**Published:** 2026-07-23

**Authors:** Lei Bao, Xueyu Wang, Yang Zhou, Beibei Zhan, Xiaheng Zhang

**Affiliations:** ^1^ School of Chemistry and Materials Science Hangzhou Institute for Advanced Study University of Chinese Academy of Sciences Hangzhou P. R. China; ^2^ College of Geography and Environmental Sciences Zhejiang Normal University Jinhua Zhejiang P. R. China

**Keywords:** 1,2‐radical migration, dual functional group transposition, energy transfer photocatalysis, molecular editing

## Abstract

Molecular editing and skeletal remodeling have emerged as highly innovative and synthetically efficient strategies. Within this context, functional group translocation facilitates streamlined synthesis and direct access to unique molecular scaffolds. While research in this area has largely centered on single‐group transposition, dual functional group transposition (dFGT) remains underdeveloped due to the demanding requirement for stringent chemo‐ and regioselectivity control. Here, we report a photocatalytic energy transfer (EnT) strategy that achieves dFGT through precisely regulated radical sorting, governing the sequence of migration and coupling events. This approach is applicable to both activated and unactivated alkyl derivatives for N–O dFGT. Combined theoretical and experimental studies reveal a mechanism involving EnT‐driven decarboxylative diradical generation, followed by 1,2‐phosphatoxy migration, and culminating in radical–radical coupling. Furthermore, this methodology has been successfully extended to N–N dFGT, demonstrating its general applicability.

## Graphical abstract

N–O dual functional group transposition (dFGT) enabled by the photocatalytic energy transfer (EnT) has been developed. This methodology is applicable to both activated and unactivated alkyl derivatives for dFGT, and it also successfully extended to N–N dFGT, demonstrating its general applicability.

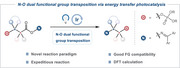

## 1 Introduction

Molecular editing has emerged as a transformative pattern in modern organic synthesis, enabling atomically precise manipulation of molecular frameworks beyond conventional peripheral functional group modification. This approach allows direct “editing” of core molecular skeletons or functional groups through insertion, deletion, or swap operations, offering unprecedented precision and efficiency in synthetic design [1, 2, 3, 4, 5, 6]. Such capabilities are particularly valuable in total synthesis and the late‐stage modification of pharmaceuticals and natural products [7, 8]. Within this context, functional group transposition has recently gained recognition as a powerful molecular editing method that facilitates the migration of a functional group to a different reactive site to rapidly access valuable molecular architectures [9, 10, 11, 12]. This strategy not only streamlines synthetic routes but also unlocks reactivity at sites that are conventionally challenging to functionalize. Despite these advances, the development of dual functional group transposition (dFGT), a promising molecular editing method that achieves the controlled, coordinated migration of two distinct functional groups across a single carbon skeleton in one synthetic operation, remains elusive. Importantly, our definition of dFGT centers on sequential radical cascades consisting of radical generation, functional group migration, and cross‐coupling, rendering it mechanistically distinct from traditional systems relying on synchronous, parallel skeletal migration of two functional groups. Its progress is primarily impeded by the formidable challenge of achieving synergistic chemo‐, regio‐, and temporal selectivity for two distinct functional groups, which often exhibit competing reactivity within a single molecular scaffold—specifically, one functional group must undergo selective rearrangement without interfering with the other, and each migration event must target a designated carbon position rather than off‐target sites. Successful implementation must prevent cross‐coupling, elimination, and other undesired side reactions while precisely controlling sequential or concurrent migration pathways to avoid isomeric mixture formation [13, 14].

**FIGURE 1:**
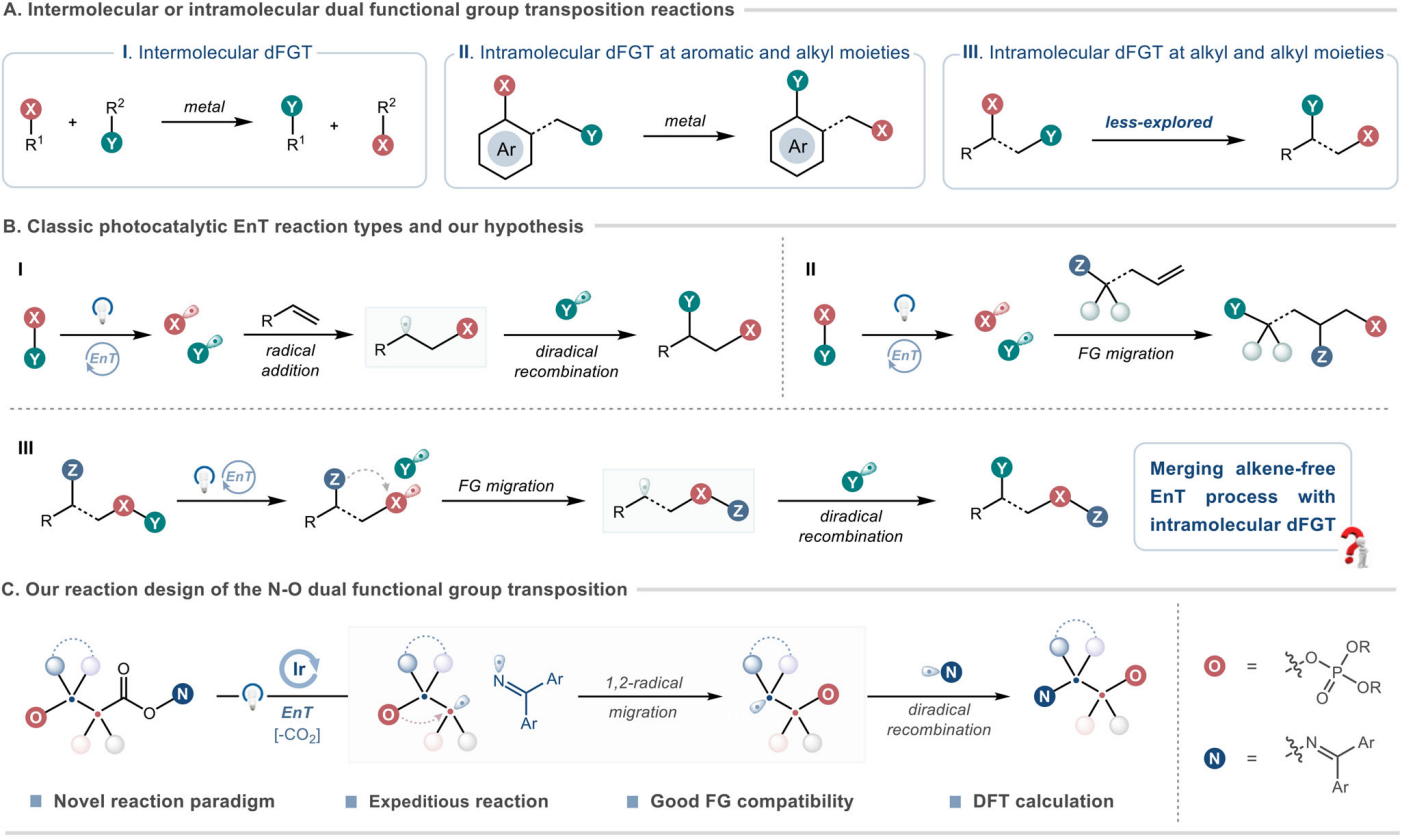
(A) Intermolecular or intramolecular dual functional group transposition reactions. (B) Classic photocatalytic EnT reaction types and our hypothesis. (C) Our reaction design of the N‐O dual functional group transposition.

Traditional dFGT strategies have largely focused on intermolecular reactions (Figure 1A, I), typically relying on metal‐catalyzed reversible oxidative addition and reductive elimination processes [15, 16, 17, 18, 19, 20, 21]. While recent reports have introduced metal‐free mediated systems [22, 23], intramolecular dFGT remains in its infancy. To date, an example of this transformation has been documented, leveraging synergistic photoredox/palladium catalysis to mediate aryl deiodination, 1,4‐boron migration, and iodine reinstallation, thereby enabling dual transposition across aromatic and alkyl sites (Figure 1A, II) [24]. However, achieving dual transposition between carbons of similar properties—particularly between two C(sp^3^) centers—remains an unexplored challenge, due to the lack of intrinsic electronic or steric differentiation between these positions (Figure 1A, III).

We envisioned addressing this gap through a photocatalytic energy transfer (EnT) process. EnT‐mediated transformations generally proceed through two pathways: the more common approach involves energy transfer generating two radicals with distinct lifetimes that sequentially add to an alkene acceptor, yielding a difunctionalized product (Figure 1B, I) [25, 26, 27, 28, 29, 30, 31, 32]. Alternatively, the short‐lived radical first adds to an acceptor and subsequently initiates a cascade, beginning with functional group migration and concluding with interception of the resultant intermediate by the persistent radical, ultimately delivering the final product (Figure 1B, II) [33, 34]. Notably, both EnT‐mediated strategies inherently rely on alkenes as radical acceptors to achieve dual functionalization, whereas our goal is to develop a novel, alkene‐free reaction paradigm. Such a strategy would eliminate the need for pre‐installed alkene acceptors, broaden substrate generality, and enable streamlined access to structurally diverse molecular scaffolds. Our designed mechanism entails EnT‐driven generation of a short‐lived radical intermediate, which undergoes intramolecular functional group migration prior to coupling with a persistent radical, thereby directly yielding the desired dFGT product (Figure 1B, III).

To realize this dFGT paradigm based on intramolecular radical migration‐coupling, we drew inspiration from the well‐established field of photocatalytic 1,2‐radical migrations, in which diverse functional group transpositions, including boryl, acyloxy, aryl, ester, and amino group—have been achieved [35, 36, 37, 38, 39, 40, 41, 42, 43, 44, 45, 46, 47, 48, 49, 50, 51]. However, their integration into 1,2‐dFGT remains largely unexplored. Building on our ongoing efforts in functional group transposition [51, 52], we leveraged radical property differences to design an unprecedented dual transposition between biologically relevant phosphatoxy and amino groups. As shown in Figure 1C, EnT between the designed substrate and a photosensitizer initiates a decarboxylative event, generating a transient β‐phosphatoxyalkyl radical along with a persistent iminyl radical. The β‐phosphatoxyalkyl radical subsequently undergoes a 1,2‐phosphatoxy migration, and the resulting carbon‐centered radical couples with the persistent iminyl radical to furnish the 1,2‐difunctional transposition product. This sequential radical generation‐migration‐rebonding cascade, enabled by the orthogonal reactivity of transient and persistent radicals, constitutes the core of our diradical‐mediated strategy, achieving N–O dual functional group transposition (dFGT) at both benzylic/aliphatic and unactivated aliphatic/aliphatic positions.

## 2 Results and Discussion

### 2.1 Reaction Evaluation and Optimization

**FIGURE 2:**
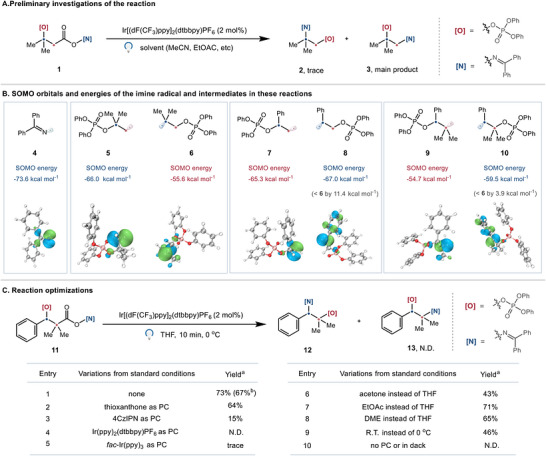
(A) Preliminary investigations of the reaction. (B) SOMO Orbitals and energies of the imine radical and intermediates in these reactions. (C) Reaction optimizations. Standard Reaction condition: 11 (0.025 mmol), Ir[(dF(CF_3_)ppy]_2_(dtbbpy)PF_6_ (2 mol%), THF (0.1 M), *λ*_max_ = 427 nm LEDs, N_2_, 10 min, 0°C. *^a^* Yield determined by ^1^H NMR with 3,4,5‐trichloropyridine as internal standard. *^b^* 0.2 mmol scale, 1 h, isolated yield is shown in parentheses. ^c^ *λ*_max_ = 390 nm LEDs. N.D., not detected. R.T., room temperature.

Our initial investigation commenced with the unactivated phosphatoxyalkyl oxime ester **1** (Figure 2A). Reaction screening employing various photocatalysts and solvents afforded only trace amounts of the dFGT product **2**, with **3** from the decarboxylative homocoupling as the major product. To gain molecular‐level insight into this observation, we performed density functional theory (DFT) calculations to analyze the singly occupied molecular orbital (SOMO) of the imine radical **4** and the radical intermediates before and after phosphatoxy migration. Following EnT between substrate **1** and the photocatalyst, decarboxylation generates the β‐phosphatoxyalkyl radical intermediate **5**, which subsequently undergoes phosphatoxy migration to form **6**. Radical‐radical coupling necessitates both orbital overlap and energy matching. Computational results revealed that although both **5** and **6** exhibit orbital overlap with **4**, the energy matching between **5** and **4** is superior to that between **6** and **4** (Figure 2B; consistent font color of SOMO indicates a better match). These SOMO energy calculations show that the competition between the desired dFGT product and the undesired homocoupling side product is directly related to the relative alignment of the singly occupied molecular orbitals of the key radical intermediates.

Consequently, in order to improve the orbital energy matching, the *gem*‐dimethyl group was substituted with a phenyl moiety, yielding the corresponding activated phosphatoxyalkyl oxime ester. Conjugative delocalization into the aromatic ring significantly stabilizes the post‐migration radical **8**, lowering its SOMO energy by ∼11.4 kcal mol^−1^ and thereby making species **4** better matched in SOMO energy with **8** than with the pre‐migration species **7** (Figure 2B). We further introduced steric hindrance at the β‐position of the phosphatoxy group to suppress homocoupling product formation. Consistently, SOMO energy analysis confirmed that **4** matches more favorably with the migrated species **10** than with its precursor **9** (Figure 2B).

Guided by these SOMO computational insights, we proceeded to optimize the reaction using substrate **11**. Satisfyingly, the system successfully afforded the dFGT product **12**, with no detectable homocoupling product **13**. The optimal conditions were identified as follows: irradiation with blue light at 0 °C in THF, using Ir[(dF(CF_3_)ppy]_2_(dtbbpy)PF_6_ as the photocatalyst for 10 min, yielding **12** in 73% yield (Figure 2C, entry 1). Scaling the reaction to 0.2 mmol maintained a 67% yield, with complete conversion achieved within 1 h. The use of other photocatalysts resulted in diminished yields (Figure 2C, entries 2–5), while solvent screening identified ether and ester solvents as optimal for this transformation (Figure 2C, entries 6–8). Elevating the temperature to room temperature led to a significant decrease in yield (Figure 2C, entry 9). Control experiments verified that both light and the photocatalyst are indispensable for the reaction (Figure 2C, entry 10).

### 2.2 Substrate Scope

**FIGURE 3:**
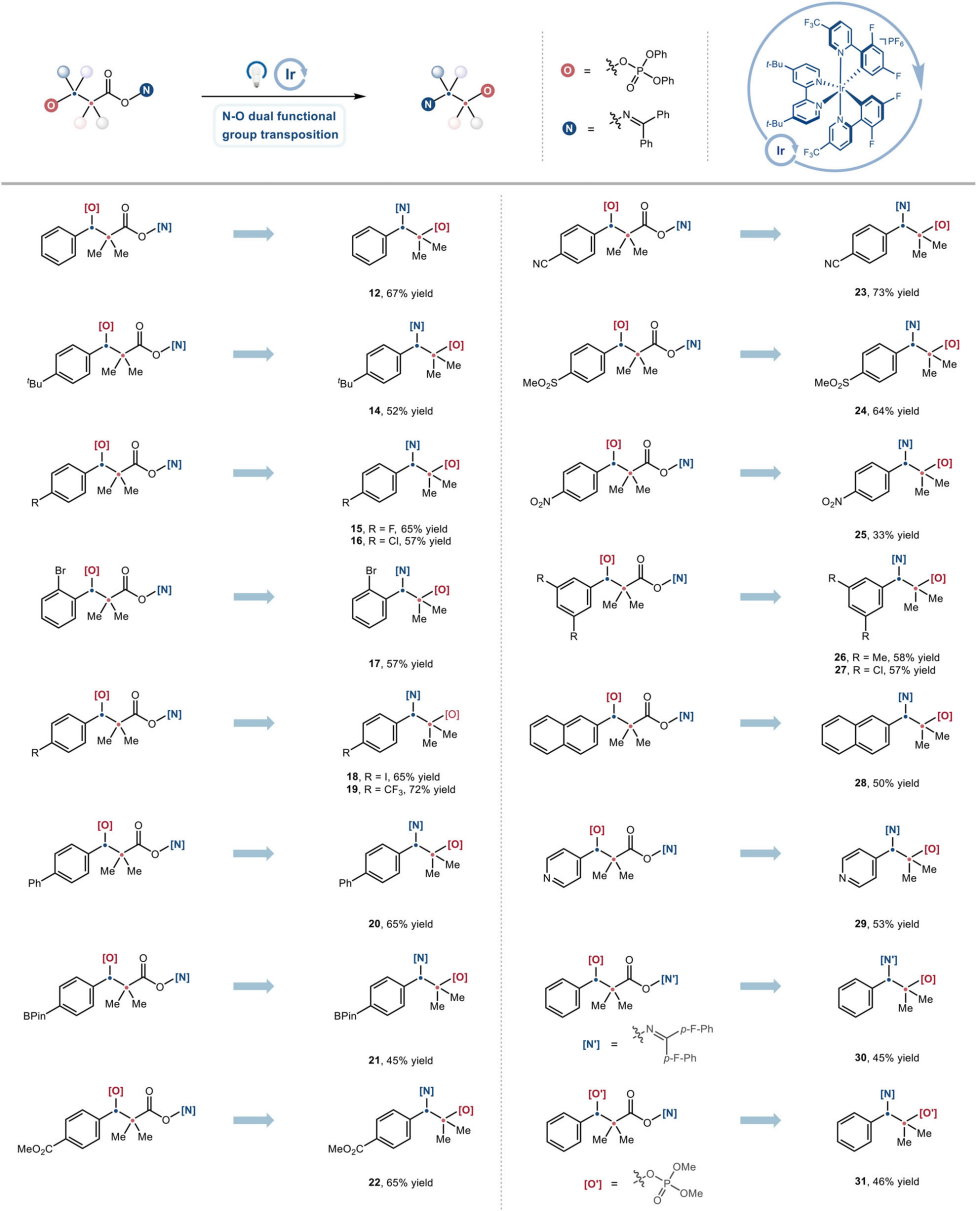
Scope of N‐O dual functional group transposition of activated phosphatoxyalkyl oxime esters. All yields are isolated yields. Reaction condition: activated phosphatoxyalkyl oxime esters (0.2 mmol), Ir[(dF(CF_3_)ppy]_2_(dtbbpy)PF_6_ (2 mol%), THF (0.1 M), *λ*_max_ = 427 nm LEDs, N_2_, 0°C, 1 h.

After establishing the optimized reaction conditions, we next investigated the substrate scope of the dFGT reaction to comprehensively evaluate its generality (Figure 3). Both electron‐donating alkyl groups (4‐*^t^* Bu) at various positions on the benzene ring and electron‐withdrawing halogen‐related substituents (including 4‐F, 4‐Cl, 2‐Br, 4‐I, and 4‐CF_3_) were well tolerated in this N–O dFGT reaction, affording the corresponding products in moderate to good yields (**14**–**19**, 52%–72% yield). Extension of the aromatic conjugation (e.g., 4‐Ph) also provided the rearrangement product in a satisfactory yield (**20**, 65% yield). Furthermore, the transformation proved compatible with the versatile functional group 4‐BPin, albeit with a slightly diminished yield (**21**, 45% yield). Substrates bearing moderately to strongly electron‐withdrawing groups (including 4‐CO_2_Me, 4‐CN, 4‐SO_2_Me) were also compatible with the reaction system, enabling efficient access to the desired products in good yields (**22**–**24**, 64%–73% yield). Notably, the nitro group, a strong electron‐withdrawing substituent that is often incompatible with most photoredox electron transfer processes, was readily accommodated under our EnT‐ catalyzed rearrangement system (**25**, 33% yield), further highlighting the synthetic utility of this transformation. Disubstituted arenes (3,5‐Me and 3,5‐Cl) were also viable substrates, yielding the corresponding products (26 and 27, 58% and 57% yield, respectively). In addition, the reaction system demonstrated good compatibility with extended aromatic systems, as evidenced by naphthalene and pyridine derivatives, which were smoothly converted to the desired products (**28** and **29**, 50% and 53% yield, respectively) We further evaluated the effect of the migratory functional group on the reaction efficiency, and found that both the difluorophenyl ketoimine group and dimethyl phosphatoxy afforded the target products in synthetically useful yields under the standard conditions (**30** and **31**, 45% and 46% yield, respectively).

**FIGURE 4:**
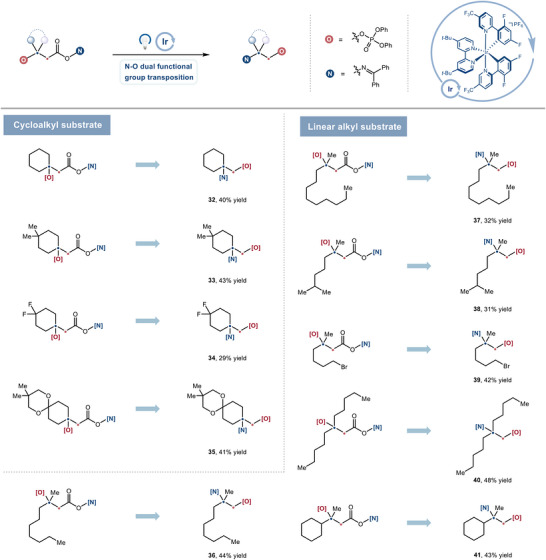
Scope of N‐O dual functional group transposition of unactivated phosphatoxyalkyl oxime esters. All yields are isolated yields. Reaction condition: unactivated phosphatoxyalkyl oxime esters (0.2 mmol), Ir[(dF(CF_3_)ppy]_2_(dtbbpy)PF_6_ (2 mol%), MeCN (0.1 M), *λ*_max_ = 427 nm LEDs, N_2_, R.T., 1 h.

Subsequently, we turned our attention to the seriously challenging unactivated phosphatoxyalkyl oxime ester system (Figure 4). We postulated that an increased electron density at the phosphatoxy substitutional α‐position would enhance the α–β electron density difference, thus accelerating phosphatoxy migration. Moreover, this electronic modulation was expected to concurrently stabilize the resulting radical intermediate and increase the likelihood of its coupling with the iminyl radical. Based on this rationale, we exchanged the *gem*‐dimethyl group in substrate **1** with a cyclohexyl group. After further optimization of the reaction conditions, the dFGT product **32** was obtained in a valuable yield (40% yield). It should be noted that the moderate yield is attributed to the difficulty in completely suppressing the decarboxylative homocoupling side reaction, along with a slight rise in imine homocoupling byproducts as the steric hindrance at the α‐position of the phosphatoxy group increases (See SI for details). The scope of the transformation was also extended to 4,4‐dimethylcyclohexyl, 4,4‐difluorocyclohexyl, and spirocyclic substrates, furnishing the corresponding products in serviceable yields (**33**–**35**, 29%–43% yield). Encouragingly, elongation of one alkyl chain in linear phosphatoxyalkyl oxime esters was also found to be feasible for the formation of the desired products (**36**–**39**, 31%–44% yield). Additionally, the concurrent elongation of both alkyl chains is further found to improve the efficacy of this transposition reaction (**40**, 48% yield). Besides, substrate bearing an α‐methyl cyclohexyl group also smoothly afforded the desired dFGT product with negligible generation of side byproducts (**41**, 43% yield). Unlike the aryl substrates in Figure 3 that stabilize carbon radicals via π‐conjugation to facilitate the final radical cross‐coupling, these aliphatic substrates lack delocalization channels for radical spin density. Their slow intramolecular migration favors competing homocoupling side reactions and ultimately leads to generally lower product yields.

### 2.3 Mechanism Investigations

**FIGURE 5:**
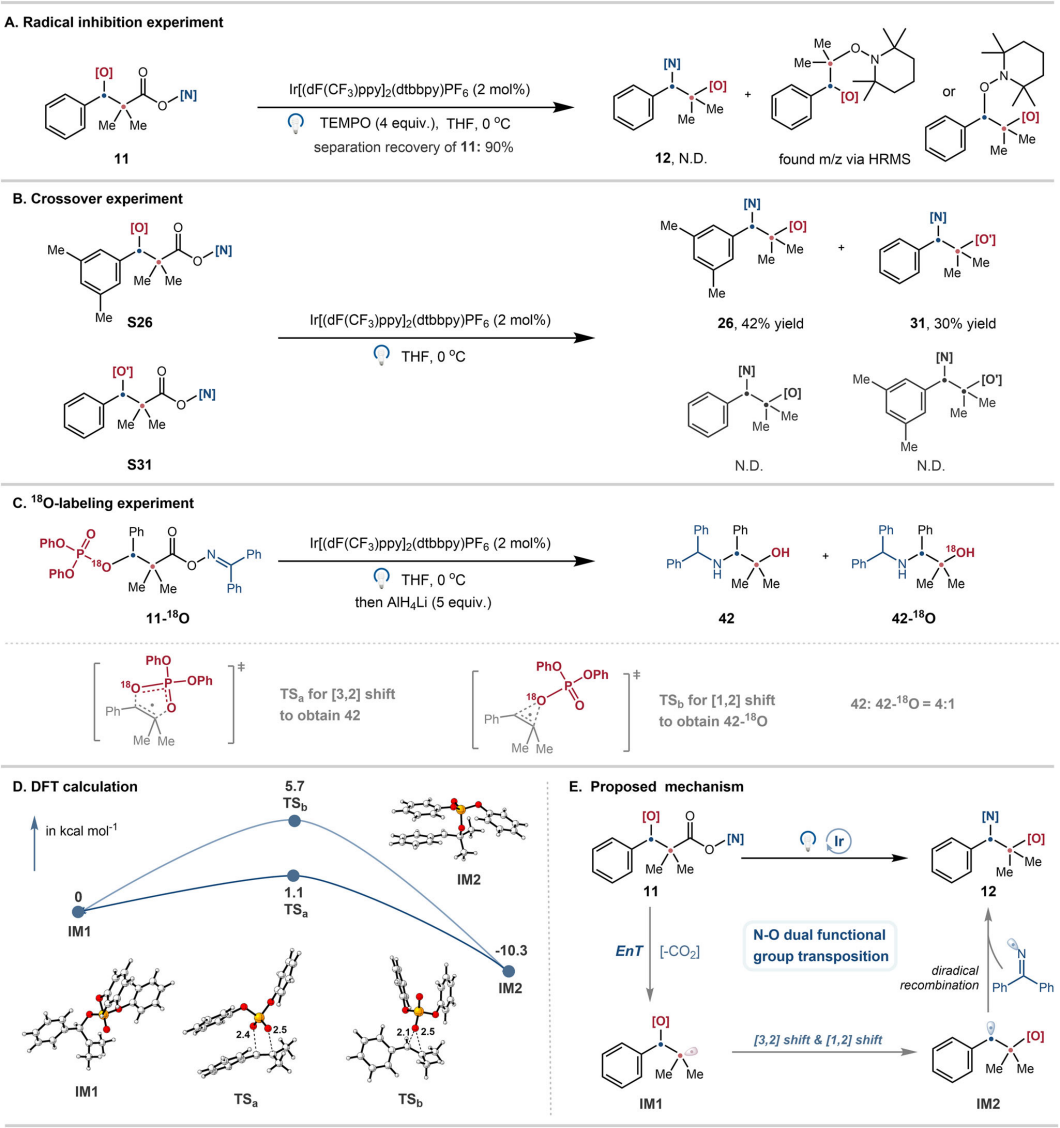
Mechanistic studies. (A) TEMPO‐radical inhibition experiment. (B) Crossover experiment. (C) ^18^O‐labeling experiment. (D) DFT calculation. (E) Proposed mechanism.

To gain deeper insights into the mechanism of the dFGT reaction, we conducted a combination of experimental and computational studies. As illustrated in Figure 5A, the addition of 4 equiv. of 2,2,6,6‐tetramethylpiperidinooxy (TEMPO) under standard conditions completely suppressed the formation of product **12,** and TEMPO‐captured products were detected via HRMS analysis, which indicates a radical nature for the dFGT process. We further performed a crossover experiment between substrates **S26** and **S31** (Figure 5B), affording products **26** and **31** in 42% and 30% yield, respectively. Significantly, no crossover products were detected, indicating that the 1,2‐phosphatoxy migration proceeds via an intramolecular concerted pathway. These findings rule out the release of a discrete phosphatoxy radical into the solvent and support a mechanism wherein migration is driven by the direct rearrangement of the original β‐phosphatoxyalkyl radical to a more stabilized counterpart.

An ^1^ ^8^O‐labeling experiment (Figure 5C) provided further insight into the migration pathway. When labeled **11**–^18^O was subjected to the standard reaction conditions, followed by reduction with 5 equiv. of LiAlH_4_, a 4:1 mixture of **42** and **42**‐^1^ ^8^O was obtained. This result confirms that phosphatoxy migration occurs via both [3,2]‐shift and [1,2]‐shift pathways, with the [3,2]‐shift being favored over the latter. DFT calculation corroborated the ^1^ ^8^O‐labeling results, revealing that the β‐phosphatoxyalkyl radical **IM1** undergoes rearrangement through a concerted five‐membered transition state **TS_a_**, which is 4.6 kcal·mol^−1^ lower in energy than the three‐membered transition state **TS_b_**, with both pathways ultimately leading to **IM2** (Figure 5D). Based on these findings, we propose the following mechanism for the dFGT reaction (Figure 5E): upon EnT with the photocatalyst, **11** undergoes decarboxylation to generate **IM1** and a persistent iminyl radical. **IM1** then undergoes 1,2‐phosphatoxy migration preferentially through a [3,2]‐shift, with a minor contribution from the [1,2]‐shift pathway, to form **IM2**. Finally, radical–radical coupling between **IM2** and the iminyl radical affords the dFGT product.

### 2.4 Reaction Derivations

**FIGURE 6:**
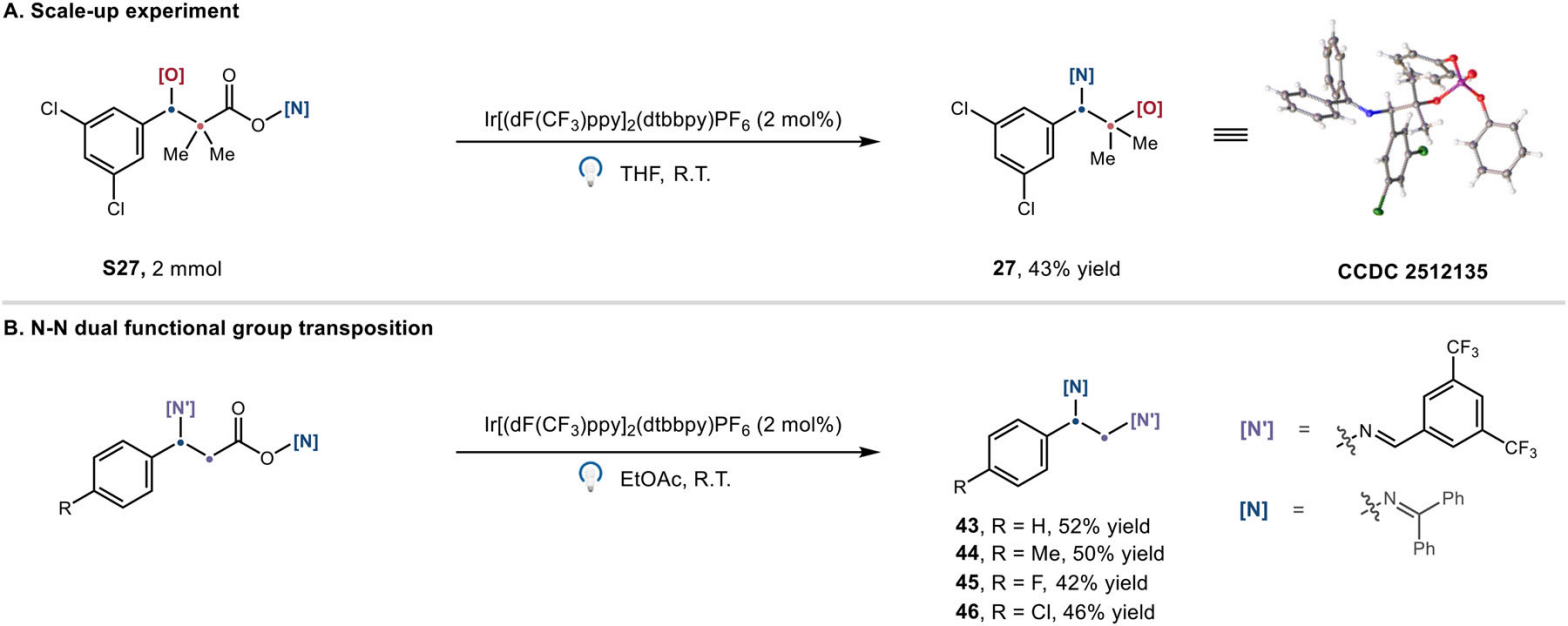
(A) Scale‐up experiment and X‐ray single crystal. (B) N–N dual functional group transposition.

We also performed a scale‐up reaction to demonstrate the synthetic utility. Under ambient conditions, **S27** (2 mmol) was smoothly converted to the corresponding product **27** in 43% yield, the structure of which was definitively established by X‐ray crystallographic analysis (Figure 6A). Given our group's earlier reports on 1,2‐amino migration [51], we sought to determine whether this strategy could be applied to substrates containing both aldimine and ketimine groups to accomplish an N‐ N dual‐functional‐group translocation (Figure 6B). Gratifyingly, the corresponding N–N dFGT products were isolated in moderate yields (**43**‐**46**, 42%–52%). In sharp contrast, aliphatic aldehyde imine substrates could not furnish the target migratory products, owing to their inherent extreme instability that renders these substrates difficult to synthesize. The differential behavior of aldimines and ketimines suggests that this strategy could offer a platform for the gradient synthesis and subsequent transformation of 1,2‐diamine scaffolds.

## 3 Conclusion

In summary, we have developed a novel dual‐functional group transposition via an energy‐transfer photocatalytic strategy. This approach unlocks dual functional group transposition of both activated and unactivated alkane derivatives, enabling precise skeletal remodeling and efficient assembly of structurally diverse molecules. Combined theoretical and experimental mechanistic investigations have clarified the reaction sequence of this photocatalytic energy transfer process, which proceeds via decarboxylative diradical formation, followed by 1,2‐phosphatoxy migration, and ultimately terminates with radical–radical coupling. Unlike classic systems where both functional groups translocate along the carbon backbone simultaneously, the phosphatoxy group first undergoes intramolecular 1,2‐rearrangement, while the iminyl radical is generated through decarboxylation and engages in the final radical cross‐coupling process, leading to a further rearrangement. Remarkably, this strategy proves effective not only in N–O transposition but can also be extended to N–N transposition. We anticipate that this reaction paradigm will offer broad applicability for the precise editing and transformation of diverse functional groups (Supporting Information).

## Author Contributions

**Lei Bao**: conceptualization, investigation, writing − original draft. **Xueyu Wang**: investigation. **Yang Zhou**: invastigation. **Beibei Zhan**: investigation, writing – original draft. **Xiaheng Zhang**: resources, supervision, conceptualization, writing – review and editing, writing – original draft.

## Funding

This work was financially supported by the Natural Science Foundation of Zhejiang Province (LR26B020002 and LDQ23B020001), the National Natural Science Foundation of China (22171049 and 22301054), the Hangzhou leading innovation and entrepreneurship team project (TD2022002), and the Optimal Funding of Postdoctoral Research Project of Zhejiang Province (ZJ2024091).

## Conflicts of Interest

The authors declare no conflicts of interest.

## Supporting information




**Supporting File**: advs76772‐sup‐0001‐SuppMat.pdf.

## Data Availability

CCDC 2512135 contains the supplementary crystallographic data for this paper. These data can be obtained free of charge from The Cambridge Crystallographic Data Centre via www.ccdc.cam.ac.uk/data_request/cif. The data that supports the findings of this study are available in the supplementary material of this article.

## References

[1] J. Jurczyk , J. Woo , S. F. Kim , B. D. Dherange , R. Sarpong , and M. D. Levin , “Single‐Atom Logic for Heterocycle Editing,” Nature Synthesis 1, no. 5 (2022): 352–364, 10.1038/s44160-022-00052-1. PMC9355079 35935106

[2] S. H. Kennedy , B. D. Dherange , K. J. Berger , and M. D. Levin , “Skeletal Editing through Direct Nitrogen Deletion of Secondary Amines,” Nature 593, no. 7858 (2021): 223–227, 10.1038/s41586-021-03448-9. 33981048

[3] J. Li , P. Tang , Y. Fan , and H. Lu , “Skeletal Editing of Pyrrolidines by Nitrogen‐Atom Insertion,” Science 389, no. 6757 (2025): 275–281, 10.1126/science.adl4755. 40674499

[4] Z. Liu , X. Zhang , P. Sivaguru , and X. Bi , “Triftosylhydrazone in Single‐Atom Skeletal Editing,” Accounts of Chemical Research 58, no. 1 (2025): 130–149, 10.1021/acs.accounts.4c00709. 39680057

[5] G. L. Bartholomew , S. L. Kraus , L. J. Karas , et al., “14 to 15N Isotopic Exchange of Nitrogen Heteroaromatics through Skeletal Editing,” Journal of the American Chemical Society 146, no. 5 (2024): 2950–2958, 10.1021/jacs.3c11515. 38286797 PMC11646074

[6] T. J. Pearson , R. Shimazumi , J. L. Driscoll , B. D. Dherange , D.‐I. Park , and M. D. Levin , “Aromatic Nitrogen Scanning by Ipso‐Selective Nitrene Internalization,” Science 381, no. 6665 (2023): 1474–1479, 10.1126/science.adj5331. 37769067 PMC10910605

[7] R. Al‐Ahmad and M. Dai , “Advancing Total Synthesis through Skeletal Editing,” Accounts of Chemical Research 58, no. 9 (2025): 1392–1406, 10.1021/acs.accounts.5c00030. 40209068 PMC12060283

[8] C. Ma , C. W. Lindsley , J. Chang , and B. Yu , “Rational Molecular Editing: A New Paradigm in Drug Discovery,” Journal of Medicinal Chemistry 67, no. 14 (2024): 11459–11466, 10.1021/acs.jmedchem.4c01347. 38905482

[9] Z. Wu , X. Xu , J. Wang , and G. Dong , “Carbonyl 1,2‐Transposition through Triflate‐Mediated α‐Amination,” Science 374, no. 6568 (2021): 734–740, 10.1126/science.abl7854. 34735246 PMC9125339

[10] S. Edelmann and J.‐P. Lumb , “A Para‐ to Meta‐Isomerization of Phenols,” Nature Chemistry 16, no. 7 (2024): 1193–1199, 10.1038/s41557-024-01512-1. 38632366

[11] Q. Zhu , J. M. Taylor , X. Liu , and G. Dong , “2‐Oxygen Transposition on Arenes Enabled by Palladium/Norbornene Cooperative Catalysis,” Journal of the American Chemical Society 147, no. 46 (2025): 43098–43104, 10.1021/jacs.5c16468. 41186675 PMC13180269

[12] R. T. Steele , M. Fujiu , and R. Sarpong , “1,2‐Acyl Transposition through Photochemical Skeletal Rearrangement of 2,3‐Dihydrobenzofurans,” Science 388, no. 6747 (2025): 631–638, 10.1126/science.adv9915. 40339026 PMC12594444

[13] X. Wu and C. Zhu , “Radical‐Mediated Remote Functional Group Migration,” Accounts of Chemical Research 53, no. 8 (2020): 1620–1636, 10.1021/acs.accounts.0c00306. 32706572

[14] S. Wang , X. Luo , Y. Wang , et al., “Radical‐Triggered Translocation of C–C Double Bond and Functional Group,” Nature Chemistry 16, no. 10 (2024): 1621–1629, 10.1038/s41557-024-01633-7. 39251841

[15] Z. Lian , B. N. Bhawal , P. Yu , and B. Morandi , “Palladium‐Catalyzed Carbon‐Sulfur or Carbon‐Phosphorus Bond Metathesis by Reversible Arylation,” Science 356, no. 6342 (2017): 1059–1063, 10.1126/science.aam9041. 28596362

[16] R. Isshiki , M. B. Kurosawa , K. Muto , and J. Yamaguchi , “Ni‐Catalyzed Aryl Sulfide Synthesis through an Aryl Exchange Reaction,” Journal of the American Chemical Society 143, no. 27 (2021): 10333–10340, 10.1021/jacs.1c04215. 34181399

[17] B. Mouhsine , M. Norlöff , J. Ghouilem , A. Sallustrau , F. Taran , and D. Audisio , “Platform for Multiple Isotope Labeling via Carbon–Sulfur Bond Exchange,” Journal of the American Chemical Society 146, no. 12 (2024): 8343–8351, 10.1021/jacs.3c14106. 38498972

[18] Y. H. Lee and B. Morandi , “Metathesis‐Active Ligands Enable a Catalytic Functional Group Metathesis between Aroyl Chlorides and Aryl Iodides,” Nature Chemistry 10, no. 10 (2018): 1016–1022, 10.1038/s41557-018-0078-8. 30082881

[19] M. De La Higuera Macias and B. A. Arndtsen , “Functional Group Transposition: A Palladium‐Catalyzed Metathesis of Ar–X σ‐Bonds and Acid Chloride Synthesis,” Journal of the American Chemical Society 140, no. 32 (2018): 10140–10144, 10.1021/jacs.8b06605. 30079726

[20] Z.‐Q. Lei , F. Pan , H. Li , et al., “Group Exchange between Ketones and Carboxylic Acids through Directing Group Assisted Rh‐Catalyzed Reorganization of Carbon Skeletons,” Journal of the American Chemical Society 137, no. 15 (2015): 5012–5020, 10.1021/ja512003d. 25843169

[21] T. Delcaillau , A. Bismuto , Z. Lian , and B. Morandi , “Nickel‐Catalyzed Inter‐ and Intramolecular Aryl Thioether Metathesis by Reversible Arylation,” Angewandte Chemie International Edition 59, no. 5 (2020): 2110–2114, 10.1002/anie.201910436. 31829493 PMC7004142

[22] J. Zhang , R. Wei , C. Ren , L. L. Liu , and L. Wu , “Si–B Functional Group Exchange Reaction Enabled by a Catalytic Amount of BH_3_: Scope, Mechanism, and Application,” Journal of the American Chemical Society 145, no. 28 (2023): 15619–15629, 10.1021/jacs.3c05625. 37411027

[23] A. Chen , Y.‐W. Zhang , J.‐Y. Lu , and D.‐W. Gao , “Selective Boron‐Heteroatom Functional Group Exchange Reactions,” CCS Chemistry 8, no. 5 (2026): 2375–2386, 10.31635/ccschem.025.202505879.

[24] M. Xu , C. Wu , and M. Chen , “Functional Group Transposition Enabled by Palladium and Photo Dual Catalysis,” Journal of the American Chemical Society 147, no. 44 (2025): 40058–40063, 10.1021/jacs.5c11429. 41144648 PMC12593406

[25] G. Tan , M. Das , R. Kleinmans , F. Katzenburg , C. Daniliuc , and F. Glorius , “Energy Transfer‐Enabled Unsymmetrical Diamination Using Bifunctional Nitrogen‐Radical Precursors,” Nature Catalysis 5, no. 12 (2022): 1120–1130, 10.1038/s41929-022-00883-3.

[26] G. Tan , M. Das , H. Keum , P. Bellotti , C. Daniliuc , and F. Glorius , “Photochemical Single‐Step Synthesis of β‐Amino Acid Derivatives from Alkenes and (hetero)Arenes,” Nature Chemistry 14, no. 10 (2022): 1174–1184, 10.1038/s41557-022-01008-w. 35915332

[27] J. E. Erchinger , R. Hoogesteger , R. Laskar , et al., “EnT‐Mediated N–S Bond Homolysis of a Bifunctional Reagent Leading to Aliphatic Sulfonyl Fluorides,” Journal of the American Chemical Society 145, no. 4 (2023): 2364–2374, 10.1021/jacs.2c11295. 36652725

[28] Q. H. Nguyen , H. S. Hwang , E. J. Cho , and S. Shin , “Energy Transfer Photolysis of N‐Enoxybenzotriazoles into Benzotriazolyl and α‐Carbonyl Radicals,” ACS Catalysis 12, no. 15 (2022): 8833–8840, 10.1021/acscatal.2c02862.

[29] Q. Sun , S.‐P. Wang , Y. Xu , et al., “Visible‐Light‐Induced Energy‐Transfer‐Mediated Hydrofunctionalization and Difunctionalization of Unsaturated Compounds via Sigma‐Bond Homolysis of Energy‐Transfer Acceptors,” ACS Catalysis 15, no. 3 (2025): 1854–1941, 10.1021/acscatal.4c07316.

[30] R. I. Rodríguez , M. Sicignano , M. J. García , R. G. Enríquez , S. Cabrera , and J. Alemán , “Taming Photocatalysis in Flow: Easy and Speedy Preparation of α‐Aminoamide Derivatives,” Green Chemistry 24, no. 17 (2022): 6613–6618, 10.1039/D2GC02087D.

[31] J. Majhi , R. K. Dhungana , Á. Rentería‐Gómez , et al., “Metal‐Free Photochemical Imino‐Alkylation of Alkenes with Bifunctional Oxime Esters,” Journal of the American Chemical Society 144, no. 34 (2022): 15871–15878, 10.1021/jacs.2c07170. 35984388 PMC10245625

[32] X.‐K. Qi , M.‐J. Zheng , C. Yang , Y. Zhao , L. Guo , and W. Xia , “Metal‐Free Amino(hetero)Arylation and Aminosulfonylation of Alkenes Enabled by Photoinduced Energy Transfer,” Journal of the American Chemical Society 145, no. 30 (2023): 16630–16641, 10.1021/jacs.3c04073. 37486736

[33] X.‐L. Luo , S.‐S. Li , Y.‐S. Jiang , F. Liu , S.‐H. Li , and P.‐J. Xia , “Photocatalytic 1,2‐Iminosulfonylation and Remote 1,6‐Iminosulfonylation of Olefins,” Organic Letters 25, no. 10 (2023): 1742–1747, 10.1021/acs.orglett.3c00437. 36883883

[34] Y. Zheng , Z. Liao , Z. Xie , et al., “Photochemical Alkene Trifluoromethylimination Enabled by Trifluoromethylsulfonylamide as a Bifunctional Reagent,” Organic Letters 25, no. 12 (2023): 2129–2133, 10.1021/acs.orglett.3c00577. 36943094

[35] X. Wu , Z. Ma , T. Feng , and C. Zhu , “Radical‐Mediated Rearrangements: Past, Present, and Future,” Chemical Society Reviews 50, no. 20 (2021): 11577–11613, 10.1039/D1CS00529D. 34661216

[36] G. Zhao , U. Mukherjee , W. Yao , and M.‐Y. Ngai , “Catalytic 1,2‐Radical Acyloxy Migration: A Strategy to Access Novel Chemical Space and Reaction Profiles,” Accounts of Chemical Research 58, no. 11 (2025): 1815–1829, 10.1021/acs.accounts.5c00205. 40402008 PMC12862922

[37] Z. Li , M. Wang , and Z. Shi , “Radical Addition Enables 1,2‐Aryl Migration from a Vinyl‐Substituted all‐Carbon Quaternary Center,” Angewandte Chemie International Edition 60, no. 1 (2021): 186–190, 10.1002/anie.202010839. 32914547

[38] G. Zhao , S. Lim , D. G. Musaev , and M.‐Y. Ngai , “Expanding Reaction Profile of Allyl Carboxylates via 1,2‐Radical Migration (RaM): Visible‐Light‐Induced Phosphine‐Catalyzed 1,3‐Carbobromination of Allyl Carboxylates,” Journal of the American Chemical Society 145, no. 15 (2023): 8275–8284, 10.1021/jacs.2c11867. PMC11694480 37017987

[39] Y. Guo , X. Wang , C. Li , J. Su , J. Xu , and Q. Song , “Decarboxylation of β‐Boryl NHPI Esters Enables Radical 1,2‐Boron Shift for the Assembly of Versatile Organoborons,” Nature Communications 14, no. 1 (2023): 5693, 10.1038/s41467-023-41254-1. PMC10502150 37709736

[40] D. Kaiser , A. Noble , V. Fasano , and V. K. Aggarwal , “1,2‐Boron Shifts of β‐Boryl Radicals Generated from Bis‐Boronic Esters Using Photoredox Catalysis,” Journal of the American Chemical Society 141, no. 36 (2019): 14104–14109, 10.1021/jacs.9b07564. 31461622 PMC7610657

[41] M. Kischkewitz , F. W. Friese , and A. Studer , “Radical‐Induced 1,2‐Migrations of Boron Ate Complexes,” Advanced Synthesis & Catalysis 362, no. 11 (2020): 2077–2087, 10.1002/adsc.201901503. 32612487 PMC7319355

[42] Y. Li , B. Liu , H.‐B. Li , Q. Wang , and J.‐H. Li , “Oxidative Radical 1,2‐Alkylarylation of Alkenes with α‐C(sp 3 )–H Bonds of Acetonitriles Involving 1,2‐Aryl Migration,” Chemical Communications 51, no. 6 (2015): 1024–1026, 10.1039/C4CC08902B. 25446150

[43] S. Patra , S. Das , S. Nandi , H. Khatua , and B. Chattopadhyay , “Catalyst‐Enabled Chemoselective Metalloradical Activation for Molecular Rearrangement via Ester Migration and Allylic C(sp3)–H Amination,” Journal of the American Chemical Society 147, no. 34 (2025): 30582–30590, 10.1021/jacs.5c07955. 40811647

[44] F. Bu , L. Lu , X. Hu , S. Wang , H. Zhang , and A. Lei , “Electrochemical Oxidative Decarboxylation and 1,2‐Aryl Migration towards the Synthesis of 1,2‐Diaryl Ethers,” Chemical Science 11, no. 36 (2020): 10000–10004, 10.1039/D0SC03708G. 34094264 PMC8162141

[45] A. Bunescu , Q. Wang , and J. Zhu , “Copper‐Catalyzed Cyanomethylation of Allylic Alcohols with Concomitant 1,2‐Aryl Migration: Efficient Synthesis of Functionalized Ketones Containing an α‐Quaternary Center,” Angewandte Chemie International Edition 54, no. 10 (2015): 3132–3135, 10.1002/anie.201411657. 25598160

[46] X. Ren , Y. Qiao , B. Zhou , S. Liu , and Y. Huang , “Nickel‐Catalysed Enantioselective Migratory Reductive Cross‐Coupling Enabled by Radical 1,2‐Amino Migration,” Nature Synthesis 4, no. 10 (2025): 1232–1246, 10.1038/s44160-025-00840-5.

[47] Z. Liao , Z. Li , M. Xiao , et al., “Asymmetric Synthesis of β‐Amino Acid Derivatives by Stereocontrolled C(sp^3^)‐C(sp^2^) Cross‐Electrophile Coupling via Radical 1,2‐Nitrogen Migration,” Nature Communications 16, no. 1 (2025): 7218, 10.1038/s41467-025-62092-3. PMC12325796 40764297

[48] A. L. J. Beckwith , D. Crich , P. J. Duggan , and Q. Yao , “Chemistry of β‐(Acyloxy)alkyl and β‐(Phosphatoxy)alkyl Radicals and Related Species: Radical and Radical Ionic Migrations and Fragmentations of Carbon−Oxygen Bonds,” Chemical Reviews 97, no. 8 (1997): 3273–3312, 10.1021/cr950207o. 11851491

[49] D. Crich , Q. Yao , and G. F. Filzen , “Chemistry of .beta.‐(Phosphatoxy)alkyl and .Beta.‐(Acyloxy)alkyl Radicals. Migration Reactions: Scope and Stereoselectivity of .beta.‐(Phosphatoxy)alkyl Rearrangement. Mechanism of .beta.‐(Phosphatoxy)alkyl and .Beta.‐(Acyloxy)alkyl Migration,” Journal of the American Chemical Society 117, no. 46 (1995): 11455–11470, 10.1021/ja00151a009.

[50] H. Huang , Z.‐Y. Yu , L.‐Y. Han , et al., “N‐Heterocyclic Carbene Catalytic 1,2‐Boron Migrative Acylation Accelerated by Photocatalysis,” Science Advances 10, no. 30 (2024): adn8401, 10.1126/sciadv.adn8401. PMC11268412 39047096

[51] W. Fan , Y. Cui , B. Zhan , et al., “Biomimetic 1,2‐Amino Migration via Photoredox Catalysis,” Nature Chemistry 17, no. 6 (2025): 941–951, 10.1038/s41557-025-01775-2. PMC12141034 40055577

[52] L. Bao , Y. Zhou , B. Zhan , Y. Liang , and X. Zhang , “Photocatalytic Alkyl‐To‐Aryl Amino Migration,” ACS Catalysis 16 (2026): 9447–9457, 10.1021/acscatal.6c01718.

