# Supplementary material for: N–O Dual Functional Group Transposition via Energy Transfer Photocatalysis
Source: Adv Sci (Weinh). 2026 Jul 23:e76772. Online ahead of print. doi: 10.1002/advs.76772 (PMC13395387; doi:10.1002/advs.76772)

N–O dual functional group transposition via energy transfer photocatalysis

Lei Bao[1†], Xueyu Wang[2†], Yang Zhou[1], Beibei Zhan[1]*, and Xiaheng Zhang[1]*

[1]School of Chemistry and Materials Science, Hangzhou Institute for Advanced Study, University of Chinese Academy of Sciences, Hangzhou, China. [2]College of Geography and Environmental Sciences, Zhejiang Normal University, Jinhua, China.

**Corresponding author. Email: xiahengz@ucas.ac.cn. [†]These authors contributed equally.*

Supporting Information

Table of Contents

1) General Information S3

2) Preparation of Starting Materials S4

3) Reaction Investigations and Optimizations S31

4) Experiment Procedure of N-O Dual Functional Group Transposition Reaction S35

5) Experimental Data S36

6) Reaction By-products and Limitations S60

7) Mechanism Experiment S67

8) DFT Calculation S74

9) Gram-scale Reaction and X-ray Diffraction Single Crystal S93

10) Experiment of N-N Dual Functional Group Transposition Reaction S95

11) References S104

12) Spectra Data S105

1) General Information

Commercial reagents were purchased from Adamas-beta, Bidepharm, J&K, TCI. Organic solutions were concentrated under reduced pressure on a Büchi rotary evaporator using a water bath. Chromatographic purification of products was accomplished using forced-flow chromatography on silica gel (Fluka, 230–400 mesh) according to the method of Still. Thin-layer chromatography (TLC) was performed on Huanghai 0.25 mm silica gel F-254 plates. Visualization of the developed chromatogram was performed by fluorescence quenching or phosphomolybdic acid chromogenic reagent stain. ^{1}H NMR spectra were recorded on a Bruker UltraShield Plus Avance III 400 MHz and are internally referenced to residual protic $CDCl_3$ (7.26 ppm), CD_2Cl_2 (5.32 ppm) and C_6D_6 (7.16 ppm) (Data for ^{1}H NMR are reported as follows: chemical shift (δ ppm), multiplicity (s = singlet, d = doublet, t = triplet, q = quartet, m = multiplet, dd = doublet of doublets, dt = doublet of triplets, ddd = doublet of doublet of doublets), coupling constant (Hz), and integration. ^{13}C NMR spectra were recorded on a Bruker UltraShield Plus Avance III 400 MHz (100 MHz) or 600 MHz (150 MHz) and data are reported in terms of chemical shift relative to $CDCl_3$ (77.16 ppm), CD_2Cl_2 (53.84 ppm) and C_6D_6 (128.06 ppm). ^{31}P NMR and ^{19}F NMR spectra were recorded on a Bruker UltraShield Plus Avance III 400 MHz (160 MHz) and 600 MHz (376 MHz). High-Resolution Mass Spectra were obtained on Thermo Fisher Exactive Plus Orbitrap Mass Spectrum (ESI).

2) Preparation of Starting Materials

General Procedure A

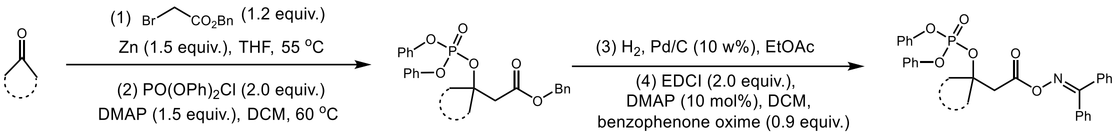

Reformatsky reaction. To a solution of ketone (1.0 equiv.) in dry THF (0.25 M), benzyl 2-bromoacetate (1.2 equiv.) and activated Zn (1.5 equiv.) were added in a nitrogen atmosphere. The reaction mixture was stirred for 5 h at 55 °C. Then the reaction mixture was filtered, the filtrate was concentrated and purified through flash column chromatography to obtain the benzyl hydroxyalkyl ester (**a1**). *Synthesis of phosphatoxy compound.* To a solution of **a1** (1.0 equiv.) in DCM (0.25 M), diphenyl chlorophosphate (2.0 equiv.) and DMAP (1.5 equiv.) were added. The reaction mixture was stirred for 12 h at 60 °C. The reaction was quenched with water, extracted with EtOAc, and the organic phases were combined, dried, concentrated, and purified through flash column chromatography to obtain the phosphatoxy compound (**a2**).

Debenzylation reaction. To a solution of **a2** (1.0 equiv.) in EtOAc (0.5 M), Pd/ C (10 w%) was added, and a hydrogen balloon (1 atm) was equipped. The reaction mixture was stirred for 2 h at room temperature. Then the reaction mixture was filtered and concentrated to obtain the crude phosphatoxy alkyl carboxylic acid (**a3**). *Condensation reaction*. To a solution of **a3** (1.0 equiv.) in DCM (0.25 M), EDCI (2.0 equiv.) and DMAP (1.5 equiv.) were added to mix well, and benzophenone oxime (0.9 equiv.) was subsequently added. The reaction mixture was stirred for 12 h at room temperature. The reaction was quenched with water, extracted with EtOAc, and the organic phases were combined, dried, concentrated, and purified through column chromatography to obtain the final product.

General Procedure B

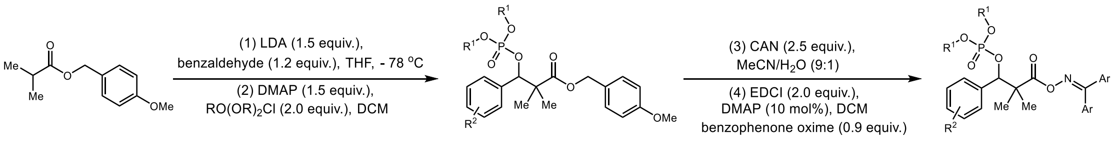

Synthesis of Phosphatoxy compound. To a solution of 4-methoxybenzyl isobutyrate (1.0 equiv.) in dry THF (0.25 M), LDA (2 M in THF, 1.2 equiv.) was added in a nitrogen atmosphere at -78 °C. After stirring for 20 minutes, benzaldehyde (1.2 equiv.) was added to the mixture. The reaction mixture was stirred for 1 h at -78 °C. The reaction was quenched with water, extracted with EtOAc, and the organic phases were combined, dried, concentrated and purified through flash column chromatography to obtain the benzyl hydroxyalkyl ester (**b1**). *Synthesis of phosphatoxy compound.* To a solution of **a1** (1.0 equiv.) in DCM (0.25 M), diphenyl chlorophosphate (2.0 equiv.) and DMAP (1.5 equiv.) were added. The reaction mixture was stirred for 12 h at room temperature. The reaction was quenched with water, extracted with EtOAc, and the organic phases were combined, dried, concentrated, and purified through flash column chromatography to obtain the phosphatoxy compound (**b2**).

Debenzylation reaction. To a solution of **b2** (1.0 equiv.) in MeCN/H_2O (9:1, 0.1 M), CAN (2.5 equiv.) was added. The reaction mixture was stirred for 3 h at room temperature. Then the reaction mixture was quenched with saturated $NaHSO_3$ aqueous solution, and vigorously stirred for another 10 minutes. Then the mixture was extracted with EtOAc, and the organic phases were combined, dried and concentrated to obtain the crude phosphatoxy alkyl carboxylic acid (**b3**). *Condensation reaction.* To a solution of **b3** (1.0 equiv.) in DCM (0.25 M), EDCI (2.0 equiv.) and DMAP (1.5 equiv.) were added to mix well, and benzophenone oxime (0.9 equiv.) was subsequently added. The reaction mixture was stirred for 12 h at room temperature. The reaction was quenched with water, extracted with EtOAc, and the organic phases were combined, dried, concentrated, and purified through column chromatography to obtain the final product.

4-(((Diphenylmethylene)amino)oxy)-2-methyl-4-oxobutan-2-yl diphenyl phosphate (1)

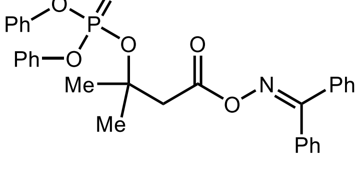

Prepared following the general procedure A outlined above starting from acetone and benzyl 2-bromoacetate on 20 mmol scale to give **1** (4.0 g, 38% total yield) as a colorless oil.

^{1}H NMR (400 MHz, CDCl$_3$) δ 7.62 – 7.59 (m, 2H), 7.49 – 7.43 (m, 4H), 7.40 – 7.26 (m, 8H), 7.23 – 7.14 (m, 6H), 2.83 (s, 2H), 1.64 (s, 6H).

^{13}C NMR (100 MHz, CDCl$_3$) 166.72, 165.31, 150.64 (d, J = 7.4 Hz), 134.52, 132.48, 131.09, 129.73, 129.69, 129.05, 128.64, 128.45, 128.31, 125.19, 120.19 (d, J = 4.9 Hz), 84.55 (d, J = 7.1 Hz), 45.61 (d, J = 6.5 Hz), 27.60, 27.57.

^{31}P NMR (160 MHz, CDCl$_3$) δ -16.95.

HRMS (ESI-TOF) m/z calcd. for $C_{30}H_{29}NO_6P^+$ ([M+H]$^+$) 530.1727, found: 530.1725.

Methyl (*E*)-3-(2-bromophenyl)-2-methyl-2-((1-phenylethylidene)amino)propanoate (11)

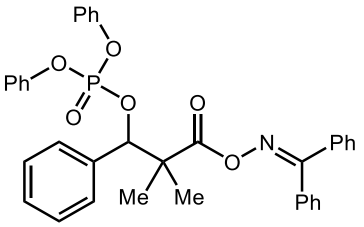

Prepared following the general procedure B outlined above starting from 4-methoxybenzyl isobutyrate and benzaldehyde on 20 mmol scale to give **11** (3.8 g, 31% total yield) as a white solid.

^{1}H NMR (400 MHz, CDCl$_3$) δ 7.57 (d, J = 7.8 Hz, 2H), 7.48 – 7.33 (m, 7H), 7.26 – 7.19 (m, 8H), 7.16 – 7.04 (m, 6H), 6.96 (d, J = 7.8 Hz, 2H), 5.70 (d, J = 8.0 Hz, 1H), 1.19 (s, 3H), 0.94 (s, 3H).

^{13}C NMR (100 MHz, $CDCl_3$) δ 171.66, 165.79, 150.64 (d, *J* = 2.8 Hz), 150.57 (d, *J* = 2.7 Hz), 135.79, 134.47, 132.61, 131.11, 129.74, 129.68, 129.57, 129.17, 128.66, 128.46, 128.25, 128.03 (d, *J* = 7.9 Hz), 127.92, 125.21 (d, *J* = 7.9 Hz), 120.28 (d, *J* = 4.9 Hz), 120.09 (d, *J* = 5.0 Hz), 85.54 (d, *J* = 6.4 Hz), 48.48 (d, *J* = 8.7 Hz), 20.75, 20.62.

^{31}P NMR (160 MHz, $CDCl_3$) δ -12.99 (d, *J* = 7.8 Hz).

HRMS (ESI-TOF) m/z calcd. for $C_{36}H_{33}NO_6P^+$ ($[M+H]^+$) 606.2040, found: 606.2038.

1-(4-(Tert-butyl)phenyl)-3-(((diphenylmethylene)amino)oxy)-2,2-dimethyl-3-oxopropyl diphenyl phosphate (S14)

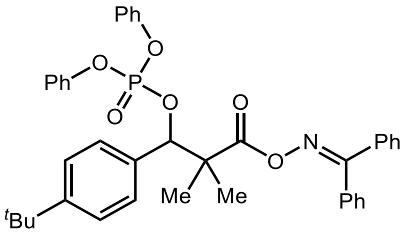

Prepared following the general procedure B outlined above starting from 4-methoxybenzyl isobutyrate and 4-(tert-butyl)benzaldehyde on 20 mmol scale to give **S14** (2.9 g, 22% total yield) as a white solid.

^{1}H NMR (400 MHz, CD_2Cl_2) δ 7.60 – 7.54 (m, 2H), 7.52 – 7.37 (m, 7H), 7.32 (dd, *J* = 8.5, 2.0 Hz, 2H), 7.28 – 7.22 (m, 3H), 7.20 – 7.06 (m, 8H), 6.90 (d, *J* = 8.4 Hz, 2H), 5.63 (d, *J* = 7.9 Hz, 1H), 1.32 (s, 9H), 1.15 (s, 3H), 0.95 (s, 3H).

^{13}C NMR (100 MHz, CD_2Cl_2) δ 171.84, 165.87, 152.06, 150.93 (d, *J* = 6.5 Hz), 150.86 (d, *J* = 6.8 Hz), 134.89, 132.93 (d, *J* = 16.8 Hz), 131.37, 130.01, 129.92, 129.87, 129.22, 128.96, 128.80, 128.54, 128.11, 125.52 (d, *J* = 7.9 Hz), 125.20, 120.52 (d, *J* = 4.8 Hz), 120.36 (d, *J* = 4.9 Hz), 85.83 (d, *J* = 6.3 Hz), 48.61 (d, *J* = 8.8 Hz), 34.86, 31.42, 21.06, 20.32.

^{31}P NMR (160 MHz, CD_2Cl_2) δ -12.54 (d, *J* = 7.7 Hz).

HRMS (ESI-TOF) m/z calcd. for $C_{40}H_{41}NO_6P^+$ ([M+H]$^+$) 662.2666, found: 662.2664.

3-(((Diphenylmethylene)amino)oxy)-1-(4-fluorophenyl)-2,2-dimethyl-3-oxopropyl diphenyl phosphate (S15)

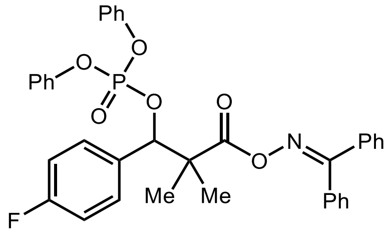

Prepared following the general procedure B outlined above starting from 4-methoxybenzyl isobutyrate and 4-fluorobenzaldehyde on 20 mmol scale to give **S15** (5.0 g, 40% total yield) as a white solid.

^{1}H NMR (400 MHz, CD_2Cl_2) δ 7.55 (d, *J* = 7.3 Hz, 2H), 7.49 – 7.36 (m, 7H), 7.31 – 7.20 (m, 5H), 7.17 – 7.10 (m, 6H), 6.91 – 6.98 (m, 4H), 5.64 (d, *J* = 8.1 Hz, 1H), 1.13 (s, 3H), 0.92 (s, 3H).

^{13}C NMR (100 MHz, CD_2Cl_2) δ 171.64, 166.09, 163.11 (d, *J* = 255.5 Hz), 150.88 (d, *J* = 6.5 Hz), 150.79 (d, *J* = 6.9 Hz), 134.78, 132.81, 132.00 (d, *J* = 3.1 Hz), 131.43, 130.08 (d, *J* = 9.0 Hz), 130.04 (d, *J* = 7.0 Hz), 129.91, 129.21, 128.89, 128.81, 128.54, 125.63 (d, *J* = 8.5 Hz), 120.47 (d, *J* = 4.9 Hz), 120.27 (d, *J* = 5.0 Hz), 115.14 (d, *J* = 21.7 Hz), 85.05 (d, *J* = 6.2 Hz), 48.58 (d, *J* = 8.4 Hz), 20.93, 20.32.

^{31}P NMR (160 MHz, CD_2Cl_2) δ -12.63 (d, *J* = 8.0 Hz).

^{19}F NMR (376 MHz, CD_2Cl_2) δ -113.79 – -113.87 (m).

HRMS (ESI-TOF) m/z calcd. for $C_{36}H_{32}FNO_6P^+$ ([M+H]$^+$) 624.1946, found: 624.1943.

1-(4-Chlorophenyl)-3-(((diphenylmethylene)amino)oxy)-2,2-dimethyl-3-oxopropyl diphenyl phosphate (S16)

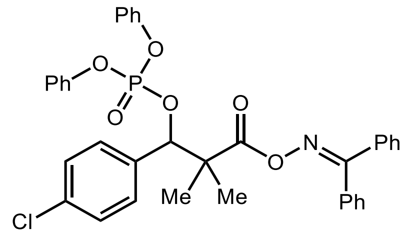

Prepared following the general procedure B outlined above starting from 4-methoxybenzyl isobutyrate and 4-chlorobenzaldehyde on 20 mmol scale to give **S16** (6.2 g, 40% total yield) as a white solid.

^{1}H NMR (400 MHz, CD_2Cl_2) δ 7.55 (d, *J* = 6.6 Hz, 2H), 7.50 – 7.37 (m, 6H), 7.32 – 7.08 (m, 14H), 6.96 (d, *J* = 8.3 Hz, 2H), 5.63 (d, *J* = 13.6 Hz, 1H), 1.12 (s, 3H), 0.92 (s, 3H).

^{13}C NMR (100 MHz, CD_2Cl_2) δ 171.54, 166.14, 150.85 (d, *J* = 7.2 Hz), 150.75 (d, *J* = 7.5 Hz), 134.78, 134.77, 134.72 (d, *J* = 4.7 Hz), 132.77, 131.45, 130.09, 130.03, 129.62, 129.21, 128.85, 128.81, 128.54, 128.41, 125.66 (d, *J* = 8.1 Hz), 120.46 (d, *J* = 4.4 Hz), 120.26 (d, *J* = 4.8 Hz), 84.95 (d, *J* = 6.6 Hz), 48.54 (d, *J* = 8.0 Hz), 20.81, 20.43.

^{31}P NMR (160 MHz, CD_2Cl_2) δ -12.65 (d, *J* = 7.6 Hz).

HRMS (ESI-TOF) m/z calcd. for $C_{36}H_{32}ClNO_6P^+$ ($[M+H]^+$) 640.1651, found: 640.1648.

1-(2-Bromophenyl)-3-(((diphenylmethylene)amino)oxy)-2,2-dimethyl-3-oxopropyl diphenyl phosphate (S17)

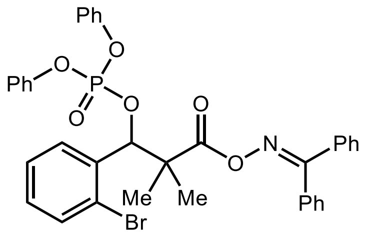

Prepared following the general procedure B outlined above starting from 4-methoxybenzyl isobutyrate and 2-bromobenzaldehyde on 10 mmol scale to give **S17** (2.6 g, 38% total yield) as a white solid.

^{1}H NMR (400 MHz, CD_2Cl_2) δ 7.54 – 7.35 (m, 10H), 7.27 – 7.04 (m, 12H), 7.02 – 6.95 (m, 2H), 6.27 (d, J = 7.8 Hz, 1H), 1.18 (s, 3H), 1.06 (s, 3H).

^{13}C NMR (100 MHz, CD_2Cl_2) δ 171.59, 165.51, 150.83 (d, J = 3.0 Hz), 150.76 (d, J = 3.0 Hz), 135.42, 135.01, 133.02 (d, J = 8.2 Hz), 131.26, 130.68, 130.56, 129.99, 129.68, 129.16, 128.89, 128.72 (d, J = 3.5 Hz), 127.44, 125.54, 124.46, 120.40 (d, J = 4.7 Hz), 120.36 (d, J = 4.7 Hz), 83.02 (d, J = 6.5 Hz), 49.21 (d, J = 8.8 Hz), 22.67, 18.44.

^{31}P NMR (160 MHz, CD_2Cl_2) δ -13.21 (d, J = 7.7 Hz).

HRMS (ESI-TOF) m/z calcd. for $C_{36}H_{32}BrNO_6P^+$ ($[M+H]^+$) 684.1145, found: 684.1136.

3-(((Diphenylmethylene)amino)oxy)-1-(4-iodophenyl)-2,2-dimethyl-3-oxopropyl diphenyl phosphate (S18)

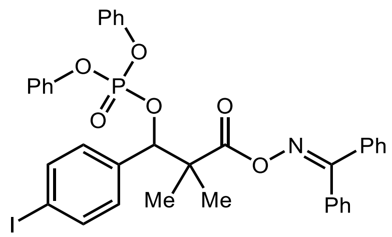

Prepared following the general procedure B outlined above starting from 4-methoxybenzyl isobutyrate and 4-iodobenzaldehyde on 10 mmol scale to give **S18** (2.9 g, 40% total yield) as a white solid.

^{1}H NMR (400 MHz, $CDCl_3$) δ 7.59 – 7.52 (m, 4H), 7.47 – 7.34 (m, 6H), 7.26 – 7.19 (m, 4H), 7.16 – 7.01 (m, 6H), 6.97 (d, *J* = 8.6 Hz, 2H), 6.87 (d, *J* = 8.4 Hz, 2H), 5.61 (d, *J* = 8.2 Hz, 1H), 1.16 (s, 3H), 0.92 (s, 3H).

^{13}C NMR (100 MHz, $CDCl_3$) δ 171.39, 166.08, 150.52 (d, *J* = 8.0 Hz), 150.45 (d, *J* = 7.1 Hz), 137.10, 135.57, 134.30, 132.53, 131.23, 129.81, 129.78, 129.76, 129.64, 129.16, 128.52 (d, *J* = 3.1 Hz), 128.30, 125.35 (d, *J* = 7.5 Hz), 120.20 (d, *J* = 4.9 Hz), 120.00 (d, *J* = 5.0 Hz), 94.68, 84.83 (d, *J* = 6.4 Hz), 48.35 (d, *J* = 8.5 Hz), 21.10, 20.19.

^{31}P NMR (160 MHz, $CDCl_3$) δ -12.99 (d, *J* = 8.1 Hz).

HRMS (ESI-TOF) m/z calcd. for $C_{36}H_{32}INO_6P^+$ ($[M+H]^+$) 732.1007, found: 732.1006.

3-(((Diphenylmethylene)amino)oxy)-2,2-dimethyl-3-oxo-1-(4-(trifluoromethyl)phenyl)propyl diphenyl phosphate (S19)

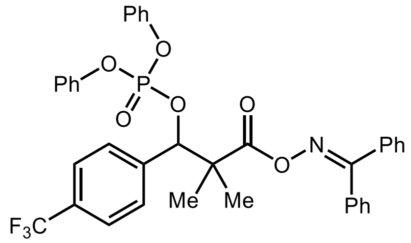

Prepared following the general procedure B outlined above starting from 4-methoxybenzyl isobutyrate and 4-(trifluoromethyl)benzaldehyde on 10 mmol scale to give **S19** (2.4 g, 36% total yield) as a white solid.

^{1}H NMR (400 MHz, CD_2Cl_2) δ 7.59 – 7.54 (m, 2H), 7.49 (t, *J* = 7.0 Hz, 3H), 7.47 – 7.37 (m, 5H), 7.34 – 7.08 (m, 12H), 6.96 (d, *J* = 8.6 Hz, 2H), 5.71 (d, *J* = 8.3 Hz, 1H), 1.15 (s, 3H), 0.94 (s, 3H).

^{13}C NMR (100 MHz, CD_2Cl_2) δ 171.42, 166.33, 150.89 (d, *J* = 7.1 Hz), 150.74 (d, *J* = 7.1 Hz), 140.27, 134.74, 132.83, 131.52, 130.81 (q, *J* = 284.3 Hz), 129.95, 129.24,

128.85 (d, J = 3.4 Hz), 128.70, 128.59, 125.73 (d, J = 6.7 Hz), 125.21 (d, J = 3.8 Hz), 123.09, 120.44 (d, J = 4.9 Hz), 120.23 (d, J = 5.0 Hz), 84.86 (d, J = 6.2 Hz), 48.54 (d, J = 8.2 Hz), 20.77, 20.62.

^{31}P NMR (160 MHz, CD_2Cl_2) δ) δ -12.67 (d, J = 8.2 Hz).

^{19}F NMR (376 MHz, CD_2Cl_2) δ -62.99.

HRMS (ESI-TOF) m/z calcd. for $C_{37}H_{32}F_3NO_6P^+$ ($[M+H]^+$) 674.1914, found: 674.1914.

1-([1,1'-Biphenyl]-4-yl)-3-(((diphenylmethylene)amino)oxy)-2,2-dimethyl-3-oxopropyl diphenyl phosphate (S20)

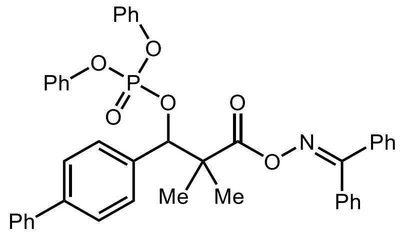

Prepared following the general procedure B outlined above starting from 4-methoxybenzyl isobutyrate and [1,1'-biphenyl]-4-carbaldehyde on 10 mmol scale to give **S20** (3.0 g, 44% total yield) as a white solid.

^{1}H NMR (400 MHz, CD_2Cl_2) δ 7.63 – 7.56 (m, 4H), 7.53 – 7.37 (m, 11H), 7.31 – 7.12 (m, 12H), 7.02 – 6.96 (m, 2H), 5.73 (d, J = 7.9 Hz, 1H), 1.20 (s, 3H), 0.99 (s, 3H).

^{13}C NMR (100 MHz, CD_2Cl_2) 171.77, 166.01, 150.93 (d, J = 6.7 Hz), 150.86 (d, J = 7.1 Hz), 141.74, 140.77, 135.08, 134.83, 132.83, 131.41, 130.02 (d, J = 7.3 Hz), 129.89, 129.23, 129.21, 128.92, 128.81, 128.76, 128.55, 127.92, 127.39, 126.88, 125.57 (d, J = 9.0 Hz), 120.51 (d, J = 4.6 Hz), 120.34 (d, J = 5.0 Hz), 85.61 (d, J = 6.2 Hz), 48.66 (d, J = 8.6 Hz), 21.07, 20.38.

^{31}P NMR (160 MHz, CD_2Cl_2) δ. -12.59 (d, *J* = 7.7 Hz).

HRMS (ESI-TOF) m/z calcd. for $C_{42}H_{37}NO_6P^+$ ($[M+H]^+$) 682.2353, found: 682.2346.

3-(((Diphenylmethylene)amino)oxy)-2,2-dimethyl-3-oxo-1-(4-(4,4,5,5-tetramethyl-1,3,2-dioxaborolan-2-yl)phenyl)propyl diphenyl phosphate (S21)

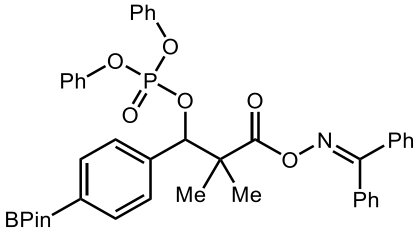

Prepared following the general procedure B outlined above starting from 4-methoxybenzyl isobutyrate and 4-(4,4,5,5-tetramethyl-1,3,2-dioxaborolan-2-yl)benzaldehyde on 20 mmol scale to give **S21** (2.9 g, 20 % total yield) as a white solid.

^{1}H NMR (400 MHz, CD_2Cl_2) δ 7.66 (d, *J* = 7.9 Hz, 2H), 7.55 (d, *J* = 7.2 Hz, 2H), 7.46 – 7.38 (m, 5H), 7.30 – 7.08 (m, 13H), 6.95 (d, *J* = 8.1 Hz, 2H), 5.65 (d, *J* = 7.9 Hz, 1H), 1.32 (s, 12H), 1.12 (s, 3H), 0.92 (s, 3H).

^{13}C NMR (100 MHz, $CDCl_3$) δ 171.71, 165.98, 150.89 (d, *J* = 6.8 Hz), 150.84 (d, *J* = 6.8 Hz), 138.87, 134.83, 134.51, 132.82, 131.38, 130.01 (d, *J* = 6.0 Hz), 129.86, 129.22, 129.22, 128.91, 128.79, 128.54, 127.53, 125.58 (d, *J* = 6.5 Hz), 120.51 (d, *J* = 4.9 Hz), 120.34 (d, *J* = 5.0 Hz), 85.60 (d, *J* = 6.2 Hz), 84.27, 48.56 (d, *J* = 8.5 Hz), 25.02, 21.10, 20.21.

^{31}P NMR (160 MHz, CD_2Cl_2) δ. -12.74 (d, *J* = 7.8 Hz).

HRMS (ESI-TOF) m/z calcd. for $C_{42}H_{44}BNO_8P^+$ ($[M+H]^+$) 733.2926, found: 733.2922.

Methyl 4-(1-((diphenoxyphosphoryl)oxy)-3-(((diphenylmethylene)amino)oxy)-

2,2-dimethyl-3-oxopropyl)benzoate (S22)

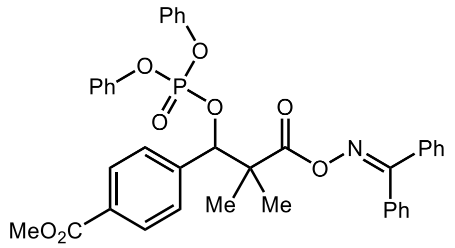

Prepared following the general procedure B outlined above starting from 4-methoxybenzyl isobutyrate and methyl 4-formylbenzoate on 20 mmol scale to give **S22** (3.2 g, 24% total yield) as a white solid.

^{1}H NMR (400 MHz, CD_2Cl_2) δ 7.89 (d, *J* = 8.4 Hz, 2H), 7.58 – 7.52 (m, 2H), 7.51 – 7.36 (m, 6H), 7.32 – 7.10 (m, 12H), 6.95 (d, *J* = 8.5 Hz, 2H), 5.70 (d, *J* = 8.2 Hz, 1H), 3.89 (s, 3H), 1.13 (s, 3H), 0.93 (s, 3H).

^{13}C NMR (100 MHz, CD_2Cl_2) δ 171.48, 166.76, 166.14, 150.83 (d, *J* = 7.1 Hz), 150.72 (d, *J* = 7.1 Hz), 140.89, 134.73, 132.75, 131.45, 130.83, 130.06 (d, *J* = 6.7 Hz), 129.91, 129.33, 129.21, 128.86, 128.81, 128.53, 128.23, 125.67 (d, *J* = 7.2 Hz), 120.45 (d, *J* = 4.9 Hz), 120.25 (d, *J* = 5.0 Hz), 85.04 (d, *J* = 6.2 Hz), 52.45, 48.54 (d, *J* = 8.3 Hz), 20.92, 20.39.

^{31}P NMR (160 MHz, CD_2Cl_2) δ -12.67 (d, *J* = 8.1 Hz).

HRMS (ESI-TOF) m/z calcd. for $C_{38}H_{35}NO_8P^+$ ($[M+H]^+$) 664.2095, found: 664.2095.

3-(((Diphenylmethylene)amino)oxy)-1-(4-isocyanophenyl)-2,2-dimethyl-3-oxopropyl diphenyl phosphate (S23)

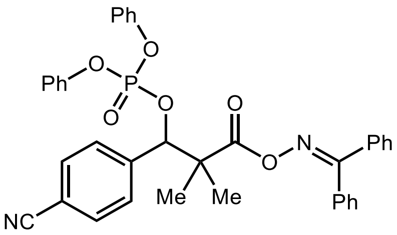

Prepared following the general procedure B outlined above starting from 4-methoxybenzyl isobutyrate and 4-cyanobenzaldehyde on 20 mmol scale to give **S23** (5.7 g, 45% total yield) as a white solid.

^{1}H NMR (400 MHz, CDCl$_3$) δ 7.59 – 7.52 (m, 2H), 7.50 – 7.34 (m, 8H), 7.26 – 7.20 (m, 6H), 7.18 – 7.08 (m, 6H), 6.98 (d, *J* = 8.5 Hz, 2H), 5.70 (d, *J* = 8.4 Hz, 1H), 1.18 (s, 3H), 0.93 (s, 3H).

^{13}C NMR (100 MHz, CDCl$_3$) 171.05, 166.32, 150.45 (d, *J* = 7.3 Hz), 150.33 (d, *J* = 7.2 Hz), 141.17, 134.16, 132.47, 131.72, 131.34, 129.86 (d, *J* = 5.8 Hz), 129.75, 129.15, 128.60, 128.55, 128.52, 128.31, 125.52 (d, *J* = 7.0 Hz), 120.12 (d, *J* = 4.9 Hz), 119.92 (d, *J* = 5.1 Hz), 118.49, 112.50, 84.29 (d, *J* = 6.2 Hz), 48.38 (d, *J* = 8.1 Hz), 21.40, 20.03.

^{31}P NMR (160 MHz, CDCl$_3$) δ -12.92 (d, *J* = 8.3 Hz).

HRMS (ESI-TOF) m/z calcd. for $C_{37}H_{32}N_2O_6P^+$ ([M+H]$^+$), 631.1993, found: 631.1992.

3-(((Diphenylmethylene)amino)oxy)-2,2-dimethyl-1-(4-((methylperoxy)thio)phenyl)-3-oxopropyl diphenyl phosphate (S24)

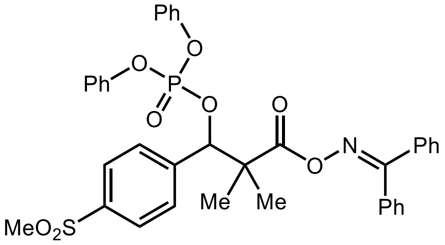

Prepared following the general procedure B outlined above starting from 4-

methoxybenzyl isobutyrate and 4-(methylsulfonyl)benzaldehyde on 10 mmol scale to give **S24** (2.6 g, 38% total yield) as a white solid.

^{1}H NMR (400 MHz, CD_2Cl_2) δ 7.77 (d, *J* = 8.4 Hz, 2H), 7.58 – 7.53 (m, 2H), 7.50 – 7.37 (m, 6H), 7.36 – 7.10 (m, 12H), 7.02 – 6.94 (m, 2H), 5.74 (d, *J* = 8.4 Hz, 1H), 3.01 (s, 3H), 1.14 (s, 3H), 0.94 (s, 3H).

^{13}C NMR (100 MHz, CD_2Cl_2) 171.31, 166.38, 150.80 (d, *J* = 7.2 Hz), 150.66 (d, *J* = 7.0 Hz), 142.09, 141.13, 134.62, 132.74, 131.53, 130.10 (d, *J* = 7.1 Hz), 129.98, 129.20, 129.11, 128.84, 128.79, 128.62, 127.29, 125.77 (d, *J* = 3.8 Hz), 120.38 (d, *J* = 4.9 Hz), 120.22 (d, *J* = 5.0 Hz), 84.54 (d, *J* = 6.1 Hz), 48.50 (d, *J* = 8.1 Hz), 44.71, 20.75, 20.53.

^{31}P NMR (160 MHz, CD_2Cl_2) δ -12.71 (d, *J* = 8.2 Hz).

HRMS (ESI-TOF) m/z calcd. for $C_{37}H_{34}NO_8PSNa^+$ ($[M+Na]^+$) 706.1641, found: 706.1635.

3-(((Diphenylmethylene)amino)oxy)-2,2-dimethyl-1-(4-nitrophenyl)-3-oxopropyl diphenyl phosphate (S25)

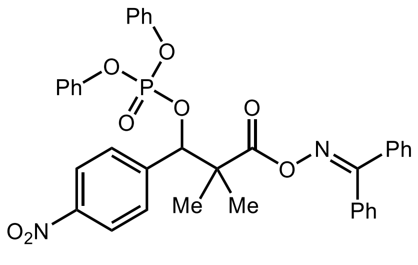

Prepared following the general procedure B outlined above starting from 4-methoxybenzyl isobutyrate and 4-nitrobenzaldehyde on 20 mmol scale to give **S25** (1.6 g, 12% total yield) as a white solid.

^{1}H NMR (400 MHz, CD_2Cl_2) δ 8.03 (d, *J* = 8.8 Hz, 2H), 7.57 – 7.53 (m, 2H), 7.50 – 7.39 (m, 6H), 7.29 (d, *J* = 8.3 Hz, 3H), 7.25 – 7.13 (m, 9H), 6.98 (d, *J* = 8.6 Hz, 2H), 5.73 (d, *J* = 8.4 Hz, 1H), 1.15 (s, 3H), 0.95 (s, 3H).

^{13}C NMR (100 MHz, CD_2Cl_2) δ 171.19, 166.39, 150.75 (d, *J* = 7.1 Hz), 150.63 (d, *J* = 8.0 Hz), 148.24, 143.21, 134.62, 132.68, 131.54, 130.59, 130.13 (d, *J* = 6.9 Hz), 130.00, 129.23, 129.08, 128.85, 128.55, 125.81 (d, *J* = 8.4 Hz), 123.32, 120.40 (d, *J* = 5.0 Hz), 120.20 (d, *J* = 5.0 Hz), 84.35 (d, *J* = 6.2 Hz), 48.53 (d, *J* = 8.0 Hz), 20.77, 20.65.

^{31}P NMR (160 MHz, CD_2Cl_2) δ -12.65 (d, *J* = 8.3 Hz).

HRMS (ESI-TOF) m/z calcd. for $C_{36}H_{32}N_2O_8P^+$ ($[M+H]^+$) 651.1891, found: 651.1891.

1-(3,5-Dimethylphenyl)-3-(((diphenylmethylene)amino)oxy)-2,2-dimethyl-3-oxopropyl diphenyl phosphate (S26)

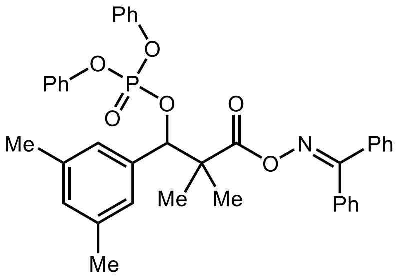

Prepared following the general procedure B outlined above starting from 4-methoxybenzyl isobutyrate and 3,5-dimethylbenzaldehyde on 20 mmol scale to give **S26** (5.5 g, 43% total yield) as a white solid.

^{1}H NMR (400 MHz, CD_2Cl_2) δ 7.56 (d, *J* = 7.9 Hz, 2H), 7.51 – 7.38 (m, 6H), 7.32 – 7.10 (m, 10H), 7.03 – 6.92 (m, 3H), 6.76 (s, 2H), 5.57 (d, *J* = 8.0 Hz, 1H), 2.23 (s, 6H), 1.14 (s, 3H), 0.94 (s, 3H).

^{13}C NMR (100 MHz, CD_2Cl_2) δ 171.87, 165.81, δ 150.96 (d, *J* = 6.6 Hz), 150.88 (d, *J* = 6.6 Hz), 137.77, 135.82, 134.93, 132.84, 131.34, 130.53, 129.97 (d, *J* = 6.6 Hz), 129.86, 129.21, 129.00, 128.79, 128.51, 126.00, 125.51 (d, *J* = 8.5 Hz), 120.50 (d, *J* = 4.9 Hz), 120.29 (d, *J* = 5.0 Hz), 85.87 (d, *J* = 6.2 Hz), 48.53 (d, *J* = 8.6 Hz), 21.42, 21.24, 20.21.

^{31}P NMR (160 MHz, CD_2Cl_2) δ -12.75 (d, *J* = 7.9 Hz).

HRMS (ESI-TOF) m/z calcd. for $C_{38}H_{37}NO_6P^+$ ($[M+H]^+$) 634.2353, found: 634.2352.

1-(3,5-dichlorophenyl)-3-(((diphenylmethylene)amino)oxy)-2,2-dimethyl-3-oxopropyl diphenyl phosphate (S27)

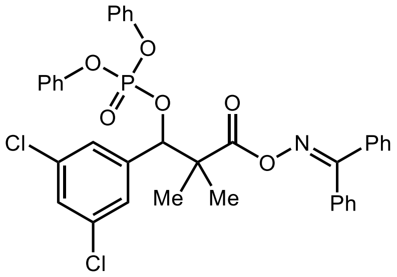

Prepared following the general procedure B outlined above starting from 4-methoxybenzyl isobutyrate and 3,5-dichlorobenzaldehyde on 20 mmol scale to give **S27** (6.2 g, 46% total yield) as a white solid.

^{1}H NMR (400 MHz, CD_2Cl_2) δ 7.56 (dd, *J* = 4.8, 3.3 Hz, 2H), 7.50 – 7.37 (m, 6H), 7.35 – 7.14 (m, 11H), 7.06 – 7.00 (m, 4H), 5.55 (d, *J* = 8.5 Hz, 1H), 1.14 (s, 3H), 0.95 (s, 3H).

^{13}C NMR (100 MHz, CD_2Cl_2) 171.18, 166.34, 150.77 (d, *J* = 6.5 Hz), 150.57 (d, *J* = 7.7 Hz), 139.60, 134.87, 134.67, 132.70, 131.49, 130.11 (d, *J* = 6.2 Hz), 129.98, 129.24, 129.10, 128.85, 128.82, 128.59, 126.69, 125.81 (d, *J* = 4.5 Hz), 120.39 (d, *J* = 4.8 Hz), 120.10 (d, *J* = 4.9 Hz), 84.02 (d, *J* = 5.9 Hz), 48.50 (d, *J* = 8.0 Hz), 20.72, 20.58.

^{31}P NMR (160 MHz, CD_2Cl_2) δ -12.66 (d, *J* = 8.1 Hz).

HRMS (ESI-TOF) m/z calcd. for $C_{36}H_{31}Cl_2NO_6P^+$ ($[M+H]^+$) 672.1261, found: 672.1260.

3-(((Diphenylmethylene)amino)oxy)-2,2-dimethyl-1-(naphthalen-2-yl)-3-oxopropyl diphenyl phosphate (S28)

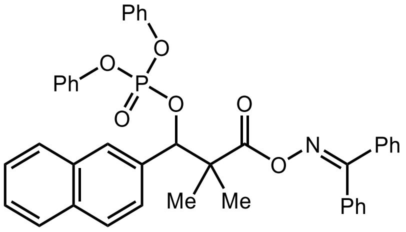

Prepared following the general procedure B outlined above starting from 4-methoxybenzyl isobutyrate and 2-naphthaldehyde on 20 mmol scale to give **S28** (5.3 g, 40% total yield) as a white solid.

^{1}H NMR (400 MHz, CDCl$_3$) δ 7.84 – 7.76 (m, 1H), 7.71 (dd, *J* = 14.8, 7.8 Hz, 2H), 7.61 – 7.42 (m, 6H), 7.38 – 7.29 (m, 4H), 7.21 (dd, *J* = 16.0, 7.7 Hz, 5H), 7.07 (ddd, *J* = 33.0, 16.6, 7.5 Hz, 8H), 6.93 (d, *J* = 8.4 Hz, 2H), 5.86 (d, *J* = 8.1 Hz, 1H), 1.24 (s, 3H), 0.98 (s, 3H).

^{13}C NMR (100 MHz, CDCl$_3$) δ 171.66, 165.85, 150.63 (d, *J* = 7.3 Hz), 150.56 (d, *J* = 7.4 Hz), 134.46, 133.39, 132.70, 132.54, 131.13, 129.73, 129.64, 129.51, 129.17, 128.54, 128.46, 128.16, 127.66 (d, *J* = 3.6 Hz), 127.58, 126.55, 126.28, 125.45, 125.21 (d, *J* = 14.1 Hz), 120.27 (d, *J* = 4.9 Hz), 120.03 (d, *J* = 5.1 Hz)., 85.67 (d, *J* = 6.4 Hz), 48.75 (d, *J* = 8.5 Hz), 21.15, 20.60.

^{31}P NMR (160 MHz, CDCl$_3$) δ -12.94 (d, *J* = 8.0 Hz).

HRMS (ESI-TOF) m/z calcd. for $C_{40}H_{35}NO_6P^+$ ([M+H]$^+$) 656.2197, found: 656.2192.

3-(((Diphenylmethylene)amino)oxy)-2,2-dimethyl-3-oxo-1-(pyridin-4-yl)propyl diphenyl phosphate (S29)

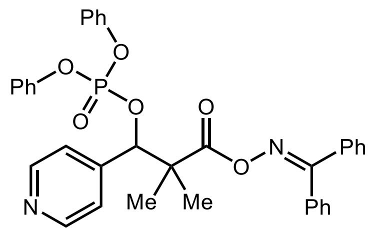

Prepared following the general procedure B outlined above starting from 4-methoxybenzyl isobutyrate and pyridin-4-carbaldehyde on 10 mmol scale to give **S29** (1.8 g, 30% total yield) as a white solid.

^{1}H NMR (400 MHz, CD_2Cl_2) δ 8.47 (d, *J* = 5.6 Hz, 2H), 7.60 – 7.53 (m, 2H), 7.51 – 7.37 (m, 6H), 7.33 – 7.23 (m, 4H), 7.20 – 7.12 (m, 6H), 7.05 (d, *J* = 5.8 Hz, 2H), 6.99 (d, *J* = 8.4 Hz, 2H), 5.63 (d, *J* = 8.3 Hz, 1H), 1.13 (s, 3H), 0.92 (s, 3H).

^{13}C NMR (100 MHz, CD_2Cl_2) δ 171.29, 166.42, 150.82 (d, *J* = 7.2 Hz), 150.70 (d, *J* = 7.2 Hz), 149.88, 144.66, 134.66, 132.78, 131.53, 130.13 (d, *J* = 4.9 Hz), 129.94, 129.22, 128.85, 128.78, 128.61, 125.80 (d, *J* = 2.6 Hz), 122.82, 120.43 (d, *J* = 4.9 Hz), 120.26 (d, *J* = 5.0 Hz), 84.00 (d, *J* = 6.1 Hz), 48.29 (d, *J* = 8.0 Hz), 20.65, 20.51.

^{31}P NMR (160 MHz, CD_2Cl_2) δ -12.69 (d, *J* = 8.2 Hz).

HRMS (ESI-TOF) m/z calcd. for $C_{35}H_{32}N_2O_6P^+$ ($[M+H]^+$) 607.1993, found: 607.1991.

3-(((Bis(4-fluorophenyl)methylene)amino)oxy)-2,2-dimethyl-3-oxo-1-phenylpropyl diphenyl phosphate (S30)

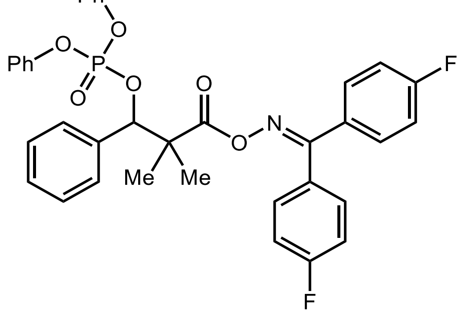

Prepared following the general procedure B outlined above starting from 4-methoxybenzyl isobutyrate and benzaldehyde on 20 mmol scale to give **S30** (2.3 g, 18% total yield) as a white solid.

^{1}H NMR (400 MHz, CD_2Cl_2) δ 7.60 – 7.51 (m, 2H), 7.34 – 7.11 (m, 19H), 6.94 (d, *J* = 8.4 Hz, 2H), 5.68 (d, *J* = 8.0 Hz, 1H), 1.17 (s, 3H), 0.97 (s, 3H).

^{13}C NMR (100 MHz, CD_2Cl_2) δ 171.70, 163.97 (d, *J* = 251.5 Hz), 163.88, 163.65 (d, *J* = 250.5 Hz), 150.89 (d, *J* = 6.7 Hz), 150.83 (d, *J* = 6.7 Hz), 135.90, 132.16, 131.43 (d, *J* = 3.7 Hz), 131.35 (d, *J* = 3.4 Hz), 131.03 (d, *J* = 3.1 Hz), 130.04 (d, *J* = 8.0 Hz), 129.09, 128.53 (d, *J* = 3.5 Hz), 128.31 (d, *J* = 3.8 Hz), 125.62 (d, *J* = 9.0 Hz), 120.52 (d, *J* = 4.9 Hz), 120.30 (d, *J* = 4.9 Hz), 115.98 (d, *J* = 19.8 Hz), 115.76 (d, *J* = 19.7 Hz), 85.82 (d, *J* = 6.2 Hz), 48.59 (d, *J* = 8.6 Hz), 21.38, 20.08.

^{31}P NMR (160 MHz, CD_2Cl_2) δ -12.50 (d, *J* = 7.6 Hz).

^{19}F NMR (376 MHz, CD_2Cl_2) δ -109.35 – -109.42 (m), -109.76 – -109.84 (m).

HRMS (ESI-TOF) m/z calcd. for $C_{36}H_{31}F_2NO_6P^+$ ($[M+H]^+$) 642.1852, found: 642.1852.

3-(((Diphenylmethylene)amino)oxy)-2,2-dimethyl-3-oxo-1-phenylpropyl dimethyl phosphate (S31)

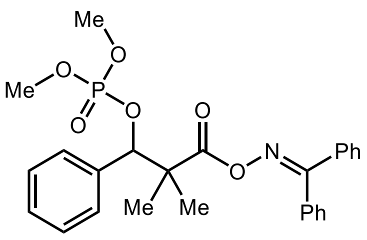

Prepared following the general procedure B outlined above starting from 4-methoxybenzyl isobutyrate and benzaldehyde on 20 mmol scale to give **S31** (2.1 g, 22%

total yield) as a white solid.

^{1}H NMR (400 MHz, CD_2Cl_2) δ 7.63 – 7.58 (m, 2H), 7.51 – 7.46 (m, 4H), 7.43 – 7.37 (m, 2H), 7.34 (dd, *J* = 5.1, 1.7 Hz, 3H), 7.29 – 7.21 (m, 4H), 5.41 (d, *J* = 8.0 Hz, 1H), 3.61 (d, *J* = 11.2 Hz, 3H), 3.49 (d, *J* = 11.3 Hz, 3H), 1.12 (s, 3H), 0.90 (s, 3H).

^{13}C NMR (100 MHz, CD_2Cl_2) δ 172.10, 166.09, 136.80, 134.91, 132.91, 131.42, 129.91, 129.21, 129.01, 128.89, 128.86, 128.56, 128.25, 128.20, 84.09 (d, *J* = 5.8 Hz), 54.64 (d, *J* = 5.9 Hz), 54.47 (d, *J* = 6.0 Hz), 48.41 (d, *J* = 8.2 Hz), 21.47, 19.81.

^{31}P NMR (160 MHz, CD_2Cl_2) δ 0.57 – 0.11 (m).

HRMS (ESI-TOF) m/z calcd. for $C_{26}H_{29}NO_6P^+$ ($[M+H]^+$) 482.1727, found: 482.1726.

1-(2-(((Diphenylmethylene)amino)oxy)-2-oxoethyl)cyclohexyl diphenyl phosphate (S32)

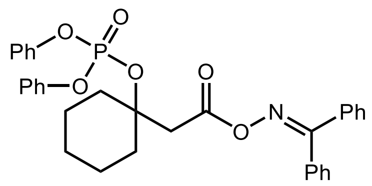

Prepared following the general procedure A outlined above starting from cyclohexanone and benzyl 2-bromoacetate on 20 mmol scale to give **S32** (3.8 g, 33% total yield) as a white solid.

^{1}H NMR (400 MHz, $CDCl_3$) δ 7.56 (d, *J* = 7.2 Hz, 2H), 7.50 – 7.30 (m, 10H), 7.29 – 7.11 (m, 8H), 2.94 (s, 2H), 2.11 – 2.03 (m, 2H), 1.75 – 1.55 (m, 4H), 1.49 – 1.25 (m, 4H).

^{13}C NMR (100 MHz, CD_2Cl_2) δ 166.94, 165.39, 151.17 (d, *J* = 7.4 Hz), 135.09, 132.99, 131.35, 130.07, 129.92, 129.21, 129.00, 128.83, 128.65, 125.54, 120.59 (d, *J* = 4.9 Hz), 87.52 (d, *J* = 8.1 Hz), 43.35, 36.12 (d, *J* = 4.8 Hz), 24.98, 22.40.

^{31}P NMR (160 MHz, CD_2Cl_2) δ -16.98.

HRMS (ESI-TOF) m/z calcd. for $C_{33}H_{33}NO_6P^+$ ($[M+H]^+$) 570.2040, found: 570.2040.

1-(2-(((Diphenylmethylene)amino)oxy)-2-oxoethyl)-4,4-dimethylcyclohexyl diphenyl phosphate (S33)

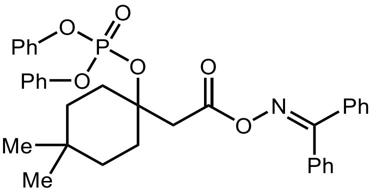

Prepared following the general procedure A outlined above starting from cyclohexanone and benzyl 2-bromoacetate on 10 mmol scale to give **S33** (1.8 g, 30% total yield) as a white solid.

^{1}H NMR (400 MHz, $CDCl_3$) δ 7.58 – 7.54 (m, 2H), 7.50 – 7.43 (m, 4H), 7.40 –7.30 (m, 6H), 7.29 – 7.16 (m, 8H), 2.95 (s, 2H), 2.04 (d, *J* = 14.3 Hz, 2H), 1.80 (t, *J* = 12.4 Hz, 2H), 1.42 – 1.32 (m, 2H), 1.16 – 1.08 (m, 2H), 0.83 (d, *J* = 6.3 Hz, 6H).

^{13}C NMR (100 MHz, CD_2Cl_2) δ 166.95, 165.22, 151.05 (d, *J* = 7.6 Hz), 134.98, 132.79, 131.31, 130.05, 129.91, 129.16, 129.00, 128.77, 128.58, 125.52, 120.52 (d, *J* = 4.9 Hz), 87.19 (d, *J* = 8.0 Hz), 42.98, 34.72, 32.17, 32.00 (d, *J* = 4.9 Hz), 29.27.

^{31}P NMR (160 MHz, CD_2Cl_2) δ -16.85.

HRMS (ESI-TOF) m/z calcd. for $C_{35}H_{38}NO_6P^+$ ($[M+H]^+$) 598.2353, found: 570.2350.

1-(2-(((Diphenylmethylene)amino)oxy)-2-oxoethyl)-4,4-difluorocyclohexyl diphenyl phosphate (S34)

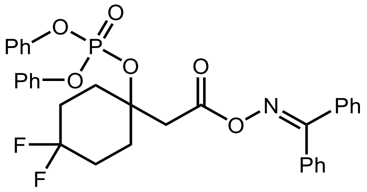

Prepared following the general procedure A outlined above starting from 4,4-difluorocyclohexan-1-one and benzyl 2-bromoacetate on 10 mmol scale to give **S34** (0.9 g, 15% total yield) as a colorless oil.

^{1}H NMR (400 MHz, CD_2Cl_2) δ 7.60 – 7.55 (m, 2H), 7.52 – 7.44 (m, 4H), 7.42 – 7.33 (m, 6H), 7.33 – 7.15 (m, 8H), 3.06 (s, 2H), 2.32 (d, *J* = 11.2 Hz, 2H), 2.02 – 1.84 (m, 6H).

^{13}C NMR (100 MHz, CD_2Cl_2) δ 166.43, 165.68, 150.95 (d, *J* = 7.5 Hz), 134.91, 132.88, 131.46, 130.23, 129.99, 129.23, 128.96, 128.86, 128.69, 125.87, 122.77(dd, *J* = 240.4 Hz, 240.4 Hz), 120.48 (d, *J* = 4.9 Hz), 84.15 (d, *J* = 7.9 Hz), 43.03, 32.60 (dd, *J* = 9.2, 5.5 Hz), 29.53 (t, *J* = 24.9 Hz).

^{31}P NMR (160 MHz, CD_2Cl_2) δ -16.61.

^{19}F NMR (100 MHz, CD_2Cl_2) δ -93.96 (d, *J* = 236.6 Hz), -98.44 – -98.58 (m), -103.31 (d, *J* = 236.8 Hz).

HRMS (ESI-TOF) m/z calcd. for $C_{33}H_{31}F_2NO_6P^+$ ($[M+H]^+$) 606.1852, found: 606.1845.

9-(2-(((Diphenylmethylene)amino)oxy)-2-oxoethyl)-3,3-dimethyl-2,4-dioxaspiro[5.5]undecan-9-yl diphenyl phosphate (S35)

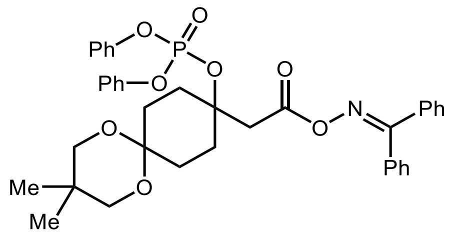

Prepared following the general procedure A outlined above starting from 3,3-dimethyl-1,5-dioxaspiro[5.5]undecan-9-one and benzyl 2-bromoacetate on 20 mmol scale to give **S35** (3.2 g, 24% total yield) as a colorless oil.

^{1}H NMR (400 MHz, CDCl$_3$) δ 7.53 (d, *J* = 7.4 Hz, 2H), 7.44 – 7.37 (m, 6H), 7.31 (dd, *J* = 15.1, 7.6 Hz, 4H), 7.24 – 7.19 (m, 3H), 7.12 (dd, *J* = 14.3, 6.9 Hz, 5H), 3.45 (s, 2H), 3.38 (s, 2H), 2.98 (s, 2H), 2.15 (d, *J* = 13.7 Hz, 2H), 1.94 (d, *J* = 12.9 Hz, 2H), 1.86 – 1.79 (m, 2H), 1.73 – 1.63 (m, 2H), 0.91 (s, 6H).

^{13}C NMR (100 MHz, CDCl$_3$) δ 166.44, 165.16, 150.66 (d, *J* = 7.4 Hz), 134.61, 132.51, 130.99, 129.73, 129.61, 129.07, 128.74, 128.40, 128.25, 125.20, 120.18 (d, *J* = 5.0 Hz), 96.38, 85.83 (d, *J* = 8.1 Hz), 70.07, 42.99, 32.18 (d, *J* = 5.4 Hz), 30.18, 27.65, 22.68.

^{31}P NMR (160 MHz, CDCl$_3$) δ -16.96.

HRMS (ESI-TOF) m/z calcd. for $C_{38}H_{41}NO_8P^+$ ($[M+H]^+$) 670.2565, found: 670.2562.

1-(((Diphenylmethylene)amino)oxy)-3-methyl-1-oxodecan-3-yl diphenyl phosphate (S36)

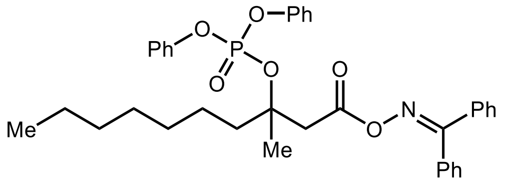

Prepared following the general procedure A outlined above starting from undecan-2-one and benzyl 2-bromoacetate on 10 mmol scale to give **S36** (1.4 g, 23% total yield) as a colorless oil.

^{1}H NMR (400 MHz, CD_2Cl_2) δ 7.58 – 7.53 (m, 2H), 7.50 – 7.25 (m, 12H), 7.22 – 7.13 (m, 6H), 2.94 – 2.74 (m, 2H), 1.87 – 1.75 (m, 2H), 1.59 (s, 3H), 1.29 – 1.15 (m, 10H), 0.87 (t, *J* = 7.0 Hz, 3H).

^{13}C NMR (100 MHz, CD_2Cl_2) 166.96, 165.25, 151.04 (d, *J* = 6.7 Hz), 135.03, 132.79, 131.34, 130.04, 129.97, 129.17, 128.98, 128.79, 128.62, 125.53, 120.53 (d, *J* = 4.5 Hz), 87.54 (d, *J* = 7.6 Hz), 43.40 (d, *J* = 5.1 Hz), 40.50 (d, *J* = 4.9 Hz), 32.11, 29.91, 29.50, 25.65, 25.63, 23.97, 23.01, 14.26.

^{31}P NMR (160 MHz, CD_2Cl_2) -16.80.

HRMS (ESI-TOF) m/z calcd. for $C_{36}H_{41}NO_6P^+$ ($[M+H]^+$) 614.2666, found: 614.2668

1-(((Diphenylmethylene)amino)oxy)-3-methyl-1-oxododecan-3-yl diphenyl phosphate (S37)

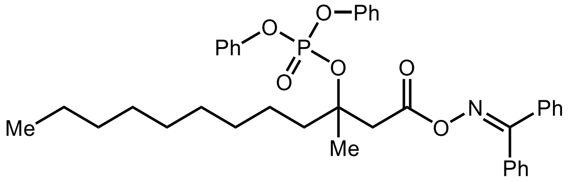

Prepared following the general procedure A outlined above starting from undecan-2-one and benzyl 2-bromoacetate on 10 mmol scale to give **S37** (1.1 g, 17% total yield) as a colorless oil.

^{1}H NMR (400 MHz, CD_2Cl_2) δ 7.59 – 7.54 (m, 2H), 7.52 – 7.44 (m, 4H), 7.42 – 7.27 (m, 8H), 7.20 (dd, *J* = 7.4, 3.7 Hz, 6H), 2.93 – 2.77 (m, 2H), 1.86 – 1.78 (m, 2H), 1.60 (s, 3H), 1.32 – 1.17 (m, 14H), 0.89 (t, *J* = 6.9 Hz, 3H).

^{13}C NMR (100 MHz, CD_2Cl_2) δ 166.99, 165.35, 151.14 (d, *J* = 6.8 Hz), 135.10, 132.90, 131.37, 130.07, 130.00, 129.22, 129.02, 128.83, 128.67, 125.56, 120.58 (d, *J* = 4.6 Hz),

87.66 (d, J = 7.6 Hz), 43.49 (d, J = 5.1 Hz), 40.59 (d, J = 4.8 Hz), 32.29, 30.01, 29.91, 29.87, 29.71, 25.72, 24.02, 23.09, 14.29.

^{31}P NMR (160 MHz, CD_2Cl_2) -16.95.

HRMS (ESI-TOF) m/z calcd. for $C_{38}H_{45}NO_6P^+$ ($[M+H]^+$) 642.2979, found: 642.2974.

1-(((Diphenylmethylene)amino)oxy)-3,7-dimethyl-1-oxooctan-3-yl diphenyl phosphate (S38)

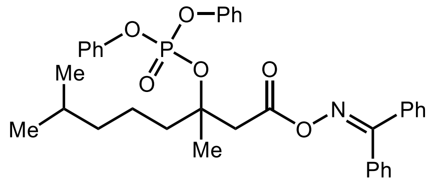

Prepared following the general procedure A outlined above starting from undecan-2-one and benzyl 2-bromoacetate on 10 mmol scale to give **S38** (1.5 g, 25% total yield) as a colorless oil.

^{1}H NMR (400 MHz, CD_2Cl_2) δ 7.59 – 7.53 (m, 2H), 7.51 – 7.25 (m, 12H), 7.18 (dd, J = 7.3, 4.0 Hz, 6H), 2.93 – 2.77 (m, 2H), 1.84 – 1.75 (m, 2H), 1.60 (s, 3H), 1.49 – 1.39 (m, 1H), 1.29 – 1.20 (m, 2H), 1.13 – 1.02 (m, 2H), 0.82 (d, J = 6.6 Hz, 6H).

^{13}C NMR (100 MHz, CD_2Cl_2) δ 166.96, 165.21, 151.00 (d, J = 7.0 Hz), 135.00, 132.75, 131.33, 130.03, 129.96, 129.16, 128.98, 128.78, 128.60, 125.52, 120.50 (d, J = 4.8 Hz), 87.50 (d, J = 7.6 Hz), 43.40 (d, J = 5.0 Hz), 40.64 (d, J = 5.1 Hz), 39.06, 28.11, 25.61, 25.59, 22.64, 22.60, 21.75.

^{31}P NMR (160 MHz, CD_2Cl_2) -16.77.

HRMS (ESI-TOF) m/z calcd. for $C_{35}H_{39}NO_6P^+$ ($[M+H]^+$) 600.2510, found: 600.2509.

7-Bromo-1-(((diphenylmethylene)amino)oxy)-3-methyl-1-oxoheptan-3-yl diphenyl phosphate (S39)

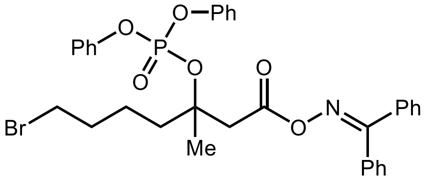

Prepared following the general procedure A outlined above starting from 7-bromoheptan-2-one and benzyl 2-bromoacetate on 10 mmol scale to give **S39** (1.2 g, 18% total yield) as a colorless oil.

^{1}H NMR (400 MHz, CD_2Cl_2) δ 7.61 – 7.55 (m, 2H), 7.53 – 7.46 (m, 4H), 7.44 – 7.28 (m, 8H), 7.21 (dd, *J* = 7.6, 3.6 Hz, 6H), 3.48 – 3.29 (m, 2H), 2.97 – 2.81 (m, 2H), 1.86 (dd, *J* = 9.5, 6.5 Hz, 2H), 1.78 – 1.65 (m, 2H), 1.63 (s, 3H), 1.46 – 1.35 (m, 2H).

^{13}C NMR (100 MHz, CD_2Cl_2) δ 166.87, 165.43, 151.06 (d, *J* = 7.5 Hz), 135.02, 132.86, 131.40, 130.11, 130.03, 129.20, 128.99, 128.83, 128.68, 125.63, 120.56 (d, *J* = 4.9 Hz), 87.05 (d, *J* = 7.5 Hz), 45.20, 43.41 (d, *J* = 5.0 Hz), 39.58 (d, *J* = 5.1 Hz), 32.80, 25.67, 25.65, 21.40.

^{31}P NMR (160 MHz, CD_2Cl_2) δ -16.79.

HRMS (ESI-TOF) m/z calcd. for $C_{33}H_{34}BrNO_6P^+$ ($[M+H]^+$) 650.1302, found: 650.1298.

6-(2-(((Diphenylmethylene)amino)oxy)-2-oxoethyl)undecan-6-yl diphenyl phosphate (S40)

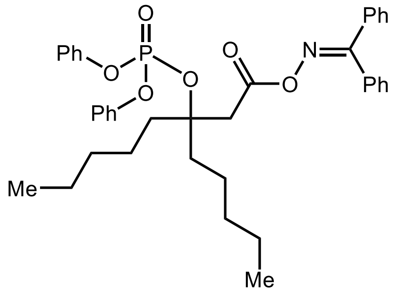

Prepared following the general procedure A outlined above starting from undecan-2-one and benzyl 2-bromoacetate on 10 mmol scale to give **S40** (1.4 g, 22% total yield) as a colorless oil.

^{1}H NMR (400 MHz, CD_2Cl_2) δ 7.60 – 7.54 (m, 2H), 7.48 – 7.42 (m, 4H), 7.39 – 7.26 (m, 8H), 7.16 (dd, *J* = 17.4, 7.9 Hz, 6H), 2.92 (s, 2H), 1.96 – 1.83 (m, 4H), 1.30 – 1.14 (m, 12H), 0.83 (t, *J* = 7.0 Hz, 6H).

^{13}C NMR (100 MHz, CD_2Cl_2) δ 166.94, 165.22, 150.83 (d, *J* = 7.4 Hz), 134.71, 132.54, 131.13, 129.80, 129.75, 129.16, 128.79, 128.50, 128.38, 125.20, 120.27 (d, *J* = 5.0 Hz), 90.60 (d, *J* = 8.1 Hz), 40.44 (d, *J* = 5.0 Hz), 38.02 (d, *J* = 3.6 Hz), 31.92, 23.10, 22.61, 14.15.

^{31}P NMR (160 MHz, CD_2Cl_2) -17.63.

HRMS (ESI-TOF) m/z calcd. for $C_{38}H_{45}NO_6P^+$ ($[M+H]^+$) 642.2979, found: 642.2979.

2-Cyclohexyl-4-(((diphenylmethylene)amino)oxy)-4-oxobutan-2-yl diphenyl phosphate (S41)

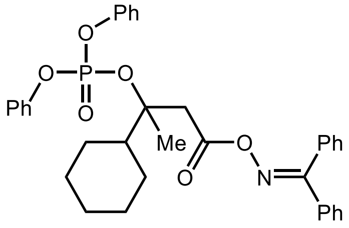

Prepared following the general procedure A outlined above starting from 1-cyclohexylethan-1-one and benzyl 2-bromoacetate on 20 mmol scale to give **S41** (1.8

g, 15% total yield) as a colorless oil.

^{1}H NMR (400 MHz, CD_2Cl_2) δ 7.61 – 7.53 (m, 2H), 7.49 – 7.27 (m, 12H), 7.24 – 7.14 (m, 6H), 2.91 (q, *J* = 15.0 Hz, 2H), 1.85 – 1.78 (m, 1H), 1.70 (d, *J* = 10.6 Hz, 4H), 1.62 (s, 3H), 1.24 – 0.86 (m, 6H).

^{13}C NMR (100 MHz, CD_2Cl_2) δ 167.04, 165.24, 151.15 (d, *J* = 7.7 Hz), 135.11, 132.89, 131.34, 130.05, 130.04, 129.98, 129.21, 129.04, 128.81, 128.62, 125.52, 120.62 (d, *J* = 12.9 Hz), 89.88 (d, *J* = 8.6 Hz), 46.38 (d, *J* = 6.5 Hz), 41.75 (d, *J* = 3.2 Hz), 27.27, 27.14, 26.65, 26.62, 26.48, 23.54.

^{31}P NMR (160 MHz, CD_2Cl_2) -16.65.

HRMS (ESI-TOF) m/z calcd. for $C_{35}H_{37}NO_6P^+$ ($[M+H]^+$) 598.2353, found: 598.2350.

3) Reaction Investigations and Optimizations

Preliminary investigations of the reaction

An oven-dried vial (8 mL) containing a stirring bar was charged with **1** (27 mg, 0.05 mmol, 1.0 equiv.), Ir[(dF(CF3)ppy]2(dtbbpy)PF6 (1 mg, 0.001 mmol, 2 mol%), then solvent (0.5 mL) was added to the mixture in the glovebox. The reaction was stirred and irradiated using 40 W Kessil 427 nm blue LED lamps (5 cm away, room temperature) for 10 min. TLC was used to monitor reaction, which indicated all started material was converted in 10 min. 3,4,5-Trichloropyridine (internal standard, 9 mg, 0.05 mmol, 1.0 equiv.) was added to the reaction mixture, then it was analyzed by ^{1}H NMR.

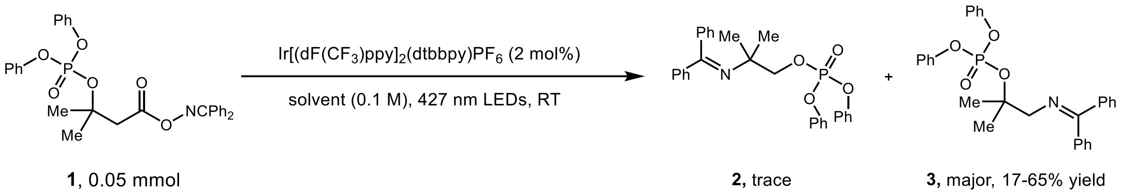

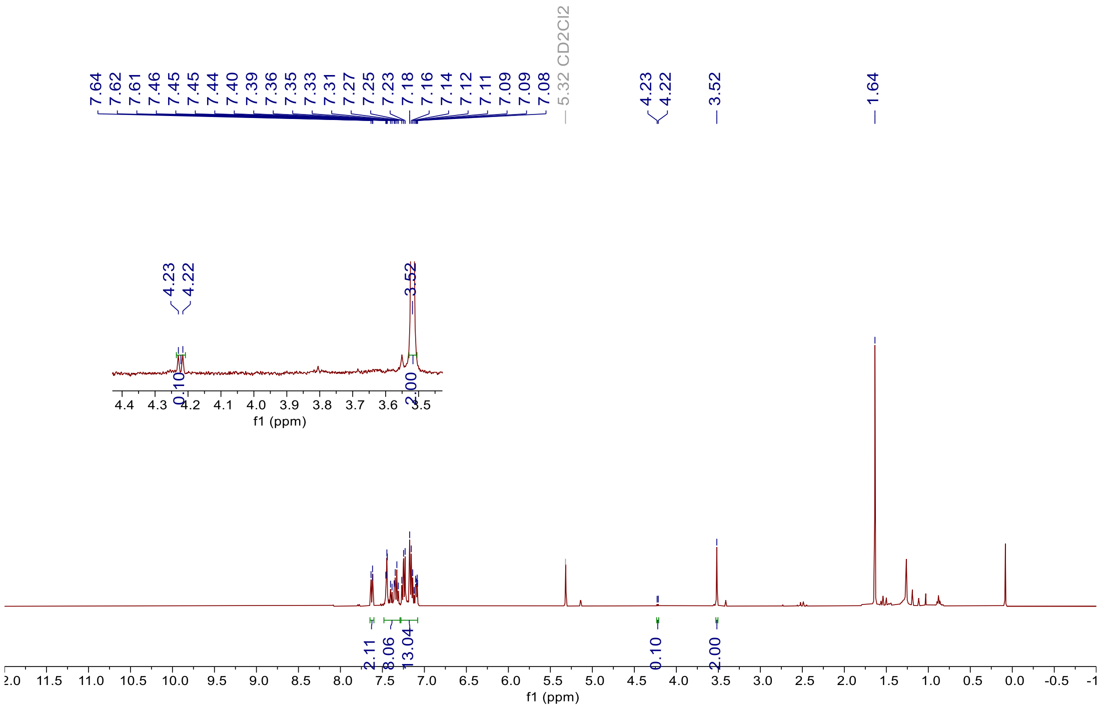

Figure S1 ^{1}H NMR of **3** with **2** (same polarity) after column chromatography separation

We found that nitrogen-oxygen dual functional group transposition (dFGT) product **2** was always trace amounts, and the decarboxylative homocoupling product **3** was the major product. The screened solvents include MeCN, THF, DCM, DCE,

acetone, 1,4-dioxane, EtOAc, MTBE, DMA, toluene, etc, the yield of **3** was 17-65% yield. We also screened other PC, such as thioxanthone and 4CzIPN, **3** was also the major product.

Reaction Optimizations

An oven-dried vial (8 mL) containing a stirring bar was charged with **11** (15 mg, 0.025 mmol, 1.0 equiv.), PC, then solvent (0.25 mL) was added to the mixture in the glovebox. The reaction was stirred and irradiated using 40 W Kessil 427 nm blue LED lamps (5 cm away, room temperature) or the Shanshi photoreactor to maintain 0 °C for 10 min. TLC was used to monitor reaction, which indicated all started material was converted in 10 min. 3,4,5-Trichloropyridine (internal standard, 9 mg, 0.05 mmol, 1.0 equiv.) was added to the reaction mixture, then it was analyzed by ^{1}H NMR.

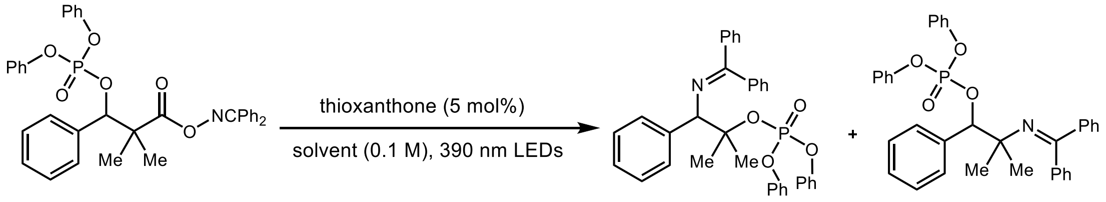

11, 0.025 mmol **12** **13**, N.D.

entry	temperature	solvent	yield[a]
1	R.T.	MeCN	15%
2	R.T.	DCM	29%
3	R.T.	acetone	19%
4	R.T.	DCE	17%
5	R.T.	THF	55%
6	R.T.	EtOAc	49%
7	R.T.	DME	46%
8	R.T.	1,4-dioxane	57%
9	R.T.	DMA	22%
10	R.T.	MTBE	42%
11	R.T.	toluene	trace
12	0 °C	DCM	43%
13	0 °C	acetone	50%
14	0 °C	DCE	32%
15	0 °C	DME	51%
16	0 °C	EtOAc	62%
17[b]	0 °C	1,4-dioxane	46%
18	0 °C	THF	**64%**

[a]NMR yield using 3,4,5-trichloropyridine as the internal standard; [b]freezing point of 1,4-dioxane is 11.8 °C.

Figure S2. Evaluation of different solvents and temperatures.

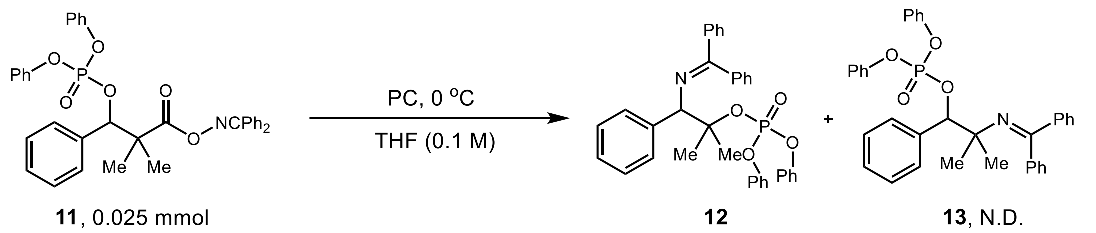

entry	PC	light	yield[a]
1	TXT (5 mol%)	390 nm	64%
2	Ir[(dF(CF$_3$)ppy]$_2$(dtbbpy)PF$_6$ (2 mol%)	427 nm	**73%**
3	4CzIPN (5 mol%)	427 nm	15%
4	Ir(*p*-Fppy)$_2$(dtbbpy)PF$_6$ (2 mol%)	427 nm	N.D.
5	Ir(ppy)$_2$(dtbbpy)PF$_6$ (2 mol%)	427 nm	N.D.
6	*fac*-Ir(ppy)$_3$ (2 mol%)	427 nm	trace
7	/	427 nm	N.D.

[a]NMR yield using 3,4,5-trichloropyridine as the internal standard.

Figure S3. Evaluation of different PCs.

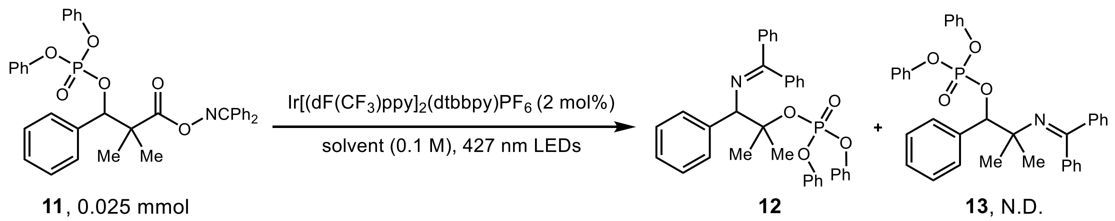

entry	temperature	solvent	yield[a]
1	0 °C	THF	**73% (67%)[d]**
2	R.T.	THF	46%
3[b]	0 °C	THF	**72%**
4	0 °C	acetone	43%
5	0 °C	EtOAc	71%
6	0 °C	DME	65%
7[c]	0 °C	THF	N.D.

[a]NMR yield using 3,4,5-trichloropyridine as the internal standard; [b]with 440 nm LEDs; [c]in dack; [d]0.2 mmol scale, 1 h, isolated yield is shown in parentheses.

Figure S4. Re-evaluation of different solvents.

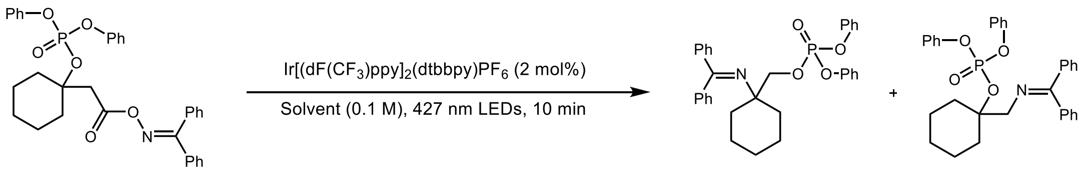

S32, 0.05 mmol **32** **bp32**

entry	temperature	solvent	yield (32)[a]	yield (bp32)[b]
1	R.T.	MeCN	46% (40%)[d]	11%
2	0 °C	MeCN	23%	25%
2[b]	R.T.	MeCN	45%	10%
3	R.T.	DCE	26%	13%
4	R.T.	EtOAc	14%	37%
5	R.T.	Acetone	32%	24%
6	R.T.	toluene	22%	15%
7	R.T.	1.4-dioxane	12%	23%
8	R.T.	DMSO	trace	trace
9	R.T.	DMF	30%	10%
10	R.T.	DMA	27%	12%
11	R.T.	MeCN	N.D.	N.D.
12[c]				

[a]NMR yield using 3,4,5-trichloropyridine as the internal standard; [b]with 440 nm LEDs; [c]with PC or in dack;[d]0.2 mmol scale, 1 h, isolated yield is shown in parentheses.

Figure S5. Evaluation of different solvents and temperatures for optimization reactions of **S32**

4) Experiment Procedure of N-O Dual Functional Group Transposition Reaction

General Procedure C

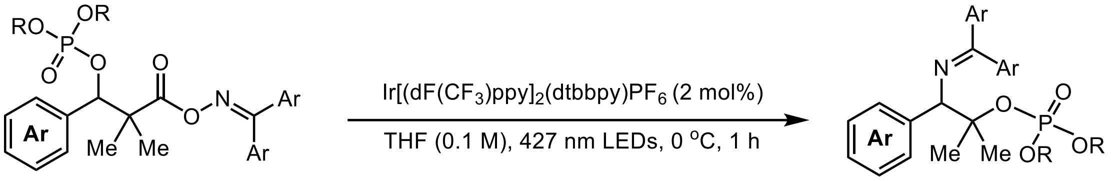

An oven-dried vial (8 mL) containing a stirring bar was charged with activated phosphatoxyalkyl oxime ester (0.2 mmol, 1.0 equiv.), Ir[(dF(CF_3)ppy]$_2$(dtbbpy)PF_6 (4.5 mg, 0.004 mmol, 2 mol%), then THF (2 mL) was added to the mixture in the glovebox. The reaction was stirred and irradiated using Shanshi photoreactor to maintain at 0 °C for 1 h. After the starting material was consumed, the reaction was concentrated in vacuo. The residue was purified by flash chromatography on silica gel (PE/EA = 20/1 to 10/1) to afford the desired product **12**, **14-31**.

General Procedure D

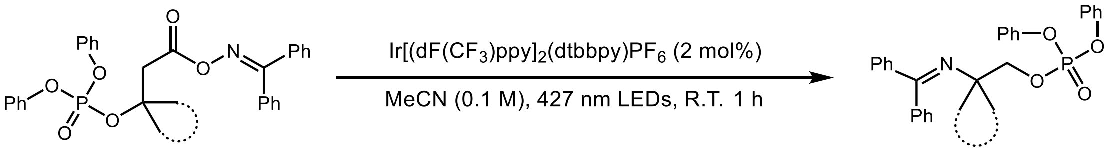

An oven-dried vial (8 mL) containing a stirring bar was charged with unactivated phosphatoxyalkyl oxime ester (0.2 mmol, 1.0 equiv.), Ir[(dF(CF_3)ppy]$_2$(dtbbpy)PF_6 (4.5 mg, 0.004 mmol, 2 mol%), then MeCN (2 mL) was added to the mixture in the glovebox. The reaction was stirred and irradiated using 40 W 427 nm Kessil blue LED lamps (5 cm away, room temperature) for 1 h. After the starting material was consumed, the reaction was concentrated in vacuo. The residue was purified by flash chromatography on silica gel (PE/EA = 20/1 to 10/1) to afford the desired product **32-41**.

.

5) Experimental Date

1-((Diphenylmethylene)amino)-2-methyl-1-phenylpropan-2-yl diphenyl phosphate (12)

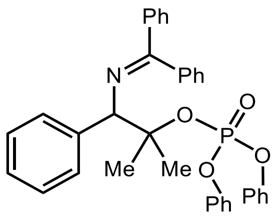

Prepared following the general procedure C from **11** (121 mg, 0.2 mmol, 1.0 equiv.). Purification by flash chromatography (PE/EA = 20/1 to 10/1) provided the title compound (75.3 mg, 67% yield) as a viscous liquid.

^{1}H NMR (400 MHz, CDCl$_3$) δ 7.70 (d, *J* = 7.1 Hz, 2H), 7.34 (dd, *J* = 8.7, 6.0 Hz, 5H), 7.22 – 7.15 (m, 10H), 7.11 – 7.03 (m, 6H), 6.79 (d, *J* = 7.1 Hz, 2H), 4.40 (s, 1H), 1.68 (s, 3H), 1.52 (s, 3H).

^{13}C NMR (100 MHz, CDCl$_3$) δ 168.44, 150.89 (d, *J* = 7.9 Hz), 150.81 (d, *J* = 8.1 Hz), 140.72, 139.80, 136.59, 130.34, 129.65, 129.42, 128.83, 128.45, 128.32, 128.18, 127.90, 127.75, 127.36, 125.02, 120.42 (d, *J* = 5.2 Hz), 120.37 (d, *J* = 4.0 Hz), 89.74 (d, *J* = 8.2 Hz), 74.31 (d, *J* = 10.4 Hz), 24.65, 24.59.

^{31}P NMR (160 MHz, CDCl$_3$) δ -16.98.

HRMS (ESI-TOF) m/z calcd. for $C_{35}H_{33}NO_4P^+$ ($[M+H]^+$) 562.2142, found: 562.2140.

1-(4-(Tert-butyl)phenyl)-1-((diphenylmethylene)amino)-2-methylpropan-2-yl diphenyl phosphate (14)

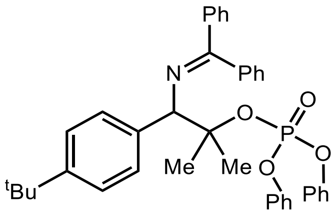

Prepared following the general procedure C from **S14** (132 mg, 0.2 mmol, 1.0 equiv.). Purification by flash chromatography (PE/EA = 20/1 to 10/1) provided the title compound (64.3 mg, 52% yield) as a viscous liquid.

^{1}H NMR (400 MHz, CDCl$_3$) δ 7.72 – 7.67 (m, 2H), 7.42 – 7.31 (m, 6H), 7.20 (dd, *J* = 12.4, 8.2 Hz, 4H), 7.16 – 7.03 (m, 10H), 6.82 (d, *J* = 7.0 Hz, 2H), 4.40 (s, 1H), 1.67 (s, 3H), 1.50 (s, 3H), 1.30 (s, 9H).

^{13}C NMR (100 MHz, CDCl$_3$) δ 168.04, 150.92 (d, *J* = 7.7 Hz), 150.82 (d, *J* = 7.5 Hz), 150.11, 139.93, 137.57, 136.62, 130.23, 129.62, 129.00, 128.83, 128.38, 128.27, 128.14, 127.88, 124.99, 124.73, 120.47 (d, *J* = 2.8 Hz), 120.42 (d, *J* = 2.9 Hz), 89.93 (d, *J* = 8.3 Hz), 74.11 (d, *J* = 10.6 Hz), 34.57, 31.54, 24.75, 24.47.

^{31}P NMR (160 MHz, CDCl$_3$) δ -16.32.

HRMS (ESI-TOF) m/z calcd. for $C_{39}H_{41}NO_4P^+$ ($[M+H]^+$) 618.2768, found: 618.2768.

1-((Diphenylmethylene)amino)-1-(4-fluorophenyl)-2-methylpropan-2-yl diphenyl phosphate (15)

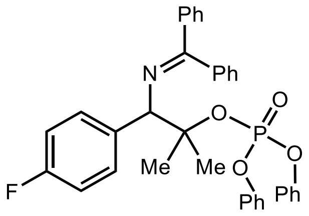

Prepared following the general procedure C from **S15** (125 mg, 0.2 mmol, 1.0 equiv.). Purification by flash chromatography (PE/EA = 20/1 to 10/1) provided the title compound (75.4 mg, 65% yield) as a viscous liquid.

^{1}H NMR (400 MHz, CDCl$_3$) δ 7.69 (d, *J* = 7.2 Hz, 2H), 7.44 – 7.32 (m, 6H), 7.25 – 7.02 (m, 12H), 6.87 (dd, *J* = 12.6, 8.7 Hz, 2H), 6.79 (d, *J* = 7.2 Hz, 2H), 4.37 (s, 1H), 1.63 (s, 3H), 1.54 (s, 3H).

^{13}C NMR (100 MHz, $CDCl_3$) δ 168.79, 162.13 (d, J = 245.4 Hz), 150.88 (d, J = 8.1 Hz), 150.80 (d, J = 8.1 Hz), 139.62, 136.50, 136.42 (d, J = 3.2 Hz), 130.81 (d, J = 7.9 Hz), 130.48, 129.67, 128.81, 128.56, 128.40, 128.23, 127.62, 125.08, 120.35 (d, J = 4.9 Hz), 120.30 (d, J = 5.0 Hz), 114.71 (d, J = 21.1 Hz), 89.29 (d, J = 7.5 Hz), 73.43 (d, J = 10.6 Hz), 24.87, 24.34.

^{31}P NMR (160 MHz, $CDCl_3$) δ -16.43.

^{19}F NMR (376 MHz, $CDCl_3$) δ -115.19.

HRMS (ESI-TOF) m/z calcd. for $C_{35}H_{32}FNO_4P^+$ ($[M+H]^+$) 580.2048, found: 580.2043.

1-(4-Chlorophenyl)-1-((diphenylmethylene)amino)-2-methylpropan-2-yl diphenyl phosphate (16)

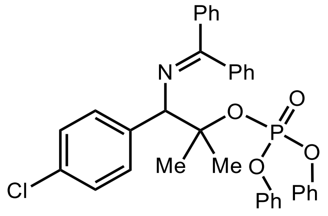

Prepared following the general procedure C from **S16** (128 mg, 0.2 mmol, 1.0 equiv.). Purification by flash chromatography (PE/EA = 20/1 to 10/1) provided the title compound (68.0 mg, 57% yield) as a viscous liquid.

^{1}H NMR (400 MHz, CD_2Cl_2) δ 7.71 – 7.65 (m, 2H), 7.45 – 7.40 (m, 2H), 7.39 – 7.33 (m, 4H), 7.27 – 7.05 (m, 14H), 6.81 (d, J = 7.0 Hz, 2H), 4.37 (s, 1H), 1.60 (s, 3H), 1.53 (s, 3H).

^{13}C NMR (100 MHz, CD_2Cl_2) δ 169.45, 151.14 (d, J = 8.0 Hz), 151.06 (d, J = 8.1 Hz), 139.88, 139.51, 136.68, 133.22, 131.01, 130.76, 129.96, 129.04, 128.87, 128.69,

128.42, 128.23, 127.86, 125.41 (d, J = 4.2 Hz), 120.56 (d, J = 4.5 Hz), 120.52 (d, J = 5.1 Hz), 89.25 (d, J = 8.0 Hz), 73.75 (d, J = 10.4 Hz), 25.09, 24.35.

^{31}P NMR (160 MHz, CD_2Cl_2) δ -16.79.

HRMS (ESI-TOF) m/z calcd. for $C_{35}H_{32}ClNO_4P^+$ ($[M+H]^+$) 596.1752, found: 596.1752.

1-(2-Bromophenyl)-1-((diphenylmethylene)amino)-2-methylpropan-2-yl diphenyl phosphate (17)

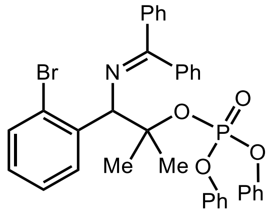

Prepared following the general procedure C from **S17** (137 mg, 0.2 mmol, 1.0 equiv.). Purification by flash chromatography (PE/EA = 20/1 to 10/1) provided the title compound (73.1 mg, 57% yield) as a viscous liquid.

^{1}H NMR (400 MHz, CD_2Cl_2) δ 7.79 – 7.65 (m, 3H), 7.50 – 7.33 (m, 7H), 7.30 – 7.07 (m, 12H), 6.83 (d, J = 6.5 Hz, 2H), 5.04 (s, 1H), 1.69 (s, 3H), 1.61 (s, 3H).

^{13}C NMR (100 MHz, CD_2Cl_2) δ 169.90, 151.23 (d, J = 1.6 Hz), 151.16 (d, J = 2.0 Hz), 140.07, 139.96, 137.17, 132.95, 132.71, 130.71, 129.99, 129.93, 129.10, 129.08, 129.02, 128.72, 128.40, 127.88, 127.36, 125.35, 120.70 (d, J = 2.1 Hz), 120.65 (d, J = 2.3 Hz), 90.31 (d, J = 8.1 Hz), 71.34 (d, J = 10.6 Hz), 24.95, 24.74.

^{31}P NMR (160 MHz, CD_2Cl_2) δ -16.63.

HRMS (ESI-TOF) m/z calcd. for $C_{35}H_{32}BrNO_4P^+$ ($[M+H]^+$) 640.1247, found: 640.1243.

1-((Diphenylmethylene)amino)-1-(4-iodophenyl)-2-methylpropan-2-yl diphenyl phosphate (18)

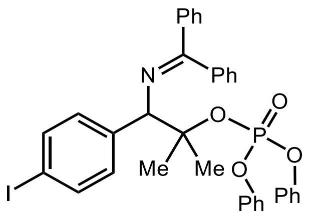

Prepared following the general procedure C from **S18** (146 mg, 0.2 mmol, 1.0 equiv.). Purification by flash chromatography (PE/EA = 20/1 to 10/1) provided the title compound (89.4 mg, 65% yield) as a viscous liquid.

^{1}H NMR (400 MHz, CD_2Cl_2) δ 7.72 – 7.66 (m, 2H), 7.54 (d, *J* = 8.4 Hz, 2H), 7.44 – 7.34 (m, 6H), 7.29 – 7.14 (m, 6H), 7.12 – 7.03 (m, 4H), 6.93 (d, *J* = 8.3 Hz, 2H), 6.83 (d, *J* = 6.9 Hz, 2H), 4.35 (s, 1H), 1.60 (s, 3H), 1.54 (s, 3H).

^{13}C NMR (100 MHz, CD_2Cl_2) δ 169.50, 151.11 (d, *J* = 8.0 Hz), 151.03 (d, *J* = 7.2 Hz), 140.64, 139.86, 137.20, 136.66, 131.61, 130.76, 129.97, 129.04, 128.88, 128.69, 128.42, 127.86, 125.42 (d, *J* = 3.1 Hz), 120.56 (d, *J* = 4.0 Hz), 120.52 (d, *J* = 4.8 Hz), 93.19, 89.15 (d, *J* = 8.1 Hz), 73.92 (d, *J* = 10.4 Hz), 25.14, 24.31.

^{31}P NMR (160 MHz, CD_2Cl_2) δ -16.82.

HRMS (ESI-TOF) m/z calcd. for $C_{35}H_{32}INO_4P^+$ ($[M+H]^+$) 688.1108, found: 688.1101.

1-((Diphenylmethylene)amino)-2-methyl-1-(4-(trifluoromethyl)phenyl)propan-2-yl diphenyl phosphate (19)

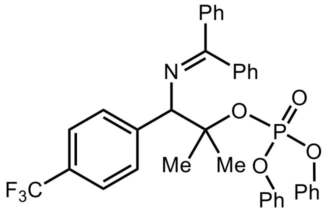

Prepared following the general procedure C from **S19** (135 mg, 0.2 mmol, 1.0 equiv.). Purification by flash chromatography (PE/EA = 20/1 to 10/1) provided the title compound (90.7 mg, 72% yield) as a viscous liquid.

^{1}H NMR (400 MHz, CD_2Cl_2) δ 7.68 (d, J = 7.5 Hz, 2H), 7.43 (dd, J = 10.7, 7.0 Hz, 4H), 7.39 – 7.33 (m, 4H), 7.28 (d, J = 8.1 Hz, 2H), 7.22 (q, J = 7.4 Hz, 4H), 7.17 – 7.12 (m, 2H), 7.09 – 7.04 (m, 4H), 6.80 (d, J = 7.2 Hz, 2H), 4.45 (s, 1H), 1.60 (s, 3H), 1.54 (s, 3H).

^{13}C NMR (100 MHz, CD_2Cl_2) δ 169.94, 151.16 (d, J = 7.6 Hz), 151.05 (d, J = 7.5 Hz), 145.06, 139.81, 136.66, 130.88, 130.10, 129.99, 129.97, 129.10, 128.97, 128.78, 128.47, 127.84, 126.90 (q, J = 285.4 Hz), 125.48 (d, J = 3.7 Hz), 124.96 (d, J = 3.8 Hz), 120.57 (d, J = 4.9 Hz), 120.50 (d, J = 5.0 Hz), δ 89.01 (d, J = 7.8 Hz), 74.06 (d, J = 10.5 Hz), 25.35, 24.28.

^{31}P NMR (160 MHz, CD_2Cl_2) δ -16.85.

^{19}F NMR (376 MHz, CD_2Cl_2) δ -62.67.

HRMS (ESI-TOF) m/z calcd. for $C_{36}H_{32}F_3NO_4P^+$ ($[M+H]^+$) 630.2016, found: 630.2014.

1-([1,1'-Biphenyl]-4-yl)-1-((diphenylmethylene)amino)-2-methylpropan-2-yl diphenyl phosphate (20)

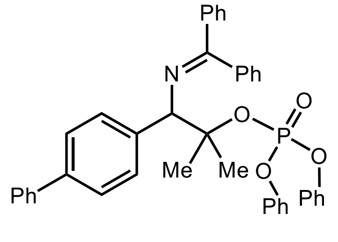

Prepared following the general procedure C from **S20** (136 mg, 0.2 mmol, 1.0 equiv.).

Purification by flash chromatography (PE/EA = 20/1 to 10/1) provided the title compound (82.9 mg, 65% yield) as a white solid.

^{1}H NMR (400 MHz, CDCl$_3$) δ 7.74 (d, *J* = 7.1 Hz, 2H), 7.61 (d, *J* = 7.3 Hz, 2H), 7.50 – 7.33 (m, 12H), 7.28 (s, 1H), 7.22 – 7.08 (m, 10H), 6.86 (d, *J* = 7.1 Hz, 2H), 4.47 (s, 1H), 1.72 (s, 3H), 1.59 (s, 3H).

^{13}C NMR (100 MHz, CDCl$_3$) δ 168.54, 150.85 (d, *J* = 7.8 Hz), 150.74 (d, *J* = 8.0 Hz), 140.92, 140.03, 139.78, 139.74, 136.52, 130.37, 129.78, 129.63, 128.89, 128.83, 128.49, 128.35, 128.19, 127.75, 127.34, 127.08, 126.52, 125.01, 120.39 (d, *J* = 4.8 Hz), 120.34 (d, *J* = 4.0 Hz), 89.62 (d, *J* = 8.1 Hz), 74.05 (d, *J* = 10.3 Hz), 24.74, 24.55.

^{31}P NMR (160 MHz, CDCl$_3$) δ -16.93.

HRMS (ESI-TOF) m/z calcd. for $C_{41}H_{37}NO_4P^+$ ($[M+H]^+$) 638.2455, found: 638.2452.

1-((Diphenylmethylene)amino)-2-methyl-1-(4-(4,4,5,5-tetramethyl-1,3,2-dioxaborolan-2-yl)phenyl)propan-2-yl diphenyl phosphate (21)

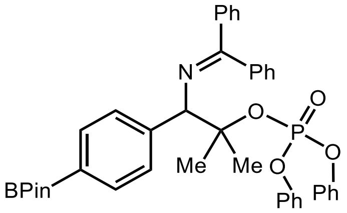

Prepared following the general procedure C from **S21** (146.4 mg, 0.2 mmol, 1.0 equiv.). Purification by flash chromatography (PE/EA = 20/1 to 10/1) provided the title compound (61.9 mg, 45% yield) as a viscous liquid.

^{1}H NMR (400 MHz, CD$_2$Cl$_2$) δ 7.70 – 7.66 (m, 2H), 7.60 (d, *J* = 8.1 Hz, 2H), 7.44 – 7.38 (m, 2H), 7.37 – 7.31 (m, 5H), 7.22 – 7.12 (m, 8H), 7.07 – 7.02 (m, 3H), 6.79 (d, *J* = 7.0 Hz, 2H), 4.41 (s, 1H), 1.63 (s, 3H), 1.50 (s, 3H), 1.32 (s, 12H).

^{13}C NMR (100 MHz, CD_2Cl_2) δ 169.13, 151.15 (d, *J* = 7.9 Hz), 151.07 (d, *J* = 7.8 Hz), 144.05, 140.08, 136.78, 134.48, 130.61, 129.96, 129.92, 129.07 (d, *J* = 2.1 Hz), 128.77, 128.59, 128.38, 127.98, 125.33 (d, *J* = 2.8 Hz), 125.05, 120.60 (d, *J* = 4.0 Hz), 120.56 (d, *J* = 4.2 Hz), 89.69 (d, *J* = 8.2 Hz), 84.11, 74.68 (d, *J* = 10.4 Hz), 25.04, 24.85, 24.66.

^{31}P NMR (160 MHz, CD_2Cl_2) δ -16.75.

HRMS (ESI-TOF) m/z calcd. for $C_{41}H_{44}BNO_6P^+$ ($[M+H]^+$) 688.2994, found: 688.2990.

Methyl 4-(2-((diphenoxyphosphoryl)oxy)-1-((diphenylmethylene)amino)-2-methylpropyl)benzoate (22)

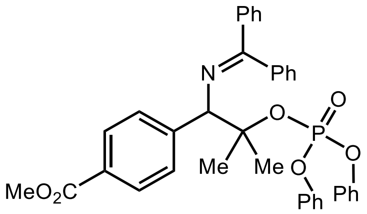

Prepared following the general procedure C from **S22** (133 mg, 0.2 mmol, 1.0 equiv.). Purification by flash chromatography (PE/EA = 20/1 to 10/1) provided the title compound (80.5 mg, 65% yield) as a viscous liquid.

^{1}H NMR (400 MHz, $CDCl_3$) δ 7.86 (d, *J* = 8.3 Hz, 2H), 7.74 – 7.69 (m, 2H), 7.45 – 7.32 (m, 6H), 7.25 – 7.15 (m, 6H), 7.13 – 7.05 (m, 6H), 6.78 (d, *J* = 7.1 Hz, 2H), 4.45 (s, 1H), 3.92 (s, 3H), 1.67 (s, 3H), 1.57 (s, 3H).

^{13}C NMR (100 MHz, $CDCl_3$) δ 169.27, 167.12, 150.86 (d, *J* = 8.0 Hz), 150.65 (d, *J* = 7.9 Hz), 145.84, 139.40, 136.33, 130.56, 129.63, 129.36, 129.09, 129.07, 128.80, 128.60, 128.41, 128.21, 127.50, 125.08, 120.28 (d, *J* = 5.0 Hz), 120.23 (d, *J* = 4.8 Hz), 89.02 (d, *J* = 8.0 Hz), 73.92 (d, *J* = 10.6 Hz), 52.21, 24.99, 24.29.

^{31}P NMR (160 MHz, $CDCl_3$) δ -17.02.

HRMS (ESI-TOF) m/z calcd. for $C_{37}H_{35}NO_6P^+$ ($[M+H]^+$) 620.2197, found: 620.2196.

1-((Diphenylmethylene)amino)-1-(4-isocyanophenyl)-2-methylpropan-2-yl diphenyl phosphate (23)

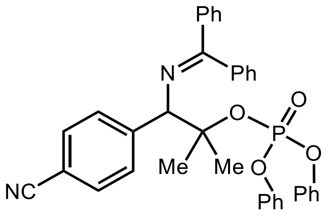

Prepared following the general procedure C from **S23** (126 mg, 0.2 mmol, 1.0 equiv.). Purification by flash chromatography (PE/EA = 20/1 to 10/1) provided the title compound (85.7 mg, 73% yield) as a white solid.

^{1}H NMR (400 MHz, CDCl$_3$) δ 7.71 – 7.66 (m, 2H), 7.43 (t, *J* = 6.7 Hz, 4H), 7.39 – 7.34 (m, 4H), 7.26 – 7.19 (m, 6H), 7.15 (dd, *J* = 6.8, 3.3 Hz, 2H), 7.07 (d, *J* = 8.2 Hz, 4H), 6.78 (d, *J* = 7.1 Hz, 2H), 4.44 (s, 1H), 1.62 (s, 3H), 1.58 (s, 3H).

^{13}C NMR (100 MHz, CDCl$_3$) δ 169.95, 150.75 (d, *J* = 8.0 Hz), 150.67 (d, *J* = 8.0 Hz), 145.95, 139.17, 136.20, 131.55, 130.79, 130.05, 129.70, 128.82, 128.79, 128.55, 128.28, 127.38, 125.19, 125.18, 120.20 (d, *J* = 5.1 Hz), 120.12 (d, *J* = 5.1 Hz), 118.96, 111.09, 88.52 (d, *J* = 7.9 Hz), 73.66 (d, *J* = 10.5 Hz), 25.40, 23.99.

^{31}P NMR (160 MHz, CDCl$_3$) δ -17.19.

HRMS (ESI-TOF) m/z calcd. for $C_{36}H_{32}N_2O_4P^+$ ($[M+H]^+$) 587.2094, found: 587.2094.

1-((Diphenylmethylene)amino)-2-methyl-1-(4-((methylperoxy)thio)phenyl)propan-2-yl diphenyl phosphate (24)

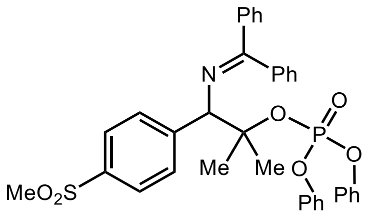

Prepared following the general procedure C from **S24** (137 mg, 0.2 mmol, 1.0 equiv.). Purification by flash chromatography (PE/EA = 20/1 to 10/1) provided the title compound (81.9 mg, 64% yield) as a viscous liquid.

^{1}H NMR (400 MHz, CDCl$_3$) δ 7.74 (d, *J* = 8.4 Hz, 2H), 7.69 (d, *J* = 7.2 Hz, 2H), 7.44 (dd, *J* = 14.2, 7.3 Hz, 2H), 7.39 – 7.34 (m, 6H), 7.24 – 7.18 (m, 4H), 7.14 (dd, *J* = 6.8, 3.2 Hz, 2H), 7.10 – 7.03 (m, 4H), 6.79 (d, *J* = 7.1 Hz, 2H), 4.49 (s, 1H), 3.04 (s, 3H), 1.63 (s, 3H), 1.58 (s, 3H).

^{13}C NMR (100 MHz, CDCl$_3$) δ 169.81, 150.66 (d, *J* = 7.9 Hz), 150.56 (d, *J* = 7.5 Hz), 146.80, 139.23, 139.11, 136.10, 130.75, 130.23, 129.68, 129.67, 128.79, 128.53, 128.24, 127.36, 126.78, 125.20, 120.16 (d, *J* = 4.9 Hz), 120.07 (d, *J* = 5.0 Hz), 88.50 (d, *J* = 7.9 Hz), 73.60 (d, *J* = 10.5 Hz), 44.50, 25.24, 24.06.

^{31}P NMR (160 MHz, CDCl$_3$) δ -17.11.

HRMS (ESI-TOF) m/z calcd. for $C_{36}H_{35}SNO_6P^+$ ($[M+H]^+$) 640.1917, found: 640.1915.

1-((Diphenylmethylene)amino)-2-methyl-1-(4-nitrophenyl)propan-2-yl diphenyl phosphate (25)

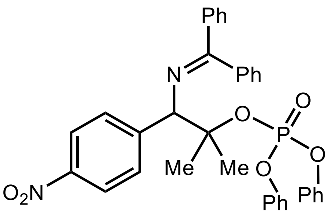

Prepared following the general procedure C from **S25** (130 mg, 0.2 mmol, 1.0 equiv.).

Purification by flash chromatography (PE/EA = 20/1 to 10/1) provided the title compound (40.1 mg, 33% yield) as a viscous liquid.

^{1}H NMR (400 MHz, CDCl$_3$) δ 7.97 (d, *J* = 8.8 Hz, 2H), 7.71 – 7.66 (m, 2H), 7.47 – 7.32 (m, 7H), 7.29 (s, 1H), 7.21 (dd, *J* = 7.6, 3.1 Hz, 4H), 7.13 (d, *J* = 7.0 Hz, 2H), 7.06 (d, *J* = 7.9 Hz, 4H), 6.77 (d, *J* = 7.1 Hz, 2H), 4.47 (s, 1H), 1.62 (s, 3H), 1.60 (s, 3H).

^{13}C NMR (100 MHz, CDCl$_3$) δ 170.15, 150.75 (d, *J* = 7.5 Hz), 150.67 (d, *J* = 8.0 Hz), 147.99, 147.10, 139.12, 136.18, 130.90, 130.11, 129.74, 129.73, 128.87, 128.64, 128.35, 127.38, 125.23, 122.95, 120.20 (d, *J* = 4.9 Hz), 120.11 (d, *J* = 5.0 Hz), 88.45 (d, *J* = 7.8 Hz), 73.46 (d, *J* = 10.5 Hz), 25.57, 23.95.

^{31}P NMR (160 MHz, CDCl$_3$) δ -17.23.

HRMS (ESI-TOF) m/z calcd. for $C_{35}H_{32}N_2O_6P^+$ ($[M+H]^+$) 607.1993, found: 607.1991.

1-(3,5-Dimethylphenyl)-1-((diphenylmethylene)amino)-2-methylpropan-2-yl diphenyl phosphate (26)

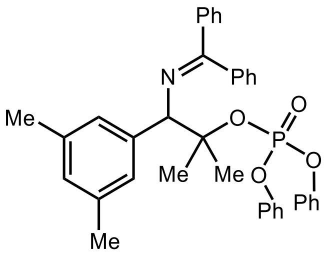

Prepared following the general procedure C from **S26** (127 mg, 0.2 mmol, 1.0 equiv.). Purification by flash chromatography (PE/EA = 20/1 to 10/1) provided the title compound (68.4 mg, 58% yield) as a viscous liquid.

^{1}H NMR (400 MHz, CD$_2$Cl$_2$) δ 7.73 – 7.67 (m, 2H), 7.44 – 7.40 (m, 2H), 7.36 (dd, *J* = 8.0, 6.6 Hz, 4H), 7.25 – 7.05 (m, 10H), 6.90 – 6.81 (m, 5H), 4.35 (s, 1H), 2.23 (s, 6H), 1.66 (s, 3H), 1.50 (s, 3H).

^{13}C NMR (100 MHz, CD_2Cl_2) δ 168.57, 151.21 (d, *J* = 8.0 Hz), 151.12 (d, *J* = 8.0 Hz), 140.74, 140.28, 137.51, 136.86, 130.50, 129.91, 129.20, 129.07, 128.69, 128.49, 128.36, 128.15, 127.41, 125.30 (d, *J* = 2.1 Hz), 120.61 (d, *J* = 1.8 Hz), 120.56 (d, *J* = 2.0 Hz)., 90.04 (d, *J* = 8.2 Hz), 74.66 (d, *J* = 10.4 Hz), 24.90, 24.67, 21.47.

^{31}P NMR (160 MHz, CD_2Cl_2) δ -16.75.

HRMS (ESI-TOF) m/z calcd. for $C_{37}H_{31}NO_4P^+$ ($[M+H]^+$) 590.2455, found: 590.2451.

1-(3,5-Dichlorophenyl)-1-((diphenylmethylene)amino)-2-methylpropan-2-yl diphenyl phosphate (27)

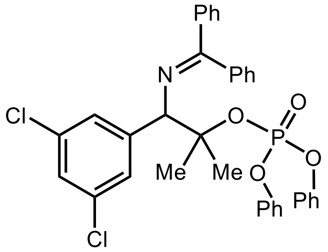

Prepared following the general procedure C from **S27** (135 mg, 0.2 mmol, 1.0 equiv.). Purification by flash chromatography (PE/EA = 20/1 to 10/1) provided the title compound (71.9 mg, 57% yield) as a white solid.

^{1}H NMR (400 MHz, CD_2Cl_2) δ 7.72 – 7.67 (m, 2H), 7.49 – 7.35 (m, 6H), 7.31 – 7.22 (m, 5H), 7.20 – 7.14 (m, 2H), 7.13 – 7.07 (m, 6H), 6.86 (d, *J* = 7.0 Hz, 2H), 4.33 (s, 1H), 1.59 (s, 3H), 1.54 (s, 3H).

^{13}C NMR (100 MHz, CD_2Cl_2) δ 170.37, 151.10 (d, *J* = 7.6 Hz), 150.98 (d, *J* = 7.3 Hz), 144.28, 139.59, 136.46, 134.53, 131.00, 130.01 (d, *J* = 4.9 Hz), 129.16, 129.08, 128.83, 128.49, 128.10, 127.81, 127.78, 125.48 (d, *J* = 4.3 Hz), 120.52 (d, *J* = 5.1 Hz), 120.47 (d, *J* = 5.1 Hz), 88.71 (d, *J* = 7.7 Hz), 73.52 (d, *J* = 10.7 Hz), 25.36, 24.17.

^{31}P NMR (160 MHz, CD_2Cl_2) δ -16.79.

HRMS (ESI-TOF) m/z calcd. for $C_{35}H_{31}Cl_2NO_4P^+$ ($[M+H]^+$) 630.1363, found: 630.1360.

1-((Diphenylmethylene)amino)-2-methyl-1-(naphthalen-2-yl)propan-2-yl diphenyl phosphate (28)

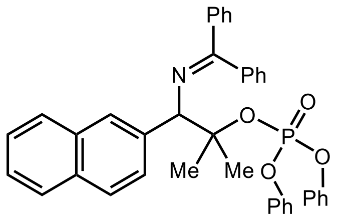

Prepared following the general procedure C from **S28** (131 mg, 0.2 mmol, 1.0 equiv.). Purification by flash chromatography (PE/EA = 20/1 to 10/1) provided the title compound (61.2 mg, 50% yield) as a white solid.

^{1}H NMR (400 MHz, CD_2Cl_2) δ 7.85 – 7.81 (m, 1H), 7.72 (dd, J = 8.6, 8.3 Hz, 4H), 7.62 (s, 1H), 7.48 – 7.33 (m, 9H), 7.18 – 7.05 (m, 8H), 7.01 (d, J = 7.8 Hz, 2H), 6.83 (d, J = 7.2 Hz, 2H), 4.59 (s, 1H), 1.69 (s, 3H), 1.57 (s, 3H).

^{13}C NMR (100 MHz, $CDCl_3$) δ 169.21, 151.16 (d, J = 7.7 Hz), 151.02 (d, J = 7.5 Hz), 140.11, 138.60, 136.82, 133.44, 133.24, 130.65, 129.88 (d, J = 5.9 Hz), 129.10, 128.80, 128.61, 128.54, 128.41, 128.39, 128.01, 127.83, 127.49, 126.16, 125.31 (d, J = 6.1 Hz), 120.59 (d, J = 4.9 Hz), 120.53 (d, J = 5.0 Hz), 89.91 (d, J = 8.1 Hz), 74.65 (d, J = 10.6 Hz), 25.02, 24.71.

^{31}P NMR (160 MHz, CD_2Cl_2) δ -16.73.

HRMS (ESI-TOF) m/z calcd. for $C_{39}H_{35}NO_4P^+$ ($[M+H]^+$) 612.2298, found: 612.2295.

1-((Diphenylmethylene)amino)-2-methyl-1-(pyridin-4-yl)propan-2-yl diphenyl phosphate (29)

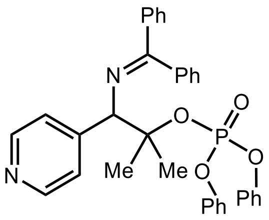

Prepared following the general procedure C from **S29** (121 mg, 0.2 mmol, 1.0 equiv.). Purification by flash chromatography (PE/EA = 2/1 to 1/1) provided the title compound (64.3 mg, 53% yield) as a viscous liquid.

^{1}H NMR (400 MHz, CD_2Cl_2) δ 8.38 (d, *J* = 5.9 Hz, 2H), 7.71 – 7.64 (m, 2H), 7.43 (dd, *J* =14.6, 7.3 Hz, 2H), 7.36 (td, *J* = 7.5, 1.9 Hz, 4H), 7.28 – 7.21 (m, 4H), 7.18 – 7.12 (m, 2H), 7.09 – 7.05 (m, 6H), 6.80 (d, *J* = 7.1 Hz, 2H), 4.38 (s, 1H), 1.60 (s, 3H), 1.55 (s, 3H).

^{13}C NMR (100 MHz, CD_2Cl_2) δ 170.37, 151.12 (d, *J* = 7.9 Hz), 151.05 (d, *J* = 7.9 Hz), 151.01, 149.65, 149.38, 139.68, 136.52, 130.96, 130.01, 129.11, 129.02, 128.81, 128.48, 127.79, 125.49 (d, *J* = 3.5 Hz), 124.71, 120.54 (d, *J* = 4.9 Hz), 120.47 (d, *J* = 5.0 Hz), 88.76 (d, *J* = 7.7 Hz), 73.55 (d, *J* = 10.3 Hz), 25.32, 24.28.

^{31}P NMR (160 MHz, CD_2Cl_2) δ -16.85

HRMS (ESI-TOF) m/z calcd. for $C_{34}H_{32}N_2O_4P^+$ ($[M+H]^+$) 563.2094, found: 563.2094.

1-((Bis(4-fluorophenyl)methylene)amino)-2-methyl-1-phenylpropan-2-yl diphenyl phosphate (30)

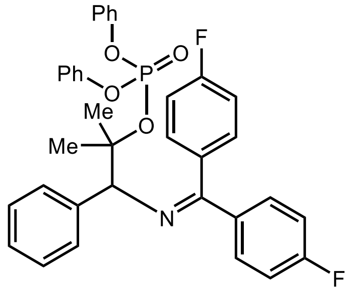

Prepared following the general procedure C from **S30** (128 mg, 0.2 mmol, 1.0 equiv.). Purification by flash chromatography (PE/EA = 20/1 to 10/1) provided the title compound (53.8 mg, 45% yield) as a viscous liquid.

^{1}H NMR (400 MHz, CD_2Cl_2) δ 7.72 – 7.65 (m, 2H), 7.27 – 7.19 (m, 9H), 7.17 – 7.12 (m, 2H), 7.11 – 7.02 (m, 8H), 6.77 (dd, *J* = 8.3, 5.5 Hz, 2H), 4.39 (s, 1H), 1.66 (s, 3H), 1.51 (s, 3H).

^{13}C NMR (100 MHz, CD_2Cl_2) δ 166.87, 165.56 (d, *J* = 251.5 Hz), 163.02 (d, *J* = 248.5 Hz), 151.21 (d, *J* = 7.2 Hz), 151.14 (d, *J* = 7.1 Hz), 140.76, 136.33 (d, *J* = 2.9 Hz), 132.49 (d, *J* = 3.6 Hz), 131.11, 131.02, 130.06, 129.98, 129.97, 129.61, 128.29, 127.81, 125.40 (d, *J* = 1.8 Hz), 120.57 (d, *J* = 2.0 Hz), 120.52 (d, *J* = 2.1 Hz), 115.73 (d, *J* = 21.6 Hz), 115.34 (d, *J* = 21.7 Hz), 89.84 (d, *J* = 8.0 Hz), 74.63 (d, *J* = 9.9 Hz), 24.91, 24.55.

^{31}P NMR (160 MHz, CD_2Cl_2) δ -16.76.

^{19}F NMR (376 MHz, CD_2Cl_2) δ -111.29 – -111.37 (m), -113.12 – -113.19 (m).

HRMS (ESI-TOF) m/z calcd. for $C_{35}H_{31}F_2NO_4P^+$ ($[M+H]^+$) 598.1954, found: 598.1949.

1-((Diphenylmethylene)amino)-2-methyl-1-phenylpropan-2-yl dimethyl phosphate (31)

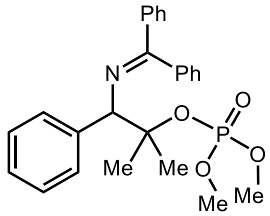

Prepared following the general procedure C from **S31** (96 mg, 0.2 mmol, 1.0 equiv.). Purification by flash chromatography (PE/EA = 20/1 to 10/1) provided the title compound (40.2 mg, 46% yield) as a viscous liquid.

^{1}H NMR (400 MHz, CD_2Cl_2) δ 7.73 – 7.67 (m, 2H), 7.42 – 7.34 (m, 6H), 7.30 – 7.24 (m, 5H), 6.95 – 6.88 (m, 2H), 4.42 (s, 1H), 3.53 (dd, *J* = 14.3, 11.2 Hz, 6H), 1.55 (s, 3H), 1.51 (s, 3H).

^{13}C NMR (100 MHz, CD_2Cl_2) δ 168.71, 141.34, 140.25, 136.96, 130.54, 129.72, 128.96, 128.79, 128.59, 128.40, 128.10, 128.04, 127.58, 6.68 (d, *J* = 7.3 Hz), 74.66 (d, *J* = 9.9 Hz), 24.95, 24.23.

^{31}P NMR (160 MHz, CD_2Cl_2) δ -3.65 – -4.07 (m).

HRMS (ESI-TOF) m/z calcd. for $C_{25}H_{29}NO_4P^+$ ($[M+H]^+$) 438.1829, found: 438.1825.

(1-((Diphenylmethylene)amino)cyclohexyl)methyl diphenyl phosphate (32)

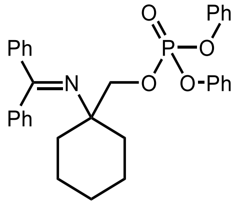

Prepared following the general procedure D from **S32** (114 mg, 0.2 mmol, 1.0 equiv.). Purification by flash chromatography (PE/EA = 20/1 to 10/1) provided the title compound (42.1 mg, 40% yield) as a viscous liquid.

^{1}H NMR (400 MHz, CD_2Cl_2) δ 7.57 – 7.54 (m, 2H), 7.39 – 7.33 (m, 4H), 7.32 – 7.24

(m, 6H), 7.18 – 7.14 (m, 8H), 4.06 (d, J = 5.3 Hz, 2H), 1.73 – 1.59 (m, 5H), 1.50 – 1.40 (m, 5H).

^{13}C NMR (150 MHz, CD_2Cl_2) δ 165.87, 151.05 (d, J = 7.1 Hz), 142.14, 139.95, 130.12, 130.07, 128.61, 128.54, 128.41, 128.18, 128.08, 125.64, 120.48 (d, J = 4.8 Hz), 74.38 (d, J = 6.7 Hz), 61.77 (d, J = 8.1 Hz), 35.19, 26.19, 22.48.

^{31}P NMR (160 MHz, CD_2Cl_2) δ -11.26.

HRMS (ESI-TOF) m/z calcd. for $C_{32}H_{33}NO_4P^+$ ($[M+H]^+$) 526.2169, found: 526.2159.

(1-((Diphenylmethylene)amino)-4,4-dimethylcyclohexyl)methyl diphenyl phosphate (33)

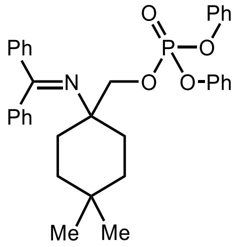

Prepared following the general procedure D from **S33** (120 mg, 0.2 mmol, 1.0 equiv.). Purification by flash chromatography (PE/EA = 20/1 to 10/1) provided the title compound (47.6 mg, 43% yield) as a viscous liquid.

^{1}H NMR (400 MHz, CD_2Cl_2) δ 7.58 – 7.53 (m, 2H), 7.38 – 7.34 (m, 4H), 7.30 – 7.21 (m, 7H), 7.19 – 7.15 (m, 7H), 4.09 (d, J = 5.2 Hz, 2H), 2.08 – 1.98 (m, 1H), 1.86 – 1.78 (m, 1H), 1.69 – 1.61 (m, 2H), 1.55 – 1.51 (m, 2H), 1.38 – 1.34 (m, 1H), 1.22 – 1.14 (m, 2H), 0.88 (d, J = 8.7 Hz, 6H).

^{13}C NMR (100 MHz, CD_2Cl_2) δ 165.96, 150.97 (d, J = 7.0 Hz), 142.04, 139.77, 130.13, 130.10, 130.05, 129.94, 128.55, 128.50, 128.40, 128.15, 128.01, 127.45, 125.63, 120.43 (d, J = 4.8 Hz), 73.83 (d, J = 6.4 Hz), 61.56 (d, J = 7.8 Hz), 35.23, 30.79, 29.81, 28.15.

^{31}P NMR (160 MHz, CD_2Cl_2) δ -11.81.

HRMS (ESI-TOF) m/z calcd. for $C_{34}H_{37}NO_4P^+$ ($[M+H]^+$) 554.2455, found: 554.2454.

(1-((Diphenylmethylene)amino)-4,4-difluorocyclohexyl)methyl diphenyl phosphate (34)

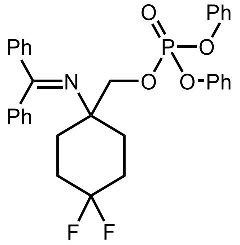

Prepared following the general procedure D from **S34** (121 mg, 0.2 mmol, 1.0 equiv.). Purification by flash chromatography (PE/EA = 20/1 to 10/1) provided the title compound (32.6 mg, 29% yield) as a viscous liquid.

^{1}H NMR (400 MHz, CD_2Cl_2) δ 7.57 – 7.52 (m, 2H), 7.43 – 7.35 (m, 5H), 7.33 – 7.24 (m, 6H), 7.18 – 7.12 (m, 7H), 3.99 (d, J = 5.4 Hz, 2H), 2.23 – 2.09 (m, 2H), 1.94 – 1.86 (m, 3H), 1.68 – 1.59 (m, 3H).

^{13}C NMR (150 MHz, CD_2Cl_2) δ 167.93, 150.93 (d, J = 8.6 Hz, 141.45, 139.06, 130.60, 130.19, 129.05, 128.76, 128.69, 128.33, 127.78, 125.80, 120.55 (t, J = 241.5 Hz), 120.46 (d, J = 4.9 Hz), 73.54 (d, J = 6.5 Hz), 60.30 (d, J = 9.7 Hz), 35.00, 31.77 (d, J = 9.2 Hz), 30.51 – 29.78 (m).

^{31}P NMR (160 MHz, CD_2Cl_2) δ -11.46.

^{13}F NMR (376 MHz, CD_2Cl_2) δ -92.40 (d, J = 236.6 Hz), -102.18 (d, J = 233.5 Hz).

HRMS (ESI-TOF) m/z calcd. for $C_{32}H_{31}F_2NO_4P^+$ ($[M+H]^+$) 562.1954, found: 562.1951.

(9-((Diphenylmethylene)amino)-3,3-dimethyl-2,4-dioxaspiro[5.5]undecan-9-yl)methyl diphenyl phosphate (35)

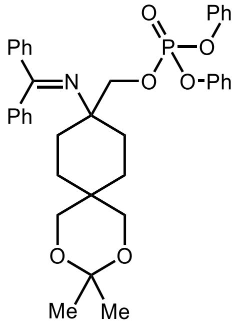

Prepared following the general procedure D from **S35** (134 mg, 0.2 mmol, 1.0 equiv.). Purification by flash chromatography (PE/EA = 20/1 to 10/1) provided the title compound (51.3 mg, 41% yield) as a viscous liquid.

^{1}H NMR (400 MHz, CDCl$_3$) δ 7.54 (d, *J* = 7.2 Hz, 2H), 7.37 – 7.29 (m, 7H), 7.22 (s, 3H), 7.14 (d, *J* = 7.9 Hz, 8H), 4.05 (d, *J* = 5.2 Hz, 2H), 3.47 (d, *J* = 25.9 Hz, 4H), 1.98 (s, 2H), 1.78 (d, *J* = 10.7 Hz, 4H), 1.61 (s, 2H), 0.95 (s, 6H).

^{13}C NMR (100 MHz, CDCl$_3$) δ 166.78, 150.63 (d, *J* = 7.3 Hz), 141.46, 139.16, 130.04, 129.83, 128.48, 128.45, 128.28, 128.00, 127.61, 125.39, 120.25 (d, *J* = 4.9 Hz), 97.53, 73.99 (d, *J* = 7.9 Hz), 70.17 (d, *J* = 7.9 Hz), 61.01, 60.93, 30.96, 30.34, 29.84, 28.25, 22.86.

^{31}P NMR (160 MHz, CDCl$_3$) δ -12.10.

HRMS (ESI-TOF) m/z calcd. for $C_{37}H_{41}F_2NO_6P^+$ ([M+H]$^+$) 626.2666, found: 626.2662.

2-((Diphenylmethylene)amino)-2-methylnonyl diphenyl phosphate (36)

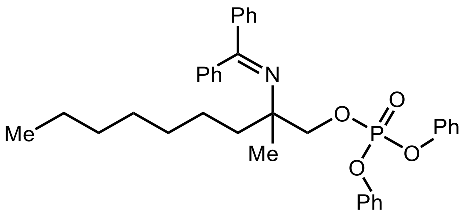

Prepared following the general procedure D from **S36** (123 mg, 0.2 mmol, 1.0 equiv.). Purification by flash chromatography (PE/EA = 20/1 to 10/1) provided the title compound (50.2 mg, 44% yield) as a viscous liquid.

^{1}H NMR (400 MHz, CD_2Cl_2) δ 7.55 – 7.50 (m, 2H), 7.42 – 7.27 (m, 10H), 7.23 – 7.15 (m, 8H), 4.26 (qd, *J* = 9.3, 4.8 Hz, 2H), 1.67 – 1.47 (m, 2H), 1.33 – 1.20 (m, 10H), 0.94 (s, 3H), 0.88 (t, *J* = 6.8 Hz, 3H).

^{13}C NMR (100 MHz, CD_2Cl_2) δ 165.71, 151.04 (d, *J* = 7.2 Hz), 141.75, 139.77, 130.10, 130.06, 128.53, 128.45, 128.33, 128.25, 128.13, 125.61, 120.41 (d, *J* = 4.9 Hz), 75.76 (d, *J* = 7.2 Hz), 62.22 (d, *J* = 9.0 Hz), 38.41, 32.24, 30.45, 29.61, 24.01, 23.66, 23.05, 14.26.

^{31}P NMR (160 MHz, CD_2Cl_2) δ -12.01.

HRMS (ESI-TOF) m/z calcd. for $C_{35}H_{41}NO_4P^+$ ($[M+H]^+$) 570.2768, found:570.2767.

2-((Diphenylmethylene)amino)-2-methylundecyl diphenyl phosphate (37)

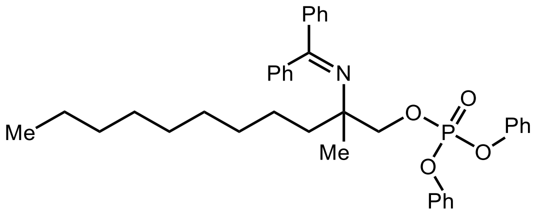

Prepared following the general procedure D from **S37** (128 mg, 0.2 mmol, 1.0 equiv.). Purification by flash chromatography (PE/EA = 20/1 to 10/1) provided the title compound (35.8 mg, 32% yield) as a viscous liquid.

^{1}H NMR (400 MHz, CD_2Cl_2) δ 7.52 (d, *J* = 7.4 Hz, 2H), 7.42 – 7.37 (m, 3H), 7.34 – 7.27 (m, 6H), 7.23 – 7.14 (m, 9H), 4.25 (qd, *J* = 9.4, 4.9 Hz, 2H), 1.60 – 1.46 (m, 2H), 1.37 –1.19 (m, 13H), 0.94 (s, 3H), 0.87 (t, *J* = 6.8 Hz, 4H).

^{13}C NMR (150 MHz, CD_2Cl_2) δ 165.74, 151.15 (d, *J* = 7.2 Hz), 141.86, 139.87, 130.20, 130.14, 130.08, 129.88, 128.60, 128.50, 128.42, 128.36, 128.30, 128.25, 128.18, 125.63, 120.48 (d, *J* = 4.9 Hz), 75.84 (d, *J* = 7.2 Hz), 62.32 (d, *J* = 8.9 Hz), 38.57, 32.31, 30.54, 30.02, 29.97, 29.75, 24.07, 23.70, 23.11, 14.29.

^{31}P NMR (160 MHz, CD_2Cl_2) δ -12.01.

HRMS (ESI-TOF) m/z calcd. for $C_{37}H_{45}NO_4P^+$ ($[M+H]^+$) 598.3081, found: 598.3080.

2-((Diphenylmethylene)amino)-2,6-dimethylheptyl diphenyl phosphate (38)

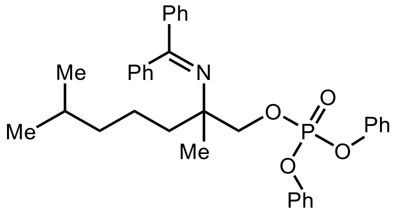

Prepared following the general procedure D from **S38** (120 mg, 0.2 mmol, 1.0 equiv.). Purification by flash chromatography (PE/EA = 20/1 to 10/1) provided the title compound (34.5 mg, 31% yield) as a viscous liquid.

^{1}H NMR (400 MHz, CD_2Cl_2) δ 7.56 – 7.49 (m, 2H), 7.41 – 7.27 (m, 10H), 7.23 – 7.15 (m, 8H), 4.26 (qd, *J* = 9.4, 4.6 Hz, 2H), 1.50 – 1.40 (m, 2H), 1.37 – 1.24 (m, 4H), 1.00 (d, *J* = 5.9 Hz, 1H), 0.94 (s, 3H), 0.82 (d, *J* = 6.6 Hz, 6H).

^{13}C NMR (100 MHz, CD_2Cl_2) δ 165.69, 150.97 (d, *J* = 7.2 Hz), 141.68, 139.70, 130.08, 128.49, 128.42, 128.23, 128.11, 125.58, 120.38 (d, *J* = 4.9 Hz), 75.70 (d, *J* = 6.8 Hz), 62.19 (d, *J* = 9.1 Hz), 39.64, 38.42, 28.22, 23.67, 22.80, 22.64, 21.73.

^{31}P NMR (160 MHz, CD_2Cl_2) δ -11.96.

HRMS (ESI-TOF) m/z calcd. for $C_{34}H_{39}NO_4P^+$ ($[M+H]^+$) 556.2611, found: 556.2612.

6-Bromo-2-((diphenylmethylene)amino)-2-methylhexyl diphenyl phosphate (39)

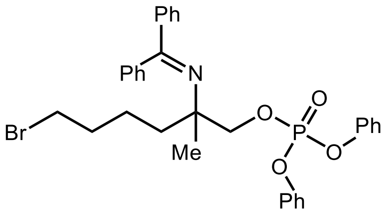

Prepared following the general procedure D from **S39** (133 mg, 0.2 mmol, 1.0 equiv.). Purification by flash chromatography (PE/EA = 20/1 to 10/1) provided the title compound (52.2 mg, 42% yield) as a viscous liquid.

^{1}H NMR (400 MHz, $CDCl_3$) δ 7.53 (dd, J = 8.4, 1.3 Hz, 2H), 7.42 – 7.38 (m, 3H), 7.35 – 7.27 (m, 7H), 7.22 – 7.15 (m, 8H), 4.24 (qd, J = 11.4, 11.6 Hz, 2H), 3.47 – 3.44 (m, 2H), 1.64 – 1.37 (m, 8H), 0.96 (s, 3H).

^{13}C NMR (100 MHz, $CDCl_3$) δ 165.73, 150.67 (d, J = 7.3 Hz), 141.28, 139.31, 129.78, 129.74, 128.19, 127.96, 127.94, 127.92, 127.78, 125.26, 120.04 (d, J = 4.8 Hz), 74.97 (d, J = 7.0 Hz), 61.69 (d, J = 9.1 Hz), 44.96, 37.37, 33.03, 23.30, 21.12.

^{31}P NMR (160 MHz, $CDCl_3$) δ -11.47.

HRMS (ESI-TOF) m/z calcd. for $C_{32}H_{34}BrNO_4P^+$ ($[M+H]^+$) 606.1404, found: 606.1401.

2-((Diphenylmethylene)amino)-2-pentylheptyl diphenyl phosphate (40)

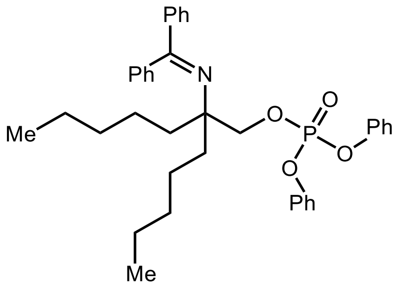

Prepared following the general procedure D from **S40** (128 mg, 0.2 mmol, 1.0 equiv.). Purification by flash chromatography (PE/EA = 20/1 to 10/1) provided the title compound (57.4 mg, 48% yield) as a viscous liquid.

^{1}H NMR (400 MHz, CD_2Cl_2) δ 7.49 (d, *J* = 7.5 Hz, 2H), 7.40 – 7.27 (m, 9H), 7.22 – 7.14 (m, 9H), 4.27 (d, *J* = 4.1 Hz, 2H), 1.41 (d, *J* = 12.2 Hz, 2H), 1.26 – 1.17 (m, 12H), 1.05 (d, *J* = 12.3 Hz, 4H), 0.82 (t, *J* = 7.3 Hz, 6H).

^{13}C NMR (100 MHz, CD_2Cl_2) δ 165.06, 150.98 (d, *J* = 7.2 Hz), 141.58, 139.88, 130.08, 128.57, 128.45, 128.29, 128.10, 127.58, 125.58, 120.32 (d, *J* = 4.9 Hz), 71.03 (d, *J* = 7.3 Hz), 64.22 (d, *J* = 9.8 Hz), 36.59, 32.60, 23.25, 22.98, 14.26.

^{31}P NMR (160 MHz, CD_2Cl_2) δ -12.12.

HRMS (ESI-TOF) m/z calcd. for $C_{37}H_{45}NO_4P^+$ ($[M+H]^+$) 598.3081, found: 598.3080.

2-Cyclohexyl-2-((diphenylmethylene)amino)propyl diphenyl phosphate (41)

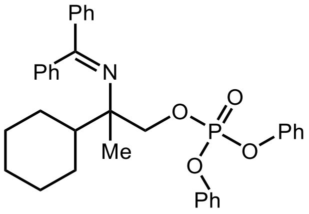

Prepared following the general procedure D from **S41** (120 mg, 0.2 mmol, 1.0 equiv.). Purification by flash chromatography (PE/EA = 20/1 to 10/1) provided the title compound (47.6 mg, 43% yield) as a viscous liquid.

^{1}H NMR (400 MHz, CD_2Cl_2) δ 7.59 – 7.51 (m, 2H), 7.44 – 7.20 (m, 10H), 7.18 – 7.05 (m, 8H), 4.32 – 4.20 (m, 2H), 1.74 (d, *J* = 8.1 Hz, 4H), 1.64 (d, *J* = 8.8 Hz, 1H), 1.33 – 1.07 (m, 6H), 0.81 (s, 3H).

^{13}C NMR (100 MHz, CD_2Cl_2) δ 165.06, 151.02 (d, *J* = 7.3 Hz), 142.06, 140.07, 130.09, 129.94, 128.60, 128.48, 128.29, 128.15, 125.58, 120.41 (d, *J* = 4.9 Hz), 120.39, 120.37, 75.37 (d, *J* = 7.5 Hz), 65.10 (d, *J* = 8.3 Hz), 46.91, 27.82, 27.78, 27.31, 27.23, 26.95, 20.82.

^{31}P NMR (160 MHz, CD_2Cl_2) δ -11.87.

HRMS (ESI-TOF) m/z calcd. for $C_{34}H_{37}NO_4P^+$ ($[M+H]^+$) 554.2455, found: 554.2452.

6) Reaction By-products and Limitations

By-products Analysis

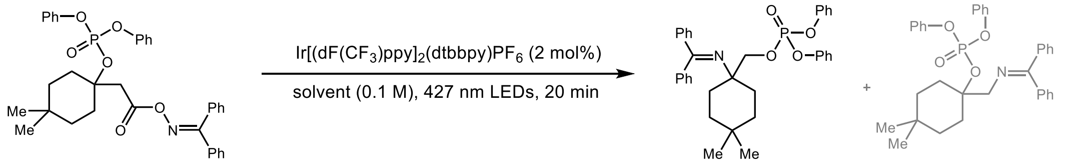

0.05 mmol 33 bp33

entry	solvent	temperature	NMR yield[a] (**33**)	NMR yield[a] (**33**)
1	MeCN	R.T.	46% (43%)[b]	9% (5%)[b]
2	DCE	R.T.	27%	14%
3	EtOAc	R.T.	13%	32%
4	acetone	R.T.	27%	17%
5	MeCN	0 °C	36%	16%
6	EtOAc	0 °C	10%	45%

[a]NMR yield using 3,4,5-trichloropyridine as the internal standard; [b]0.2 mmol scale, 1 h, isolated yield is shown in parentheses.

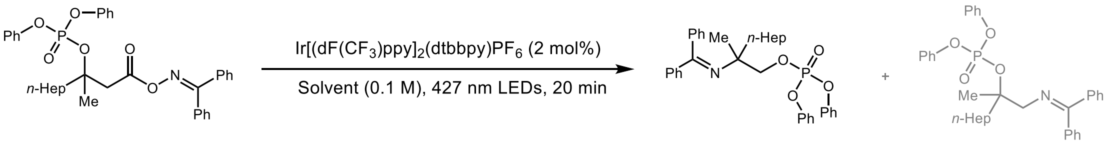

0.05 mmol 36 bp36

entry	solvent	temperature	NMR yield[a] (**36**)	NMR yield[a] (**36**)
1	MeCN	R.T.	48% (44%)[b]	3% (< 3%)[b]
2	DCE	R.T.	32%	4%
3	EtOAc	R.T.	27%	19%
4	acetone	R.T.	36%	6%
5	MeCN	0 °C	28%	10%
6	EtOAc	0 °C	15%	30%

[a]NMR yield using 3,4,5-trichloropyridine as the internal standard; [b]0.2 mmol scale, 1 h, isolated yield is shown in parentheses.

Figure S6. The yields of by-products with solvents and temperature

1-(((Diphenylmethylene)amino)methyl)-4,4-dimethylcyclohexyl diphenyl phosphate (bp33)

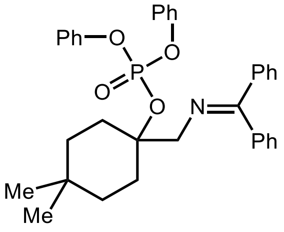

^{1}H NMR (400 MHz, CDCl$_3$) δ 7.64 – 7.59 (m, 2H), 7.42 – 7.30 (m, 6H), 7.22 (d, J = 6.9 Hz, 4H), 7.17 – 7.10 (m, 8H), 3.69 (s, 2H), 2.10 – 2.04 (m, 2H), 1.97 – 1.89 (m,

2H), 1.44 – 1.38 (m, 2H), 1.16 (s, 2H), 0.88 (s, 3H), 0.84 (s, 3H).

^{13}C NMR (100 MHz, CDCl$_3$) δ 169.14, 151.05 (d, *J* = 7.6 Hz), 139.68, 136.80, 130.16, 129.83, 129.65, 128.75, 128.57, 128.14, 127.75, 124.98, 120.37 (d, *J* = 5.0 Hz), 90.27 (d, *J* = 8.3 Hz), 60.80, 34.49, 30.45, 30.40, 29.84, 29.37.

^{31}P NMR (160 MHz, CDCl$_3$) δ -17.24.

HRMS (ESI-TOF) m/z calcd. for $C_{34}H_{37}BrNO_4P^+$ ($[M+H]^+$) 554.2449, found: 554.2452.

1-((Diphenylmethylene)amino)-2-methylnonan-2-yl diphenyl phosphate (bp36)

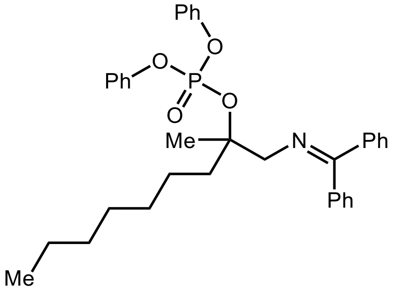

^{1}H NMR (400 MHz, CDCl$_3$) δ 7.64 – 7.60 (m, 2H), 7.45 – 7.32 (m, 6H), 7.23 – 7.08 (m, 12H), 3.59 (s, 2H), 1.91 (d, *J* = 7.6 Hz, 2H), 1.66 (s, 3H), 1.26 – 1.17 (m, 10H), 0.86 (d, *J* = 6.8 Hz, 3H).

^{13}C NMR (100 MHz, CDCl$_3$) δ 169.18, 151.03 (d, *J* = 7.2 Hz), 139.72, 136.75, 130.22, 129.84, 129.65 (d, *J* = 2.6 Hz), 128.73, 128.62, 128.15, 127.78, 124.98 (d, *J* = 4.0 Hz), 120.33 (d, *J* = 8.9 Hz), 90.63 (d, *J* = 8.2 Hz), 60.95 (d, *J* = 5.5 Hz), 38.99 (d, *J* = 4.6 Hz), 31.90, 29.93, 29.32, 24.29, 23.64, 22.80, 14.24.

^{31}P NMR (160 MHz, CDCl$_3$) δ -17.00.

HRMS (ESI-TOF) m/z calcd. for $C_{35}H_{41}NO_4P^+$ ($[M+H]^+$) 570.2768, found: 570.2762.

Reaction Limitations

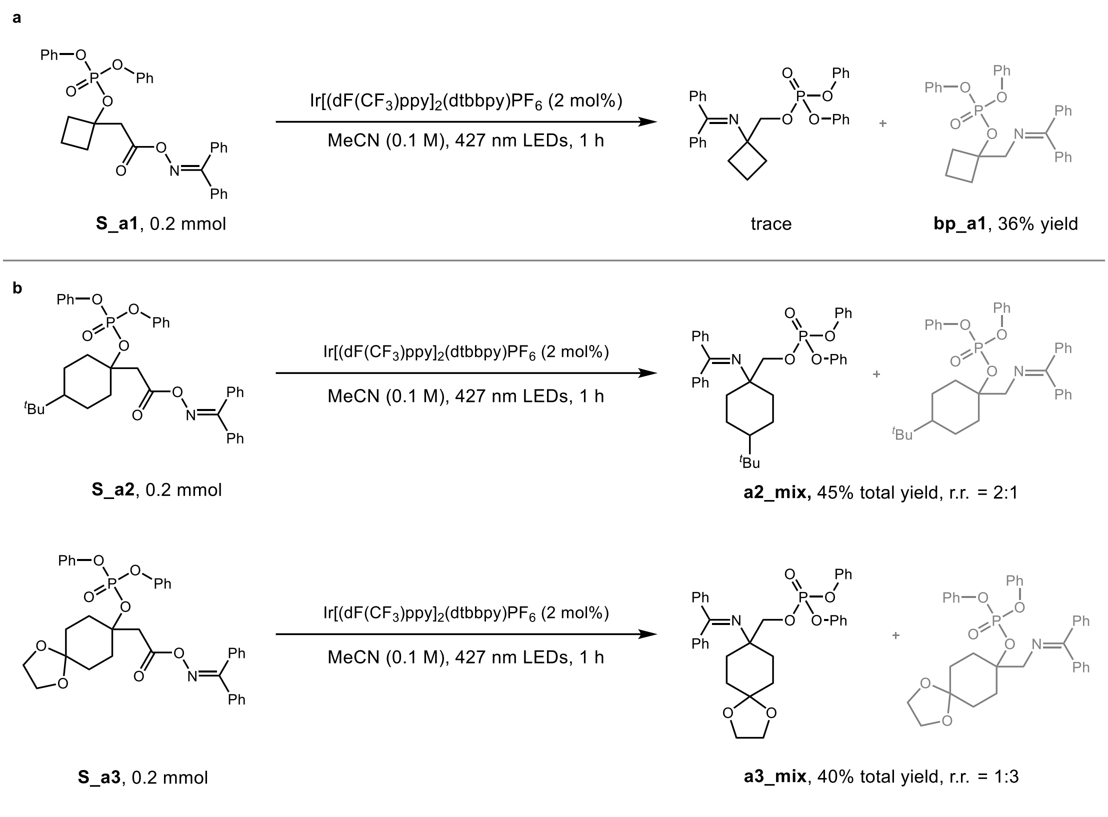

Figure S7. The additional examples of unactivated substrates

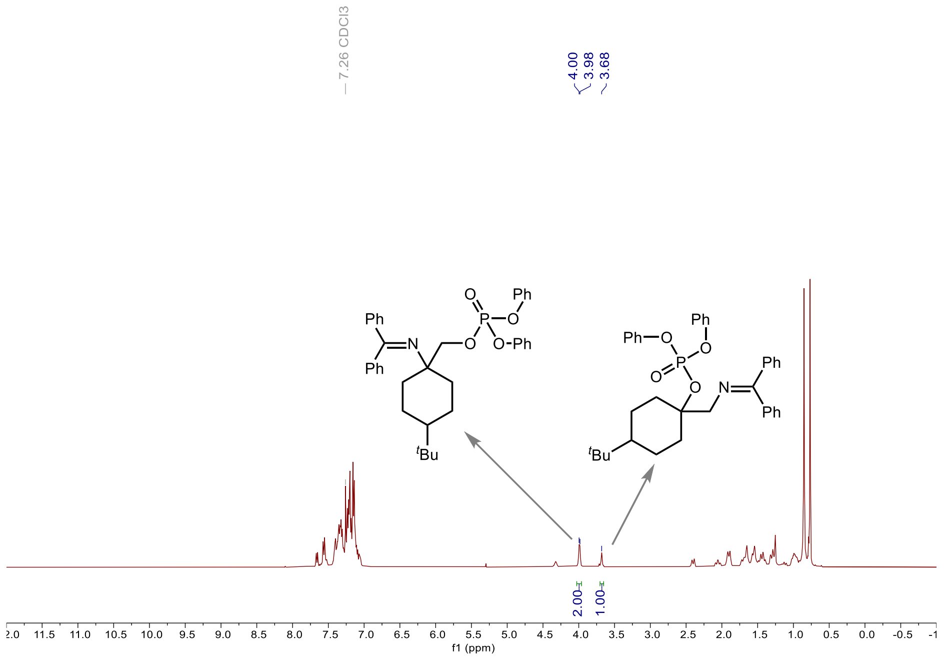

Figure S8. The ratio of migration and homocoupling product in **a2_mix**

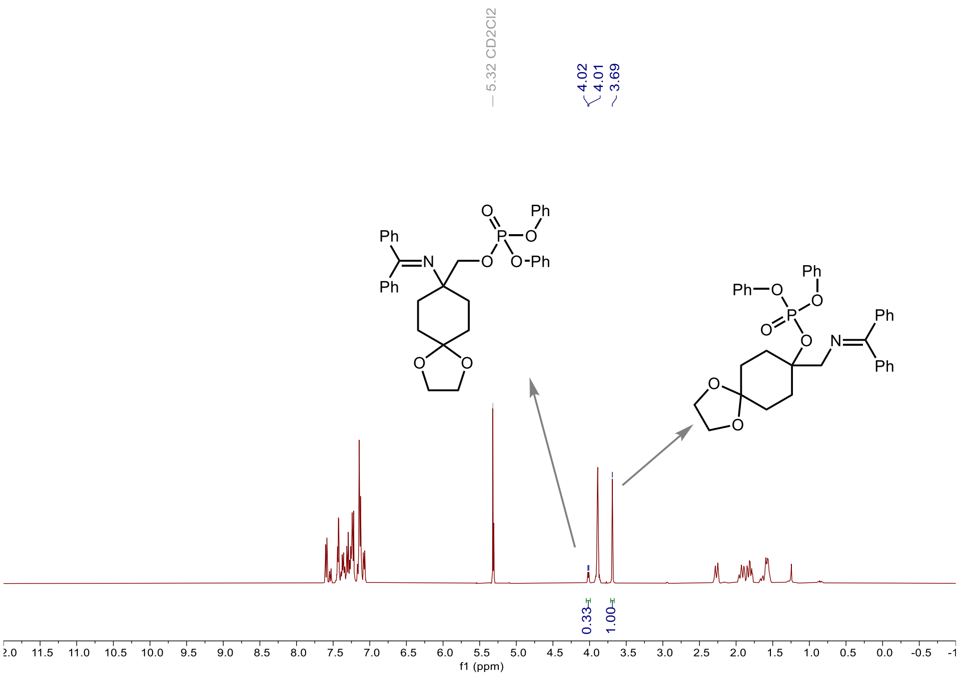

Figure S9. The ratio of migration and homocoupling product in **a3_mix**

An increased electron density at the phosphatoxy substitutional α-position may enhance the α–β electron density difference, thereby accelerating phosphatoxy migration. However, for small ring compounds, for example, four-membered cyclic substrate, no desired dFGT product was detected and only decarboxylative homocoupling byproduct was isolated, which may attribute to the severe ring strain of this scaffold that completely suppresses the target rearrangement pathway (Figure S7). Six-membered cyclohexyl substrates bearing tert-butyl or ketal protecting groups underwent the transformation under standard conditions yet afforded inseparable mixtures of target dFGT adducts and decarboxylative homocoupling dimers (Figure S7, S8 and S9).

1-(2-(((Diphenylmethylene)amino)oxy)-2-oxoethyl)cyclobutyl diphenyl phosphate (S_a1)

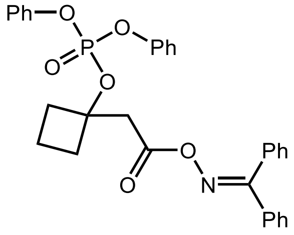

Prepared following the general procedure A outlined above starting from undecan-2-

one and benzyl 2-bromoacetate on 10 mmol scale to give **S_a1** (1.4 g, 26% total yield) as a colorless oil.

^{1}H NMR (400 MHz, CD_2Cl_2) δ 7.63 – 7.60 (m, 2H), 7.49 – 7.32 (m, 12H), 7.26 – 7.14 (m, 8H), 2.98 (s, 2H), 2.74 – 2.65 (m, 2H), 2.33 – 2.27 (m, 2H), 1.89 –1.80 (m, 1H), 1.62 – 1.51 (m, 1H).

^{13}C NMR (100 MHz, CD_2Cl_2) δ 166.85, 164.98, 150.59 (d, *J* = 7.5 Hz), 134.69, 132.57, 131.01, 129.78, 129.60, 129.07, 128.72, 128.44, 128.29, 125.29, 120.31 (d, *J* = 4.9 Hz), 82.95 (d, *J* = 7.8 Hz), 41.63 (d, *J* = 4.6 Hz), 34.60, 34.55, 12.73.

^{31}P NMR (160 MHz, CD_2Cl_2) δ -16.60.

HRMS (ESI-TOF) m/z calcd. for $C_{31}H_{29}NO_6P^+$ ($[M+H]^+$) 542.1727, found: 542.1730.

4-(*tert*-butyl)-1-(2-(((diphenylmethylene)amino)oxy)-2-oxoethyl)cyclohexyl diphenyl phosphate (S_a2)

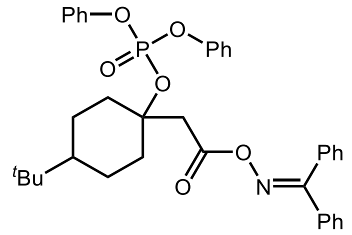

Prepared following the general procedure A outlined above starting from 4-(*tert*-butyl)cyclohexan-1-one and benzyl 2-bromoacetate on 10 mmol scale to give **S_a2** (1.5 g, 24% total yield) as a white soild.

^{1}H NMR (400 MHz, CD_2Cl_2) δ 7.58 (d, *J* = 7.5 Hz, 2H), 7.51 – 7.39 (m, 6H), 7.36 – 7.29 (m, 4H), 7.28 – 7.13 (m, 8H), 2.83 (s, 2H), 2.17 (d, *J* = 12.2 Hz, 2H), 2.00 (t, *J* = 11.5 Hz, 2H), 1.63 (d, *J* = 12.8 Hz, 2H), 1.06 (t, *J* = 12.3 Hz, 1H), 0.97 – 0.88 (m, 2H), 0.80 (s, 9H).

^{13}C NMR (100 MHz, CD_2Cl_2) δ 166.82, 164.91, 151.05 (d, J = 7.0 Hz), 135.06, 133.04, 131.29, 130.01, 129.80, 129.13, 128.85, 128.79, 128.66, 125.44, 120.63 (d, J = 4.9 Hz), 88.15 (d, J = 7.6 Hz), 46.90, 39.79, 39.73, 36.82, 36.79, 32.31, 27.52, 24.87.

^{31}P NMR (160 MHz, CD_2Cl_2) -17.11.

HRMS (ESI-TOF) m/z calcd. for $C_{37}H_{41}NO_6P^+$ ($[M+H]^+$) 626.2666, found: 626.2661.

8-(2-(((diphenylmethylene)amino)oxy)-2-oxoethyl)-1,4-dioxaspiro[4.5]decan-8-yl diphenyl phosphate (Sa_3)

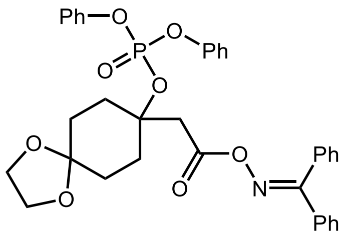

Prepared following the general procedure A outlined above starting from 1,4-dioxaspiro[4.5]decan-8-one and benzyl 2-bromoacetate on 10 mmol scale to give **Sa_3** (1.1 g, 18% total yield) as a colorless oil.

^{1}H NMR (400 MHz, CD_2Cl_2) δ 7.57 – 7.51 (m, 2H), 7.49 – 7.29 (m, 10H), 7.28 – 7.14 (m, 8H), 3.88 (s, 4H), 2.99 (s, 2H), 2.23 (d, J = 13.5 Hz, 2H), 1.94 – 1.69 (m, 4H), 1.52 (d, J = 13.0 Hz, 2H).

^{13}C NMR (100 MHz, CD_2Cl_2) δ 166.72, 165.27, 150.99 (d, J = 7.4 Hz), 135.00, 132.80, 131.32, 130.08, 129.90, 129.18, 129.02, 128.77, 128.57, 125.58, 120.45 (d, J = 4.9 Hz), 107.61, 85.66, 64.66 (d, J = 12.8 Hz), 43.02, 33.83, 33.77, 30.59.

^{31}P NMR (160 MHz, CD_2Cl_2) δ -16.67.

HRMS (ESI-TOF) m/z calcd. for $C_{35}H_{35}NO_8P^+$ ($[M+H]^+$) 628.2095, found: 628.2093.

(((Dphenylmethylene)amino)methyl)cyclobutyl diphenyl phosphate (bp33)

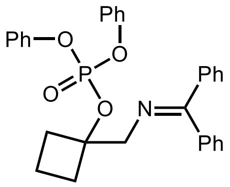

^{1}H NMR (400 MHz, CDCl$_3$) δ 7.68 – 7.62 (m, 2H), 7.42 – 7.39 (m, 3H), 7.33 (d, *J* = 7.7 Hz, 2H), 7.24 – 7.15 (m, 9H), 7.11 (d, *J* = 6.9 Hz, 2H), 7.04 (dd, *J* = 6.5, 3.0 Hz, 2H), 3.68 (s, 2H), 2.76 – 2.68 (m, 2H), 2.43 – 2.35 (m, 2H), 1.87 (dd, *J* = 7.5, 3.1 Hz, 1H), 1.59 (d, *J* = 9.1 Hz, 1H).

^{13}C NMR (100 MHz, CDCl$_3$) δ 169.98, 150.86 (d, *J* = 7.6 Hz), 139.68, 136.79, 130.24, 129.82, 129.70, 128.73, 128.69, 128.54, 128.42, 128.14, 127.78, 125.12, δ 120.41 (d, *J* = 5.0 Hz), 85.71 (d, *J* = 8.3 Hz), 59.48 (d, *J* = 4.9 Hz) 33.16, 33.11, 13.02.

^{31}P NMR (160 MHz, CDCl$_3$) δ -16.68.

HRMS (ESI-TOF) m/z calcd. for $C_{30}H_{29}NO_4P^+$ ($[M+H]^+$) 498.1829, found: 498.1824.

7)Mechanism Experiment

Radical Inhibition Experiment

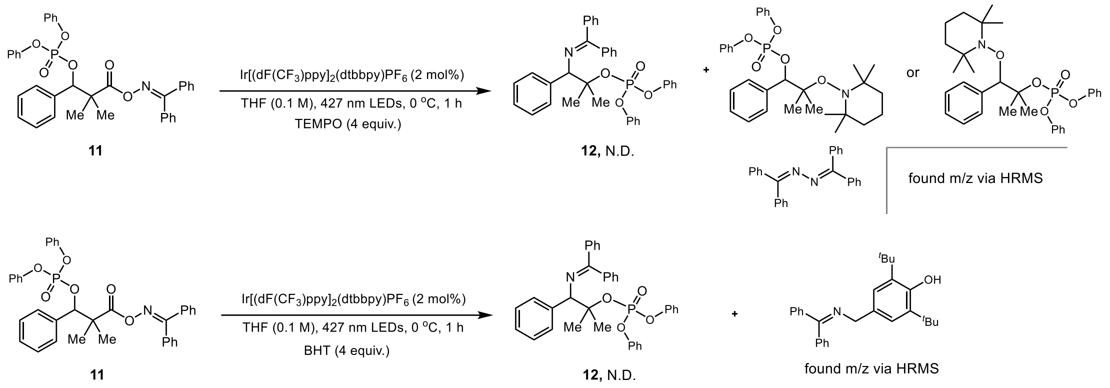

An oven-dried vial (8 mL) containing a stirring bar was charged with **11** (61 mg, 0.1 mmol, 1.0 equiv.), Ir[(dF(CF_3)ppy]$_2$(dtbbpy)PF_6 (2 mg, 0.002 mmol, 2 mol%), TEMPO (62 mg, 0.4 mmol, 4.0 equiv.) or BTH (88 mg, 0.4 mmol, 4.0 equiv.), then THF (1 mL) was added to the mixture in the glovebox. The reaction was stirred and irradiated using Shanshi photoreactor to maintain at 0 °C for 1 h. The reactions were not found the product **12**, and radical capture products were detected by HMRS.

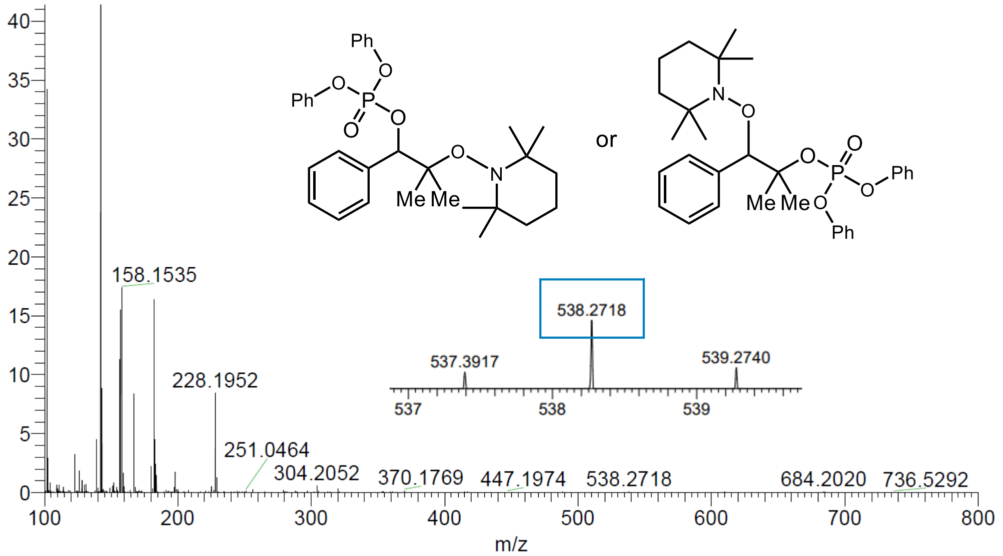

Figure S10. The HRMS of alkyl radical capture product

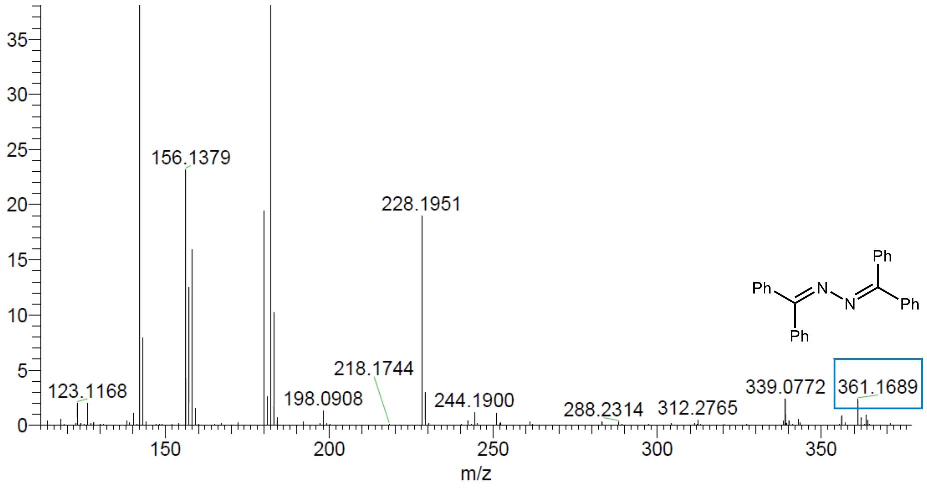

Figure S11. The HRMS of diphenylimine radical dimerization product

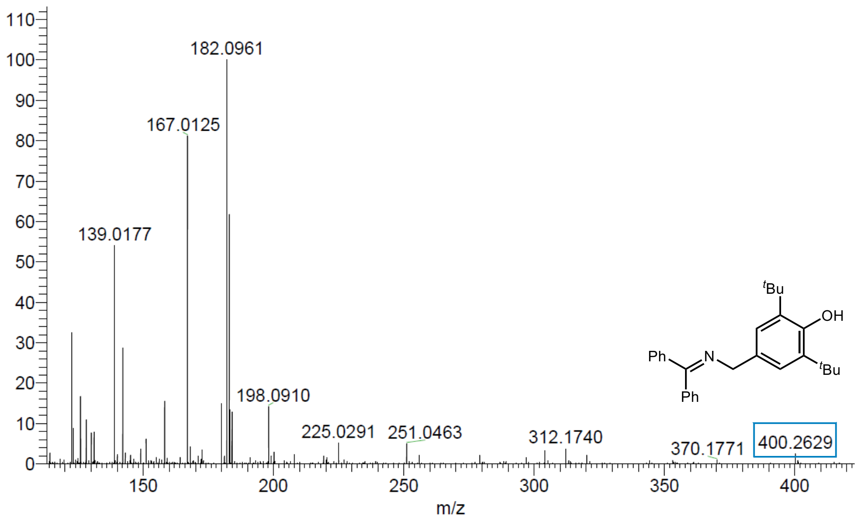

Figure S12. The HRMS of diphenylimine radical capture product

Stern-Volmer Luminescence Quenching Experiment

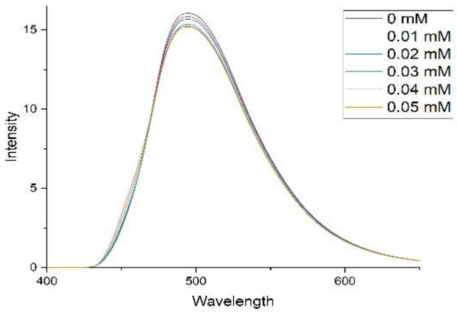

Figure S13 Fluorescence quenching experiments of **11** to **PC**

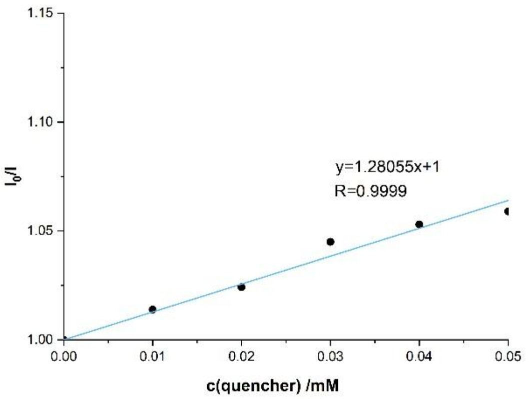

Figure S14. Stern-Volmer quenching plot of oxime ester substrate

EnT Mechanism Exploration

To differentiate between the two possible mechanistic manifolds (EnT vs. SET), we deliberately selected a series of photocatalysts that exhibit significant variations in both their triplet excited-state energies E_T and their redox potentials ($E^*_{1/2}$, spanning a window of >1.0 V). A remarkably strong positive correlation was observed between the reaction efficiency and the E_T values of the photocatalysts—catalysts with higher triplet energies consistently afforded relatively superior product yields. In stark contrast, no discernible correlation was found between the reaction outcome and the excited-state reduction potentials ($E^*_{1/2}$) of the catalysts, despite the wide potential range covered by the selected photosensitizers. This stark dichotomy strongly suggests that the critical activation step is governed by an EnT pathway, rather than a thermodynamically driven SET process (Figure S15).

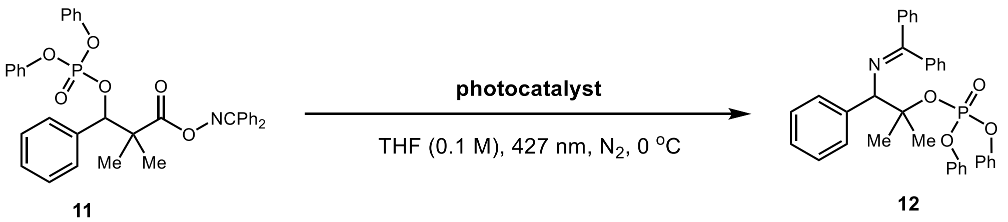

photocatalyst	E_T (kcal mol^{-1})	$E_{1/2}$ (M$^-$/M*) (V)	$E_{1/2}$ (M*/M$^+$) (V)	yield[a]
[Ru(bpy)$_3$](PF$_6$)$_2$ (2 mol%)	49.0	+0.77	-0.81	N.D.
Ir(ppy)$_2$(dtbbpy)PF$_6$ (2 mol%)	49.2	+0.66	-0.96	N.D.
fac-Ir(ppy)$_3$ (2 mol%)	58.1	+0.31	-1.73	trace
4CzIPN (5 mol%)	58.3	+1.35	-1.04	15%
Ir[(dF(CF$_3$)ppy]$_2$(dtbbpy)PF$_6$ (2 mol%)	61.8	+1.21	-0.89	73%
thioxanthone (5 mol%, 390 nm)	65.5	+1.18	-1.11	64%

[a]NMR yield using 3,4,5-trichloropyridine as the internal standard.

Figure S15. The positive correlation between the yields and the E_T values of photocatalysts

Crossover Experiment

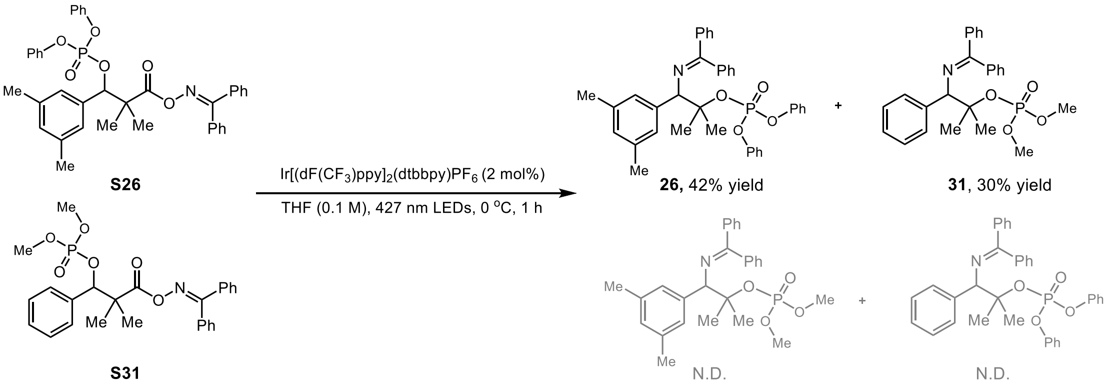

An oven-dried vial (8 mL) containing a stirring bar was charged with **S26** (64 mg, 0.1 mmol, 1.0 equiv.), **S26** (48 mg, 0.1 mmol, 1.0 equiv.), Ir[(dF(CF$_3$)ppy]$_2$(dtbbpy)PF$_6$ (4 mg, 0.004 mmol, 2 mol%), then THF (2 mL) was added to the mixture in the glovebox. The reaction was stirred and irradiated using Shanshi photoreactor to maintain at 0 °C for 1 h. The reaction was not found the product **12**, while **11** was recovered in 90% yield. The reaction afforded products **26** and **31** in 42% and 30% yield, respectively, however, no crossover products were detected, indicating that the 1,2-phosphatoxy migration proceeds via an intramolecular and concerted pathway.

Synthesis of 11-^{18}O

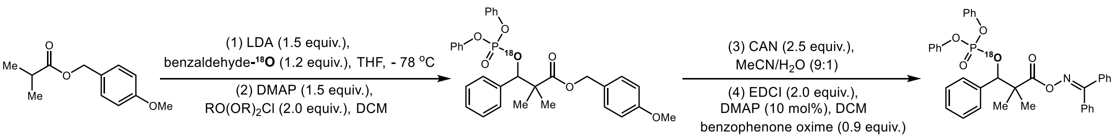

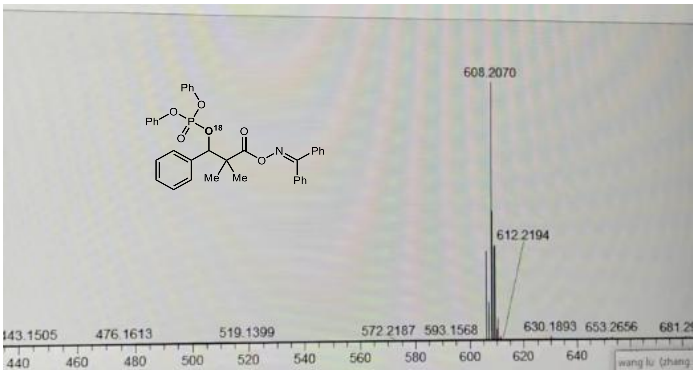

Figure S16. The HRMS of **11-^{18}O**

^{18}O-labeling Experiment

An oven-dried vial (8 mL) containing a stirring bar was charged with **11-^{18}O** (61 mg, 0.1 mmol, 1.0 equiv.) and photocatalyst, then THF (2 mL) was added to the mixture in the glovebox. The reaction was stirred and irradiated using Shanshi photoreactor to maintain at 0 °C or using Kessil blue LED lamps at R.T. for 1 h. The reaction was not found the product **12**, while **11** was recovered in 90% yield. After the starting material was consumed in 1 h, then the reaction was added AlH_4Li (19 mg, 0.5 mmol, 5.0 equiv.) to stirring another 6 h. After complete reaction, a mixture of **42** and **42-^{18}**O was obtained through HRMS.

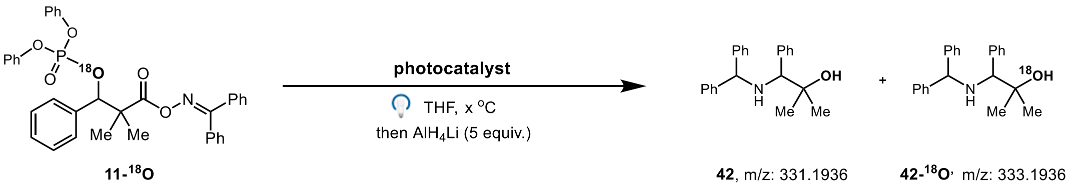

photocatalyst	temperature	ratio (42:42-^{18}O)
Ir[(dF(CF$_3$)ppy]$_2$(dtbbpy)PF$_6$ (2 mol%)	0 °C	4:1
Ir[(dF(CF$_3$)ppy]$_2$(dtbbpy)PF$_6$ (2 mol%)	R.T.	5:1
4CzIPN (5 mol%)	0 °C	1.6:1
thioxanthone (5 mol%, 390 nm)	0 °C	1.1:1

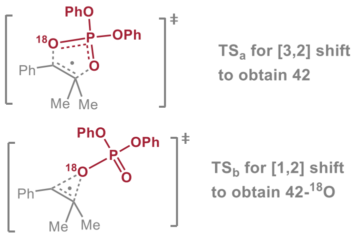

Figure S17. O^{18}-labeling Experiment in different reaction condition

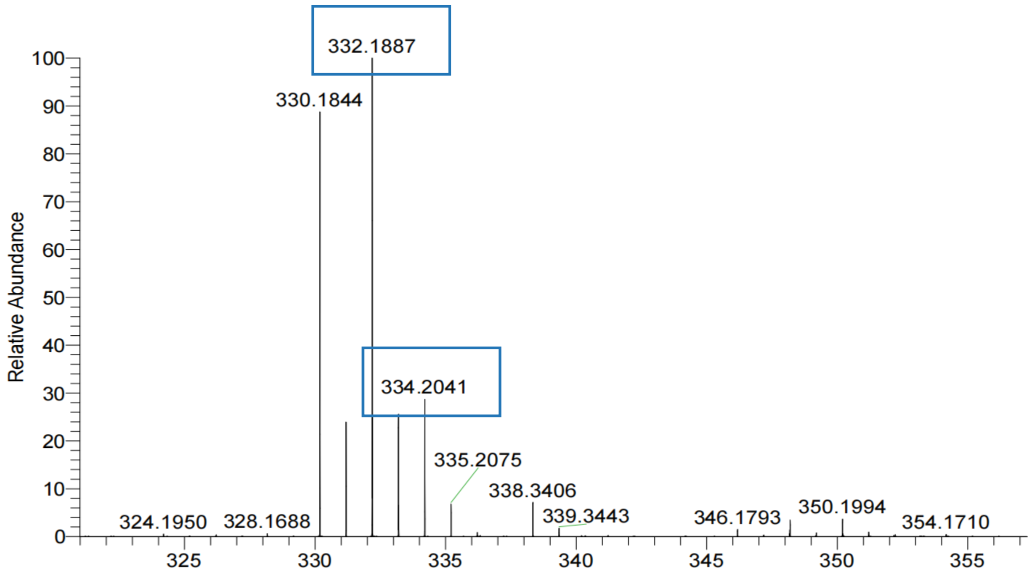

Figure S18 The HRMS of **42** and **42-**^{18}O under standard conditions

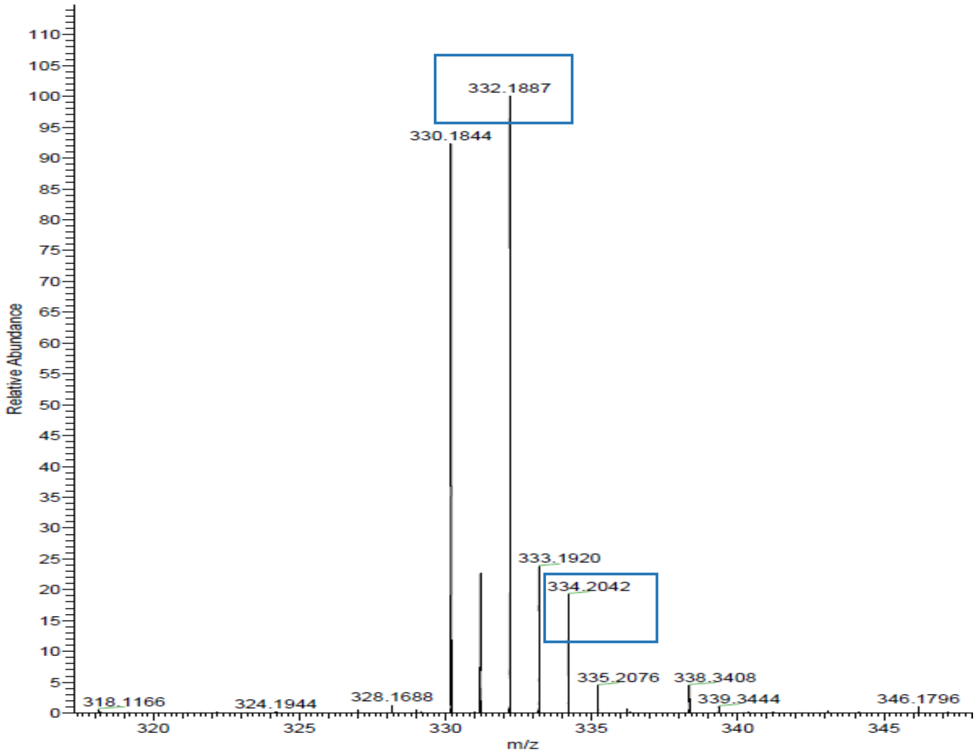

Figure S19. The HRMS of 42 and 42-^{18}O under conditions of using $Ir[(dF(CF_3)ppy]_2(dtbbpy)PF_6$ at R.T.

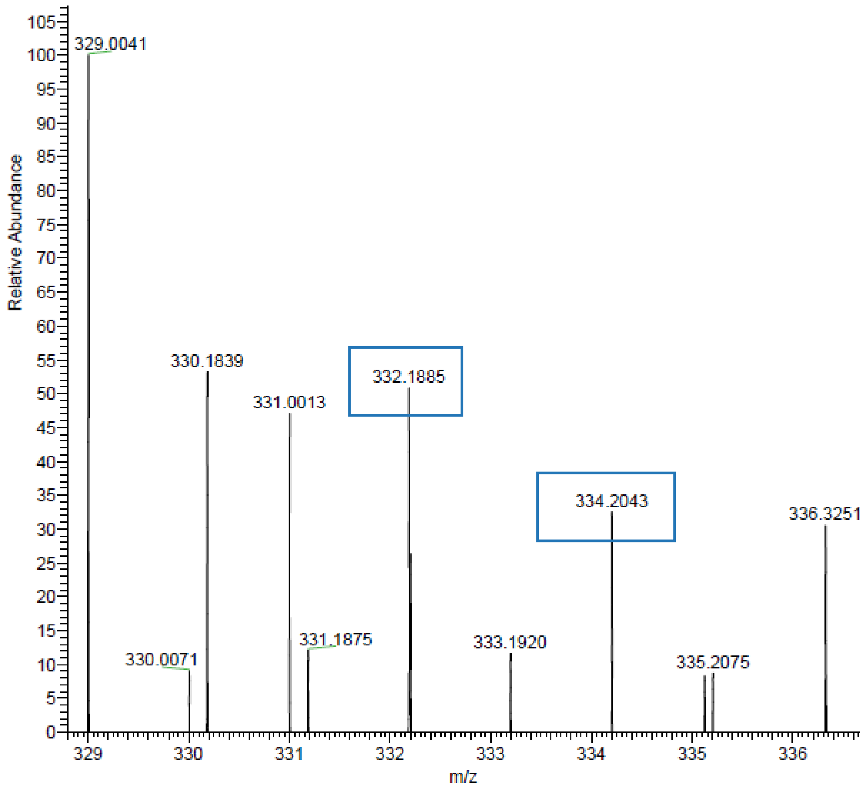

Figure S20. The HRMS (ESI-TOF) of 42 and 42-^{18}O under conditions of using 4CzIPN at 0 °C.

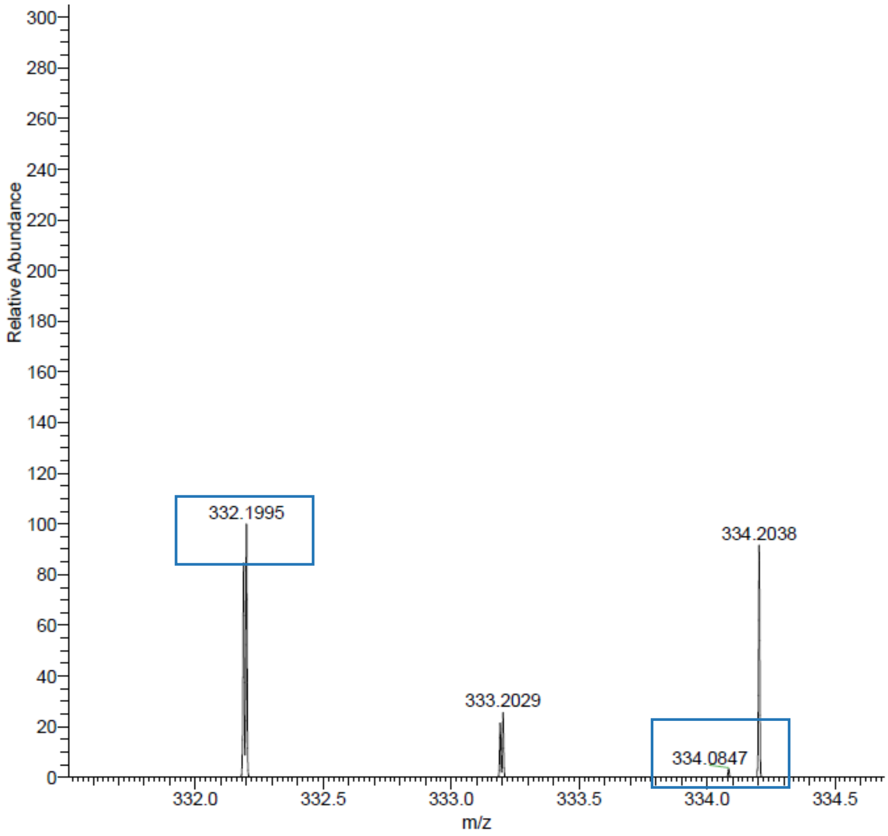

Figure S21. The HRMS (ESI-TOF) of 42 and 42-^{18}O under conditions of using thioxanthone at 0 °C.

8) DFT Calculation

Computation Details

The geometries were optimized in vacuum at B3LYP[1]-D3[2]/def2-SVP[3] level. Harmonic vibrational frequency analyses were performed at the same level to verify the nature of stationary points (no imaginary frequency for minima and only one imaginary frequency for transition states). The energies were further improved by B3LYP-D3/def2-TZVPP single-point calculations in vacuum or with THF SMD solvent model. Harmonic vibration frequencies at B3LYP-D3/def2-SVP level were used to correct the singlet-point energies to free energies at 298.15 K. All DFT calculations were carried out by using Gaussian 16 program[4]. Selected computed structures and orbitals were illustrated using the CYLview[5].

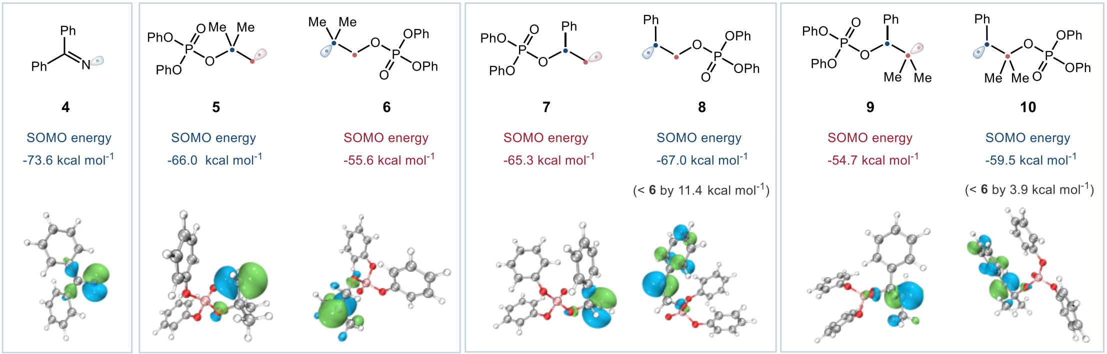

Figure S22. SOMO orbitals and energies of the imine radical and intermediates in these reactions

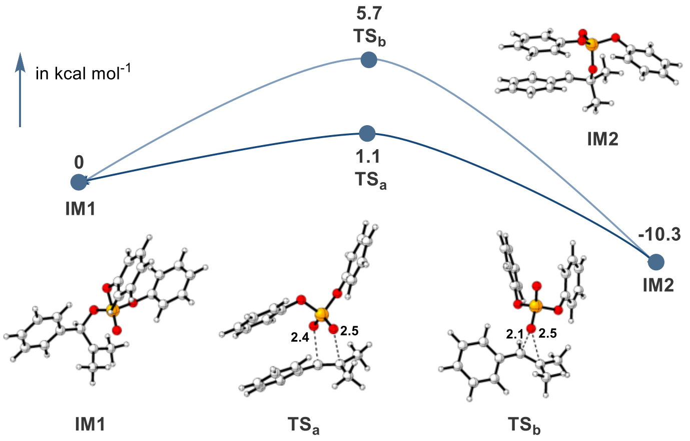

Figure S23. Free energy profiles of 1,2-phosphatoxy migration calculated at the B3LYP(D3)/def2-TZVPP/SMD(THF)//B3LYP(D3)/def2-SVP level of theory

Energies and Cartesian coordinates at 298.15 K and 1 atm for all structures (Energies are given in Hartree and coordinates in angstroms)

4

B3LYP-D3/def2-SVP SCF energy in vacuum: -555.597163

B3LYP-D3/def2-TZVPP SCF energy in vacuum: -556.3551004

B3LYP-D3/def2-TZVPP free energy in vacuum: -556.2026014

C	-3.65046200	0.27279500	-0.55334300
C	-2.42507600	0.93638900	-0.61366900
C	-1.29916800	0.40323900	0.03478700
C	-1.42112000	-0.79853700	0.75012200
C	-2.65075600	-1.45687700	0.81227500
C	-3.76643800	-0.92489100	0.15936800
H	-4.51940600	0.69034800	-1.06740700
H	-2.32299600	1.87068600	-1.16847300
H	-0.55223600	-1.21279800	1.26464200
H	-2.73792200	-2.38957200	1.37459400
H	-4.72643100	-1.44466000	0.20455100
C	-0.00000700	1.14936900	-0.00000400
C	1.29916300	0.40324500	-0.03477100
C	2.42503400	0.93635400	0.61377700
C	1.42115300	-0.79847700	-0.75018900
C	3.65042600	0.27276500	0.55346600
H	2.32292700	1.87061500	1.16863600
C	2.65079400	-1.45680800	-0.81233200
H	0.55229400	-1.21270100	-1.26478300
C	3.76644200	-0.92486500	-0.15933000
H	4.51934300	0.69028500	1.06760300
H	2.73799200	-2.38946100	-1.37471800
H	4.72643900	-1.44462600	-0.20450700
N	0.00001100	2.41281100	-0.00015400

5

B3LYP-D3/def2-SVP SCF energy in vacuum: -1261.876070

B3LYP-D3/def2-TZVPP SCF energy in vacuum: -1263.325053

B3LYP-D3/def2-TZVPP free energy in vacuum: -1263.069302

C	1.97386400	2.28782300	-0.03402900
C	2.81071100	1.29629900	0.70214600
O	0.78368500	1.59855000	-0.63064100
P	-0.17633800	0.62483200	0.18671700
O	-0.10095100	0.66938500	1.66892100
O	0.06173500	-0.84982600	-0.46735800
O	-1.58717700	0.99115400	-0.50235300
C	1.24330300	-1.54233000	-0.25217500
C	1.61090400	-1.94900800	1.03321500
C	2.04941900	-1.82006400	-1.35745300
C	2.81031700	-2.64412000	1.20500600
H	0.96535700	-1.69860900	1.87523200
C	3.24415600	-2.51999500	-1.17235800
H	1.72877700	-1.47842400	-2.34287300
C	3.62911500	-2.93074500	0.10809100
H	3.10638300	-2.96316100	2.20714500
H	3.87855300	-2.74201200	-2.03376400
H	4.56490900	-3.47574300	0.25028000
C	-2.76513800	0.29350400	-0.26155400
C	-3.56275000	-0.00080700	-1.36788600
C	-3.15489300	-0.06732400	1.03108200
C	-4.77433000	-0.66815400	-1.17728400
H	-3.21990500	0.29691300	-2.35996900
C	-4.36644700	-0.74191600	1.20441900

H	-2.51120200	0.17945300	1.87603300
C	-5.17871200	-1.04341300	0.10766300
H	-5.40281400	-0.89985600	-2.04043700
H	-4.67718500	-1.02973000	2.21160600
H	-6.12506000	-1.56878100	0.25351500
H	3.44419700	0.60221300	0.14587400
H	2.65733600	1.13125900	1.76876300
C	2.69660700	2.82747600	-1.26362700
H	3.00367700	1.99835100	-1.91781800
H	2.03301400	3.49466200	-1.83292800
H	3.59239400	3.38868500	-0.95890100
C	1.45860500	3.40240600	0.87184600
H	2.30478500	3.98445800	1.26760400
H	0.80077300	4.07782000	0.30494700
H	0.89437000	2.97812000	1.71376300

6

B3LYP-D3/def2-SVP SCF energy in vacuum: -1261.879726

B3LYP-D3/def2-TZVPP SCF energy in vacuum: -1263.328383

B3LYP-D3/def2-TZVPP free energy in vacuum: -1263.071590

P	0.10387300	0.59587700	-0.61401500
O	0.11515900	0.90601400	0.96079400
O	-1.41024000	0.17311800	-0.98963000
C	-0.73572300	1.96304600	1.54920000
O	0.84420600	-0.84084100	-0.64457600
O	0.61185200	1.64323200	-1.52388200
C	-2.21387200	-0.80444300	-0.42797800
C	-0.67822600	3.26078900	0.83153000
H	-0.33677400	2.04378400	2.57137100
H	-1.76072100	1.56285700	1.59488300

C	2.18207900	-1.04587400	-0.33329100
C	-3.38730900	-1.09490400	-1.12902900
C	-1.91045200	-1.46538700	0.76668900
C	-1.73288500	3.62180100	-0.16237500
C	0.61381300	4.00753900	0.81615300
C	2.48711000	-2.18046100	0.41942000
C	3.18281600	-0.17926200	-0.78035000
C	-4.26783900	-2.05506100	-0.63002500
H	-3.58721000	-0.55986300	-2.05870200
C	-2.80270400	-2.42621300	1.25230500
H	-0.99542500	-1.23424400	1.30978000
H	-2.68109100	3.09426400	0.02453600
H	-1.92973900	4.70796600	-0.16249200
H	-1.40204800	3.35909200	-1.18690400
H	1.20844500	3.82602500	1.72584900
H	0.45424700	5.09356200	0.70833500
H	1.22305200	3.68093100	-0.04937200
C	3.82039600	-2.45233000	0.73288100
H	1.67561100	-2.83368300	0.74426300
C	4.51151100	-0.46115500	-0.45138800
H	2.91333700	0.69285600	-1.37732000
C	-3.97952500	-2.72611200	0.56262000
H	-5.18436800	-2.28089700	-1.18009900
H	-2.56750600	-2.94469600	2.18490900
C	4.83582600	-1.59274300	0.30237000
H	4.06494100	-3.34046200	1.32035200
H	5.30017300	0.21191800	-0.79593900
H	-4.66847100	-3.47895100	0.95126600
H	5.87753800	-1.80617500	0.55147300

7

B3LYP-D3/def2-SVP SCF energy in vacuum: -1414.177735

B3LYP-D3/def2-TZVPP SCF energy in vacuum: -1415.809652

B3LYP-D3/def2-TZVPP free energy in vacuum: -1415.529646

C	4.12334300	1.02863300	-0.84553600
C	3.60507000	-0.26937300	-0.87865800
C	3.06897400	-0.84613900	0.27769800
C	3.04762800	-0.10162500	1.46670100
C	3.55663500	1.19590500	1.49909500
C	4.10173700	1.76295500	0.34211500
H	4.53777800	1.46913700	-1.75536300
H	3.60420000	-0.83356400	-1.81225200
H	2.60545200	-0.53831700	2.36523800
H	3.52551900	1.76942700	2.42822100
H	4.50027600	2.77987600	0.36614400
C	2.46970400	-2.24843500	0.27949600
H	2.89404400	-2.79541200	1.13467900
C	2.65827600	-3.04237800	-0.96261100
O	1.04504800	-2.18069000	0.63453100
P	0.03768900	-1.23726300	-0.16618000
O	0.34184600	-0.93182500	-1.58374200
O	-0.11883700	0.05726200	0.80420400
O	-1.33084100	-2.05800800	0.13387000
C	-0.40822200	1.34307200	0.36135500
C	0.50151400	2.04134100	-0.43263400
C	-1.61445200	1.91728700	0.76387400
C	0.19223600	3.34683100	-0.82147600
H	1.42920900	1.56267400	-0.74083100
C	-1.90929900	3.22438100	0.37015800
H	-2.30913500	1.33049600	1.36644700

C	-1.00813300	3.94250900	-0.42211500
H	0.89964000	3.90066100	-1.44326000
H	-2.85301600	3.67918200	0.68018800
H	-1.24266800	4.96380200	-0.73066100
C	-2.55931900	-1.42037700	0.01724000
C	-3.41683000	-1.45814000	1.11754400
C	-2.91839100	-0.75703200	-1.15915900
C	-4.65520300	-0.81662100	1.03878900
H	-3.09563400	-1.98104900	2.01967100
C	-4.15597200	-0.11212400	-1.22081200
H	-2.22133700	-0.73701900	-1.99806100
C	-5.02587100	-0.13832200	-0.12704700
H	-5.33099100	-0.84232300	1.89694500
H	-4.43819900	0.41725200	-2.13366600
H	-5.99216500	0.36772100	-0.18253800
H	2.04697600	-2.81848800	-1.83796500
H	3.45644300	-3.78311100	-1.02523300

8

B3LYP-D3/def2-SVP SCF energy in vacuum: -1414.192565

B3LYP-D3/def2-TZVPP SCF energy in vacuum: -1415.828547

B3LYP-D3/def2-TZVPP free energy in vacuum: -1415.546466

C	-3.77668500	1.51955200	1.51695400
C	-3.13893000	0.29025800	1.40263300
C	-3.38110500	-0.55828500	0.28522200
C	-4.30133500	-0.10043300	-0.69891300
C	-4.93277400	1.13007200	-0.57636600
C	-4.67513000	1.95025900	0.53106000
H	-3.57080100	2.15615100	2.38090000
H	-2.42584600	-0.01587600	2.16928300

H	-4.49694000	-0.73346600	-1.56787500
H	-5.63030800	1.46113300	-1.34938900
H	-5.17030500	2.91927700	0.62437900
C	-2.71497300	-1.80117800	0.10843100
H	-2.90758000	-2.36613800	-0.80699500
C	-1.65371000	-2.31214700	1.01560000
O	0.22153800	-3.35773600	-1.05863500
P	0.51388900	-2.03569100	-0.48292300
O	-0.37788600	-1.62393000	0.80236100
O	0.32354800	-0.84125700	-1.56338000
O	2.02334500	-1.88930900	0.07415600
C	0.54282900	0.51601700	-1.35695800
C	-0.42632000	1.29022000	-0.72107500
C	1.73355100	1.08035700	-1.81562500
C	-0.17937500	2.64889600	-0.51129000
H	-1.35141700	0.82777500	-0.38674100
C	1.97168400	2.43757800	-1.59682700
H	2.46819400	0.44481200	-2.31074100
C	1.02020000	3.22417400	-0.93901300
H	-0.93512700	3.25549300	-0.00675800
H	2.91124400	2.87980700	-1.93505600
H	1.21313500	4.28511900	-0.76544300
C	2.69451500	-0.78215200	0.56848700
C	4.01839900	-0.62009600	0.15678800
C	2.09445500	0.12780000	1.44177000
C	4.74283500	0.48680400	0.60213100
H	4.45305100	-1.35818100	-0.51918300
C	2.82678700	1.23717600	1.86832500
H	1.06239700	-0.02336000	1.75436200
C	4.14744100	1.42453600	1.45101400

H	5.77723700	0.61952800	0.27634000
H	2.35352900	1.96412300	2.53219900
H	4.71166600	2.29645700	1.78866400
H	-1.87921600	-2.12924900	2.07480600
H	-1.48976800	-3.38663000	0.86097900

9

B3LYP-D3/def2-SVP SCF energy in vacuum: -1492.722257

B3LYP-D3/def2-TZVPP SCF energy in vacuum: -1494.489412

B3LYP-D3/def2-TZVPP free energy in vacuum: -1494.158459

C	-5.71145900	1.02960800	-0.15386600
C	-4.88203400	-0.08434500	-0.27854400
C	-3.50354800	0.06855200	-0.49528800
C	-2.97427000	1.35826100	-0.60535200
C	-3.80636900	2.47563400	-0.47753300
C	-5.17373500	2.31764400	-0.24895600
H	-6.78188900	0.89281300	0.01781500
H	-5.30510700	-1.08927400	-0.19705800
H	-1.91369700	1.50066600	-0.80839400
H	-3.37681800	3.47667300	-0.56316100
H	-5.82091100	3.19229300	-0.14999100
C	-2.65887700	-1.18140000	-0.61999700
H	-3.01446800	-1.74796400	-1.49746400
C	-2.66356900	-2.10369300	0.55958000
C	-3.00619400	-1.66918800	1.94436500
H	-3.63843200	-2.42240800	2.44830900
H	-2.08204500	-1.55994200	2.54089000
H	-3.52576800	-0.70291700	1.97079400
C	-2.00213900	-3.43219400	0.38041900
H	-0.95046500	-3.40338700	0.72892200

H	-2.50477300	-4.21283600	0.97692800
H	-1.98097300	-3.75130100	-0.67342600
O	-1.28805000	-0.84346800	-1.06466500
P	-0.07241700	-0.49700200	-0.10999400
O	-0.29290700	-0.36126200	1.34948800
O	0.50553600	0.83550000	-0.84808300
O	0.97903300	-1.66166800	-0.54830200
C	1.52574700	1.61539700	-0.30720900
C	1.37983500	2.22071800	0.94219500
C	2.68636700	1.77854900	-1.06336100
C	2.43060400	2.99476500	1.44123300
H	0.46798400	2.05522200	1.51544100
C	3.72679900	2.55669500	-0.55349200
H	2.76533400	1.27722600	-2.02848900
C	3.60374500	3.16400900	0.70021900
H	2.32847500	3.46737500	2.42103000
H	4.64184700	2.68052200	-1.13707100
H	4.42164200	3.76811100	1.09907200
C	2.33993700	-1.55146400	-0.28787900
C	3.21538200	-1.80106000	-1.34523400
C	2.81398600	-1.19540600	0.97753800
C	4.59038500	-1.67519300	-1.13603200
H	2.80366800	-2.07645500	-2.31751900
C	4.19067700	-1.05986700	1.16825500
H	2.10401000	-1.00618600	1.78322200
C	5.08130600	-1.29583100	0.11709600
H	5.28074800	-1.86624100	-1.96106100
H	4.56703300	-0.76408400	2.15007600
H	6.15651200	-1.18681400	0.27461800

10

B3LYP-D3/def2-SVP SCF energy in vacuum: -1492.735805

B3LYP-D3/def2-TZVPP SCF energy in vacuum: -1494.507804

B3LYP-D3/def2-TZVPP free energy in vacuum: -1494.172645

C	3.24122600	0.26564400	1.74713600
C	2.05279900	0.81075900	1.28046300
C	2.05551100	1.83317600	0.28699400
C	3.32262500	2.23930800	-0.22113400
C	4.50460000	1.68119000	0.24683800
C	4.47417600	0.69394800	1.24058100
H	3.20749300	-0.52491100	2.49928700
H	1.10636300	0.42905800	1.65737100
H	3.35297700	3.01016000	-0.99529100
H	5.46104500	2.01523500	-0.16302100
H	5.40306400	0.25106900	1.60650300
C	0.88426100	2.45616900	-0.22331500
H	1.02114900	3.20243200	-1.00857200
C	-0.53200600	2.17753800	0.19270800
C	-0.81651800	2.55835200	1.64899600
H	-0.72160600	3.64718500	1.76967800
H	-1.83975600	2.25562000	1.91761100
H	-0.11193800	2.07429500	2.33631000
C	-1.52818700	2.87171100	-0.73845500
H	-1.35283700	2.57898900	-1.78271000
H	-2.55847500	2.61175700	-0.45472800
H	-1.40935300	3.96244600	-0.65726100
O	-0.18512300	0.44492300	-2.40378800
P	-0.80292700	-0.14196100	-1.19675700
O	-0.77197100	0.71401500	0.15915500
O	-0.23338400	-1.57956800	-0.70115400

O	-2.36166200	-0.50932100	-1.40994400
C	1.10947200	-1.88010800	-0.50372500
C	2.11984800	-1.35024500	-1.31165400
C	1.40264200	-2.77039900	0.53030500
C	3.44213800	-1.72292000	-1.06403600
H	1.86279000	-0.65250600	-2.10796400
C	2.72991300	-3.13977000	0.75839500
H	0.58748400	-3.16363800	1.13991300
C	3.75358400	-2.61612400	-0.03599800
H	4.23695500	-1.29921800	-1.68131800
H	2.96274400	-3.83882300	1.56524700
H	4.79187500	-2.89919300	0.14950100
C	-3.31860100	-0.61277400	-0.41351000
C	-3.13355000	-1.43507600	0.70002800
C	-4.49347000	0.12263700	-0.57883300
C	-4.14380700	-1.51056000	1.66175500
H	-2.20947300	-2.00396300	0.80064800
C	-5.49876000	0.03313100	0.38689700
H	-4.60227400	0.75006800	-1.46500100
C	-5.32630800	-0.77985500	1.51112100
H	-4.00381500	-2.15100100	2.53582600
H	-6.42062200	0.60545600	0.25934300
H	-6.11230600	-0.84588100	2.26648000

IM1

B3LYP-D3/def2-SVP SCF energy in vacuum: -1492.721371

B3LYP-D3/def2-TZVPP SCF energy in THF: -1494.491068

B3LYP-D3/def2-TZVPP free energy in THF: -1494.157735

C	-5.71154800	1.02827100	-0.14946800
C	-4.88160700	-0.08504600	-0.27634000

C	-3.50349900	0.06890600	-0.49470900
C	-2.97514600	1.35904800	-0.60413200
C	-3.80774000	2.47578800	-0.47410000
C	-5.17471900	2.31672900	-0.24392800
H	-6.78166900	0.89065600	0.02346900
H	-5.30403900	-1.09028800	-0.19533600
H	-1.91490300	1.50227100	-0.80836500
H	-3.37891300	3.47717900	-0.55923300
H	-5.82228600	3.19088700	-0.14321500
C	-2.65816200	-1.18027600	-0.62180800
H	-3.01463000	-1.74640600	-1.49919300
C	-2.65945300	-2.10361600	0.55751800
C	-3.00535400	-1.67240300	1.94271600
H	-3.64288200	-2.42435700	2.44208700
H	-2.08289100	-1.56954200	2.54321800
H	-3.52046600	-0.70382700	1.97067900
C	-2.00000100	-3.43307500	0.37585700
H	-0.94943300	-3.40758500	0.72830900
H	-2.50619600	-4.21497200	0.96781800
H	-1.97565100	-3.74854700	-0.67898300
O	-1.28814000	-0.84140700	-1.06696500
P	-0.07307400	-0.49517700	-0.11103800
O	-0.29512600	-0.35737600	1.34798600
O	0.50643800	0.83584000	-0.85047600
O	0.97781600	-1.66126800	-0.54684500
C	1.52688600	1.61564800	-0.30991000
C	1.38068400	2.22254400	0.93870100
C	2.68805800	1.77706400	-1.06558300
C	2.43173500	2.99639500	1.43744100
H	0.46836900	2.05841200	1.51160500

C	3.72876800	2.55503900	-0.55601700
H	2.76720700	1.27456800	-2.03008500
C	3.60543800	3.16389300	0.69691700
H	2.32938300	3.47023900	2.41661700
H	4.64424300	2.67752600	-1.13921000
H	4.42354900	3.76785800	1.09553900
C	2.33860700	-1.55172600	-0.28546200
C	3.21473000	-1.80358500	-1.34170700
C	2.81184900	-1.19407500	0.97980300
C	4.58965100	-1.67838300	-1.13155300
H	2.80362700	-2.08017700	-2.31391000
C	4.18847600	-1.05922300	1.17145700
H	2.10132300	-1.00311500	1.78459200
C	5.07980100	-1.29743600	0.12139400
H	5.28056300	-1.87119400	-1.95571000
H	4.56423100	-0.76219100	2.15313100
H	6.15495300	-1.18894200	0.27963700

TS_a

B3LYP-D3/def2-SVP SCF energy in vacuum: -1492.713466

B3LYP-D3/def2-TZVPP SCF energy in THF: -1494.48836

B3LYP-D3/def2-TZVPP free energy in THF: -1494.155963

C	3.45165400	-0.33273000	1.42793400
C	2.44608900	0.55436300	1.06692300
C	2.59659800	1.37129400	-0.08206200
C	3.75602400	1.21781000	-0.87944000
C	4.76427100	0.33945600	-0.50166100
C	4.61565600	-0.43409100	0.65659400
H	3.31539700	-0.97920000	2.29606100
H	1.50316600	0.58335500	1.61190100

H	3.85720400	1.81292300	-1.79007500
H	5.66316300	0.24190900	-1.11423000
H	5.39817300	-1.14006400	0.94361300
C	1.59053000	2.31087900	-0.50161100
H	1.62358200	2.61037000	-1.55033700
C	0.68791300	3.01692000	0.31837500
O	-0.00323700	0.95216100	-1.65080500
P	-0.85886400	0.39001700	-0.53169500
O	-0.67604800	0.94791800	0.86410500
O	-0.74259400	-1.26331600	-0.48102100
O	-2.41489200	0.48605800	-1.03104000
C	0.45715700	-1.90548000	-0.32404200
C	0.63388700	-2.72650300	0.79583100
C	1.46979900	-1.78793900	-1.28721600
C	1.81845200	-3.45144400	0.93948600
H	-0.17228200	-2.78871600	1.52862100
C	2.64828800	-2.51746100	-1.13101600
H	1.31019300	-1.12244300	-2.13521500
C	2.82874600	-3.35177000	-0.02314300
H	1.95166000	-4.10073000	1.80856500
H	3.43693800	-2.42787300	-1.88161600
H	3.75342400	-3.92228800	0.09050900
C	-3.51607600	-0.01220300	-0.37432700
C	-4.64246500	-0.27190900	-1.16274400
C	-3.55047000	-0.23687100	1.00792300
C	-5.80784700	-0.75764000	-0.56849500
H	-4.57996400	-0.08863000	-2.23678200
C	-4.72277800	-0.72911800	1.58724900
H	-2.66811300	-0.01415800	1.60785900
C	-5.85397400	-0.99166600	0.80948700

H	-6.68443300	-0.95918500	-1.18934600
H	-4.74881200	-0.90586000	2.66569000
H	-6.76522000	-1.37595400	1.27331000
C	0.86575400	3.21337700	1.78734700
H	0.02392500	2.73379300	2.30914600
H	1.80614800	2.80220400	2.17015100
H	0.82727200	4.29294800	2.01356900
C	-0.40477900	3.79880500	-0.33162300
H	-1.33340200	3.67462100	0.24572900
H	-0.15402200	4.87591800	-0.31829900
H	-0.58142500	3.46775500	-1.36191500

TS_b

B3LYP-D3/def2-SVP SCF energy in vacuum: -1492.70601

B3LYP-D3/def2-TZVPP SCF energy in THF: -1494.482276

B3LYP-D3/def2-TZVPP free energy in THF: -1494.148633

0 2

C	-5.74398000	0.85422100	-0.36906900
C	-4.77783600	-0.14326500	-0.50116100
C	-3.40788500	0.18306000	-0.49558900
C	-3.03487200	1.53905500	-0.37374100
C	-4.00125500	2.52856200	-0.22818200
C	-5.35994600	2.18982100	-0.22341000
H	-6.80300300	0.58687400	-0.38378600
H	-5.09642800	-1.17542600	-0.64148600
H	-1.97352700	1.79209800	-0.36491100
H	-3.69666800	3.57166300	-0.11777200
H	-6.11860100	2.96831900	-0.11342600
C	-2.34569100	-0.80537300	-0.67165000
H	-1.47224700	-0.43553800	-1.21305300

C	-2.41271200	-2.19670200	-0.49982900
C	-3.41276000	-2.86190600	0.38715600
H	-4.19209100	-3.37247700	-0.20862900
H	-2.90963700	-3.64439000	0.97769300
H	-3.89278000	-2.15690400	1.07574400
C	-1.33018200	-3.05604600	-1.05987000
H	-0.58055500	-3.23172200	-0.26638200
H	-1.72291100	-4.03600900	-1.37500600
H	-0.80859500	-2.58048400	-1.90195000
O	-1.43531400	-0.61183300	1.23133600
P	0.04994600	-0.20845800	1.27357600
O	0.63174200	0.34990800	2.52240300
O	0.22905100	0.80861000	-0.03182300
O	0.84989000	-1.55078400	0.71991000
C	1.37214600	1.55791000	-0.25693000
C	1.81718600	2.49250700	0.68196500
C	2.05960400	1.36158300	-1.45643400
C	2.97249400	3.22879000	0.40922200
H	1.27632000	2.59988900	1.62162200
C	3.21109200	2.10521400	-1.71805700
H	1.69801300	0.60728800	-2.15646300
C	3.67283400	3.03975100	-0.78594900
H	3.32976800	3.95473000	1.14378000
H	3.75626000	1.94373900	-2.65096300
H	4.57785800	3.61660500	-0.98932800
C	2.18114100	-1.52777100	0.35022800
C	2.53047300	-2.16964300	-0.84141900
C	3.15259500	-0.88377100	1.12508100
C	3.85832700	-2.14891800	-1.27309600
H	1.75294500	-2.66506200	-1.42465400

C	4.47347000	-0.85911900	0.67444700
H	2.85171200	-0.39126800	2.04981300
C	4.83320200	-1.48557000	-0.52256500
H	4.12920500	-2.64705300	-2.20735300
H	5.22895700	-0.34000600	1.26885700
H	5.86918900	-1.45954200	-0.86749700

IM2

B3LYP-D3/def2-SVP SCF energy in vacuum: -1492.737312

B3LYP-D3/def2-TZVPP SCF energy in THF: -1494.510686

B3LYP-D3/def2-TZVPP free energy in THF: -1494.174176

C	-3.23742400	-0.26189900	1.75291900
C	-2.05091600	-0.80652200	1.28345100
C	-2.05572900	-1.83094700	0.28786400
C	-3.32752900	-2.23569500	-0.21780200
C	-4.50680500	-1.67722300	0.25384200
C	-4.47328000	-0.68982800	1.24928800
H	-3.20183600	0.52834600	2.50534700
H	-1.10321100	-0.42529100	1.65768100
H	-3.36019600	-3.00602400	-0.99241600
H	-5.46482500	-2.01009600	-0.15334600
H	-5.40091100	-0.24679300	1.61811100
C	-0.88962900	-2.45345200	-0.22322600
H	-1.02857800	-3.20244900	-1.00602000
C	0.52979500	-2.17674400	0.18896900
C	0.81721800	-2.55959800	1.64382400
H	0.71715600	-3.64798800	1.76450800
H	1.84279700	-2.26223900	1.90942800
H	0.11729600	-2.07229700	2.33362400
C	1.52141500	-2.87284100	-0.74558700

H	1.34333100	-2.57965100	-1.78922300
H	2.55320000	-2.61516100	-0.46516700
H	1.40061400	-3.96333900	-0.66403900
O	0.18996800	-0.44323600	-2.40885400
P	0.80553800	0.14240200	-1.20013800
O	0.77158500	-0.71457600	0.15510600
O	0.23532800	1.57972200	-0.70441100
O	2.36475700	0.50954100	-1.40976700
C	-1.10778800	1.87898400	-0.50692500
C	-2.11780300	1.34731400	-1.31412900
C	-1.40170700	2.76968100	0.52656000
C	-3.44051300	1.71828900	-1.06613000
H	-1.86017000	0.64951800	-2.11021500
C	-2.72934600	3.13735600	0.75499600
H	-0.58682300	3.16433100	1.13562000
C	-3.75269500	2.61162900	-0.03847400
H	-4.23503100	1.29305400	-1.68273900
H	-2.96279400	3.83661800	1.56148600
H	-4.79129200	2.89330900	0.14740900
C	3.31950800	0.61228000	-0.41111200
C	3.13240900	1.43448800	0.70215000
C	4.49431100	-0.12380500	-0.57386000
C	4.14053300	1.50922900	1.66617400
H	2.20846300	2.00392400	0.80076500
C	5.49745300	-0.03506600	0.39417100
H	4.60480400	-0.75115100	-1.45987400
C	5.32293100	0.77784200	1.51813500
H	3.99892400	2.14962600	2.54002000
H	6.41924500	-0.60794200	0.26860900
H	6.10721500	0.84323500	2.27532800

9) Gram-scale Reaction and X-ray Diffraction Single Crystal

Gram-scale Reaction

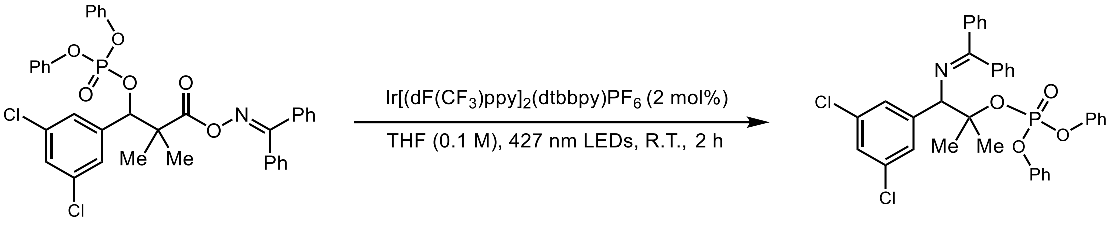

An oven-dried vial (40 ml) containing a stirring bar was charged with **S27** (1.35g, 2 mmol, 1.0 equiv.), Ir[(dF(CF$_3$)ppy]$_2$(dtbbpy)PF$_6$ (40 mg, 0.04 mmol, 2 mol%), then THF (20 mL) was added to the mixture in the glovebox. The reaction was stirred and irradiated using two 40 W 440 nm blue LED lamps (5 cm away, room temperature) for 2 h. After the starting material was consumed, the reaction solution was concentrated in vacuo. The residue was purified by flash chromatography on silica gel (PE/EA = 20/1 to 10/1) to afford the white solid **27** (0.53g, 42% yield).

X-ray Diffraction Single Crystal

Method for single crystals cultivation: a pure solid sample **27** (10 mg) was dissolved in DCM (2 mL) in a vial at room temperature, and PE (1 mL) was added into the above solution slowly while keeping the sample completely dissolved. The vial was properly sealed with parafilm and kept at room temperature to allow the slow evaporation of the solvents until a single crystal was obtained.

A suitable crystal was selected and checked on a ROD, Synergy Custom system, HyPix diffractometer. The crystal was kept at 100.00(10) K during data collection. Using Olex2[6], the structure was solved with the SHELXS[7] structure solution program using Direct Methods and refined with the SHELXL[8] refinement package using Least Squares minimisation.

Crystallographic data for compound **27** (**CCDC 2512135**) has been deposited with the Cambridge Crystallographic Data Centre, Copies of the data can be obtained, free of charge, on application to CCDC (Email:deposit@ccdc.cam.ac.uk).

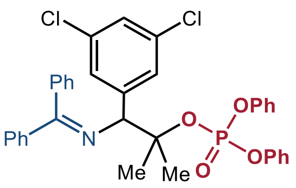

27

≡

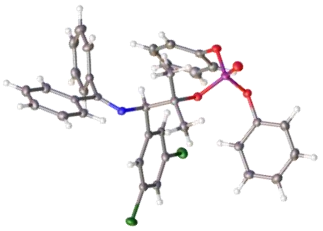

CCDC 2512135

Bond precision: C-C = 0.0040 A Wavelength=1.54184

Cell:	a=9.3929(4)	b=10.1580(4)	c=16.1976(6)
	alpha=89.661(3)	beta=87.684(3)	gamma=82.322(3)
Temperature:	100 K		

	Calculated	Reported
Volume	1530.36(11)	1530.36(10)
Space group	P -1	P -1
Hall group	-P 1	-P 1
Moiety formula	C35 H30 Cl2 N O4 P	C35 H30 Cl2 N O4 P
Sum formula	C35 H30 Cl2 N O4 P	C35 H30 Cl2 N O4 P
Mr	630.47	630.47
Dx,g cm-3	1.368	1.368
Z	2	2
Mu (mm-1)	2.733	2.733
F000	656.0	656.0
F000’	659.71	
h,k,lmax	11,12,20	11,12,19
Nref	6073	5666
Tmin,Tmax	0.716,0.761	0.625,1.000
Tmin’	0.650	

Correction method= # Reported T Limits: Tmin=0.625 Tmax=1.000
AbsCorr = MULTI-SCAN

Data completeness= 0.933 Theta(max)= 72.545

R(reflections)= 0.0540(4696) wR2(reflections)= 0.1830(5666)

S = 1.072 Npar= 390

10) Experiment of N-N Dual Functional Group Transposition Reaction

Substrate Synthesis

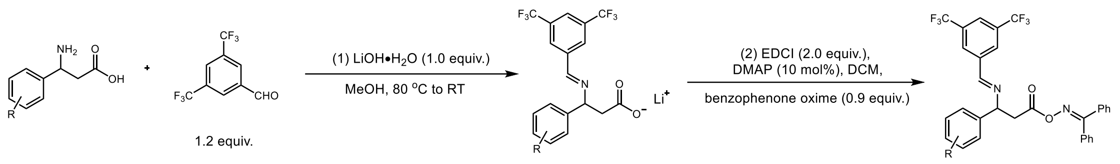

Schiff base reaction. To a solution of LiOH·H_2O (1.0 equiv.) in MeOH (0.3 M, reflux in 80 °C), 3-amino-3-phenylpropanoic acid (1.0 equiv.) was added. The reaction mixture was stirred for 0.5 h at 80 °C. Then the reaction mixture was cooled to room temperature, and 3,5-bis(trifluoromethyl)benzaldehyde (1.2 equiv.) was added to stirring for another 4.5 h. Then the mixture was filtered, dried and concentrated to obtain the crude lithium salt of imino benzyl carboxylate (**c1**).

Condensation reaction. To a solution of **c1** (1.0 equiv.) in DCM (0.25 M), EDCI (2.0 equiv.) and DMAP (1.5 equiv.) were added to mix well, and benzophenone oxime (0.9 equiv.) was subsequently added. The reaction mixture was stirred for 12 h at room temperature. The reaction was quenched with water, extracted with EtOAc, and the organic phases were combined, dried, concentrated, and purified through column chromatography to obtain the final product.

(*E*)-Diphenylmethanone O-(3-((3,5-bis(trifluoromethyl)benzylidene)amino)-3-phenylpropanoyl) oxime (S43)

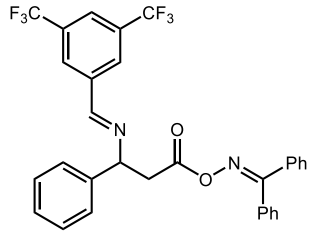

Prepared following the general procedure C outlined above starting from 3-amino-3-phenylpropanoic acid and 3,5-bis(trifluoromethyl)benzaldehyde on 10 mmol scale to give **S43** (4.2 g, 74% total yield) as a white solid.

^{1}H NMR (400 MHz, CDCl$_3$) δ 8.28 (s, 1H), 8.08 (s, 2H), 7.79 (s, 1H), 7.45 – 7.40 (m, 2H), 7.38 – 7.27 (m, 6H), 7.23 (dd, J = 8.0, 1.4 Hz, 4H), 7.13 (d, J = 4.4 Hz, 3H), 4.82 (dd, J = 9.6, 3.9 Hz, 1H), 3.16 – 2.71 (m, 2H).

^{13}C NMR (100 MHz, CDCl$_3$) δ 168.75, 165.22, 158.98, 141.86, 138.04, 134.67, 132.56, 132.06 (q, J = 33.0 Hz), 131.12, 129.84, 129.17, 128.95, 128.50, 128.43 (q, J = 2.7 Hz), 128.32, 127.95, 127.02, 124.16 (q, J = 8.2 Hz), 123.12 (q, J = 273.7 Hz), 70.63, 41.60.

^{19}F NMR (100 MHz, CD$_2$Cl$_2$) -62.90.

HRMS (ESI-TOF) m/z calcd. for $C_{31}H_{23}F_6N_2O_2^+$ ([M+H]$^+$) 569.1658, found: 569.1652.

(*E*)-diphenylmethanone O-(3-((3,5-bis(trifluoromethyl)benzylidene)amino)-3-(p-tolyl)propanoyl) oxime (S44)

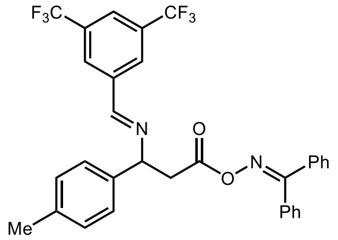

Prepared following the general procedure C outlined above starting from 3-amino-3-(p-tolyl)propanoic acid and 3,5-bis(trifluoromethyl)benzaldehyde on 10 mmol scale to give **S44** (2.8 g, 48% total yield) as a white solid.

^{1}H NMR (400 MHz, CDCl$_3$) δ 8.36 (s, 1H), 8.17 (s, 2H), 7.89 (s, 1H), 7.53 (d, J = 7.2 Hz, 2H), 7.49 – 7.39 (m, 4H), 7.35 (d, J = 7.7 Hz, 2H), 7.30 (s, 1H), 7.25 – 7.22 (m, 2H), 7.16 (d, J = 7.9 Hz, 2H), 4.89 (dd, J = 9.5, 3.9 Hz, 1H), 3.13 – 2.88 (m, 2H), 2.33 (s, 3H).

^{13}C NMR (100 MHz, CDCl$_3$) δ 168.86, 165.19, 158.79, 138.81, 138.07, 137.73, 134.68, 132.55, 132.06 (q, *J* = 33.2 Hz), 131.14, 129.87, 129.62, 129.20, 128.99, 128.52, 128.32, 126.94, 123.14 (q, *J* = 273.5 Hz), 70.37, 41.52, 21.25.

^{19}F NMR (100 MHz, CD$_2$Cl$_2$) -62.90.

HRMS (ESI-TOF) m/z calcd. for $C_{32}H_{25}F_6N_2O_2^+$ ([M+H]$^+$) 583.1815, found: 583.1812.

(*E*)-diphenylmethanone O-(3-((3,5-bis(trifluoromethyl)benzylidene)amino)-3-(4-fluorophenyl)propanoyl) oxime (S45)

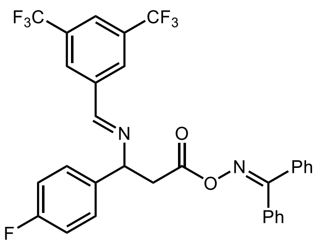

Prepared following the general procedure C outlined above starting from 3-amino-3-(4-fluorophenyl)propanoic acid and 3,5-bis(trifluoromethyl)benzaldehyde on 10 mmol scale to give **S45** (3.2 g, 55% total yield) as a white solid.

^{1}H NMR (400 MHz, CDCl$_3$) δ 8.39 (s, 1H), 8.18 (s, 2H), 7.91 (s, 1H), 7.55 – 7.50 (m, 2H), 7.48 – 7.31 (m, 8H), 7.24 (dd, *J* = 8.0, 1.4 Hz, 2H), 7.06 – 7.02 (m, 2H), 4.91 (dd, *J* = 9.3, 4.2 Hz, 1H), 3.06 (dd, *J* = 16.3, 9.4 Hz, 1H), 2.90 (dd, *J* = 16.3, 4.2 Hz, 1H).

^{13}C NMR (100 MHz, CDCl$_3$) δ 168.61, 165.31, 162.35 (d, *J* = 246.2 Hz), 159.08, 137.88, 137.61 (d, *J* = 3.2 Hz), 134.61, 132.52, 132.16 (q, *J* = 33.2 Hz), 131.19, 129.18, 128.93, 128.68, 128.60, 128.53, 128.42 (q, *J* = 2.7 Hz), 128.34, 124.30 (q, *J* = 8.6 Hz), 123.32 (q, *J* = 272.6 Hz), 115.91, 115.70, 69.95, 41.75.

^{19}F NMR (100 MHz, CD$_2$Cl$_2$) -62.91, -114.37 – -114.44 (m).

HRMS (ESI-TOF) m/z calcd. for $C_{31}H_{22}F_7N_2O_2^+$ ([M+H]$^+$) 587.1564, found: 587.1561.

(*E*)-diphenylmethanone O-(3-((3,5-bis(trifluoromethyl)benzylidene)amino)-3-(4-chlorophenyl)propanoyl) oxime (S46)

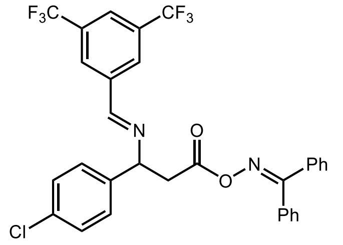

Prepared following the general procedure C outlined above starting from 3-amino-3-(4-chlorophenyl)propanoic acid and3,5-bis(trifluoromethyl)benzaldehyde on 5 mmol scale to give **S46 (**2.1 g, 70% total yield) as a white solid.

^{1}H NMR (400 MHz, CD_2Cl_2) δ 8.37 (s, 1H), 8.22 (s, 2H), 7.95 (s, 1H), 7.54 – 7.42 (m, 6H), 7.40 – 7.30 (m, 6H), 7.25 (dd, *J* = 7.9, 1.5 Hz, 2H), 4.89 (dd, *J* = 9.1, 4.5 Hz, 1H), 3.11 – 2.90 (m, 2H).

^{13}C NMR (100 MHz, CD_2Cl_2) δ 168.59, 165.26, 159.44, 140.88, 138.32, 135.05, 133.73, 132.77, 132.20 (q, *J* = 33.0 Hz), 131.39, 130.13, 129.22, 129.20, 128.85, 128.83, 128.75 (d, *J* = 2.6 Hz), 128.74, 128.62, 124.64 (dd, *J* = 8.0, 2.6 Hz), 123.66 (q, *J* = 273.7 Hz), 70.17, 41.89.

^{19}F NMR (100 MHz, CD_2Cl_2) -69.29.

HRMS (ESI-TOF) m/z calcd. for $C_{31}H_{22}ClF_6N_2O_2^+$ ([M+H]$^+$) 603.1269, found: 603.1268.

Experiment Procedure and Data

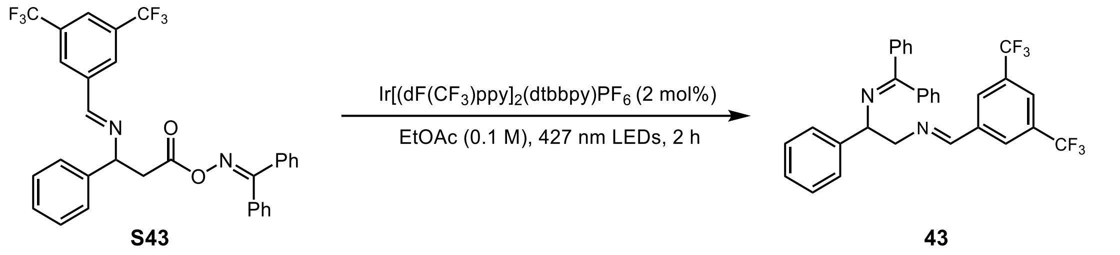

An oven-dried vial (8 mL) containing a stirring bar was charged with **S43** (114 mg, 0.2 mmol, 1.0 equiv.), Ir[(dF(CF_3)ppy]$_2$(dtbbpy)PF_6 (4.5 mg, 0.004 mmol, 2 mol%), then EtOAc (2 mL) was added to the mixture in the glovebox. The reaction was stirred and irradiated using 40 W 427 nm Kessil blue LED lamps (5 cm away, room temperature) for 2 h. After the starting material was consumed the reaction was concentrated in vacuo. The residue was purified by flash chromatography on silica gel (PE/EA = 30/1 to 20/1) to afford the desired product **43** (54.6 mg, 52% yield) as a viscous liquid. The target compound is sensitive to trace amounts of water, and its NMR spectrum was acquired using deuterated benzene.

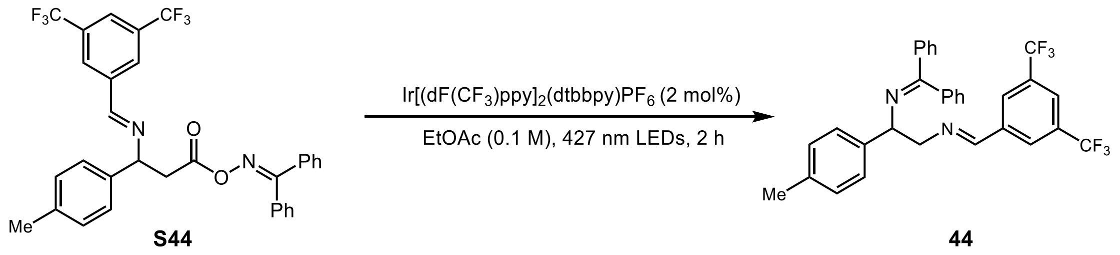

An oven-dried vial (8 mL) containing a stirring bar was charged with **S44** (117 mg, 0.2 mmol, 1.0 equiv.), Ir[(dF(CF_3)ppy]$_2$(dtbbpy)PF_6 (4.5 mg, 0.004 mmol, 2 mol%), then EtOAc (2 mL) was added to the mixture in the glovebox. The reaction was stirred and irradiated using 40 W 427 nm Kessil blue LED lamps (5 cm away, room temperature) for 2 h. After the starting material was consumed the reaction was concentrated in vacuo. The residue was purified by flash chromatography on silica gel (PE/EA = 30/1 to 20/1) to afford the desired product **44** (53.0 mg, 50% yield) as a viscous liquid. The target compound is sensitive to trace amounts of water, and its NMR spectrum was acquired using deuterated benzene.

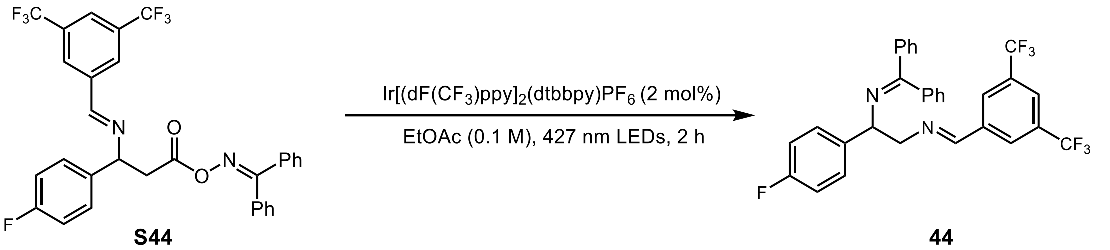

S44 **44**

An oven-dried vial (8 mL) containing a stirring bar was charged with **S44** (117 mg, 0.2 mmol, 1.0 equiv.), Ir[(dF(CF$_3$)ppy]$_2$(dtbbpy)PF$_6$ (4.5 mg, 0.004 mmol, 2 mol%), then EtOAc (2 mL) was added to the mixture in the glovebox. The reaction was stirred and irradiated using 40 W 427 nm Kessil blue LED lamps (5 cm away, room temperature) for 2 h. After the starting material was consumed the reaction was concentrated in vacuo. The residue was purified by flash chromatography on silica gel (PE/EA = 30/1 to 20/1) to afford the desired product **44** (45.6 mg, 42% yield) as a viscous liquid. The target compound is sensitive to trace amounts of water, and its NMR spectrum was acquired using deuterated benzene.

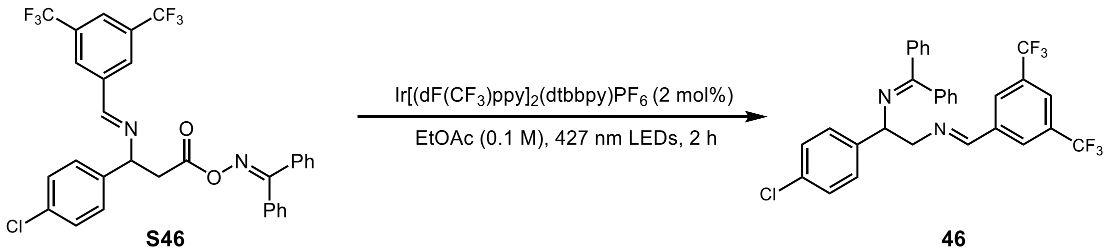

S46 **46**

An oven-dried vial (8 mL) containing a stirring bar was charged with **S46** (121 mg, 0.2 mmol, 1.0 equiv.), Ir[(dF(CF$_3$)ppy]$_2$(dtbbpy)PF$_6$ (4.5 mg, 0.004 mmol, 2 mol%), then EtOAc (2 mL) was added to the mixture in the glovebox. The reaction was stirred and irradiated using 40 W 427 nm Kessil blue LED lamps (5 cm away, room temperature) for 2 h. After the starting material was consumed the reaction was concentrated in vacuo. The residue was purified by flash chromatography on silica gel (PE/EA = 30/1 to 20/1) to afford the desired product **43** (51.4 mg, 46% yield) as a viscous liquid. The target compound is sensitive to trace amounts of water, and its NMR spectrum was acquired using deuterated benzene.

(*E*)-*N*-(2-((3,5-bis(trifluoromethyl)benzylidene)amino)-1-phenylethyl)-1,1-diphenylmethanimine (43)

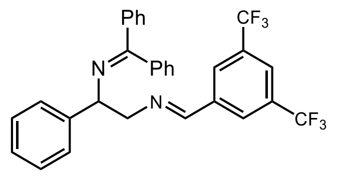

^{1}H NMR (400 MHz, C_6D_6) 7.83 – 7.81 (m, 3H), 7.59 (s, 1H), 7.56 (s, 1H), 7.54 (s, 1H), 7.24 (dd, *J* = 7.6 4.0 Hz, 2H), 7.12 (d, *J* = 7.4 Hz, 5H), 7.09 – 7.02 (m, 4H), 6.95 (d, *J* = 6.9 Hz, 2H), 5.15 (dd, *J* = 8.2, 4.2 Hz, 1H), 4.14 – 4.01 (m, 2H).

^{13}C NMR (100 MHz, C_6D_6) δ 168.33, 158.70, 143.50, 140.21, 138.62, 137.36, 131.90 (q, *J* = 30.2 Hz), 130.41, 129.01, 128.94, 128.70, 128.56, 127.61, 123.56 (q, *J* = 271.5 Hz), 69.74, 67.03.

^{19}F NMR (376 MHz, C_6D_6) δ -62.76.

HRMS (ESI-TOF) m/z calcd. for $C_{30}H_{23}F_6N_2^+$ ($[M+H]^+$) 525.1760, found: 525.1758.

(E)-*N*-(2-((3,5-bis(trifluoromethyl)benzylidene)amino)-1-(p-tolyl)ethyl)-1,1-diphenylmethanimine (44)

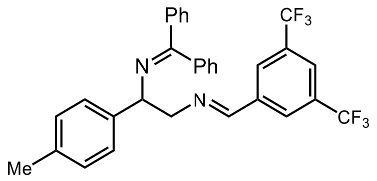

^{1}H NMR (400 MHz, C_6D_6) δ 7.84 (d, *J* = 4.4 Hz, 4H), 7.58 (d, *J* = 9.8 Hz, 2H), 7.49 (d, *J* = 7.9 Hz, 2H), 7.13 (s, 1H), 7.10 – 6.98 (m, 9H), 5.16 (dd, *J* = 8.0, 4.5 Hz, 1H), 4.16 – 4.07 (m, 2H), 2.14 (s, 3H).

^{13}C NMR (100 MHz, C_6D_6) δ 168.15, 158.66, 140.58, 140.29, 138.68, 137.45, 136.98, 131.93 (q, *J* = 30.4 Hz), 130.37, 129.62, 129.01, 128.69, 123.53 (q, *J* = 272.0 Hz), 69.86, 66.83, 21.10.

^{19}F NMR (376 MHz, C_6D_6) δ -62.68.

HRMS (ESI-TOF) m/z calcd. for $C_{31}H_{25}F_6N_2^+$ ($[M+H]^+$) 539.1916, found: 539.1914.

(*E*)-*N*-(2-((3,5-bis(trifluoromethyl)benzylidene)amino)-1-(4-fluorophenyl)ethyl)-1,1-diphenylmethanimine (45)

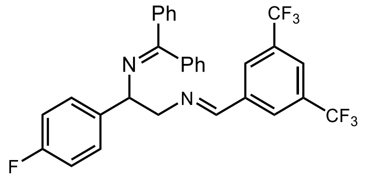

^{1}H NMR (400 MHz, C_6D_6) δ 7.82 (s, 2H), 7.81 – 7.77 (m, 2H), 7.60 (s, 1H), 7.54 (s, 1H), 7.32 (dd, J = 8.6, 5.5 Hz, 2H), 7.13 – 7.03 (m, 6H), 6.94 – 6.86 (m, 4H), 5.05 (dd, J = 8.1, 4.3 Hz, 1H), 4.02 – 3.89 (m, 2H).

^{13}C NMR (100 MHz, C_6D_6) δ 168.51, 162.54 (d, J = 245.2 Hz), 158.81, 158.01, 140.06, 139.15, 139.12, 138.52, 137.25, 131.96 (q, J = 32.4 Hz), 130.56, 129.25, 129.18, 128.97, 128.80, 128.76, 128.60, 123.65 (q, J = 275.5 Hz), 115.67 (d, J = 21.1 Hz), 69.63, 66.14.

^{19}F NMR (376 MHz, C_6D_6) δ -62.76, -115.06 – -115.14 (m).

HRMS (ESI-TOF) m/z calcd. for $C_{30}H_{22}F_7N_2^+$ ($[M+H]^+$) 543.1666, found: 543.1663.

(*E*)-*N*-(2-((3,5-bis(trifluoromethyl)benzylidene)amino)-1-(4-chlorophenyl)ethyl)-1,1-diphenylmethanimine (46)

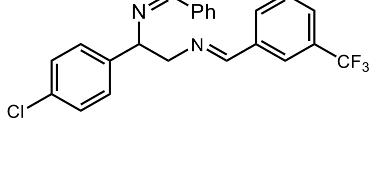

^{1}H NMR (400 MHz, C_6D_6) δ 7.82 (s, 2H), 7.78 (dd, *J* = 8.3, 4.6 Hz, 2H), 7.60 (s, 1H), 7.53 (s, 1H), 7.25 (d, *J* = 8.4 Hz, 2H), 7.20 (s, 1H), 7.12 (s, 3H), 7.09 – 7.03 (m, 4H), 6.91 (d, *J* = 7.0 Hz, 2H), 5.01 (dd, *J* = 8.0, 4.2 Hz, 1H), 4.00 – 3.89 (m, 2H).

^{13}C NMR (100 MHz, C_6D_6) δ 168.74, 158.88, 141.86, 140.01, 138.49, 137.20, 133.34, 132.14, 131.81, 130.60, 129.07, 128.98, 128.83, 128.62, 128.30, 127.34, 123.63 (q, *J* = 271.2 Hz), 69.43, 66.21.

^{19}F NMR (100 MHz, C_6D_6) δ -62.77.

HRMS (ESI-TOF) m/z calcd. for $C_{30}H_{22}ClF_6N_2^+$ ([M+H]$^+$) 559.1370 found: 559.1363.

11) References

1. Stephens, P. J.; Devlin, F. J.; Chabalowski, C. F.; Frisch, M. J., *J. Chem. Phys.* **1994,** *98,* 11623-11627.
2. Grimme, S.; Antony, J.; Ehrlich, S.; Krieg, H., *J. Chem. Phys.* **2010,** *132,* 154104.
3. Davidson, E. R.; Feller, D., Basis set selection for molecular calculations. *Chem. Rev.* **1986,** *86,* 681-696.
4. Frisch, M. J.; Trucks, G. W.; Schlegel, H. B.; Scuseria, G. E.; Robb, M. A.; Cheeseman, JR; Scalmani, G.; Barone, V.; Petersson, G. A.; Nakatsuji, H. *Gaussian 16*; Gaussian, Inc. Wallingford, CT, **2016**.
5. CYLview20; Legault, C. Y., Université de Sherbrooke. CYLview, 1.0 b, Université de Sherbrooke, Sherbrooke, Quebec, Canada, 2020, (http://www.cylview.org).
6. Dolomanov, O.V., Bourhis, L.J., Gildea, R.J, Howard, J.A.K. & Puschmann, H., *J. Appl. Cryst.* **2009**, 42, 339-341.
7. Sheldrick, G.M., *Acta Cryst.* **2008**, A64, 112-122.
8. Sheldrick, G.M., *Acta Cryst.* **2015**, C71, 3-8.

12) Spectral Data

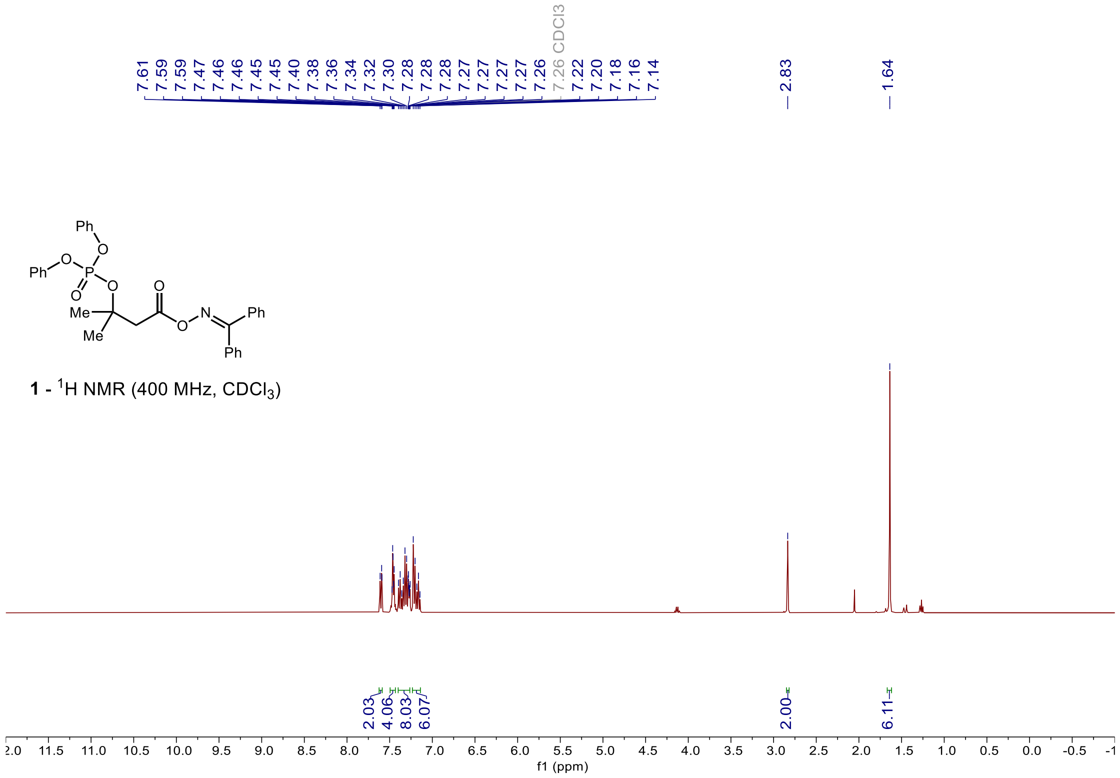

1 - ^{1}H NMR (400 MHz, $CDCl_3$)

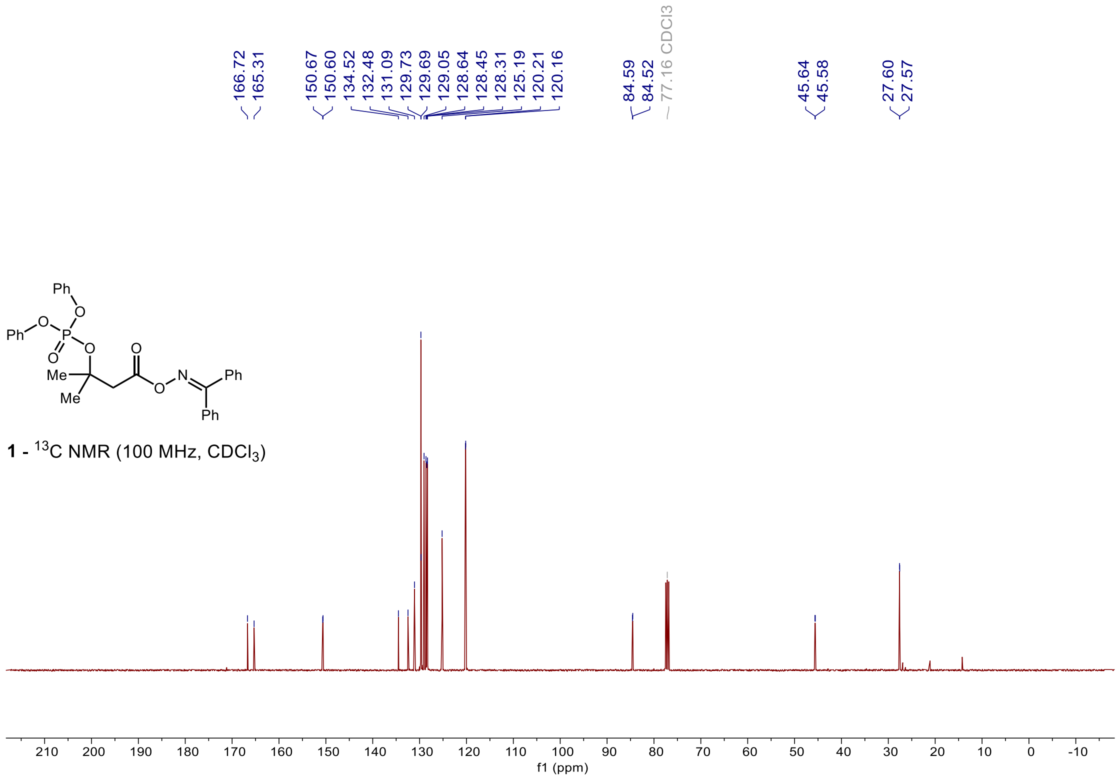

1 - ^{13}C NMR (100 MHz, $CDCl_3$)

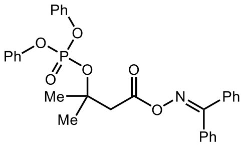

1 - ^{31}P NMR (160 MHz, $CDCl_3$)

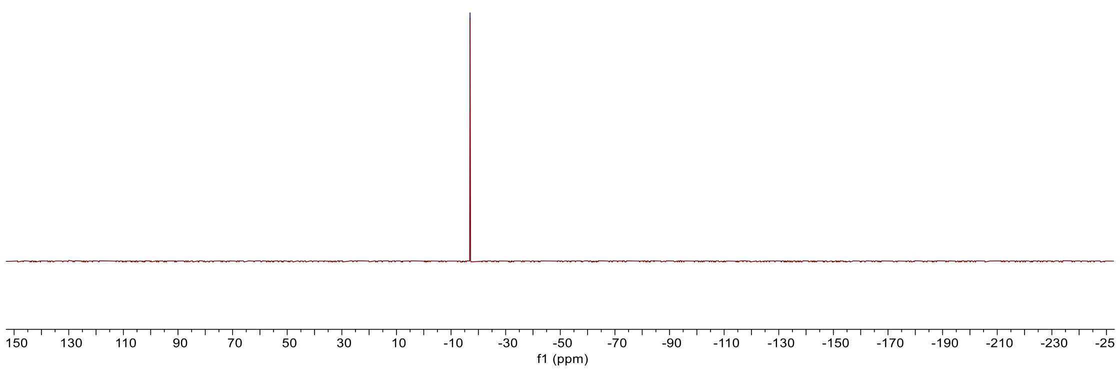

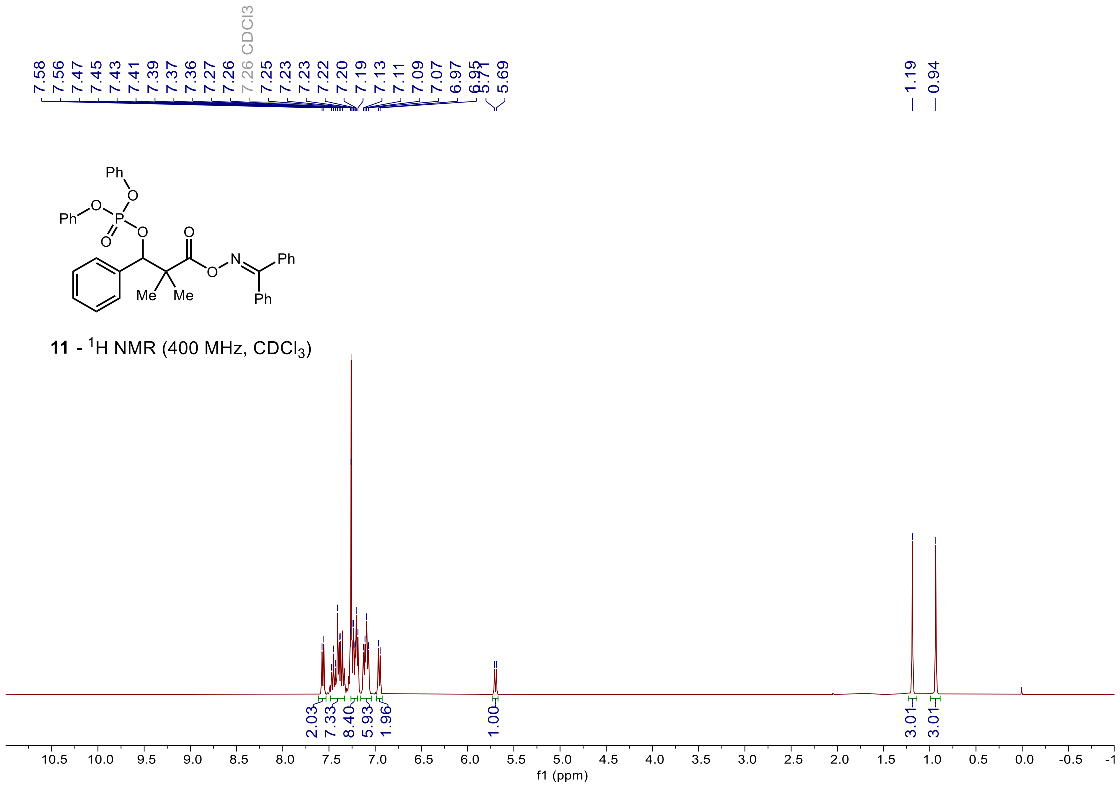

11 - ^{1}H NMR (400 MHz, $CDCl_3$)

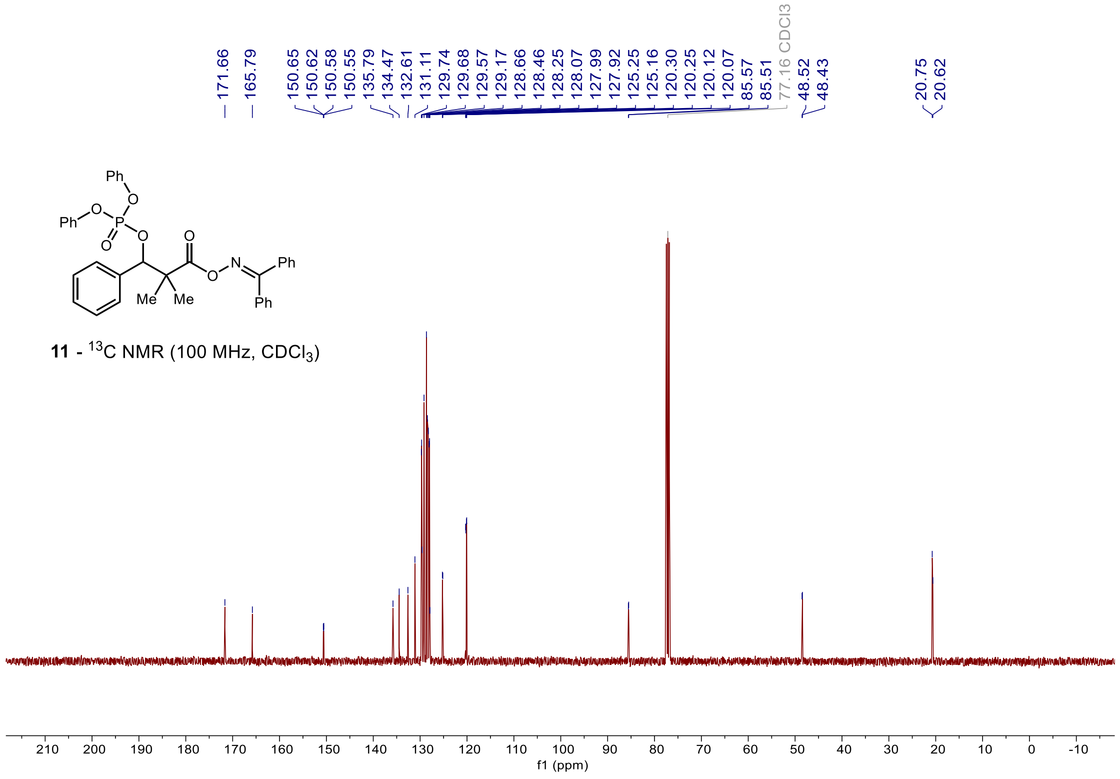

11 - ^{13}C NMR (100 MHz, $CDCl_3$)

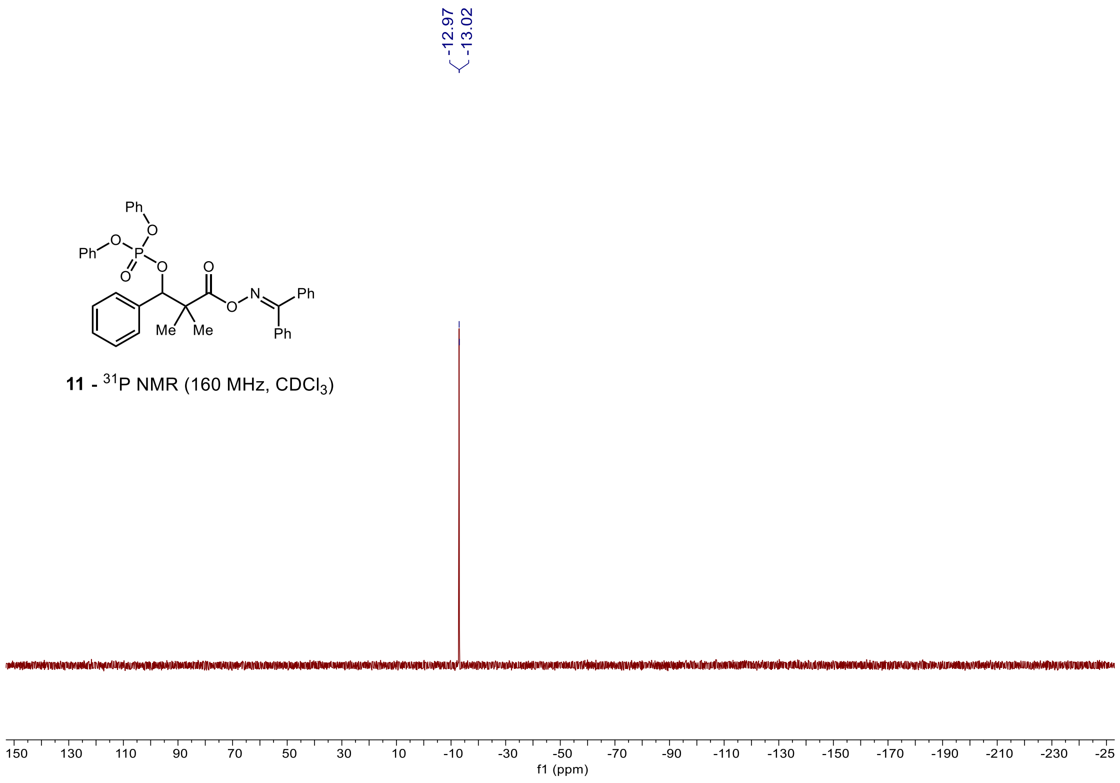

11 - ^{31}P NMR (160 MHz, $CDCl_3$)

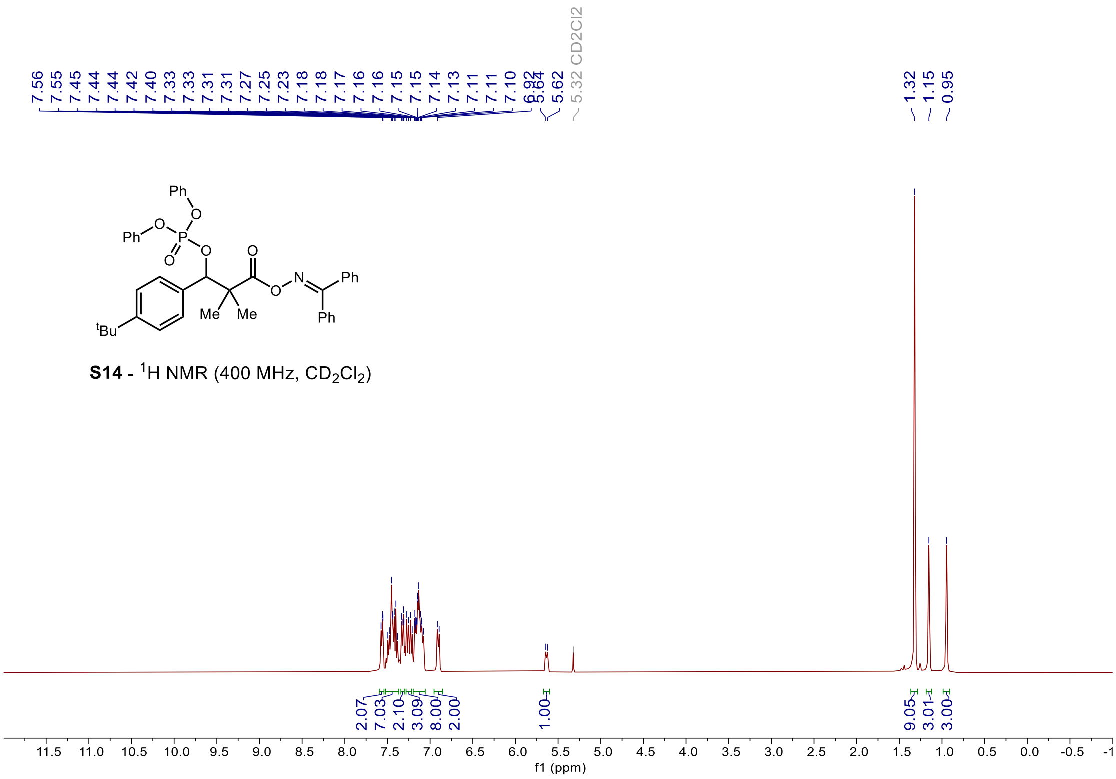

S14 - ^{1}H NMR (400 MHz, CD_2Cl_2)

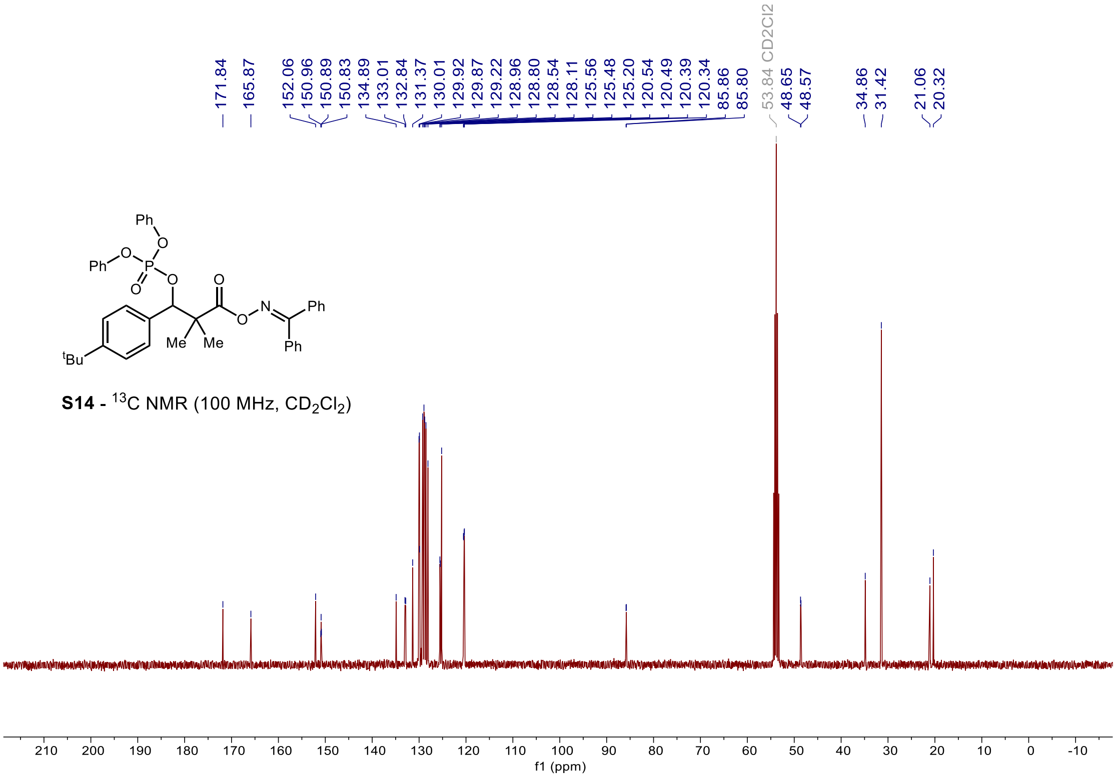

S14 - ^{13}C NMR (100 MHz, CD_2Cl_2)

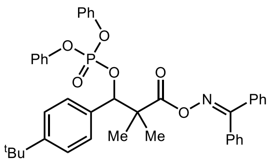

S14 - ^{31}P NMR (160 MHz, CD_2Cl_2)

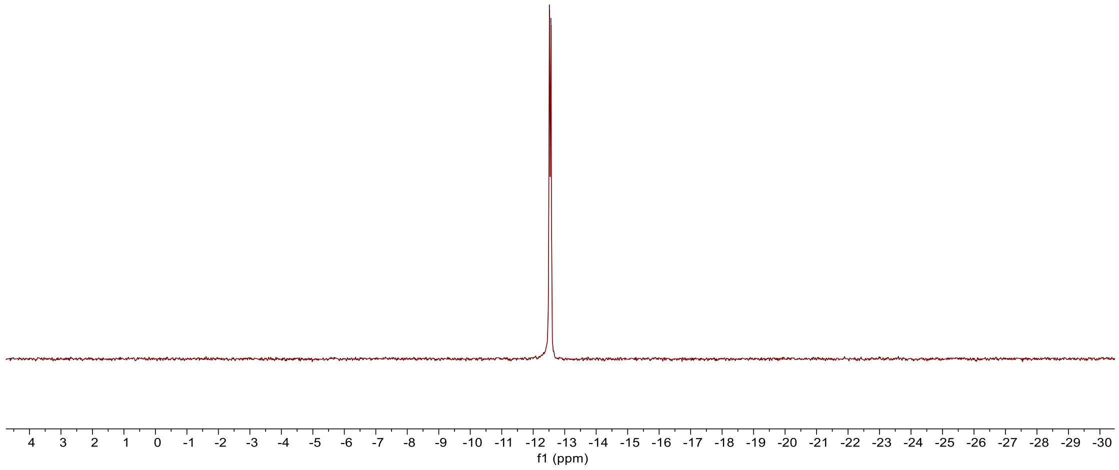

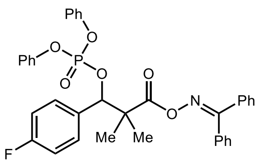

S15 - ^{1}H NMR (400 MHz, CD_2Cl_2)

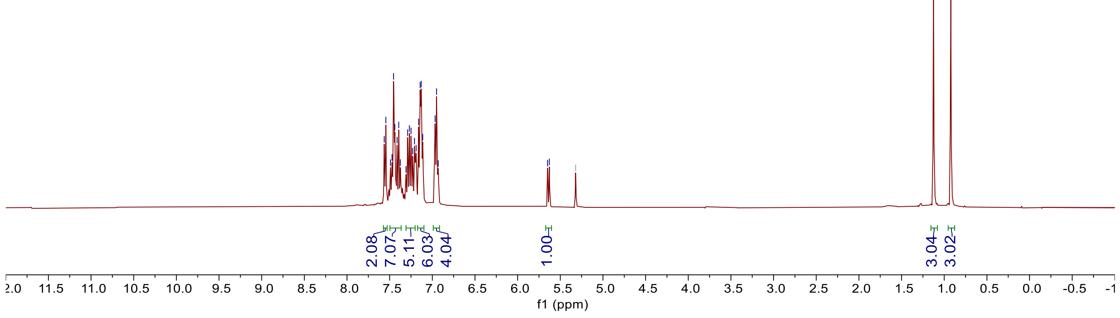

S15 - ^{13}C NMR (100 MHz, CD_2Cl_2)

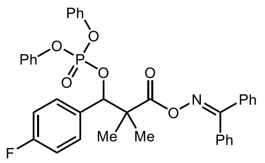

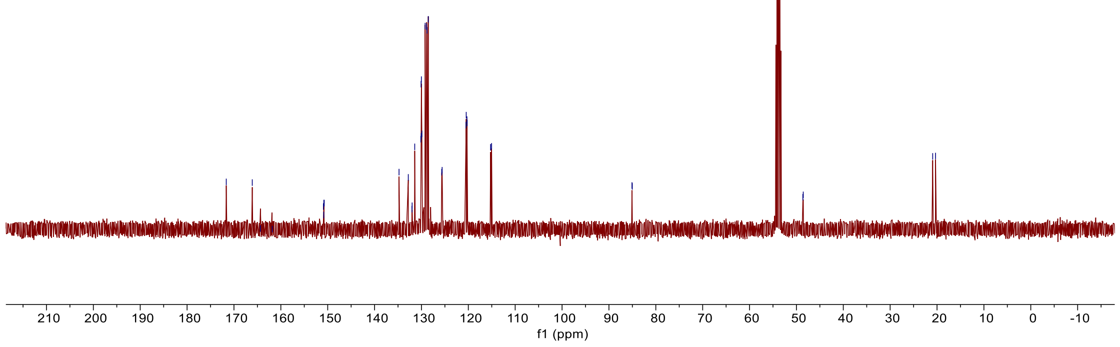

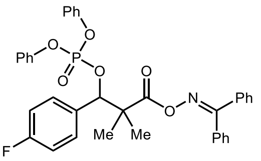

S15 - ^{31}P NMR (160 MHz, CD_2Cl_2)

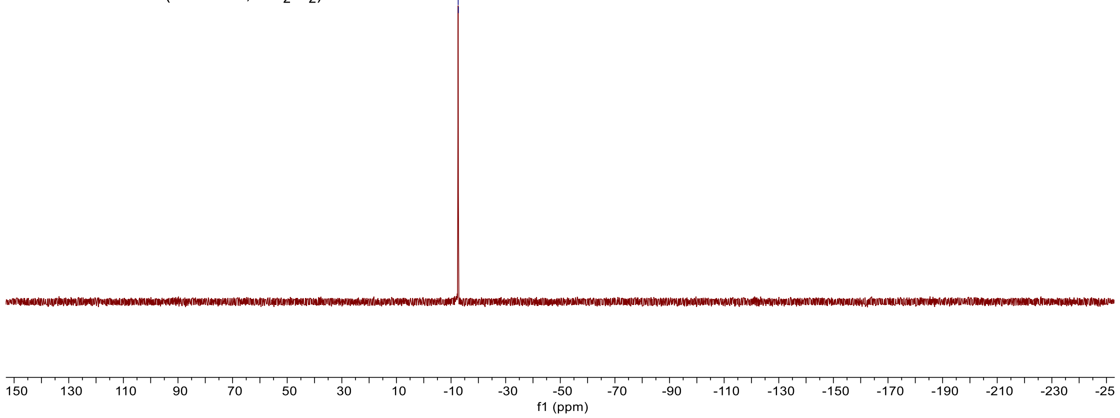

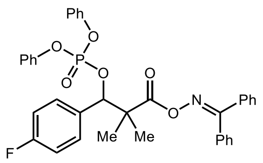

4 - ^{19}F NMR (376 MHz, CD_2Cl_2)

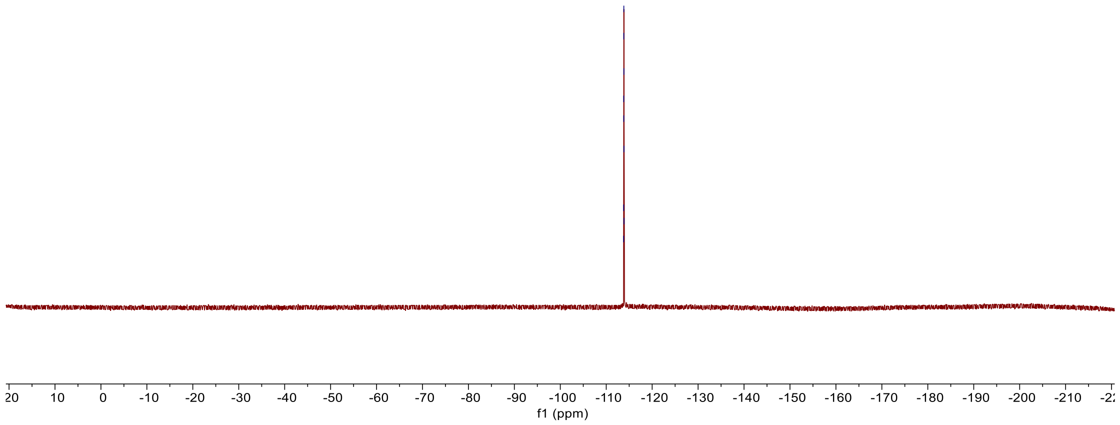

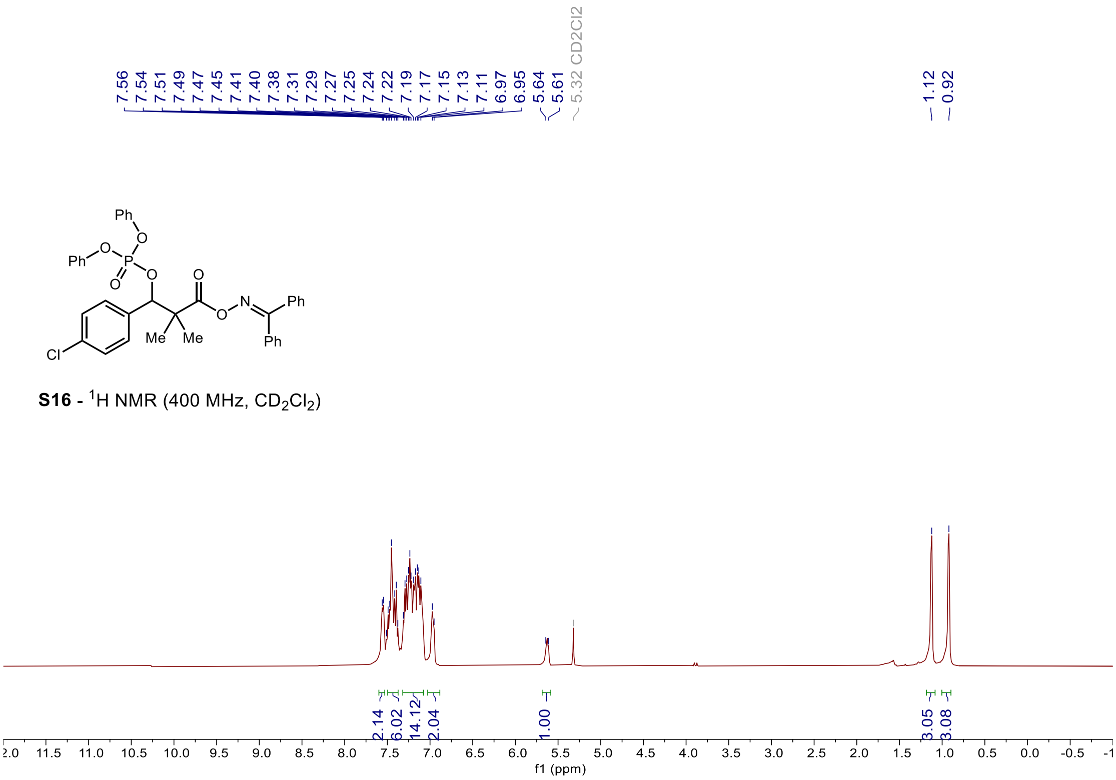

S16 - ^{1}H NMR (400 MHz, CD_2Cl_2)

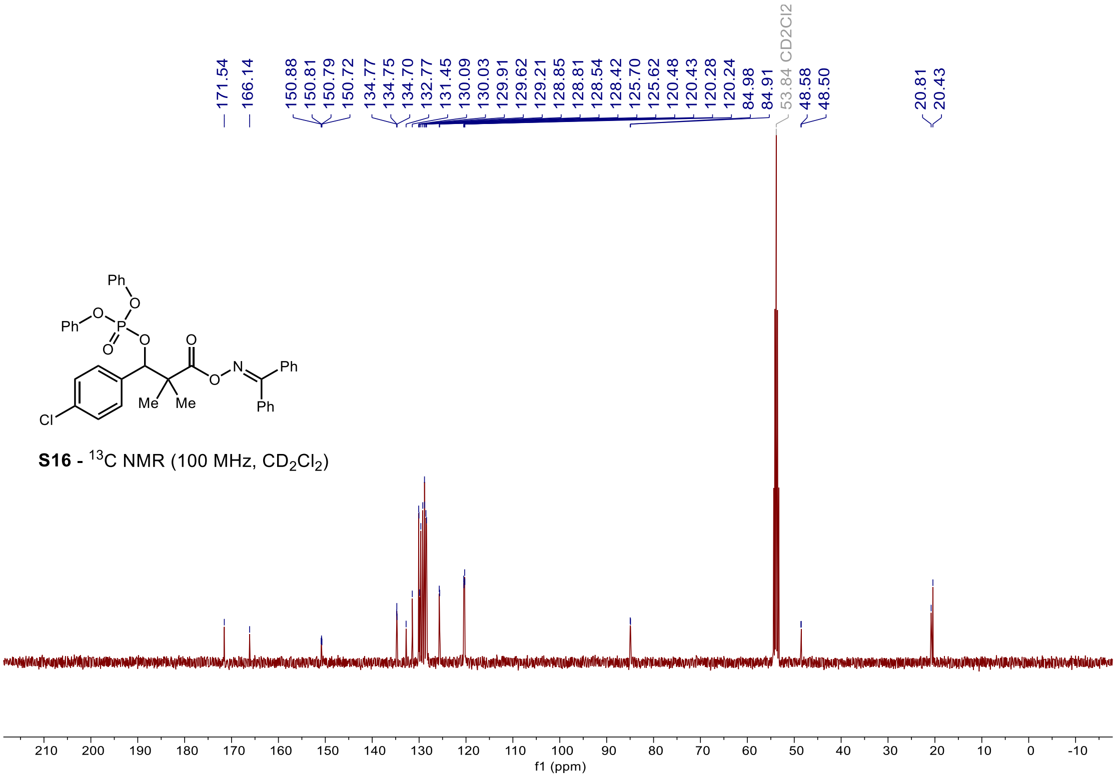

S16 - ^{13}C NMR (100 MHz, CD_2Cl_2)

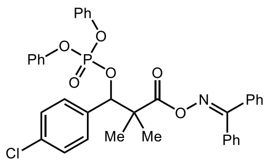

S16 - ^{31}P NMR (160 MHz, CD_2Cl_2)

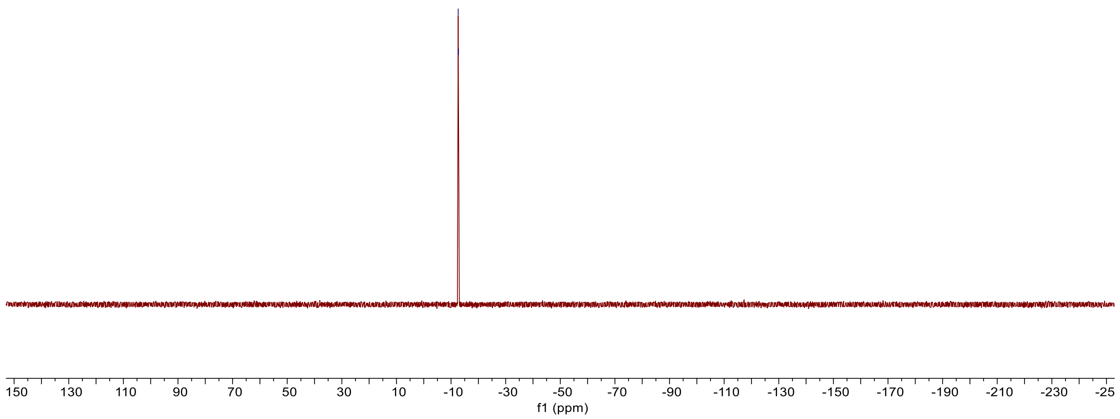

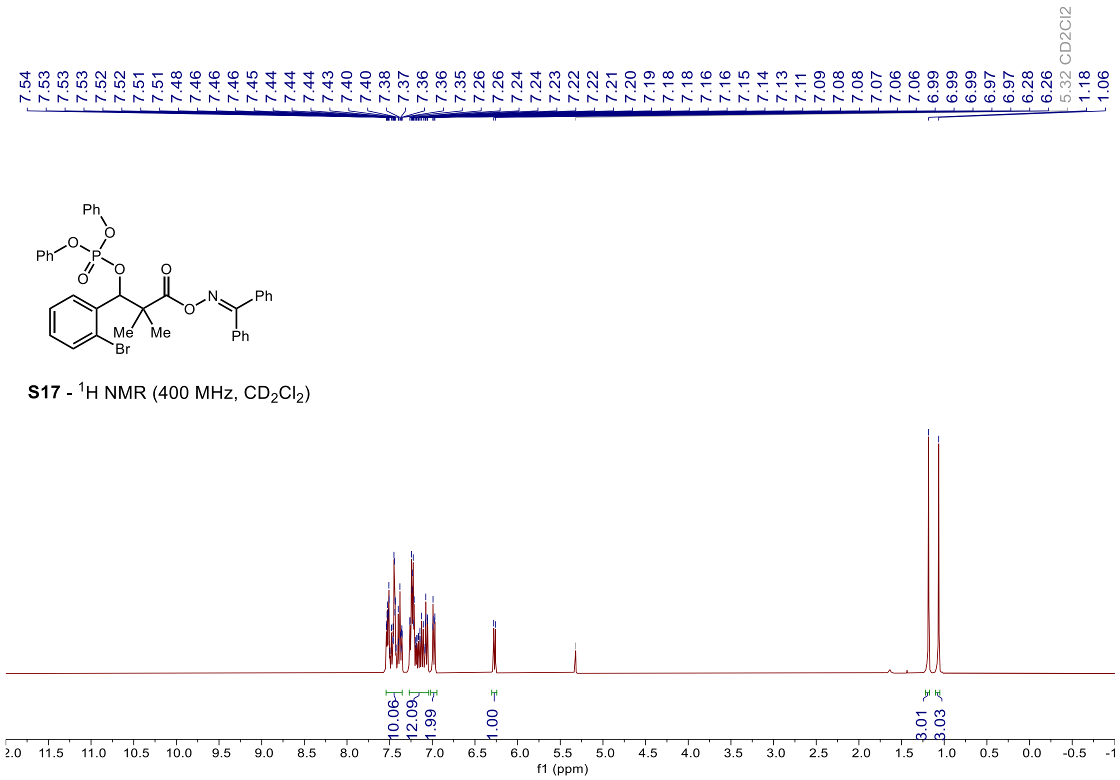

S17 - ^{1}H NMR (400 MHz, CD_2Cl_2)

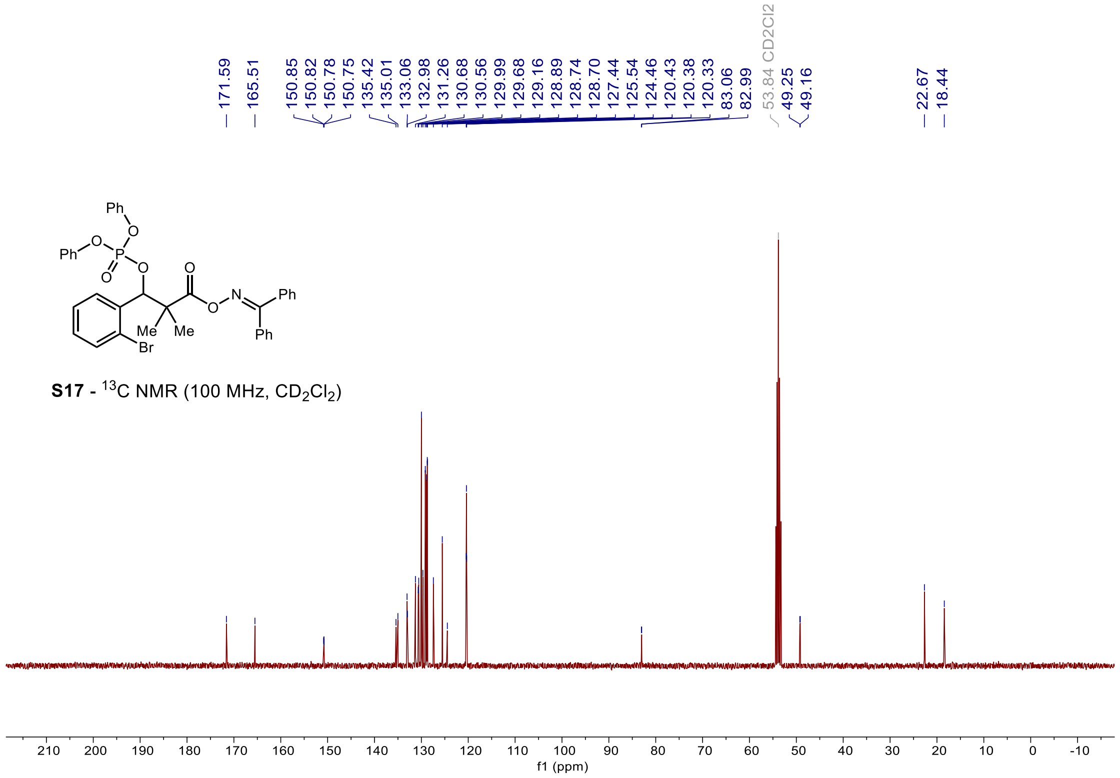

S17 - ^{13}C NMR (100 MHz, CD_2Cl_2)

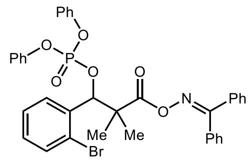

S17 - ^{31}P NMR (160 MHz, CD_2Cl_2)

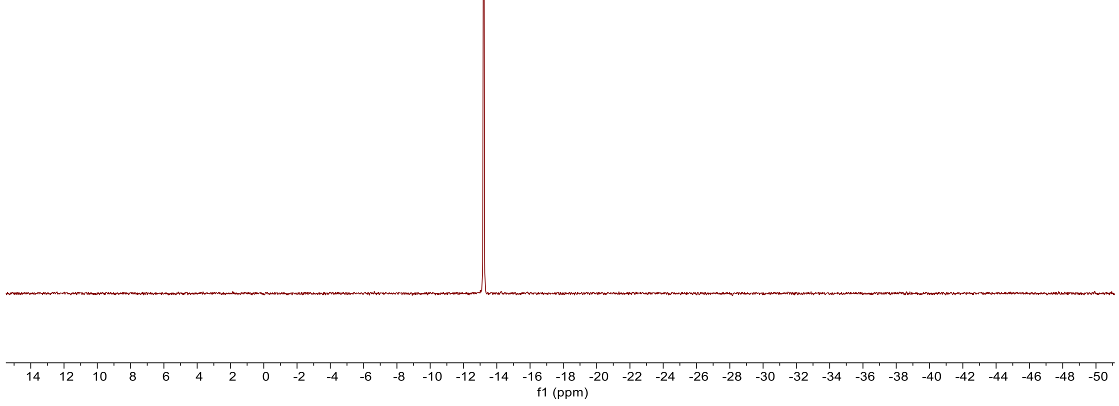

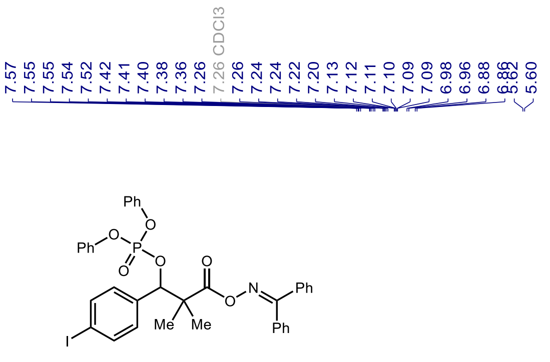

S18 - ^{1}H NMR (400 MHz, $CDCl_3$)

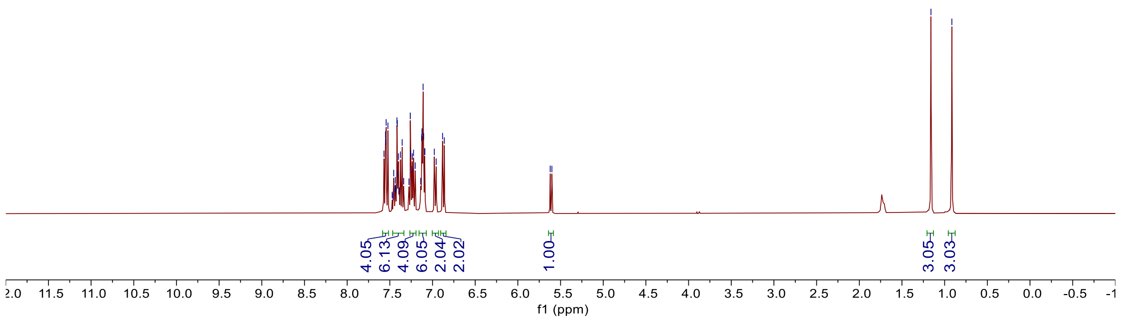

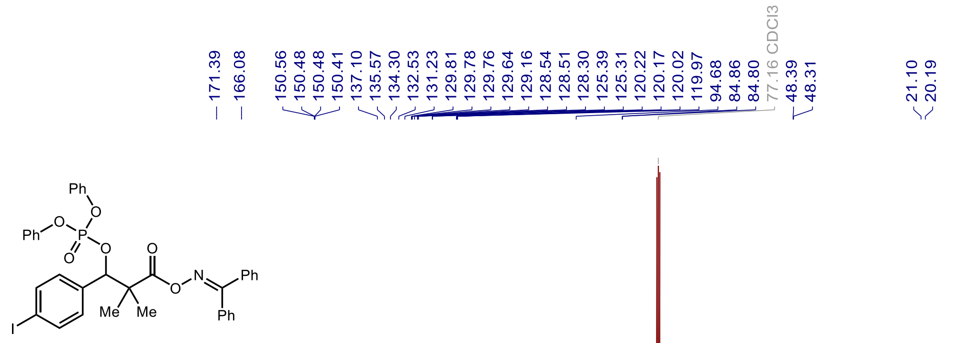

S18 - ^{13}C NMR (100 MHz, $CDCl_3$)

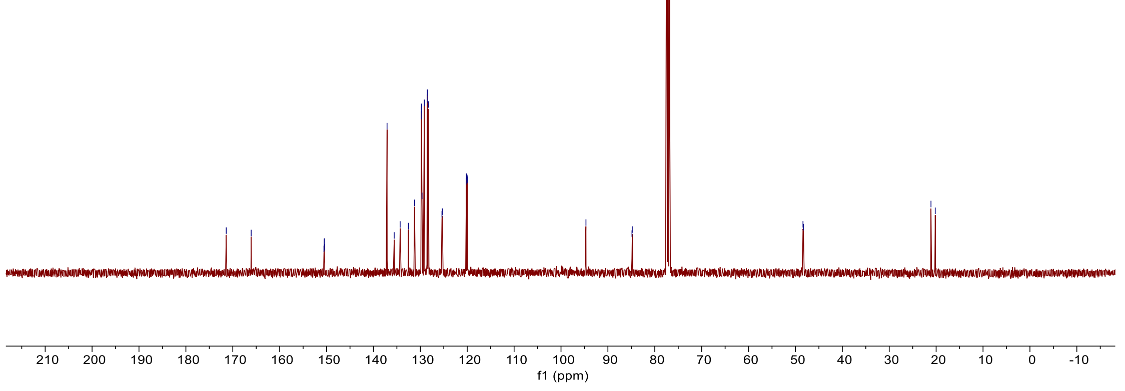

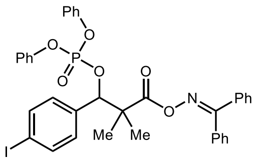

S18 - ^{31}P NMR (160 MHz, $CDCl_3$)

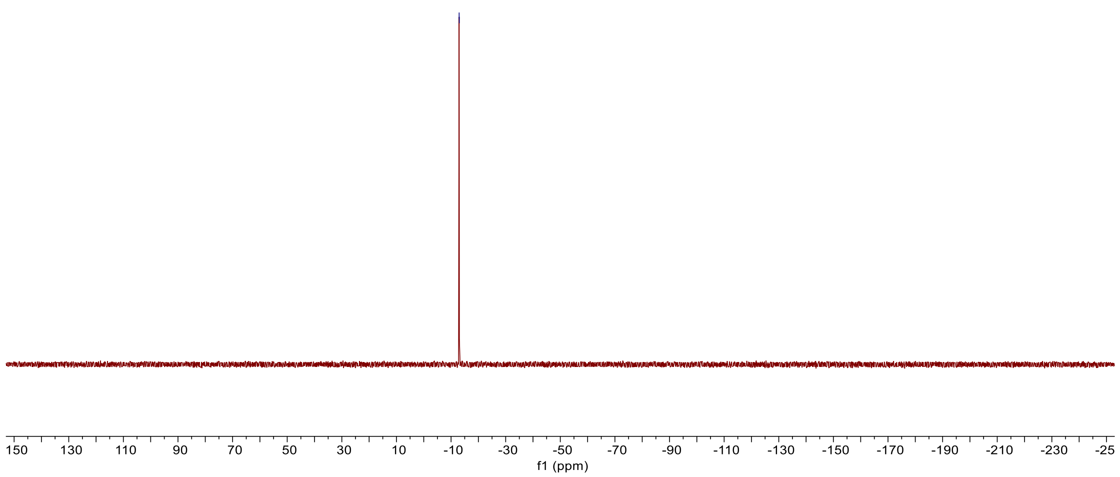

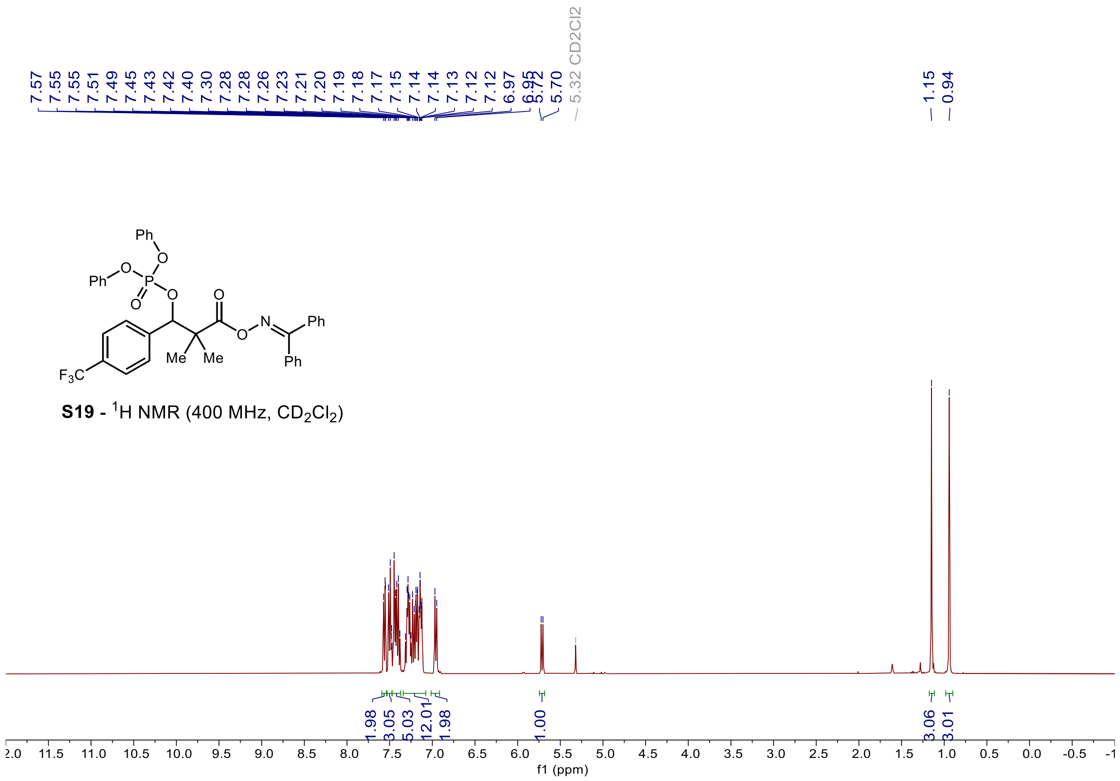

S19 - ^{1}H NMR (400 MHz, CD_2Cl_2)

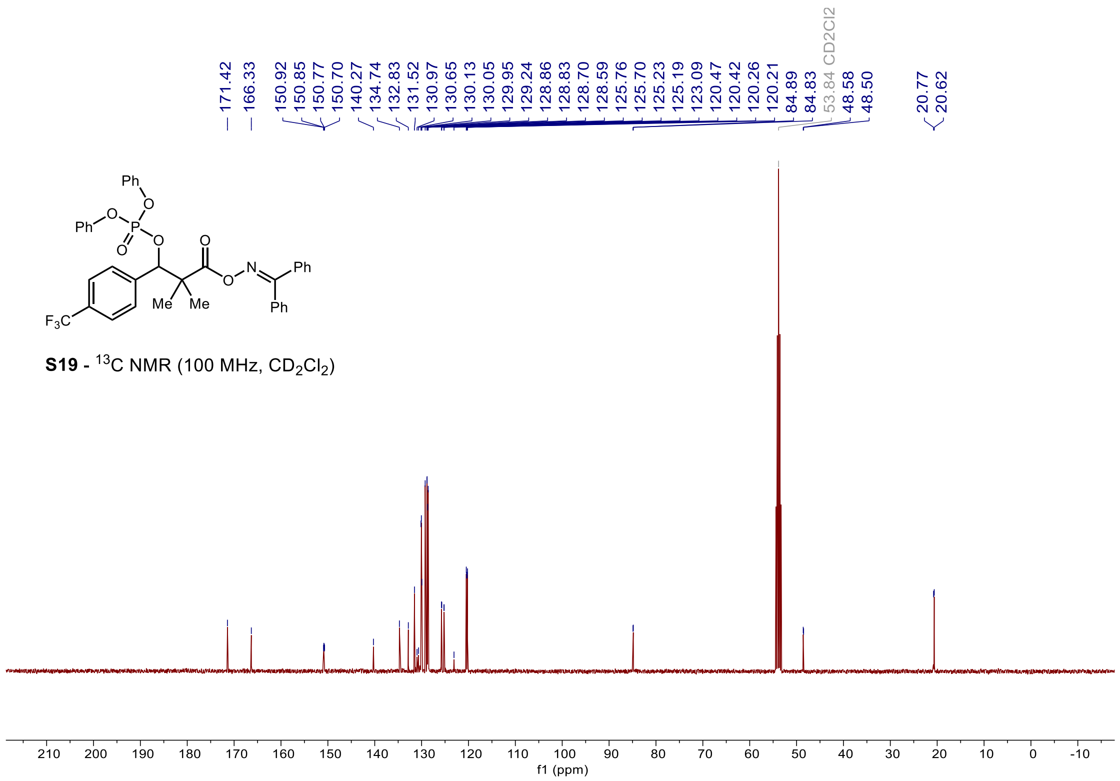

S19 - ^{13}C NMR (100 MHz, CD_2Cl_2)

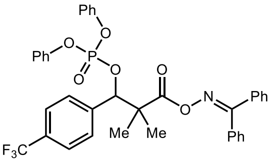

S19 - ^{31}P NMR (160 MHz, CD_2Cl_2)

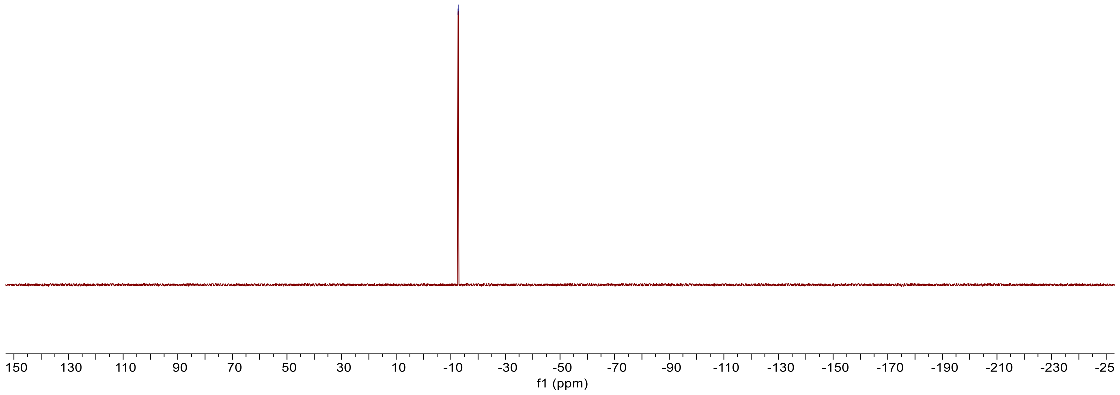

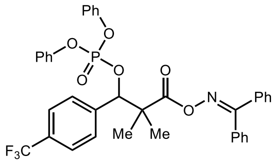

S19 - ^{19}F NMR (376 MHz, CD_2Cl_2)

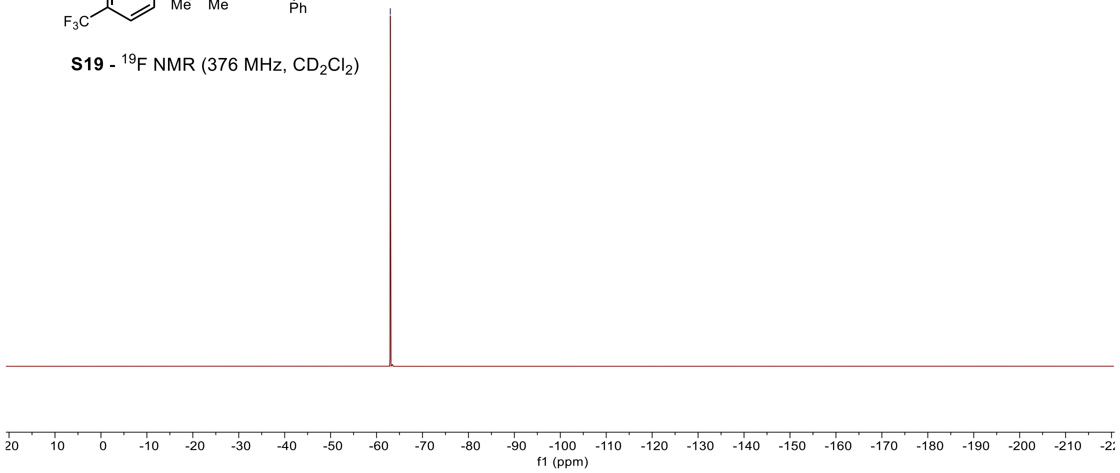

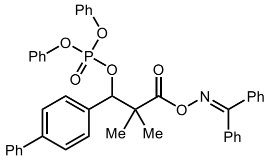

S20 - ^{1}H NMR (400 MHz, CD_2Cl_2)

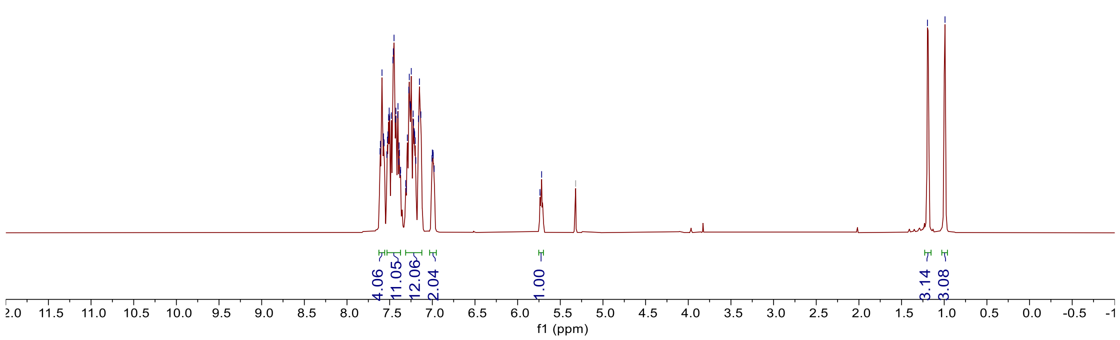

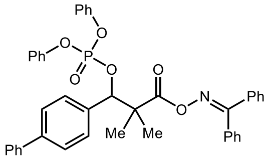

S20 - ^{13}C NMR (100 MHz, CD_2Cl_2)

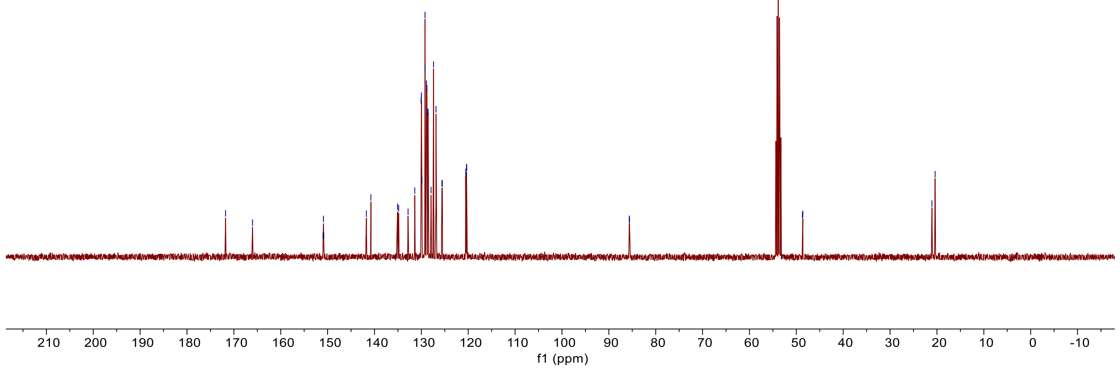

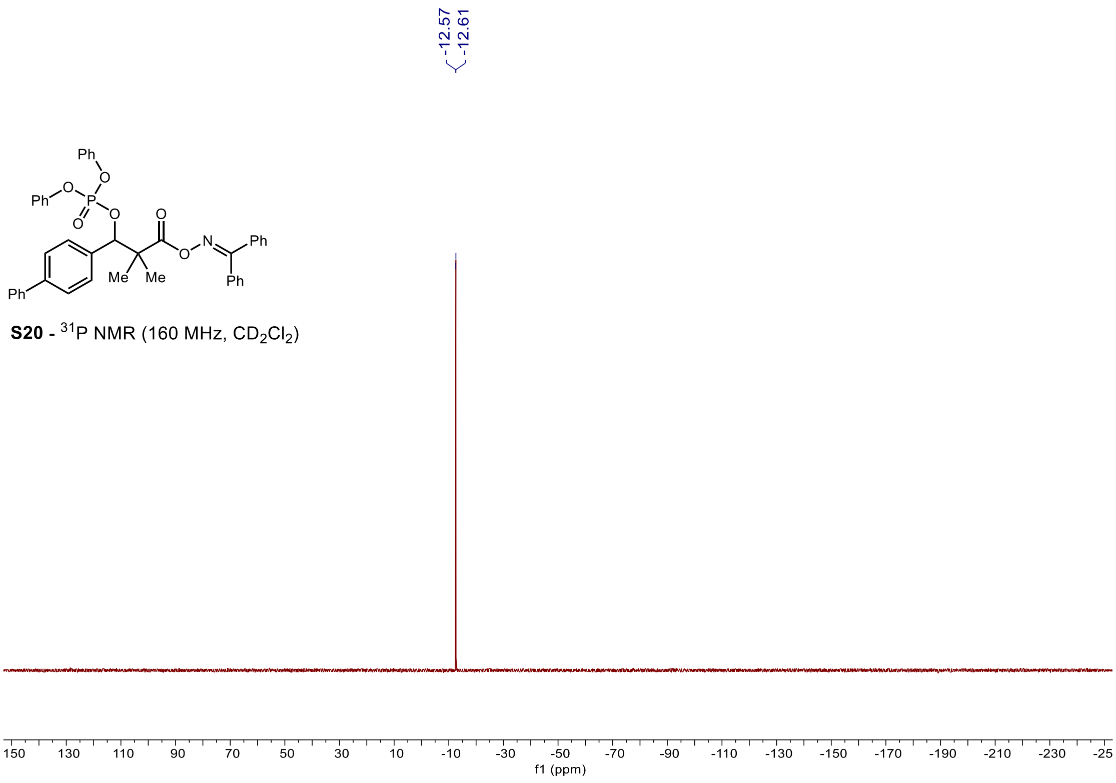

S20 - ^{31}P NMR (160 MHz, CD_2Cl_2)

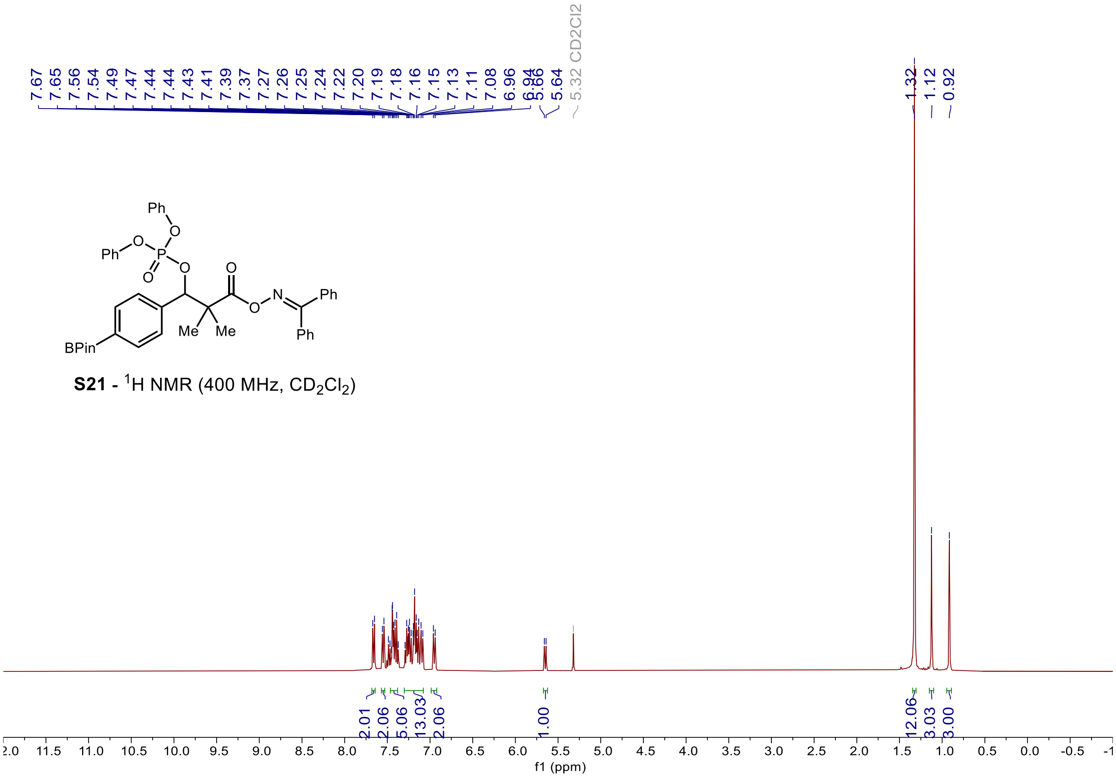

S21 - ^{1}H NMR (400 MHz, CD_2Cl_2)

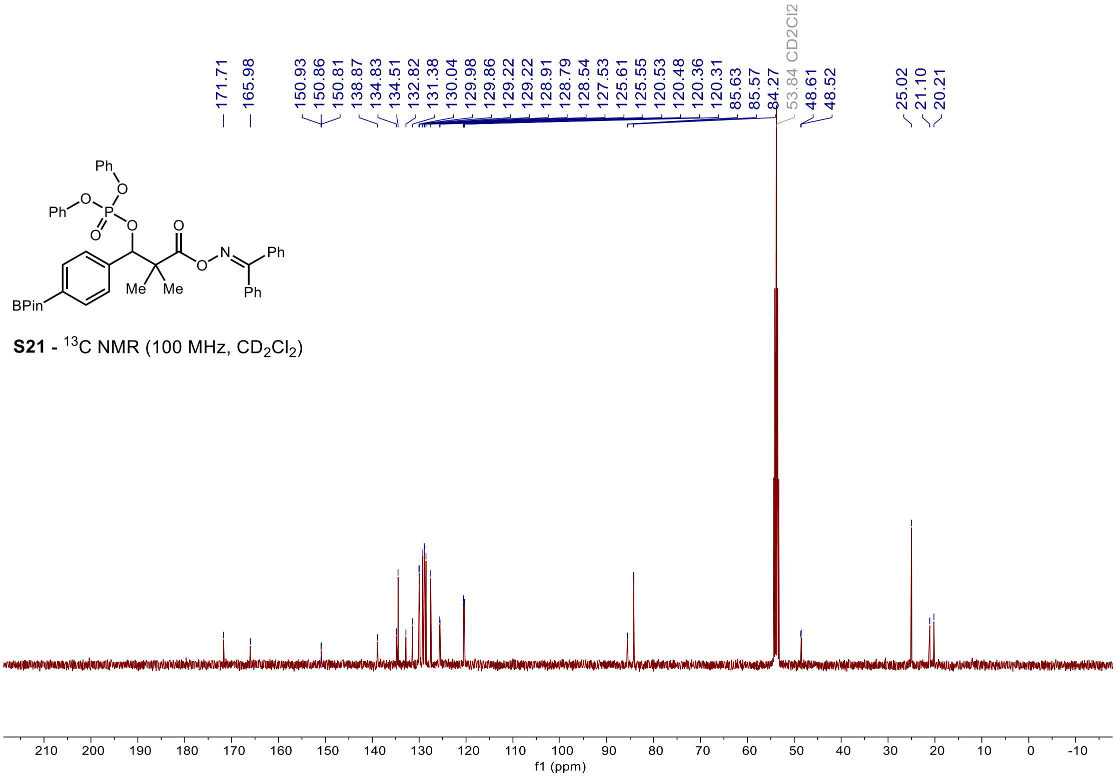

S21 - ^{13}C NMR (100 MHz, CD_2Cl_2)

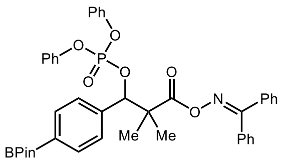

S21 - ^{31}P NMR (160 MHz, CD_2Cl_2)

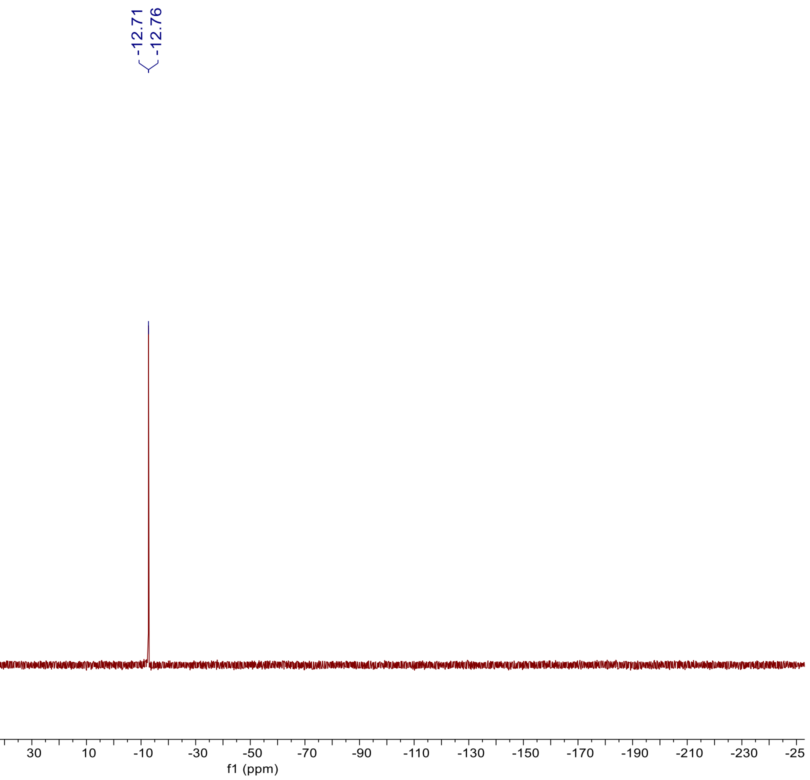

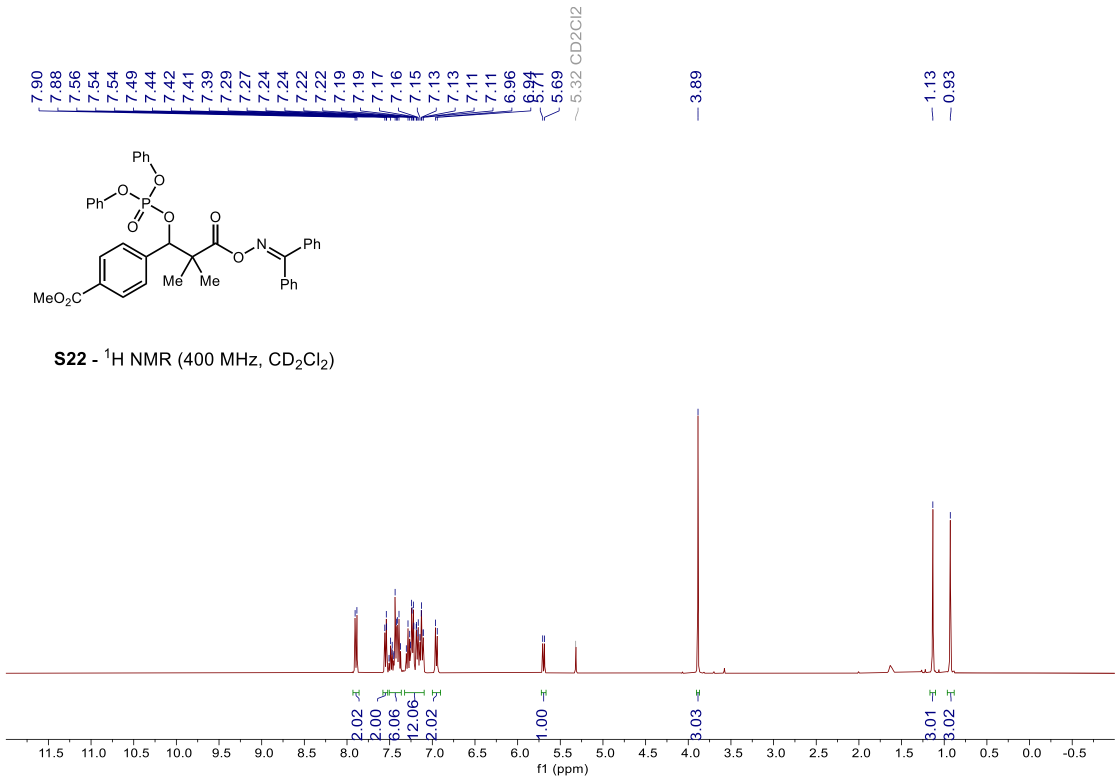

S22 - ^{1}H NMR (400 MHz, CD_2Cl_2)

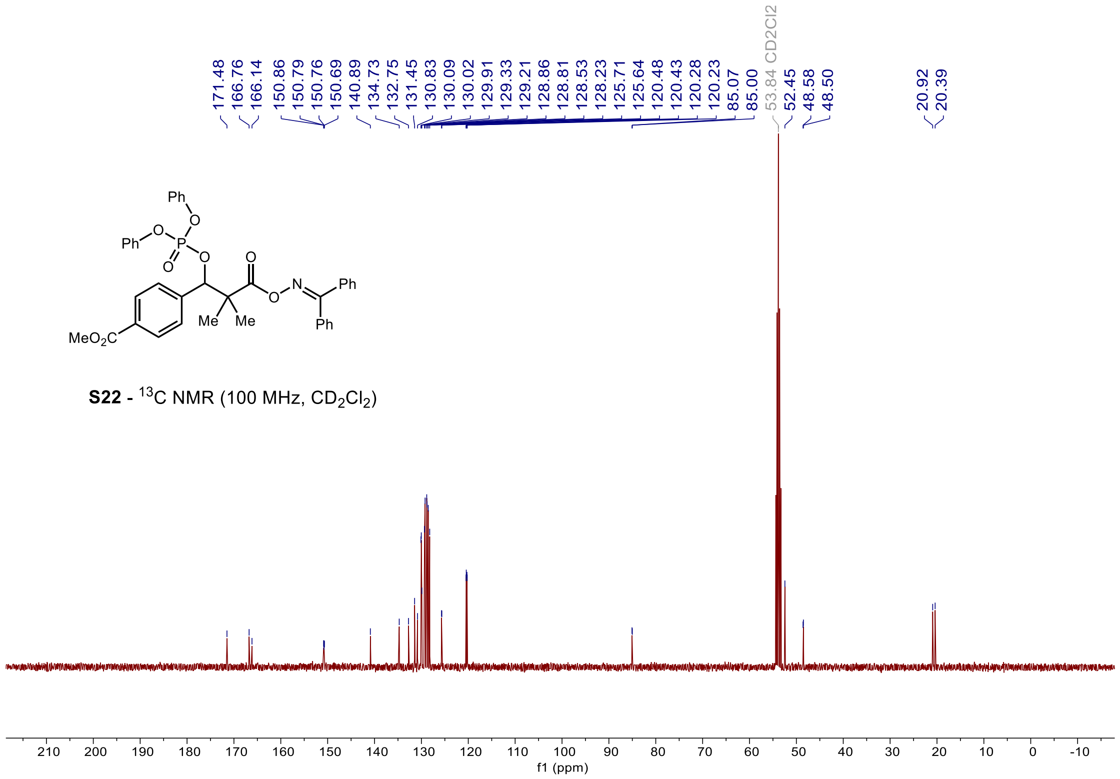

S22 - ^{13}C NMR (100 MHz, CD_2Cl_2)

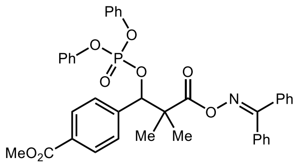

S22 - ^{31}P NMR (160 MHz, CD_2Cl_2)

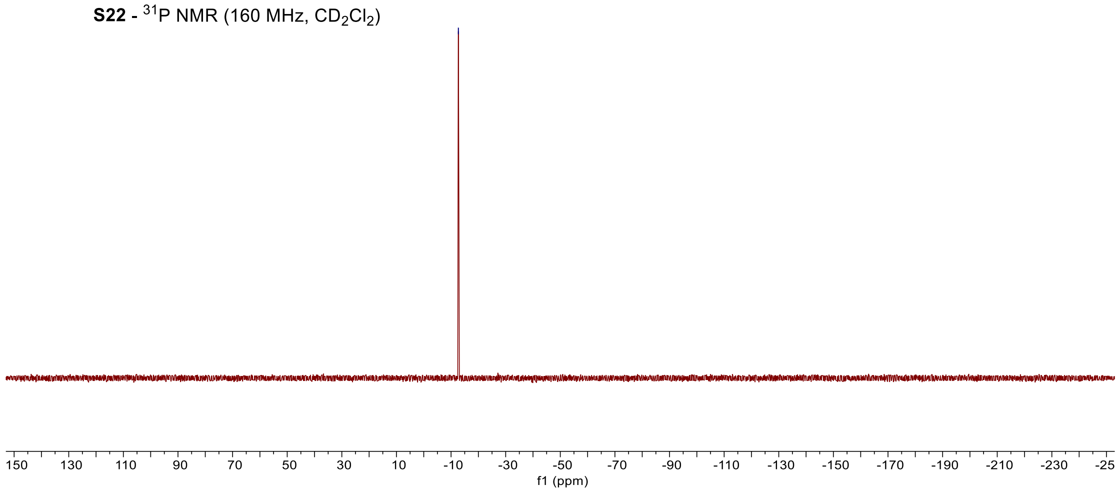

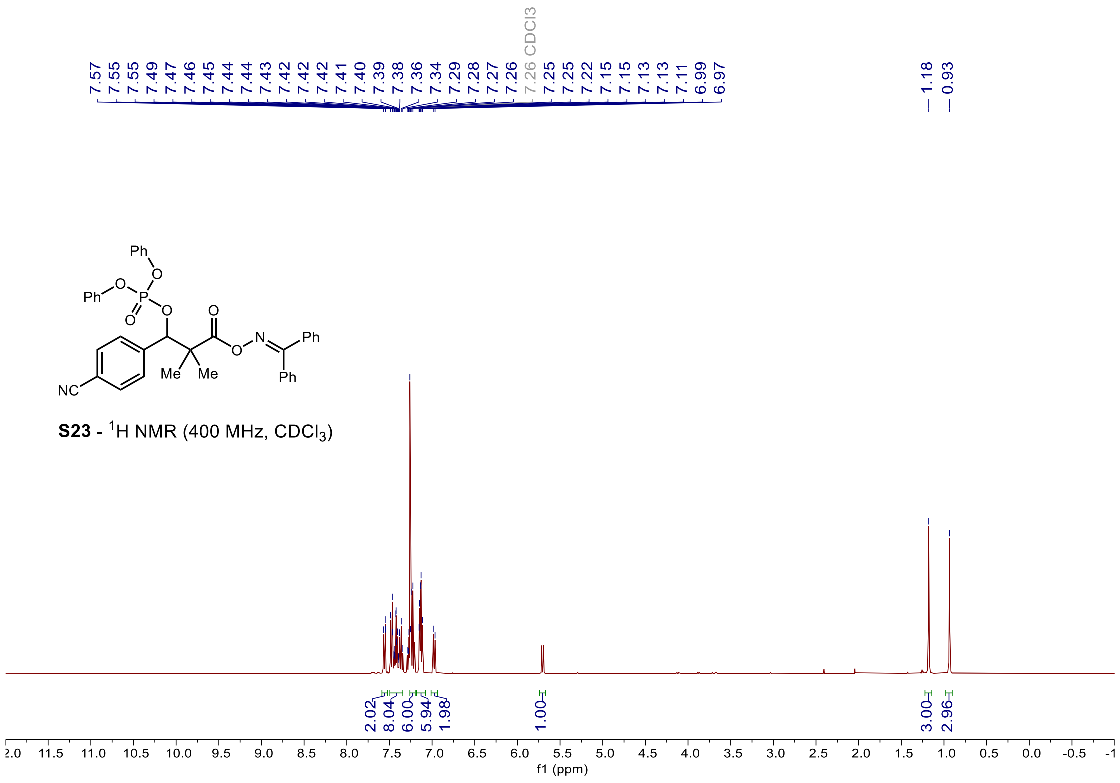

S23 - ^{1}H NMR (400 MHz, $CDCl_3$)

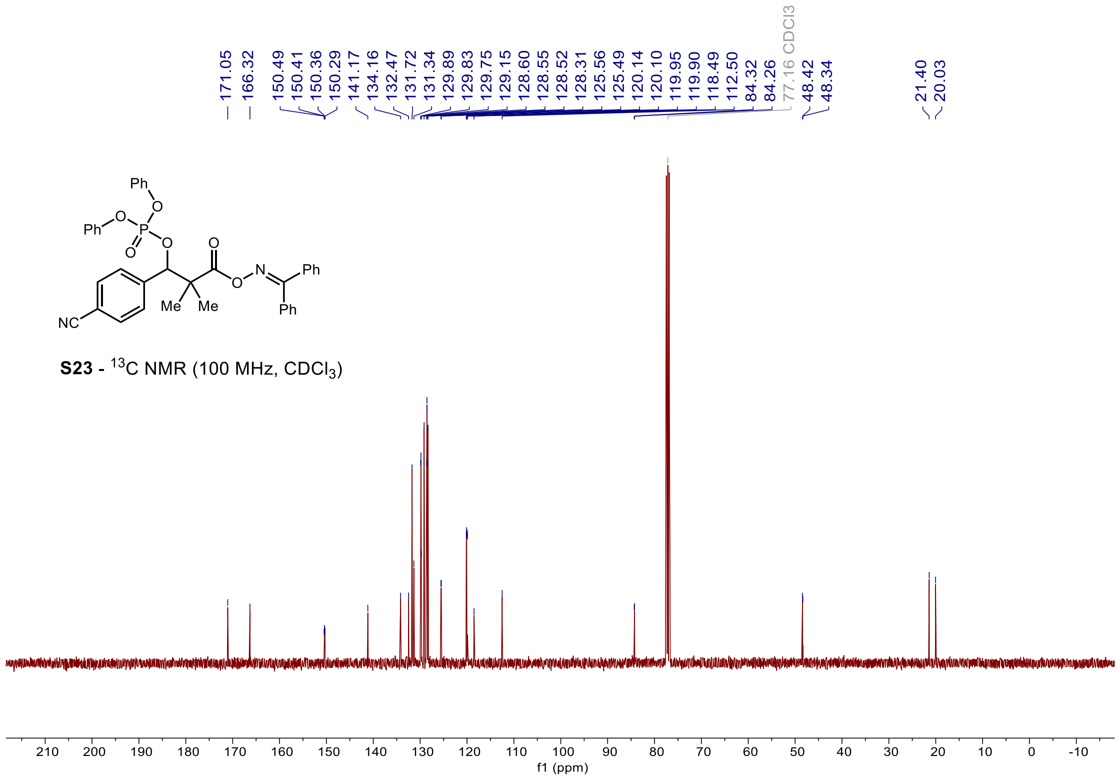

S23 - ^{13}C NMR (100 MHz, $CDCl_3$)

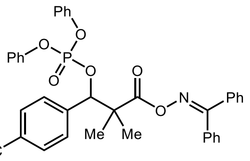

S23 - ^{31}P NMR (160 MHz, $CDCl_3$)

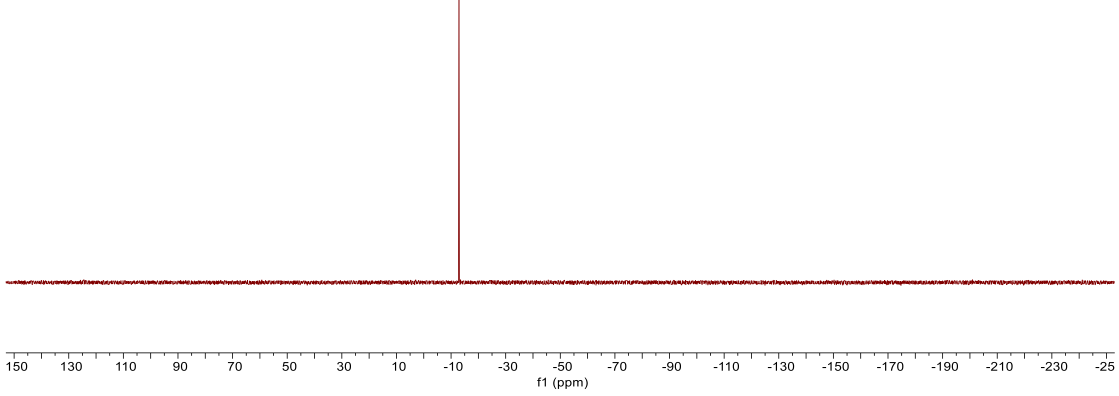

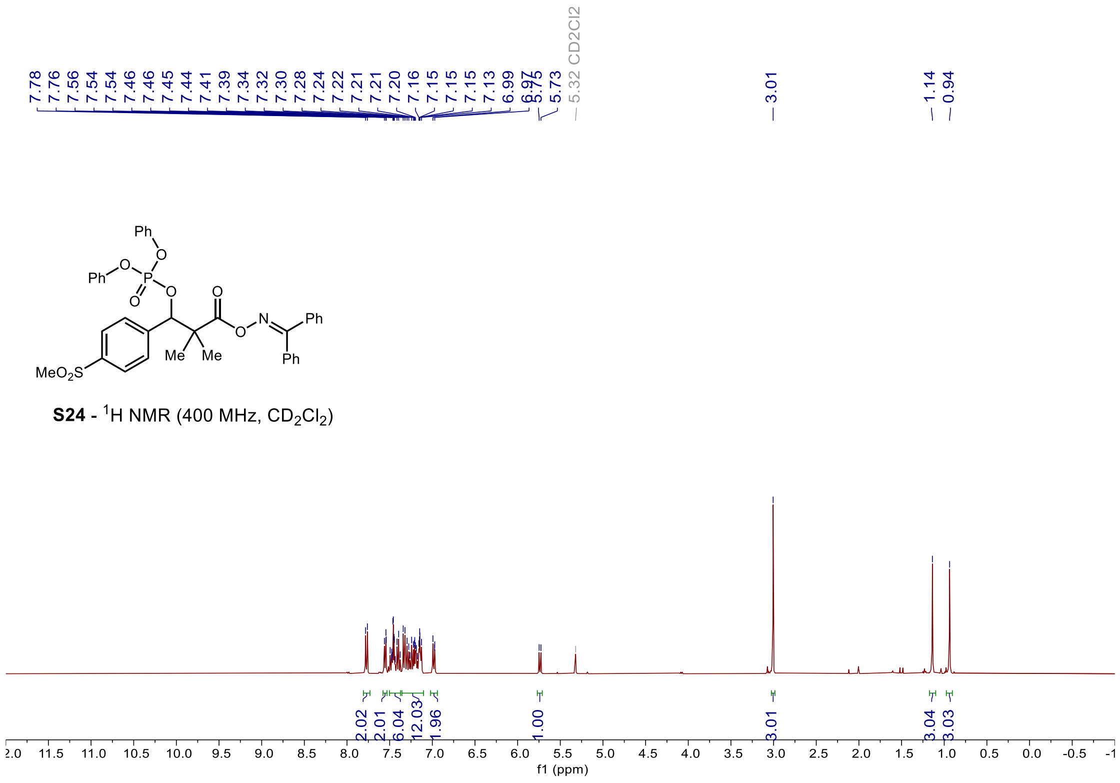

S24 - ^{1}H NMR (400 MHz, CD_2Cl_2)

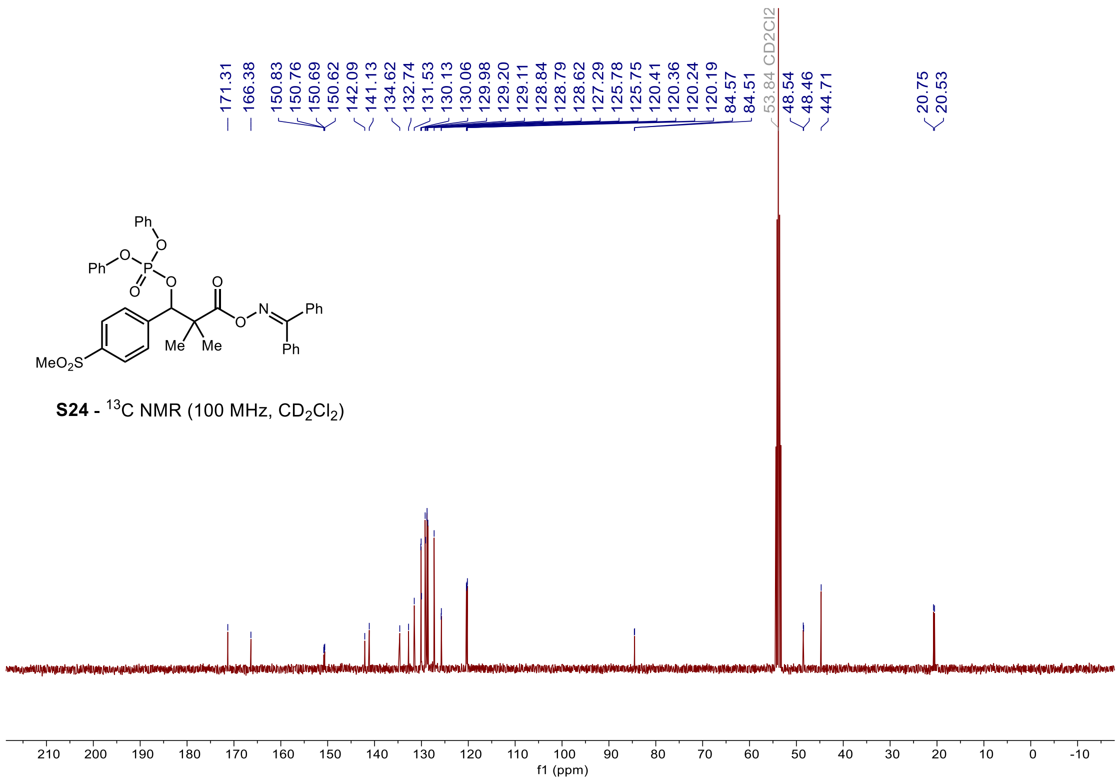

S24 - ^{13}C NMR (100 MHz, CD_2Cl_2)

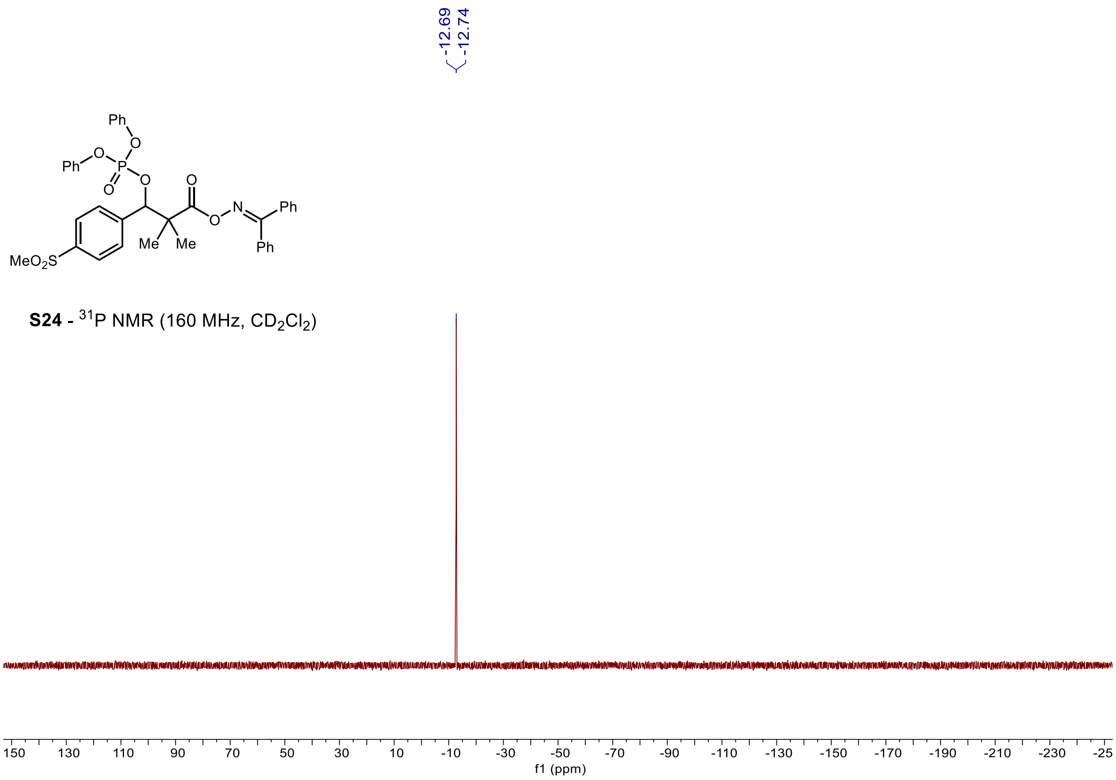

S24 - ^{31}P NMR (160 MHz, CD_2Cl_2)

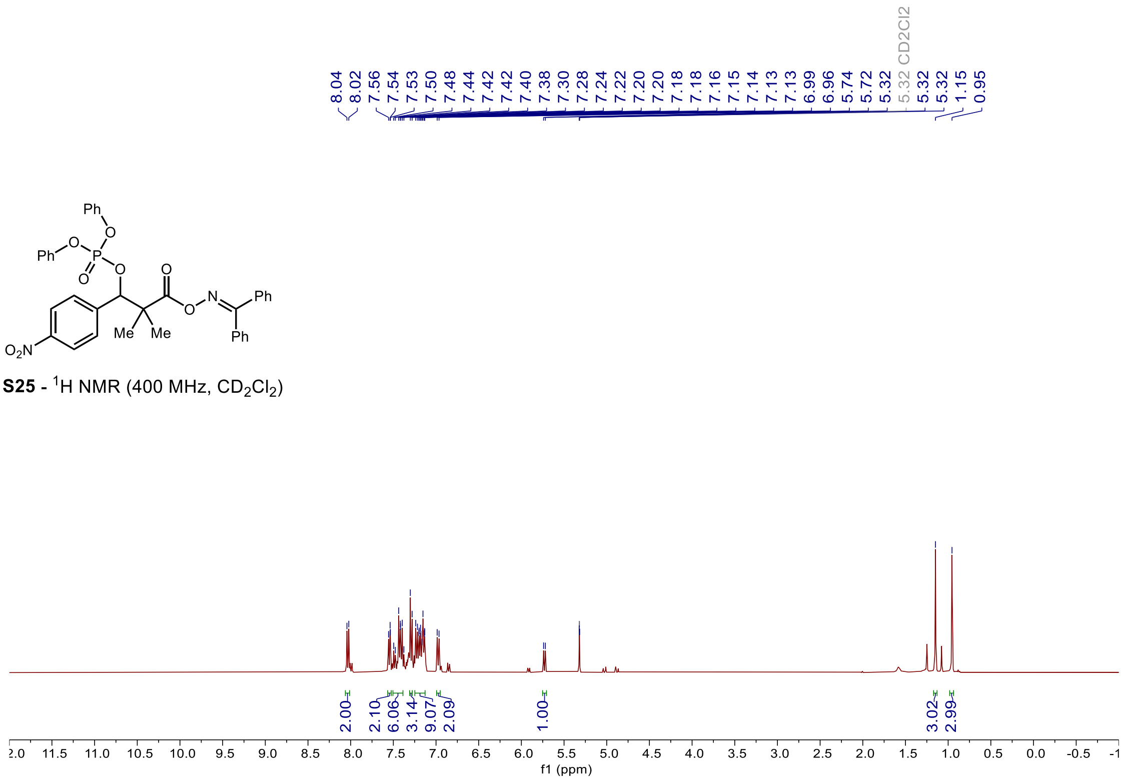

S25 - ^{1}H NMR (400 MHz, CD_2Cl_2)

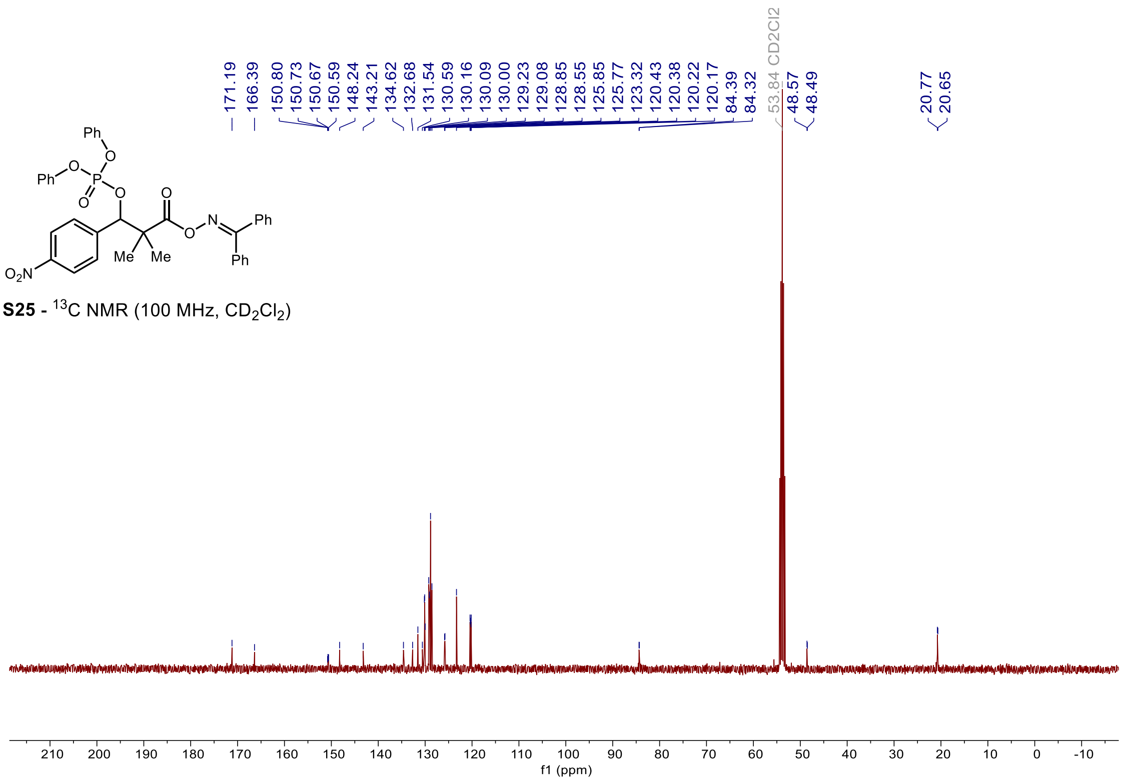

S25 - ^{13}C NMR (100 MHz, CD_2Cl_2)

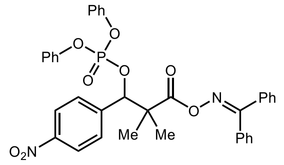

S25 - ^{31}P NMR (160 MHz, CD_2Cl_2)

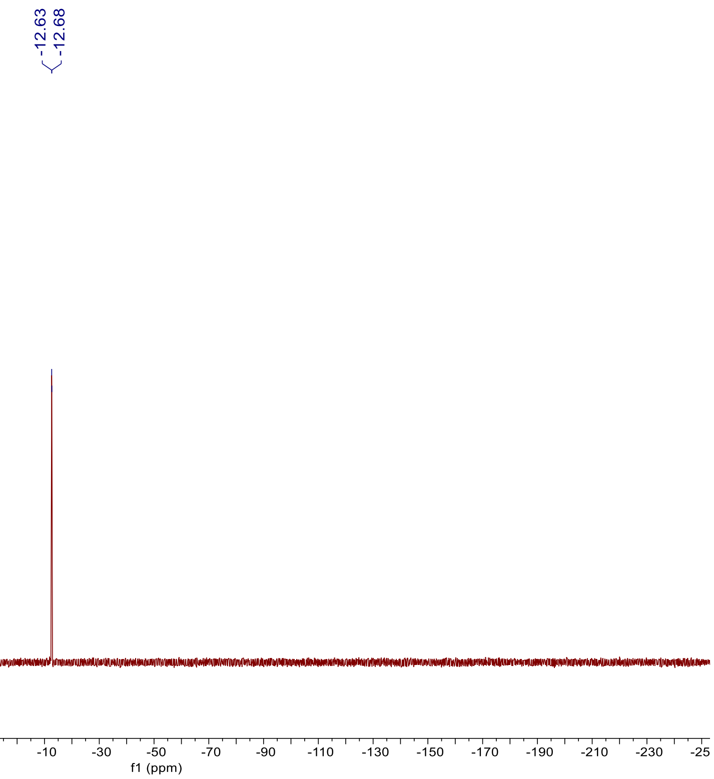

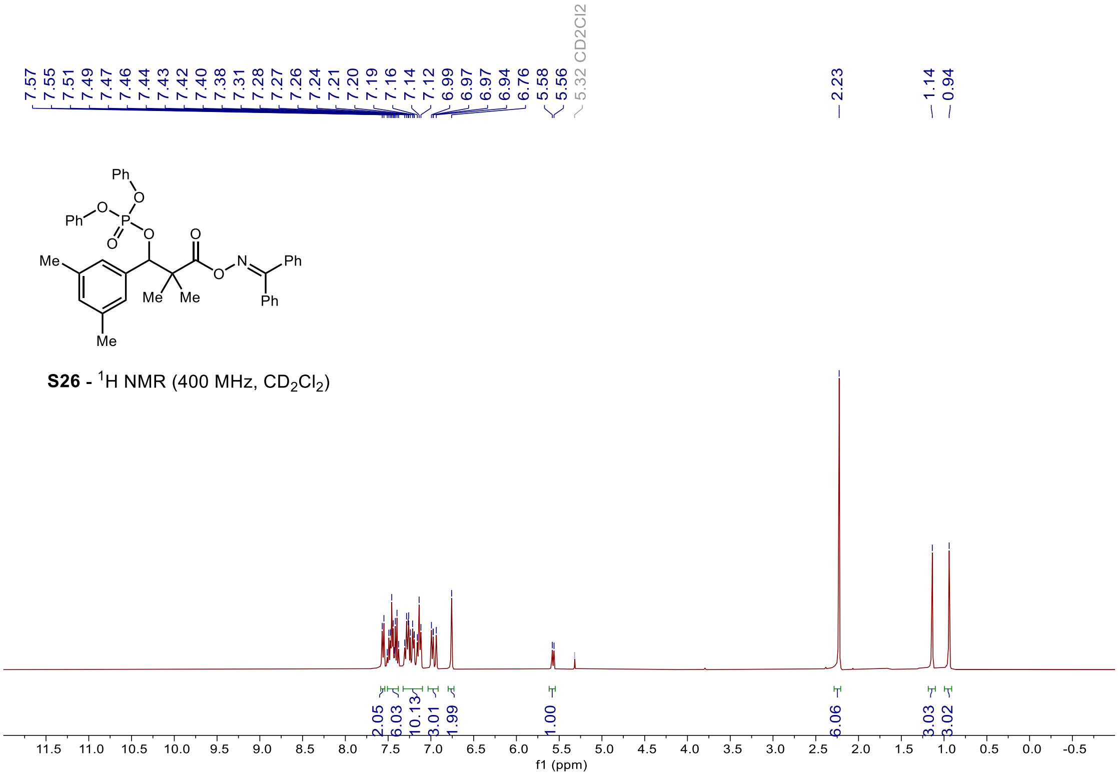

S26 - ^{1}H NMR (400 MHz, CD_2Cl_2)

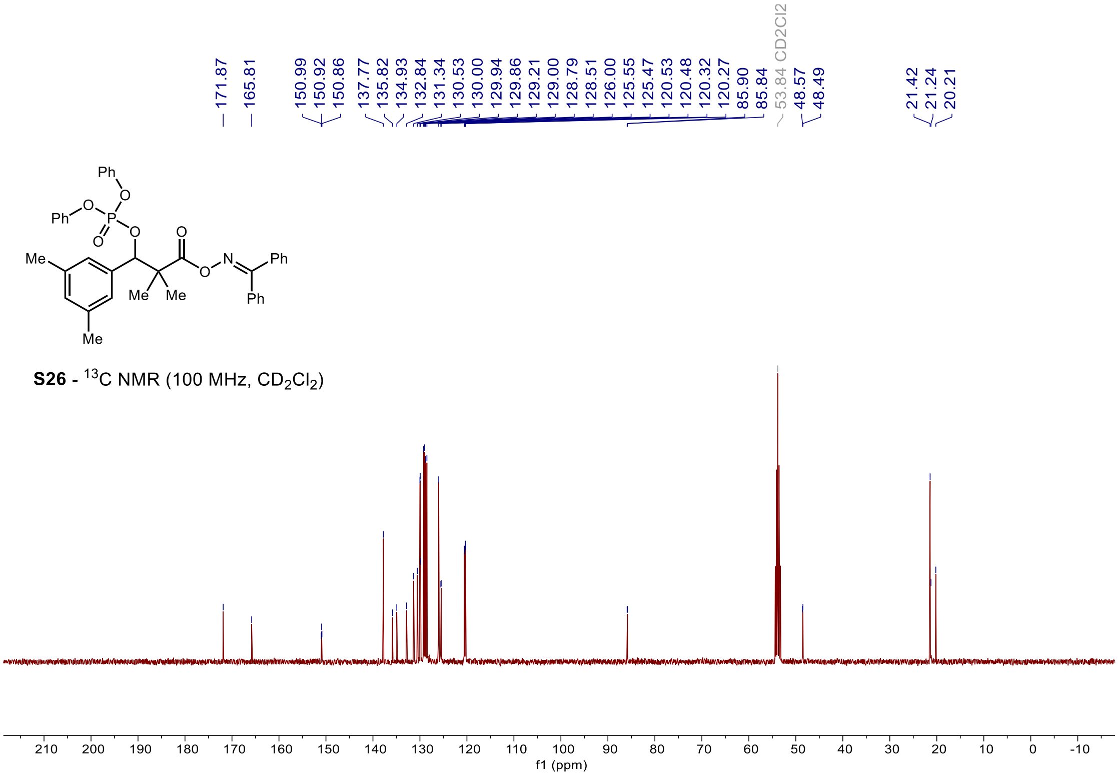

S26 - ^{13}C NMR (100 MHz, CD_2Cl_2)

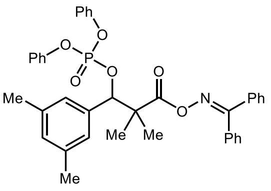

S26 - ^{31}P NMR (160 MHz, CD_2Cl_2)

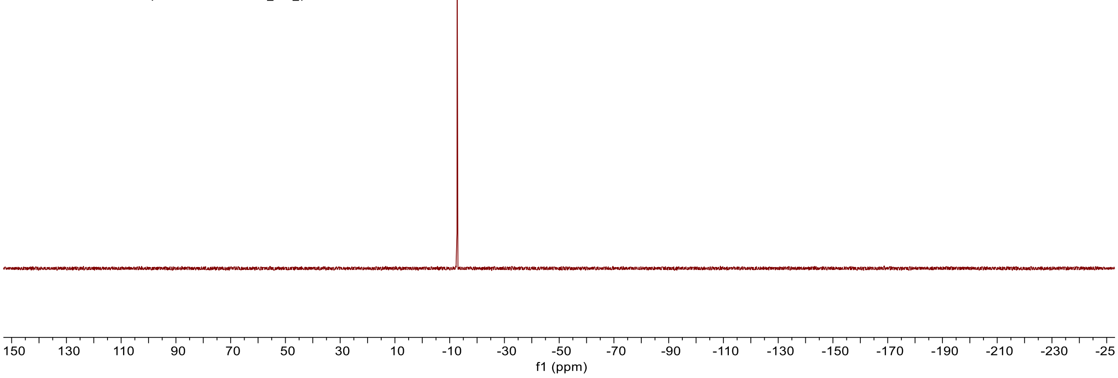

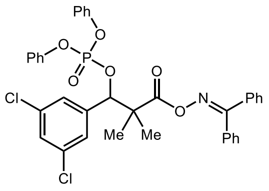

S27 - ^{1}H NMR (400 MHz, CD_2Cl_2)

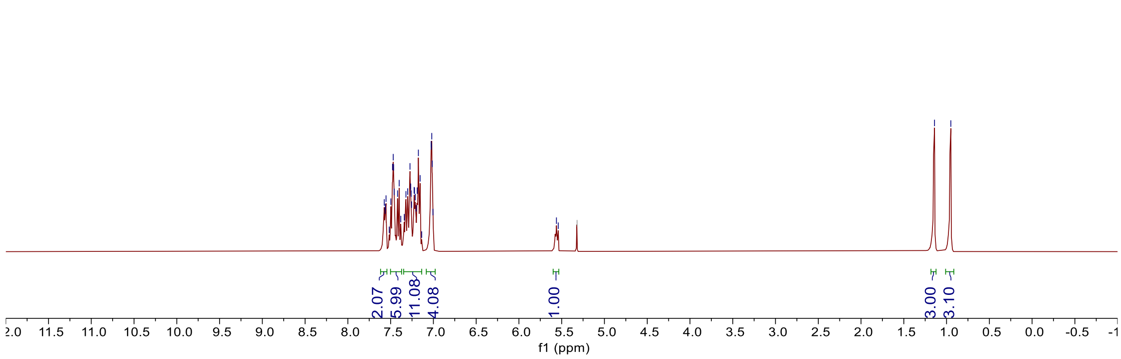

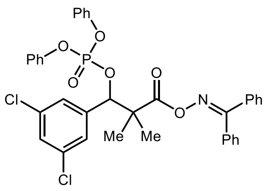

S27 - ^{13}C NMR (100 MHz, CD_2Cl_2)

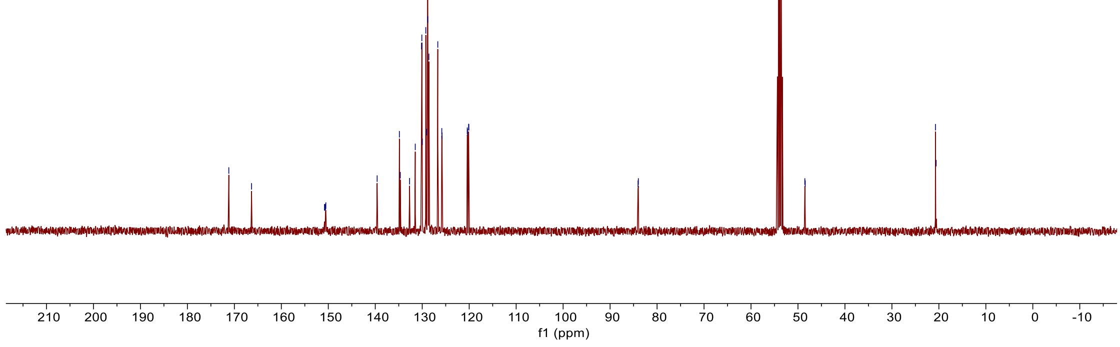

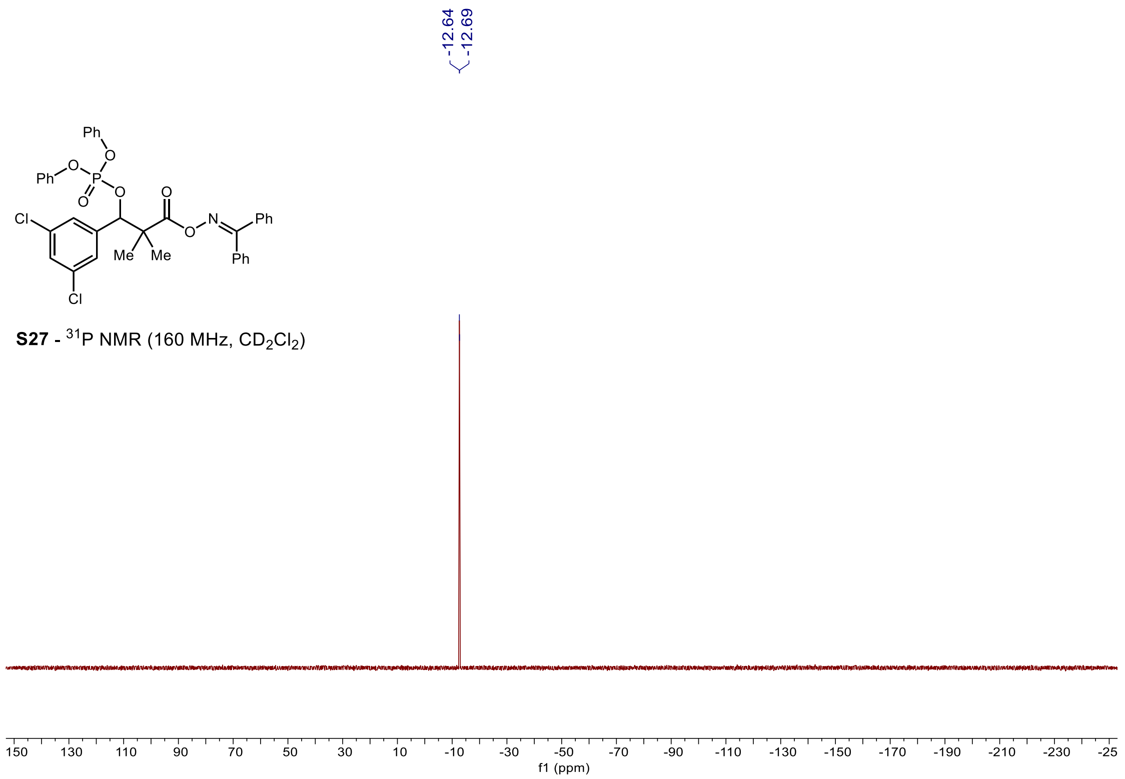

S27 - ^{31}P NMR (160 MHz, CD_2Cl_2)

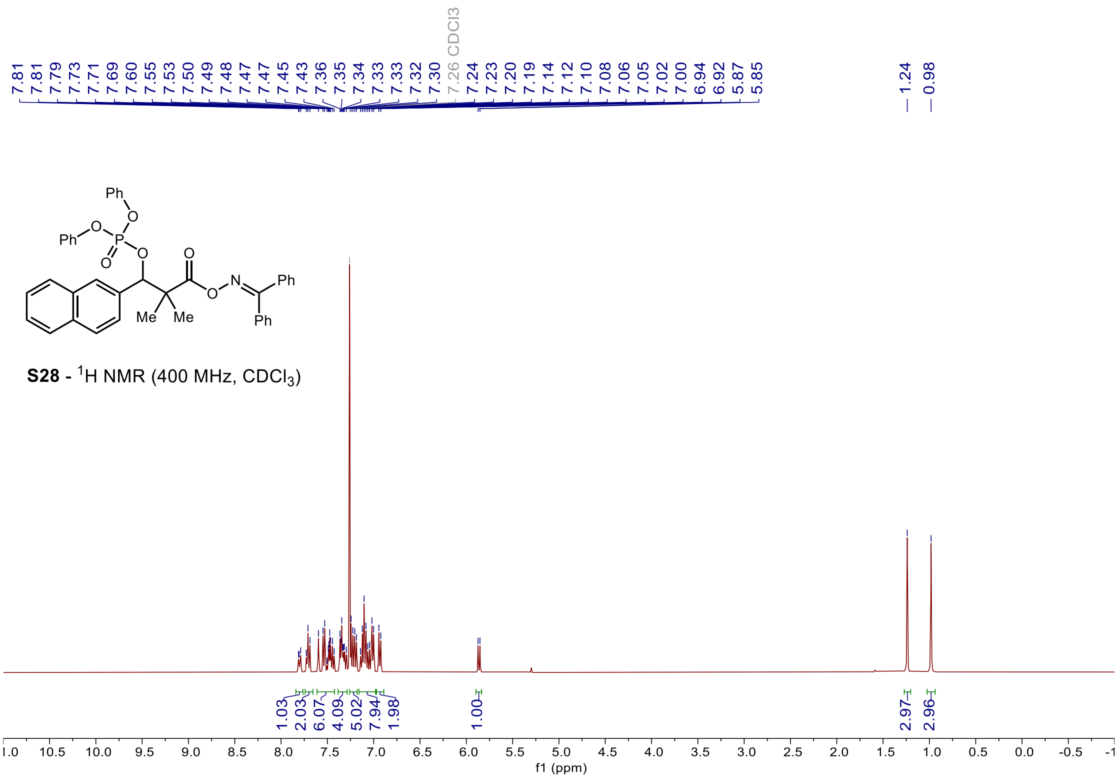

S28 - ^{1}H NMR (400 MHz, $CDCl_3$)

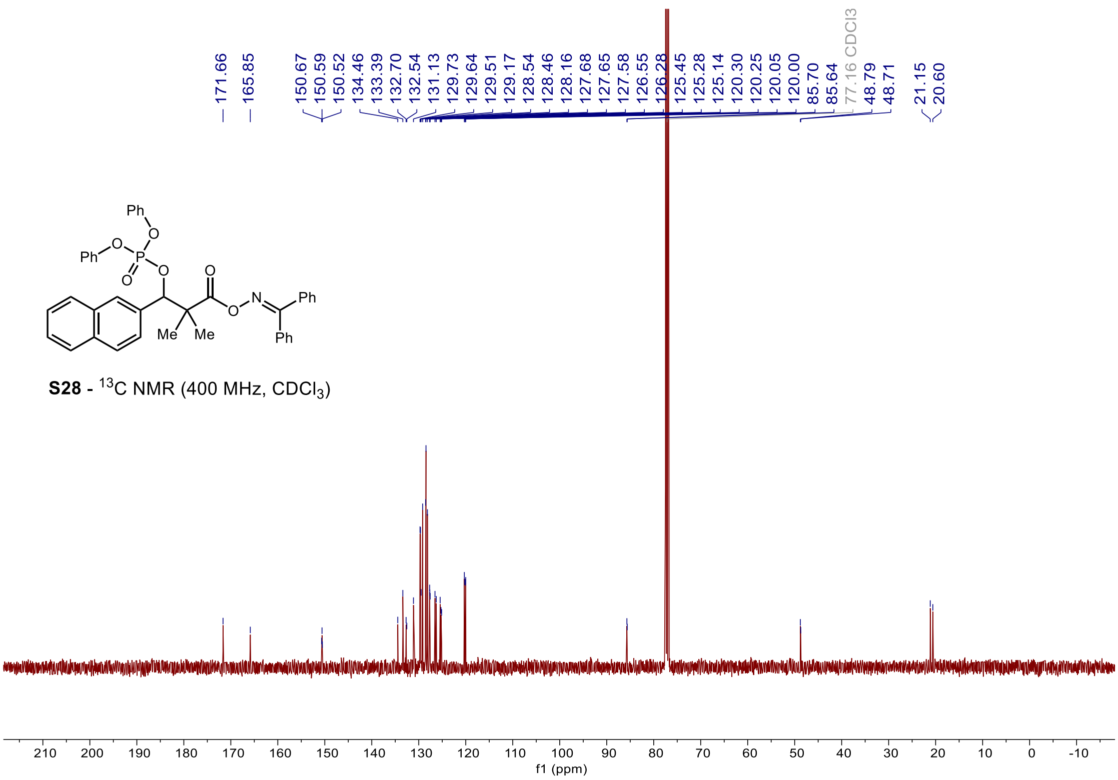

S28 - ^{13}C NMR (400 MHz, $CDCl_3$)

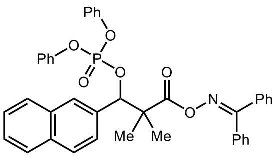

S28 - ^{31}P NMR (160 MHz, $CDCl_3$)

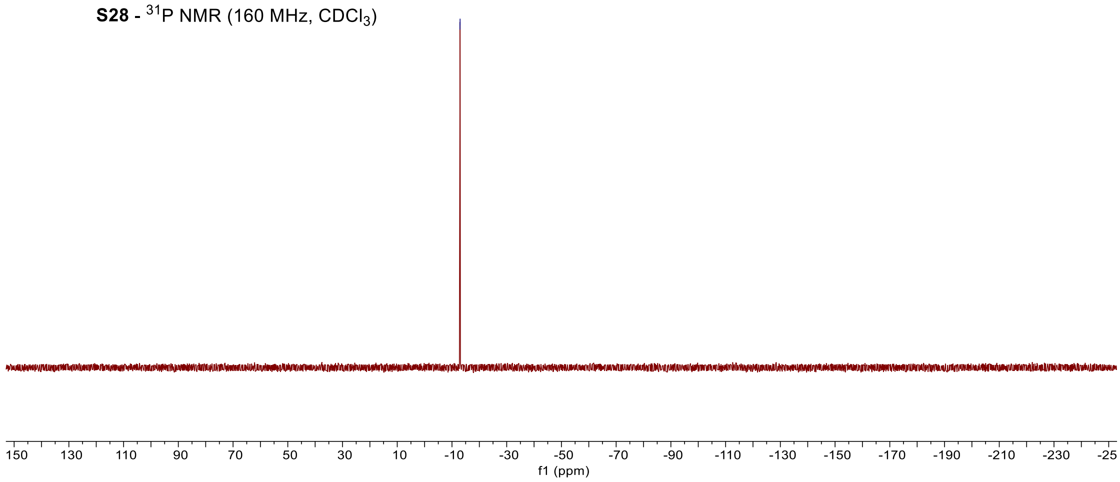

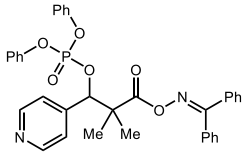

S29 - ^{1}H NMR (400 MHz, CD_2Cl_2)

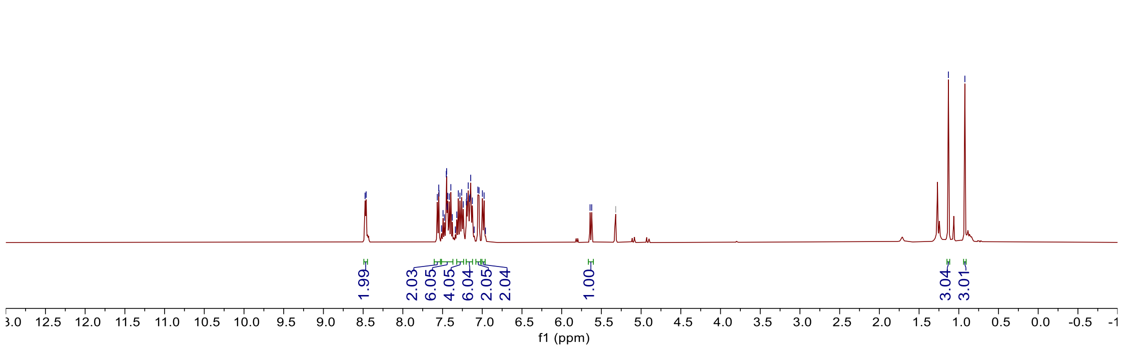

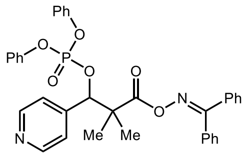

S29 - ^{13}C NMR (100 MHz, CD_2Cl_2)

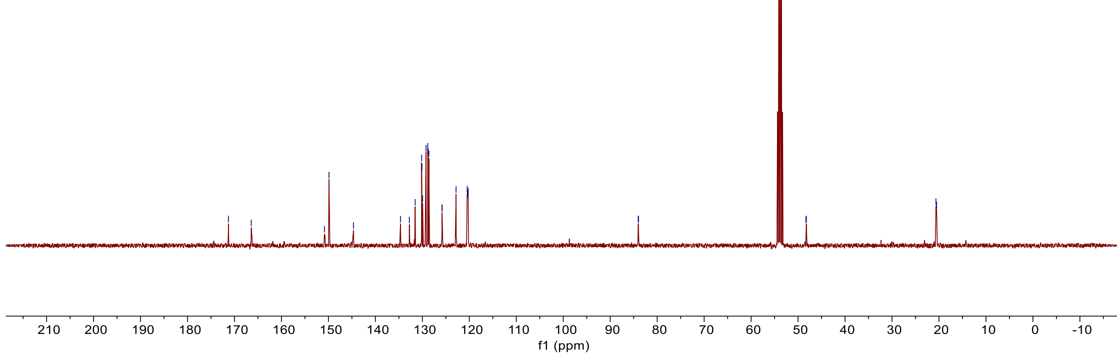

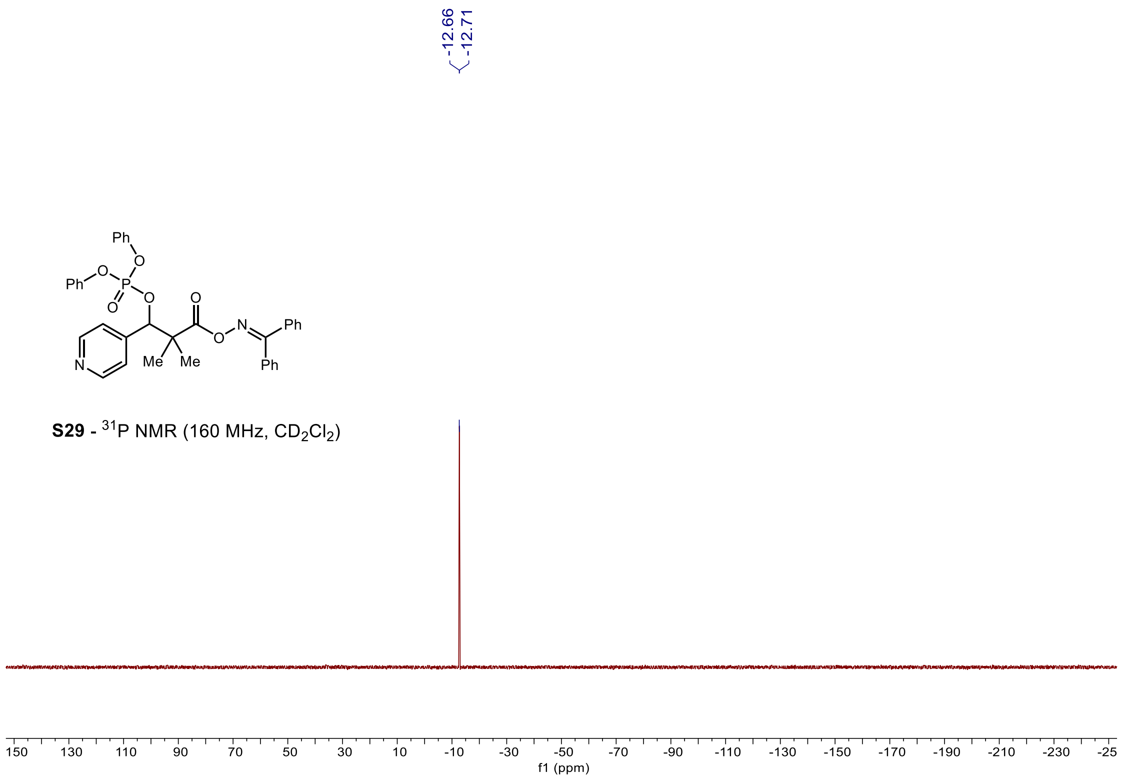

S29 - ^{31}P NMR (160 MHz, CD_2Cl_2)

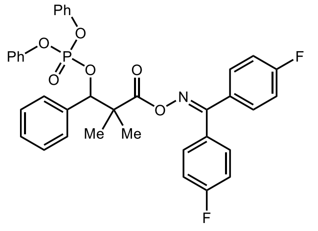

S30 - ^{1}H NMR (400 MHz, CD_2Cl_2)

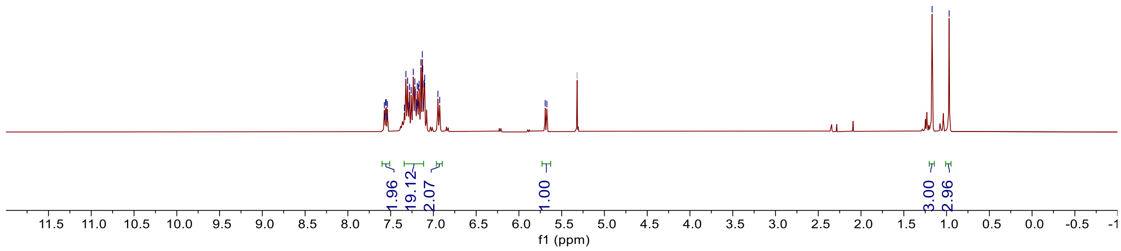

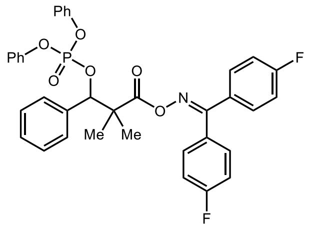

S30 - ^{13}C NMR (100 MHz, CD_2Cl_2)

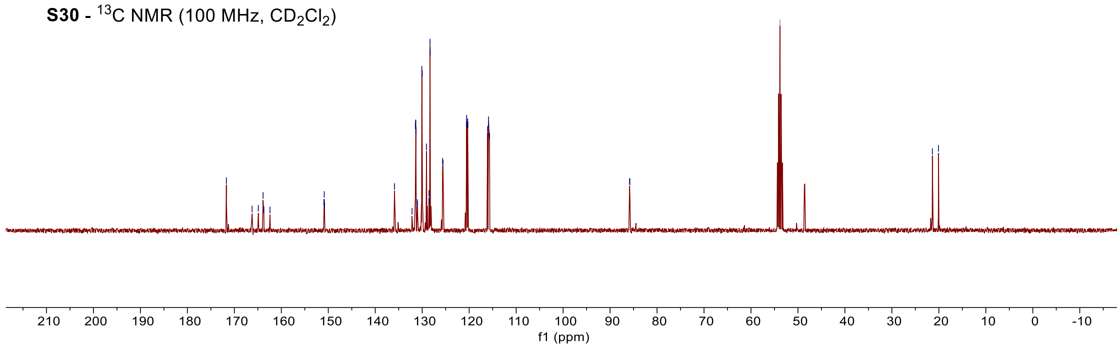

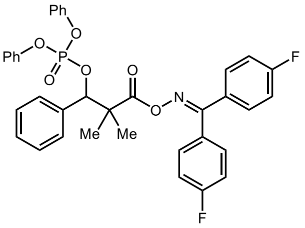

S30 - ^{31}P NMR (160 MHz, CD_2Cl_2)

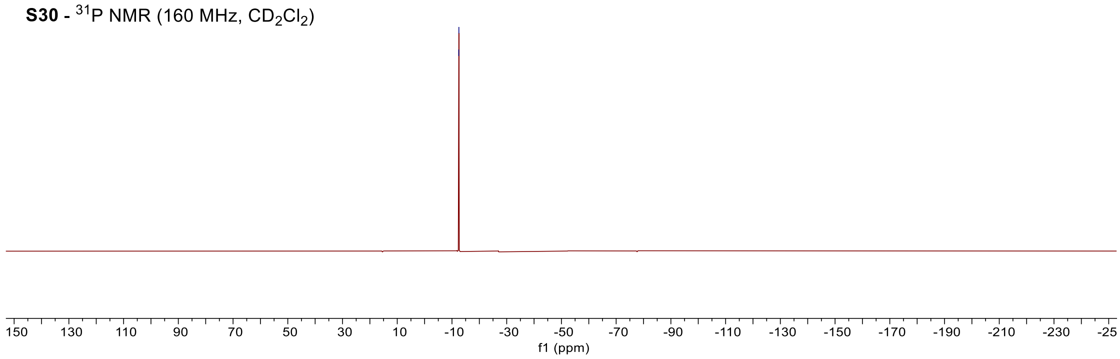

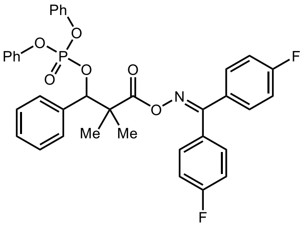

S30 - ^{19}F NMR (376 MHz, CD_2Cl_2)

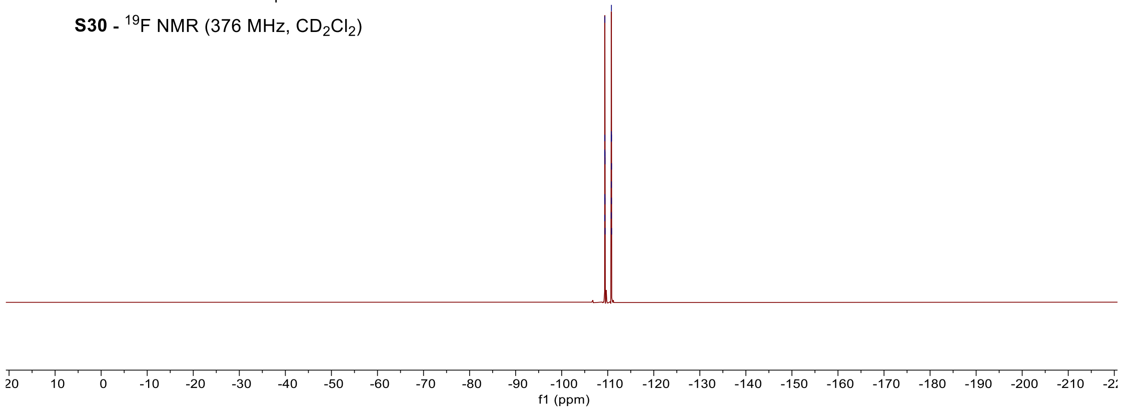

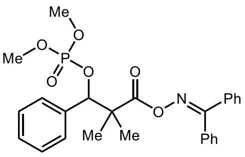

S31 - ^{1}H NMR (400 MHz, CD_2Cl_2)

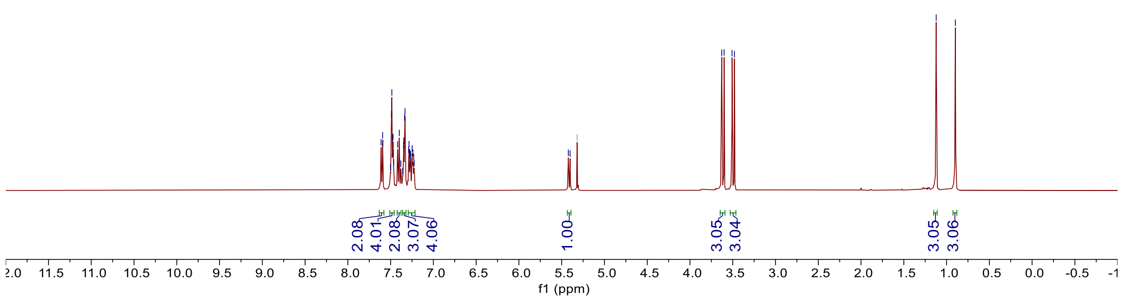

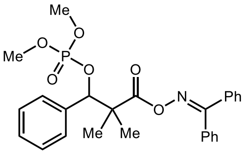

S31 - ^{13}C NMR (100 MHz, CD_2Cl_2)

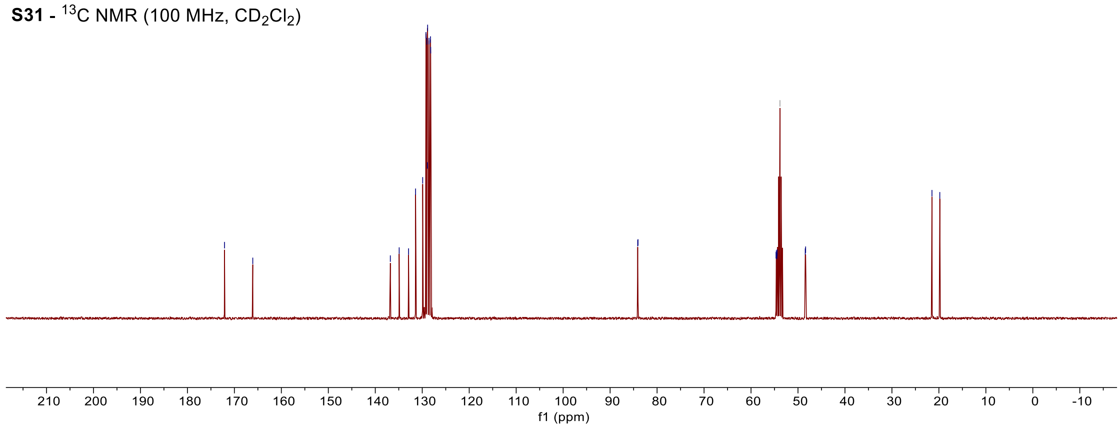

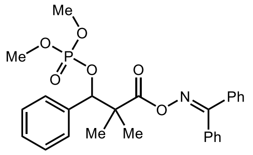

S31 - ^{31}P NMR (160 MHz, CD_2Cl_2)

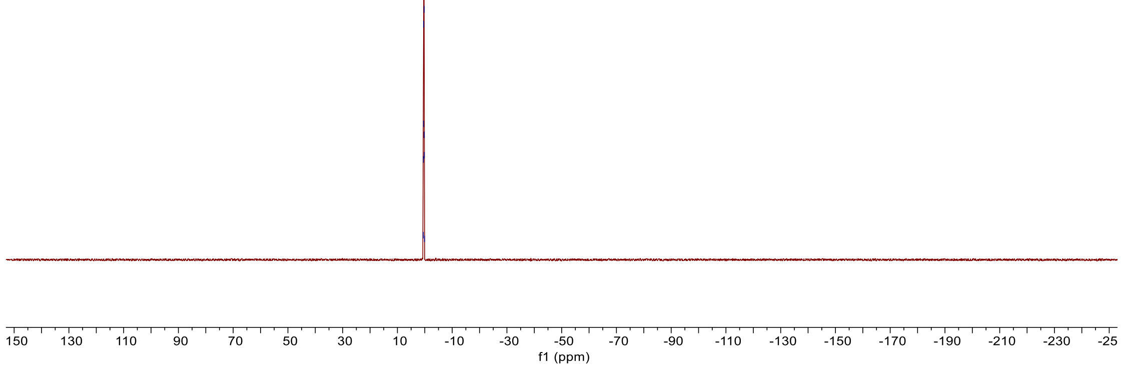

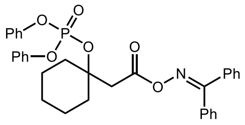

S32 - ^{1}H NMR (400 MHz, CD_2Cl_2)

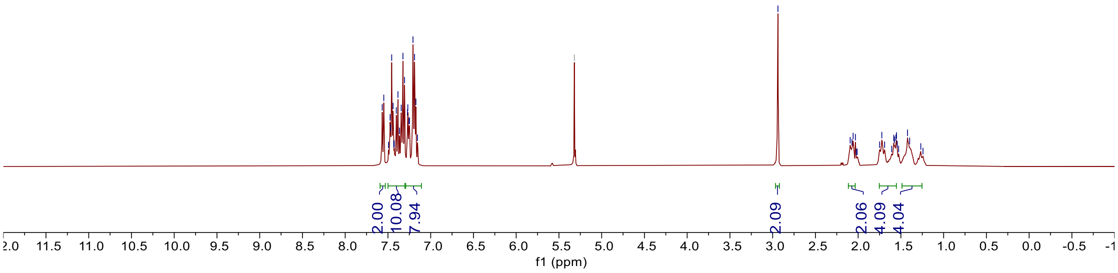

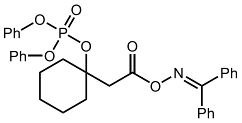

S32 - ^{13}C NMR (100 MHz, CD_2Cl_2)

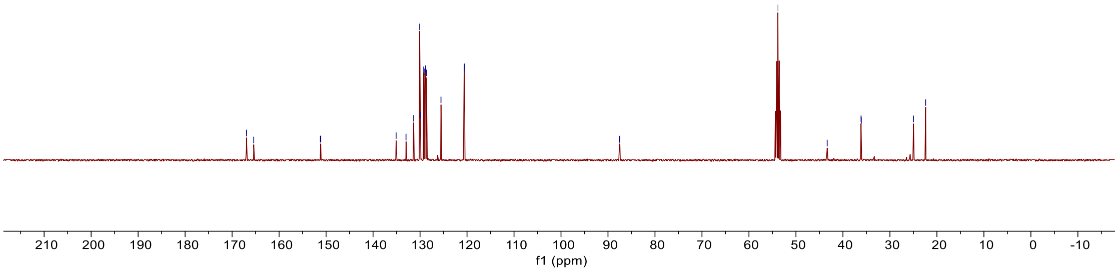

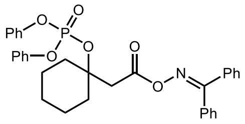

S32 - ^{31}P NMR (160 MHz, CD_2Cl_2)

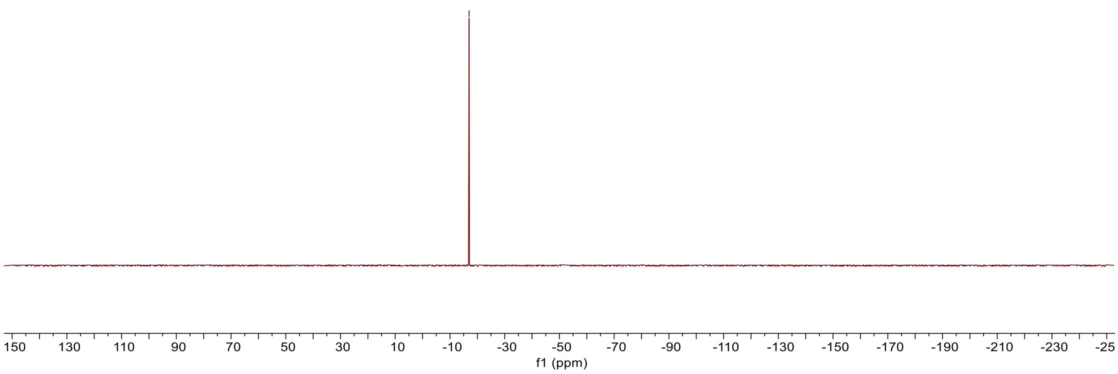

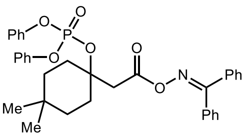

S33 - ^{1}H NMR (400 MHz, CD_2Cl_2)

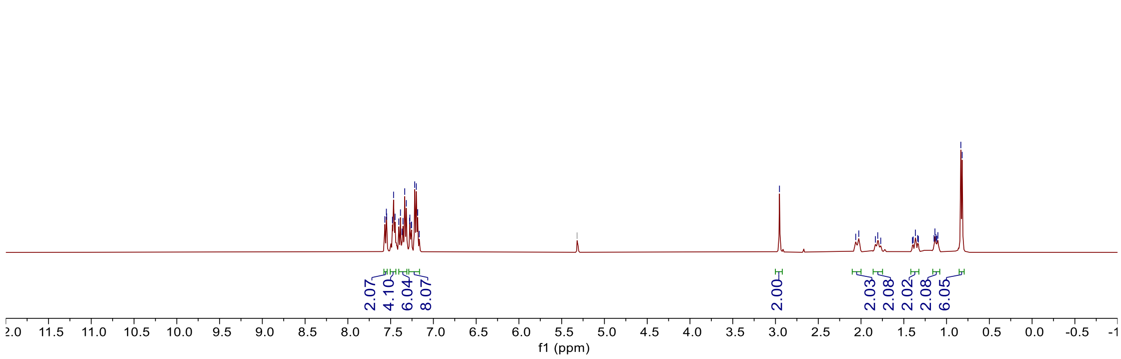

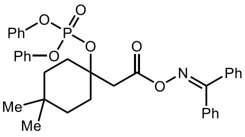

S33 - ^{13}C NMR (100 MHz, CD_2Cl_2)

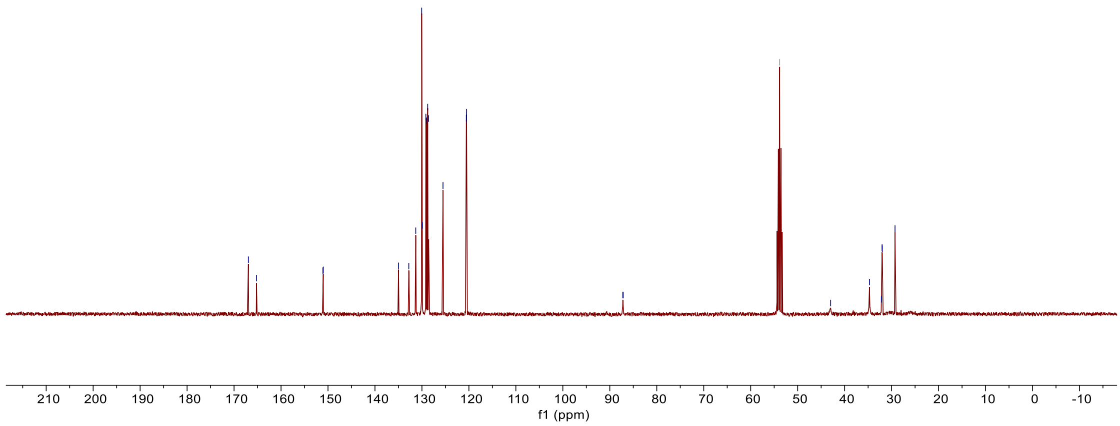

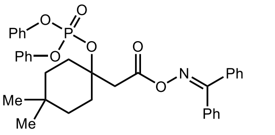

S33 - ^{31}P NMR (160 MHz, CD_2Cl_2)

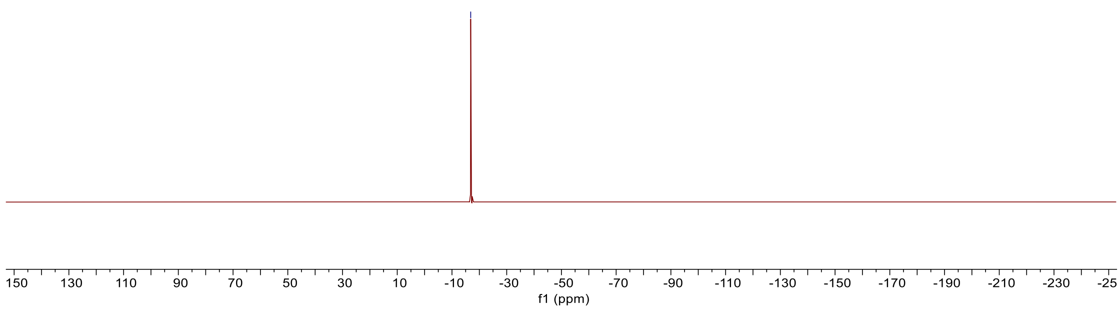

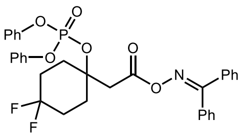

S34 - ^{1}H NMR (400 MHz, CD_2Cl_2)

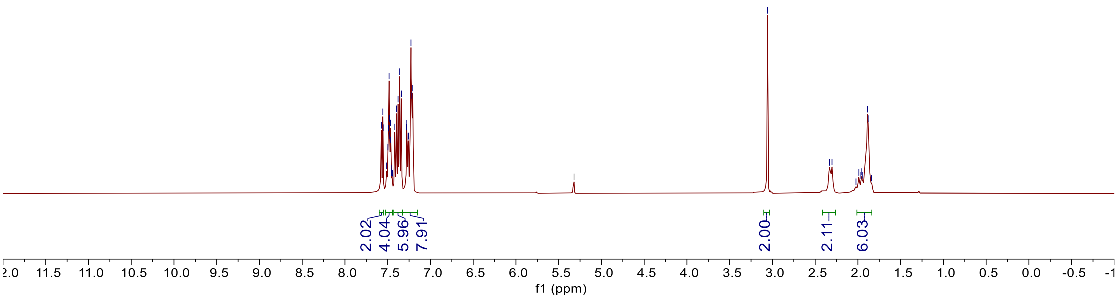

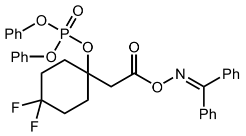

S34 - ^{13}C NMR (100 MHz, CD_2Cl_2)

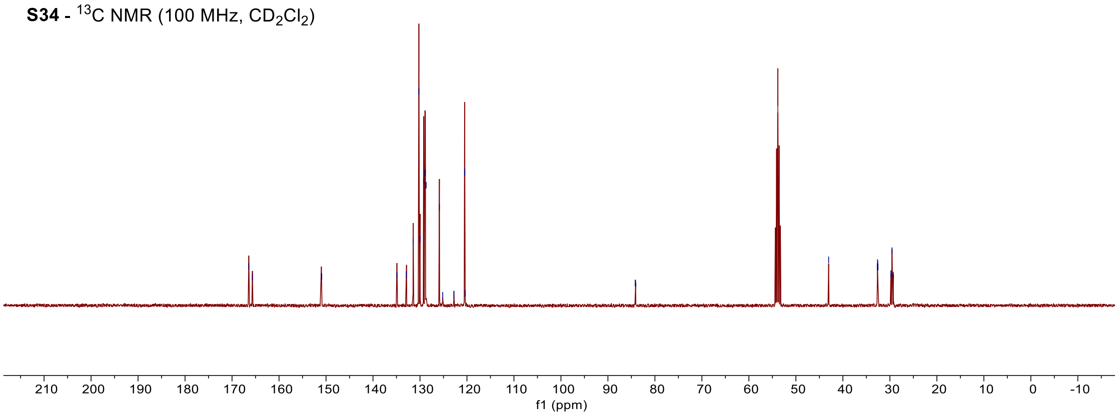

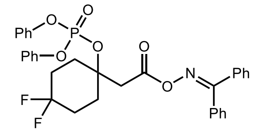

S34 - ^{31}P NMR (160 MHz, CD_2Cl_2)

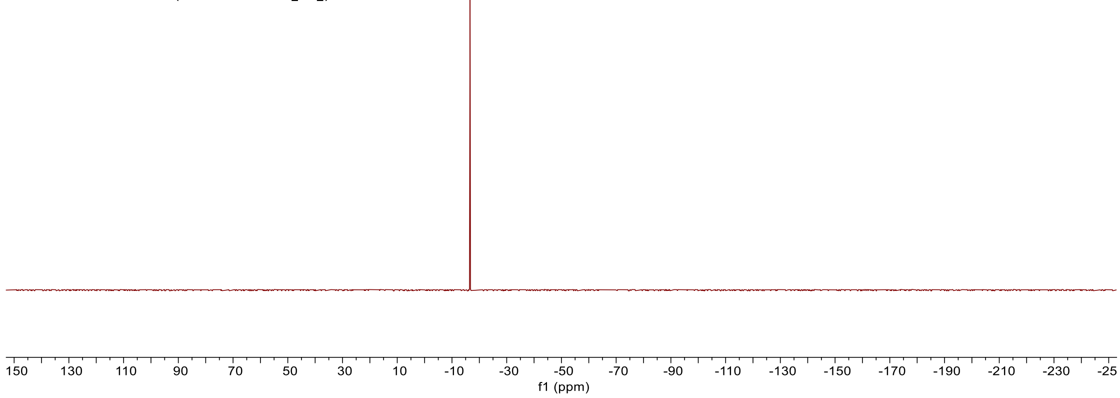

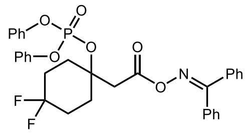

S34 - ^{31}P NMR (160 MHz, CD_2Cl_2)

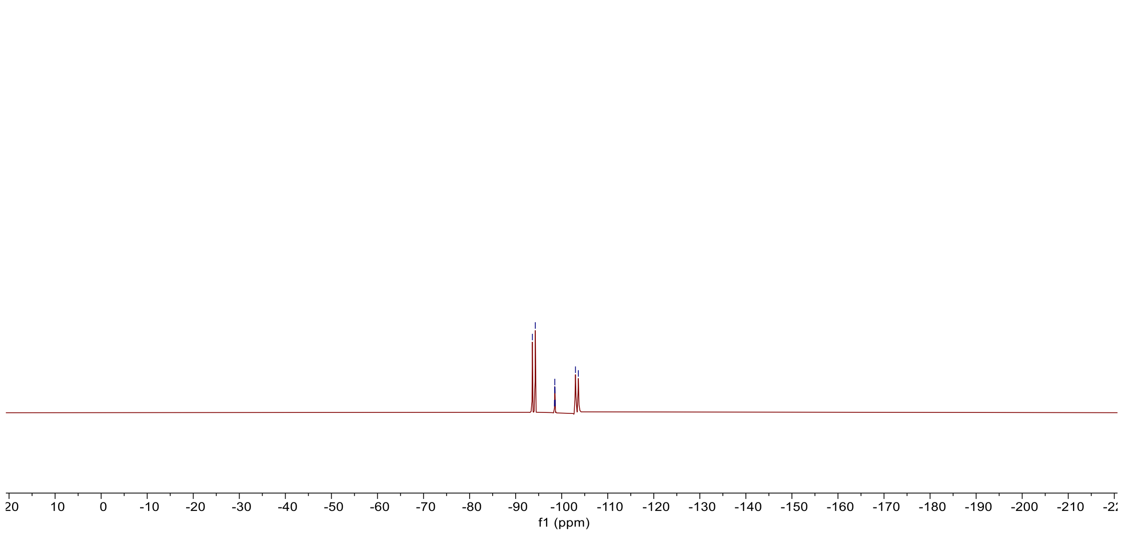

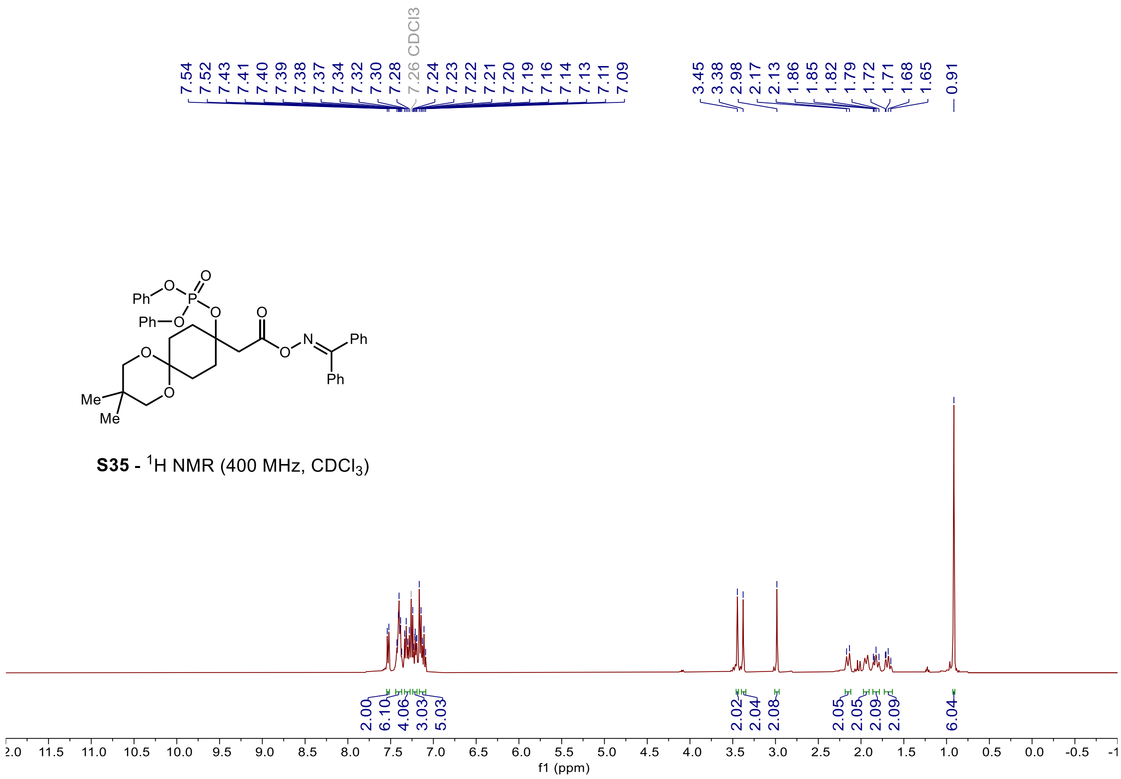

S35 - ^{1}H NMR (400 MHz, $CDCl_3$)

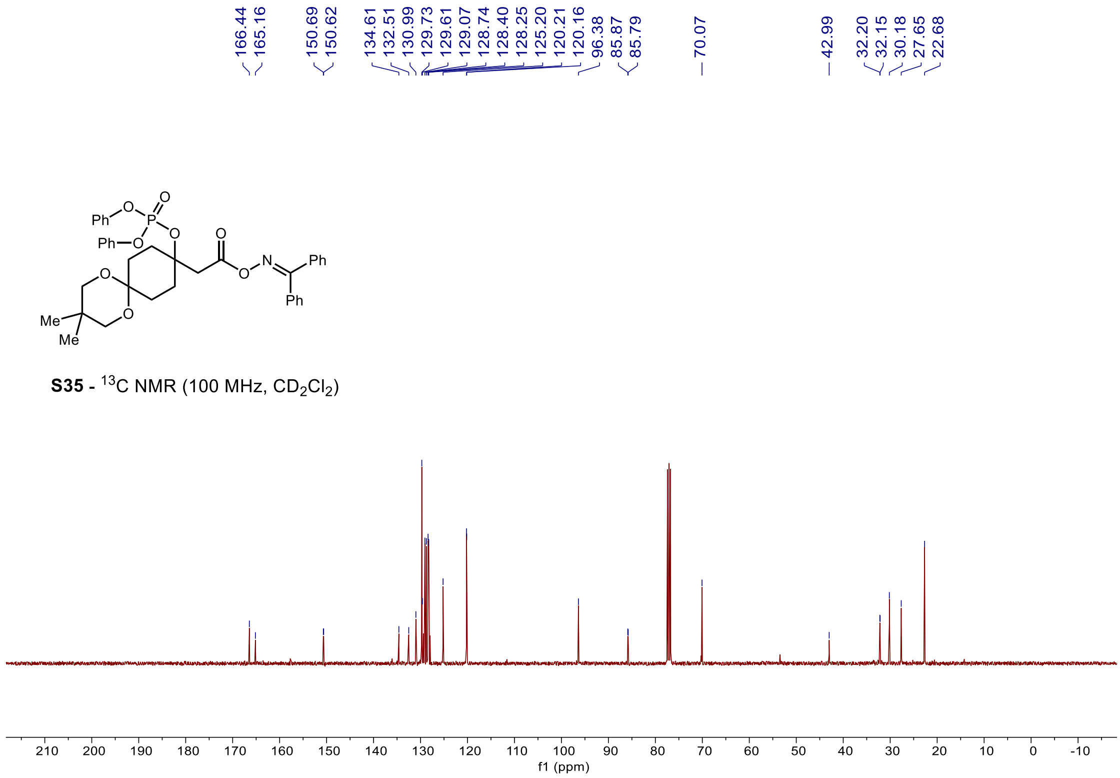

S35 - ^{13}C NMR (100 MHz, CD_2Cl_2)

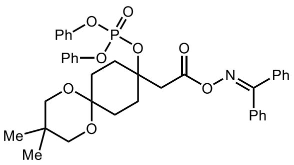

S35 - ^{31}P NMR (160 MHz, $CDCl_3$)

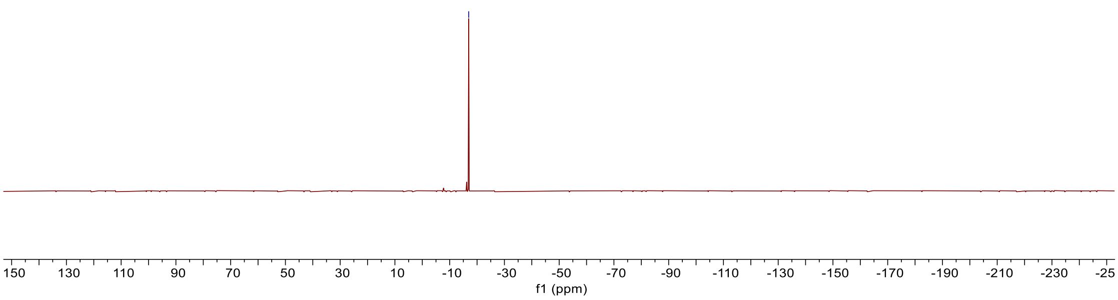

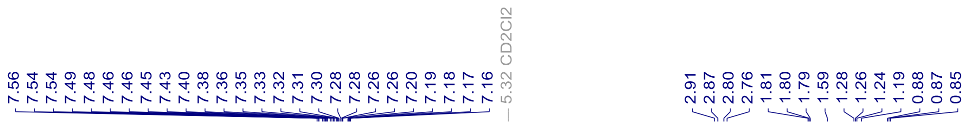

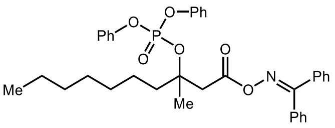

S36- ^{1}H NMR (400 MHz, CD_2Cl_2)

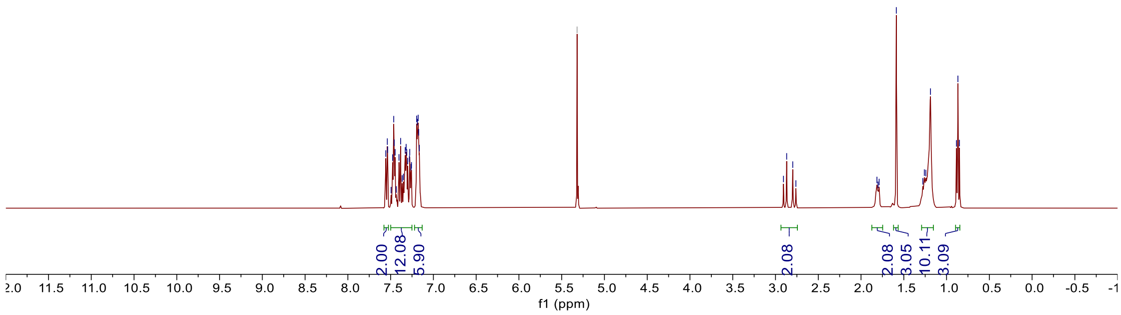

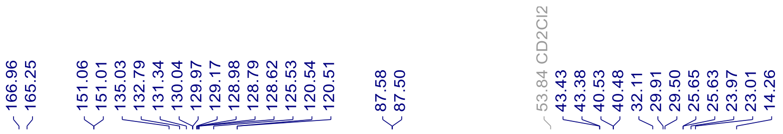

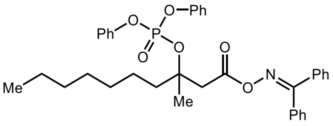

S36- ^{13}C NMR (100 MHz, CD_2Cl_2)

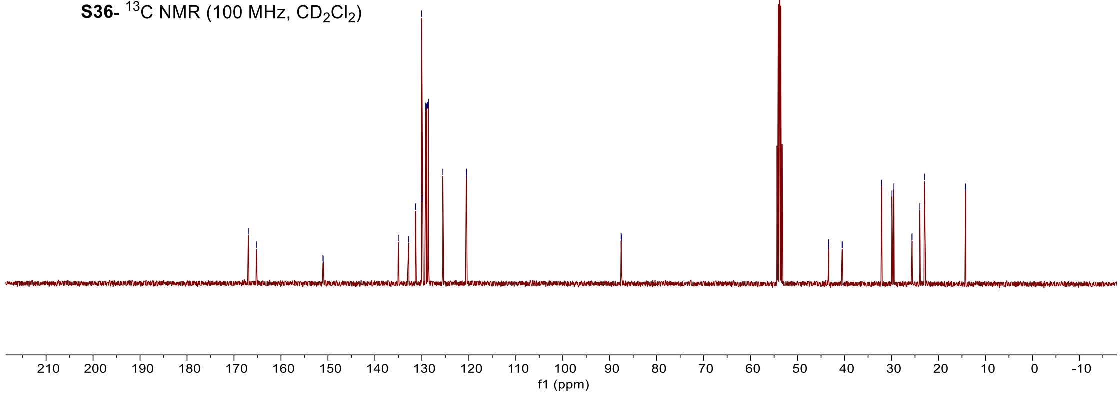

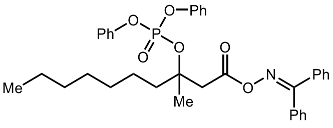

S36- ^{31}P NMR (160 MHz, CD_2Cl_2)

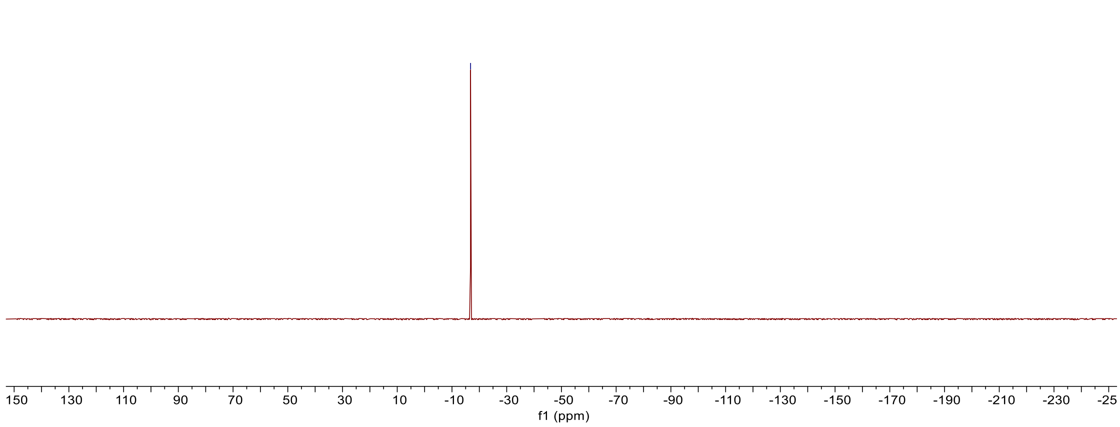

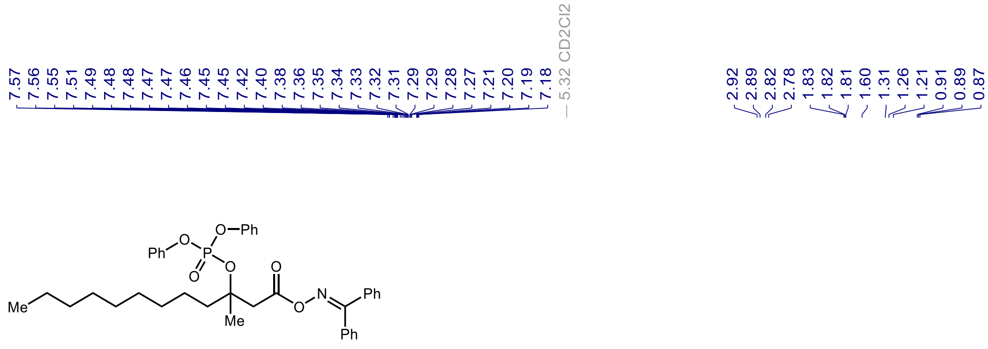

S37- ^{1}H NMR (400 MHz, CD_2Cl_2)

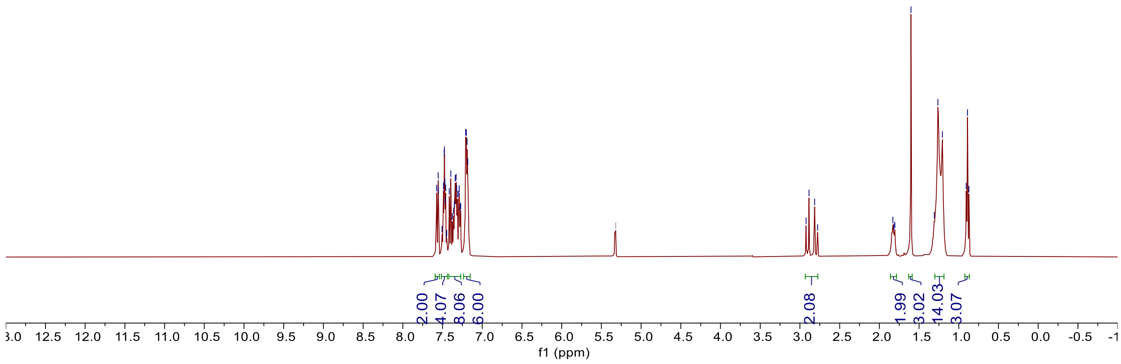

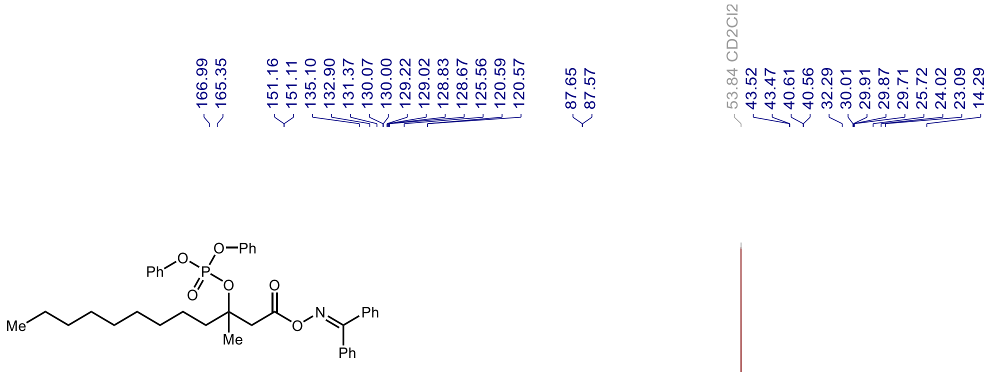

S37- ^{13}C NMR (100 MHz, CD_2Cl_2)

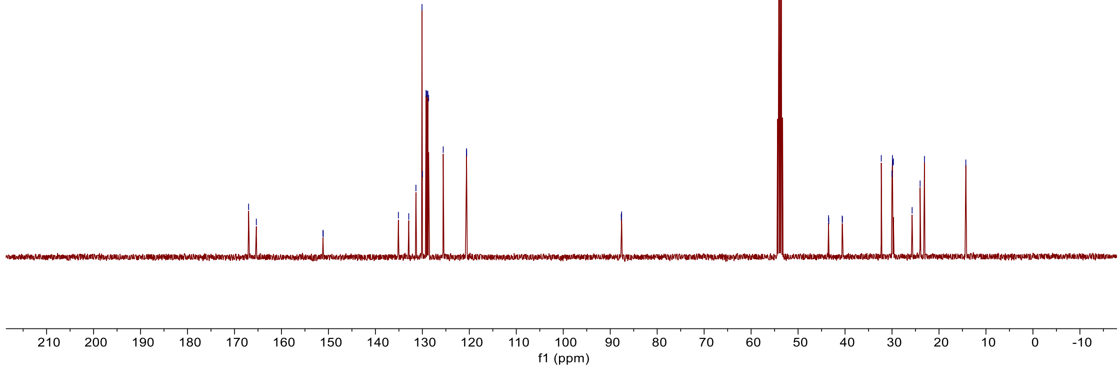

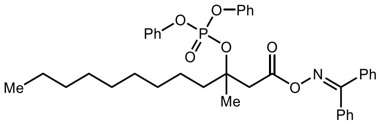

S37- ^{31}P NMR (160 MHz, CD_2Cl_2)

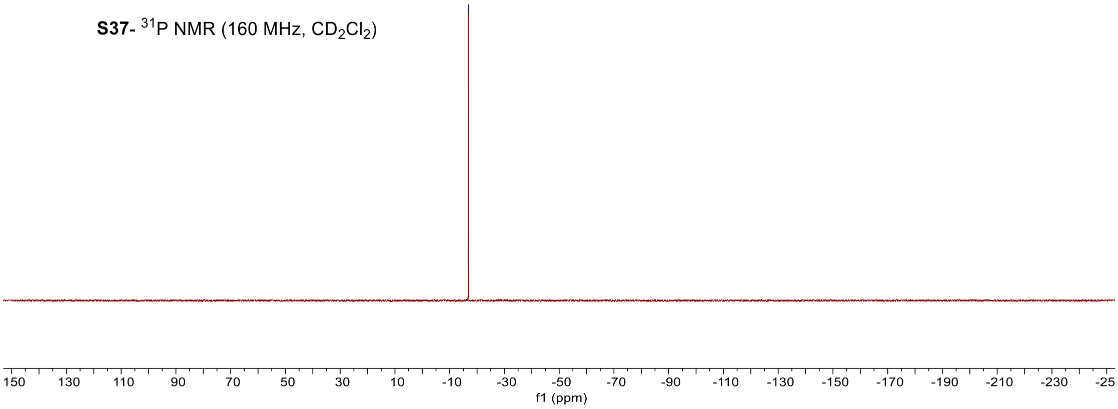

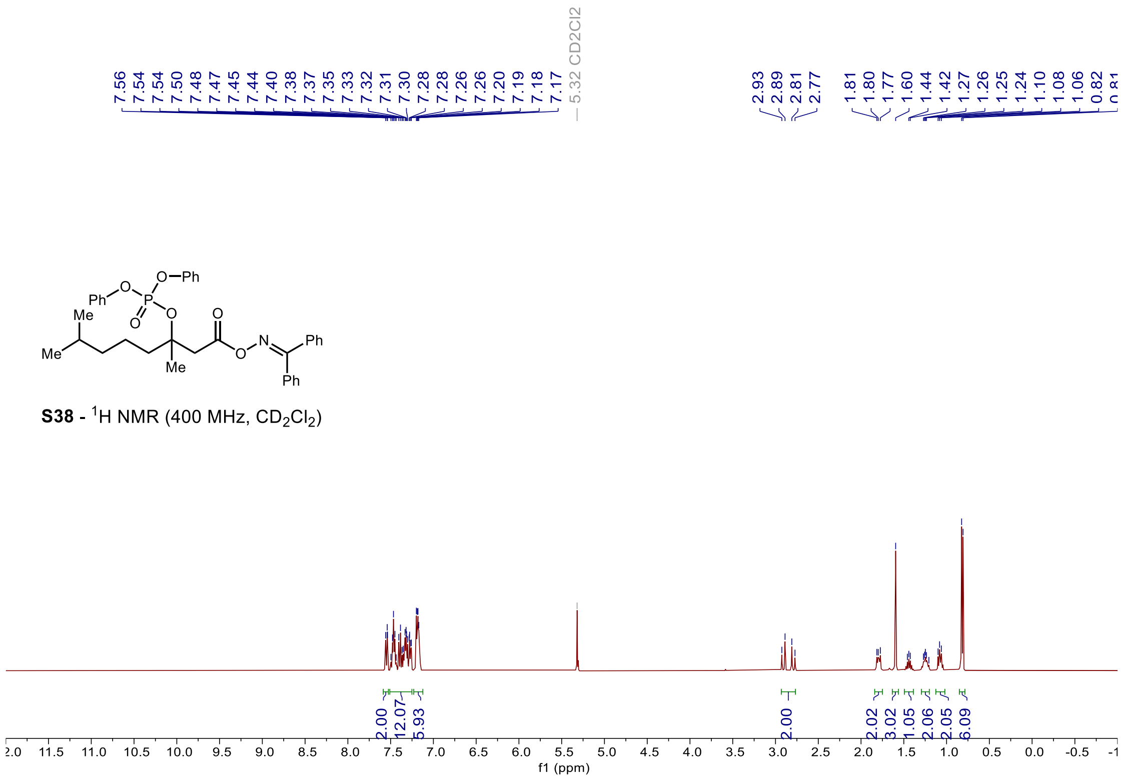

S38 - ^{1}H NMR (400 MHz, CD_2Cl_2)

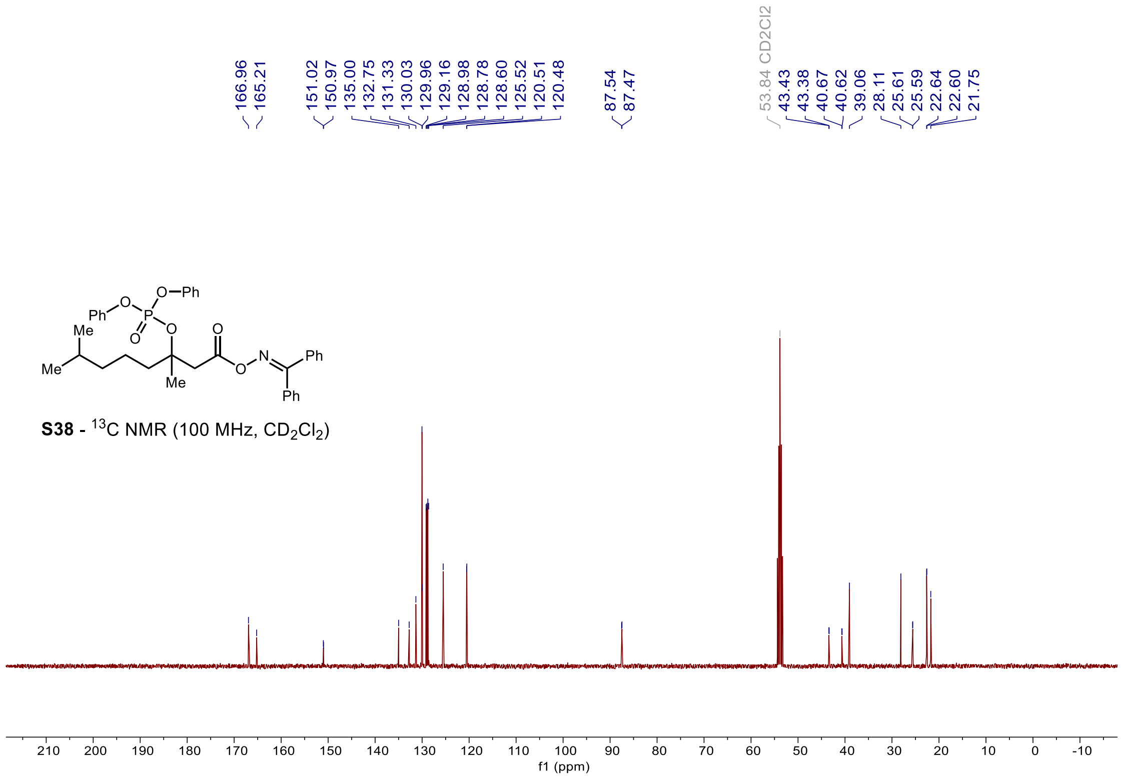

S38 - ^{13}C NMR (100 MHz, CD_2Cl_2)

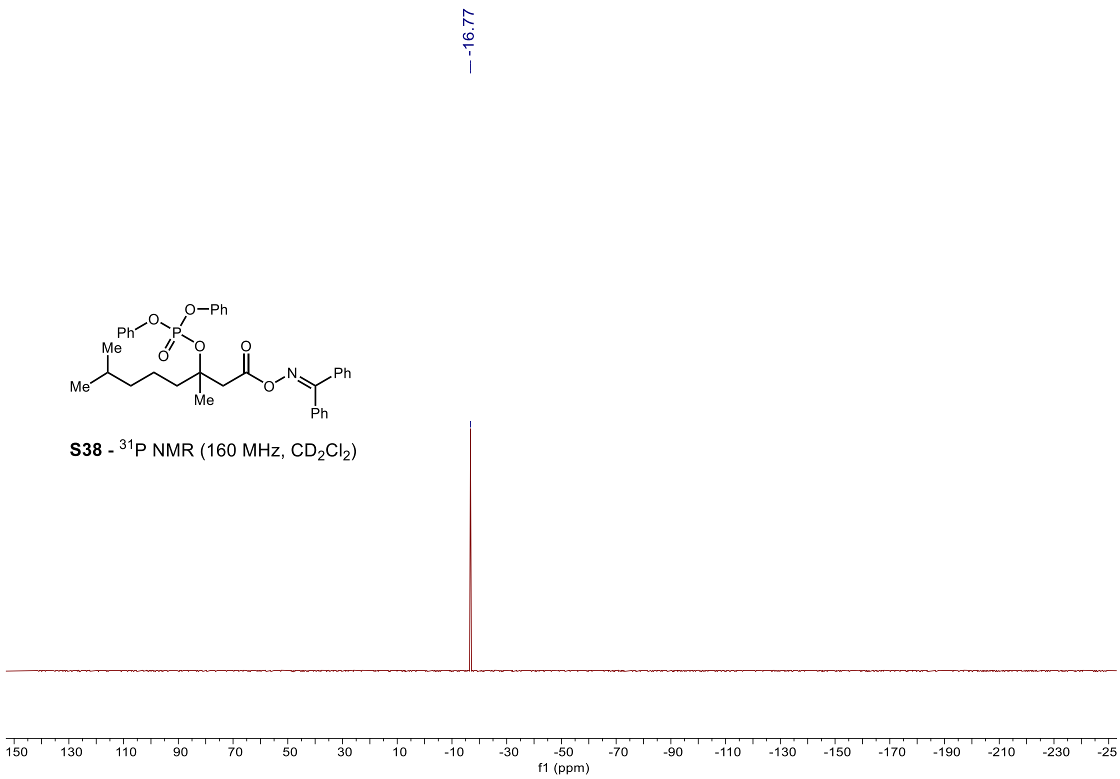

S38 - ^{31}P NMR (160 MHz, CD_2Cl_2)

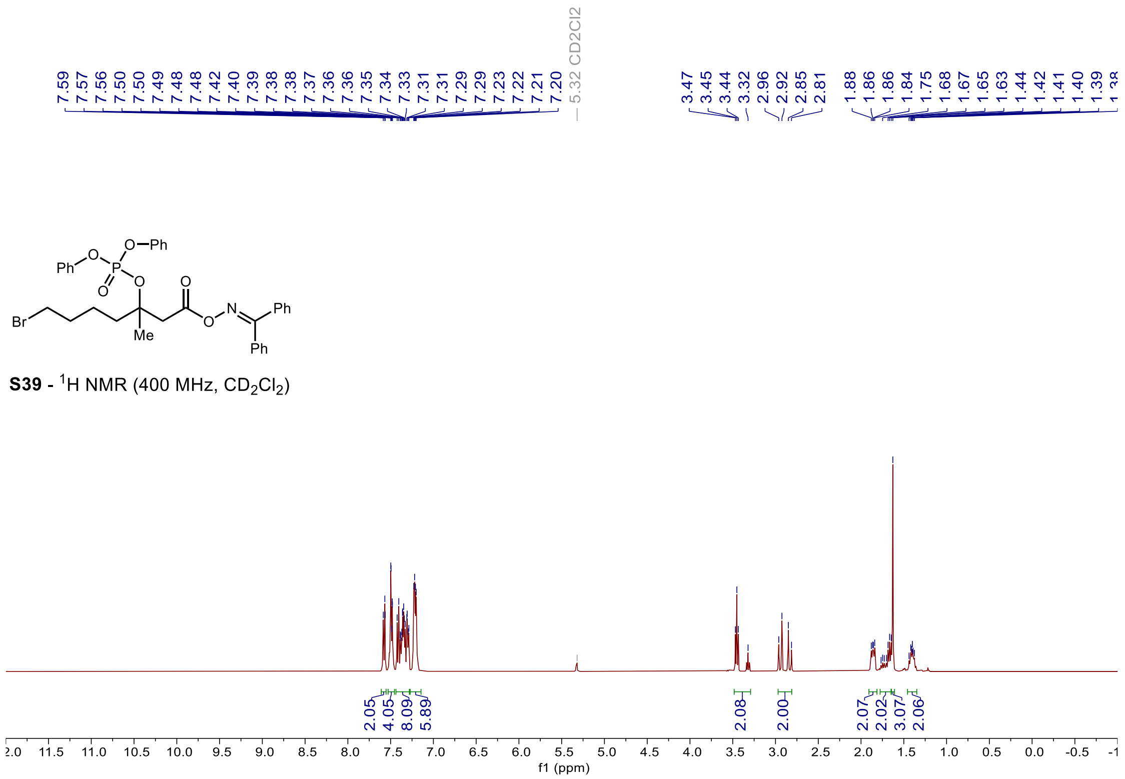

S39 - ^{1}H NMR (400 MHz, CD_2Cl_2)

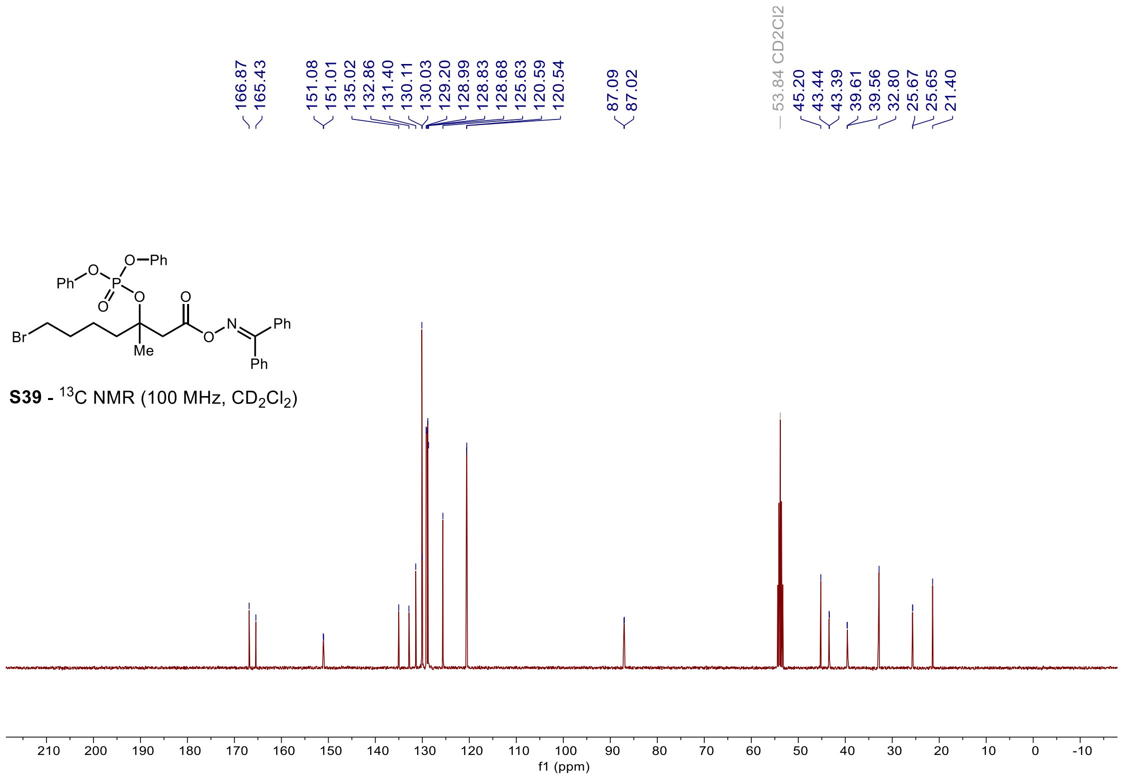

S39 - ^{13}C NMR (100 MHz, CD_2Cl_2)

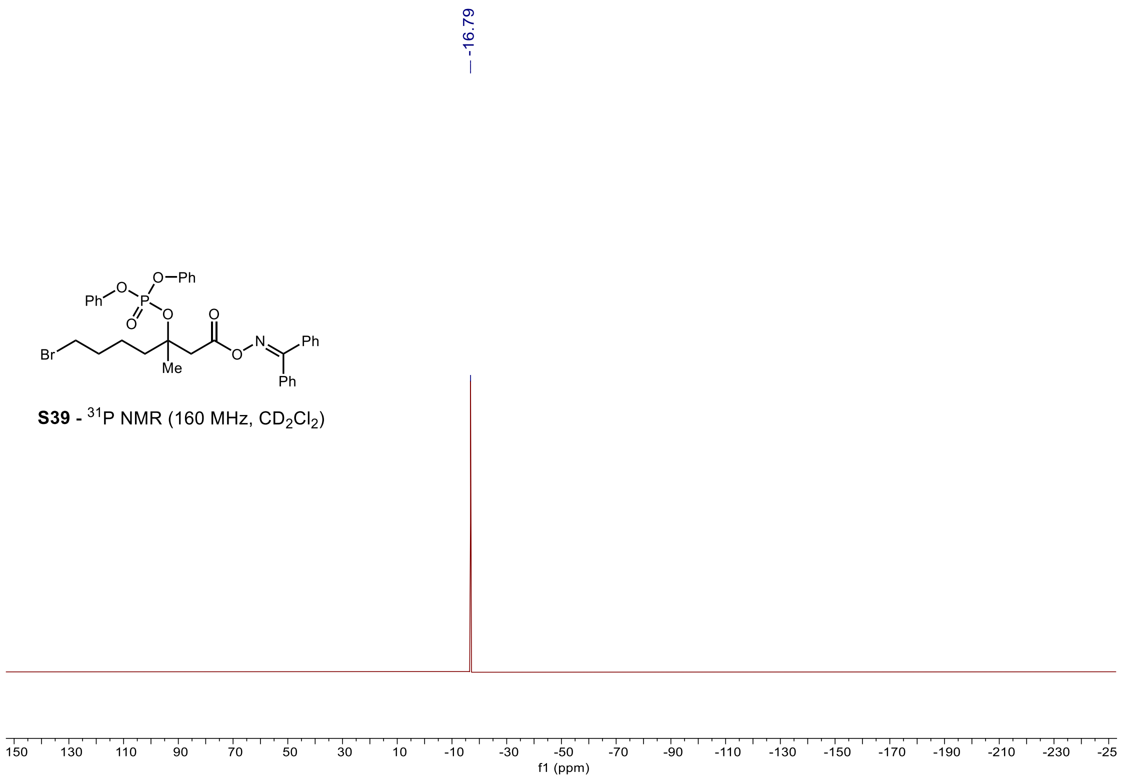

S39 - ^{31}P NMR (160 MHz, CD_2Cl_2)

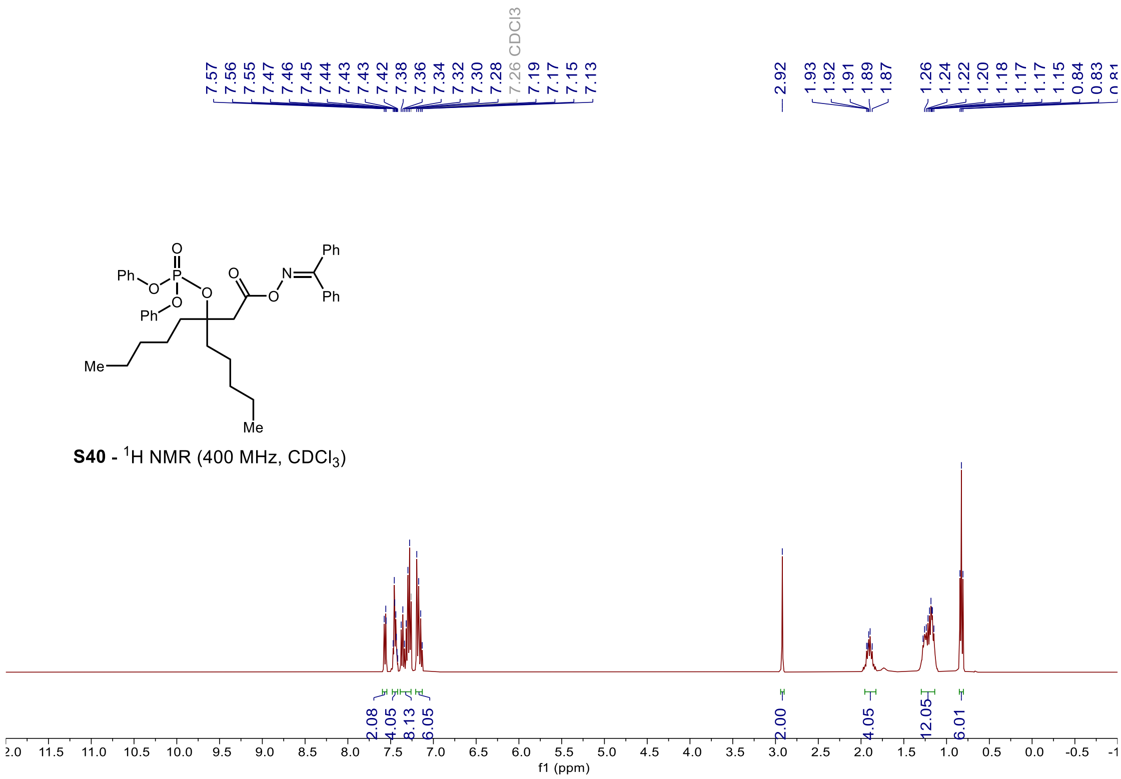

S40 - ^{1}H NMR (400 MHz, $CDCl_3$)

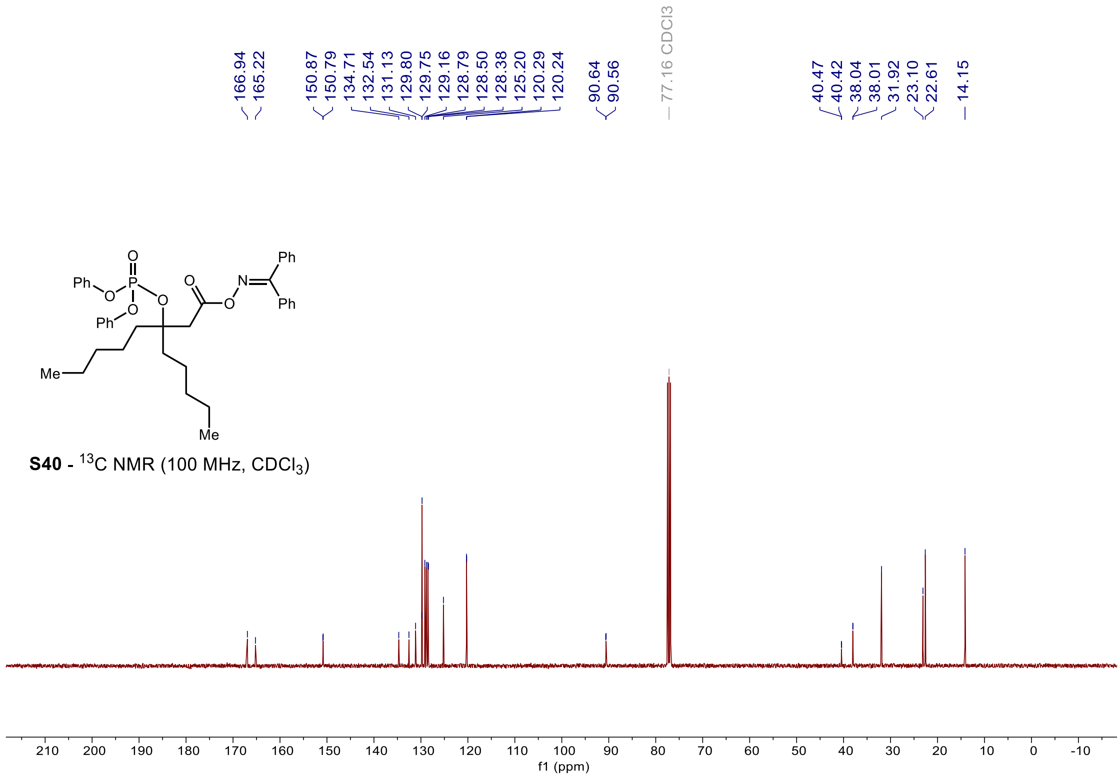

S40 - ^{13}C NMR (100 MHz, $CDCl_3$)

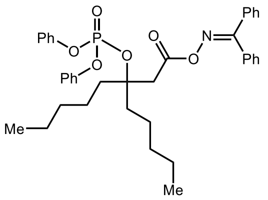

S40 - ^{31}P NMR (160 MHz, $CDCl_3$)

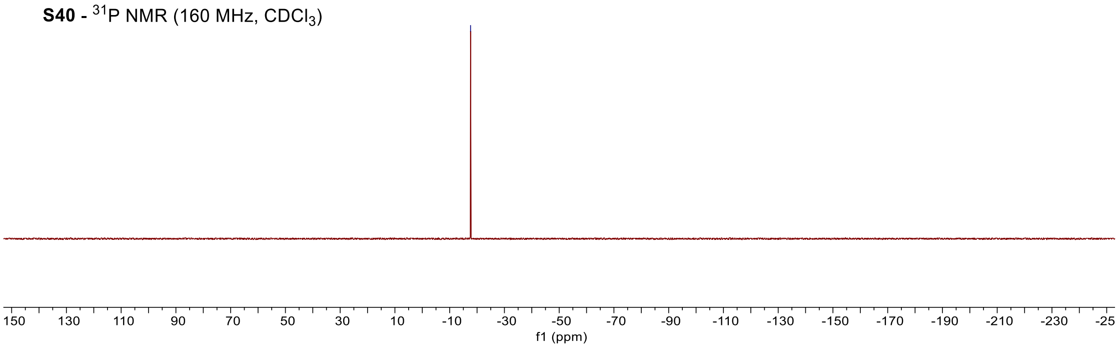

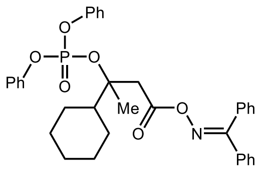

S41 - ^{1}H NMR (400 MHz, CD_2Cl_2)

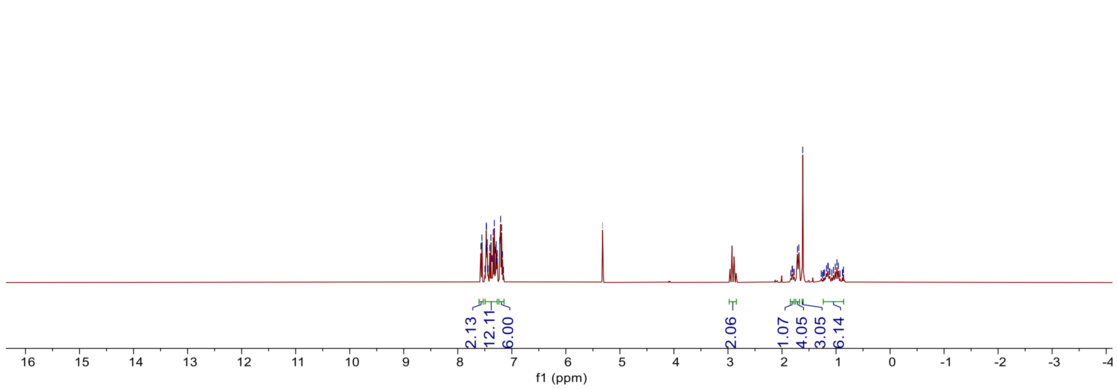

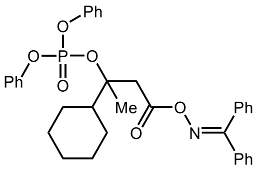

S41 - ^{13}C NMR (100 MHz, CD_2Cl_2)

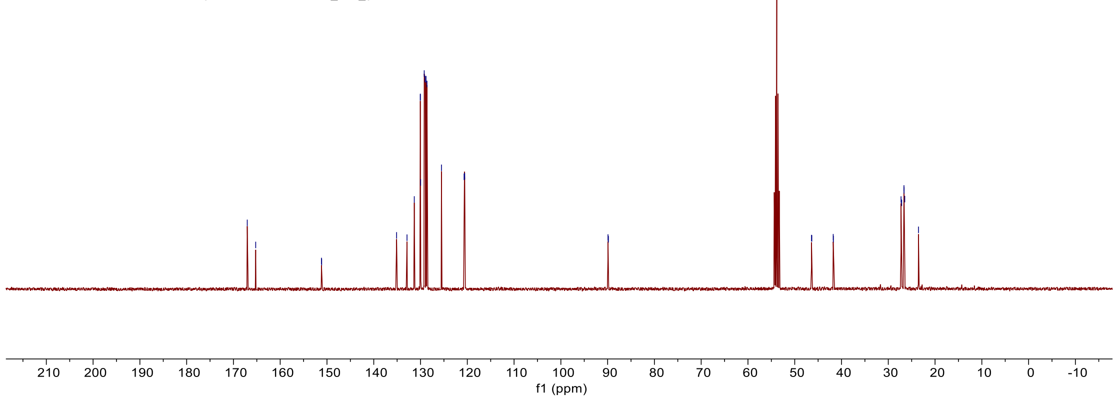

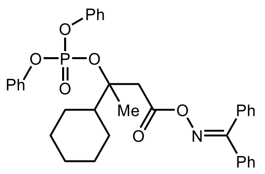

S41 - ^{31}P NMR (160 MHz, CD_2Cl_2)

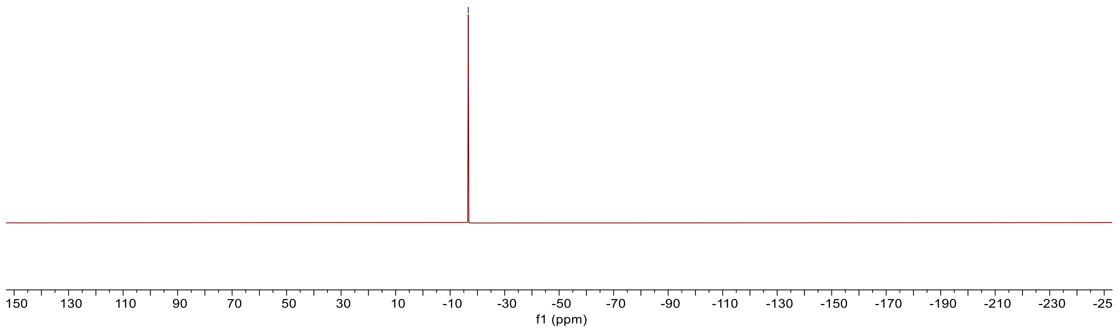

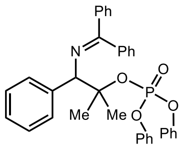

12 - ^{1}H NMR (400 MHz, CDCl$_3$)

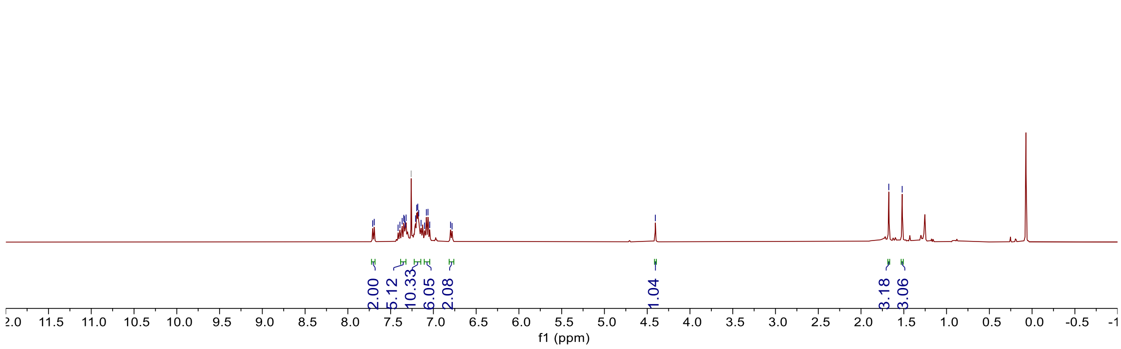

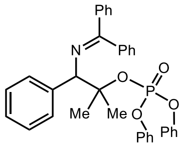

12 - ^{13}C NMR (100 MHz, CDCl$_3$)

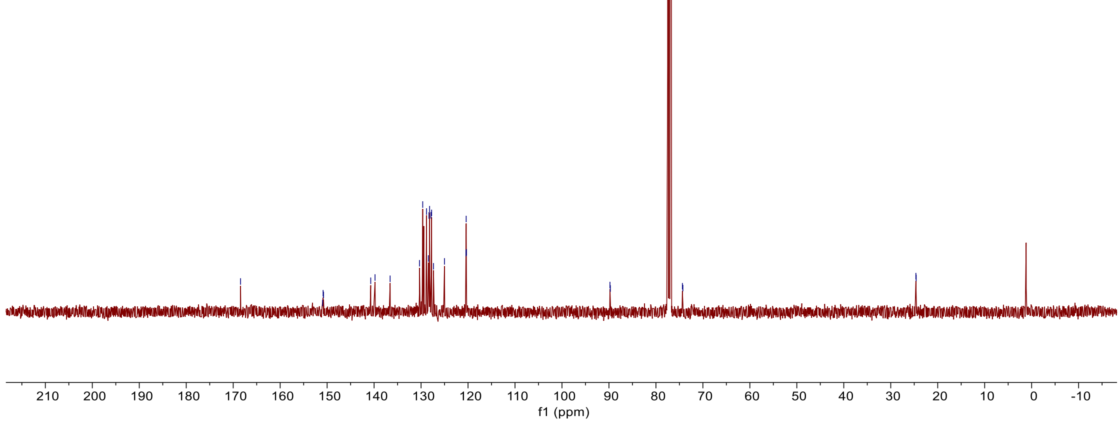

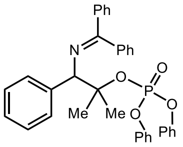

12 - ^{31}P NMR (160 MHz, $CDCl_3$)

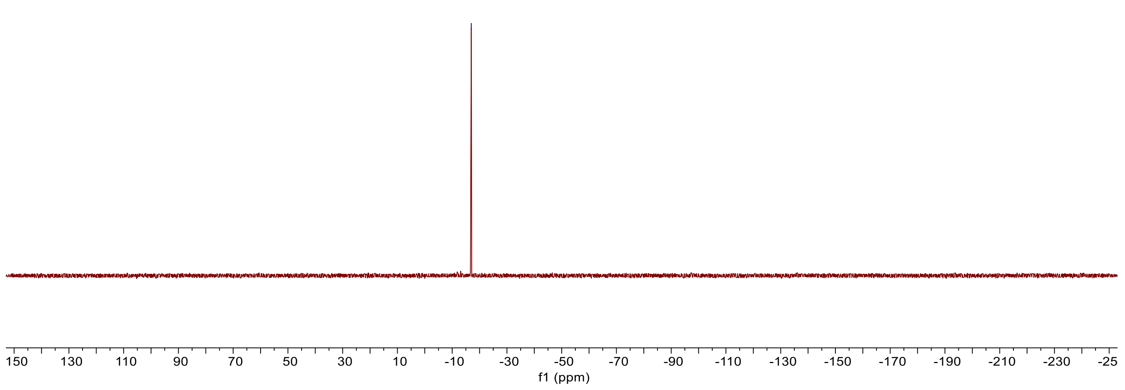

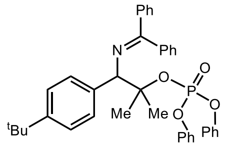

14 - ^{1}H NMR (400 MHz, $CDCl_3$)

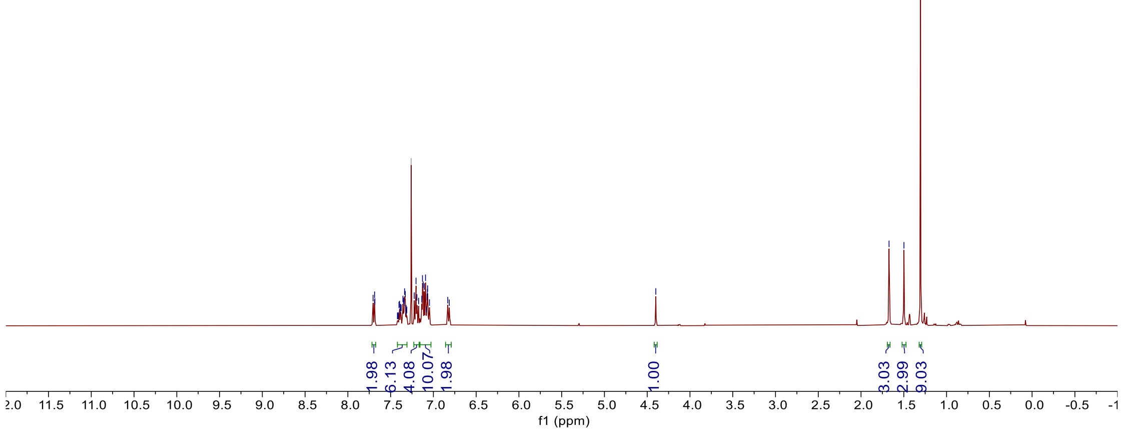

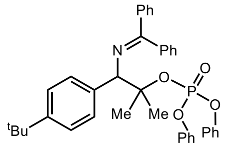

14 - ^{13}C NMR (100 MHz, $CDCl_3$)

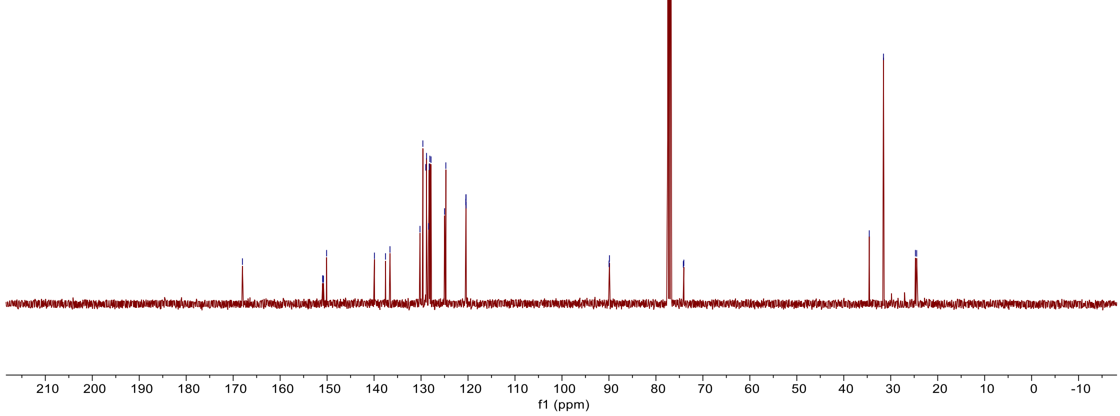

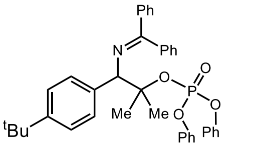

14 - ^{31}P NMR (160 MHz, CDCl$_3$)

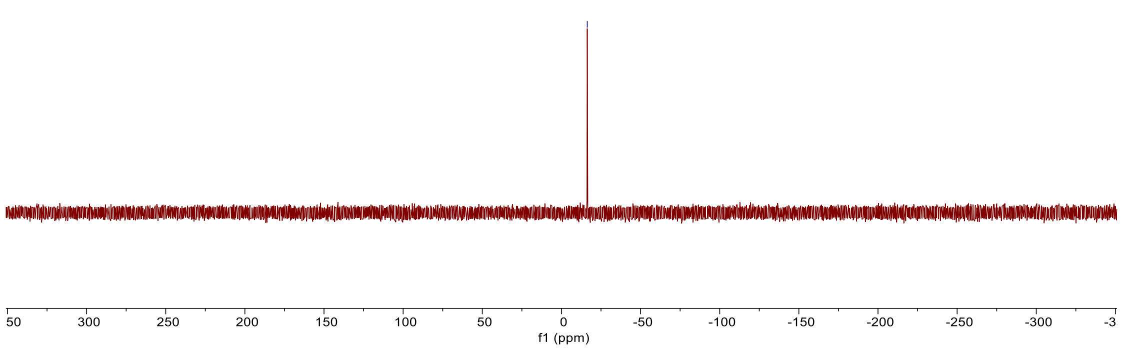

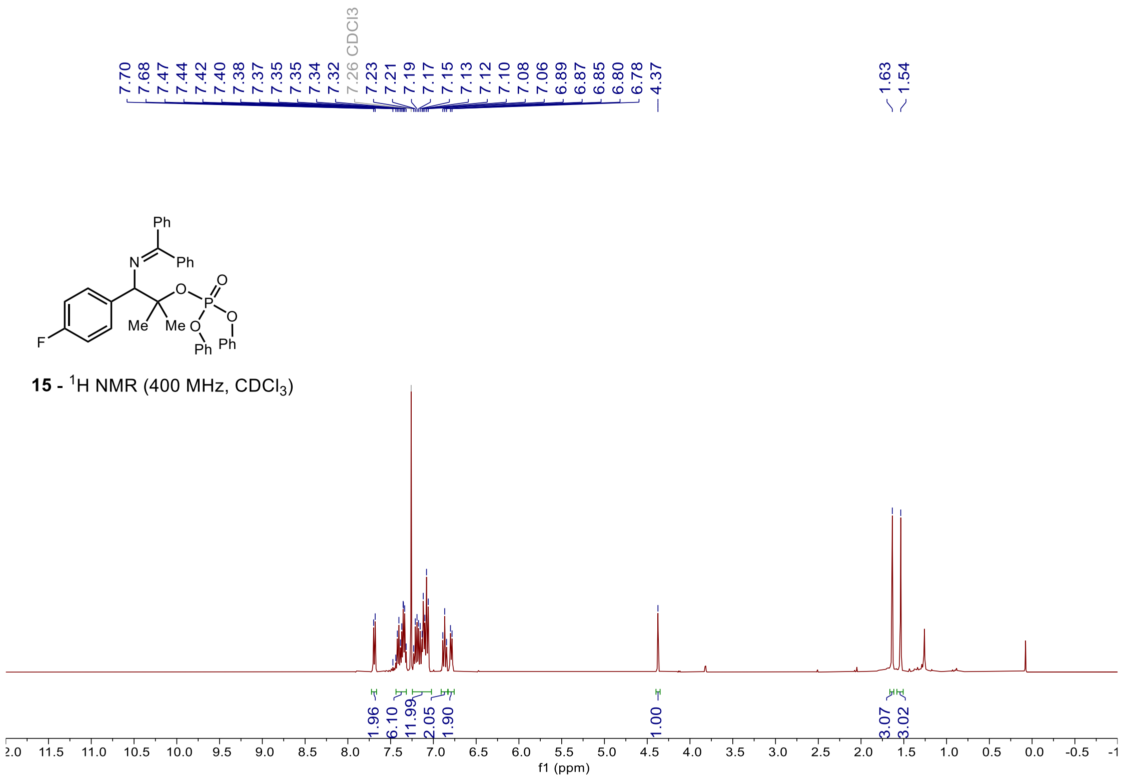

15 - ^{1}H NMR (400 MHz, $CDCl_3$)

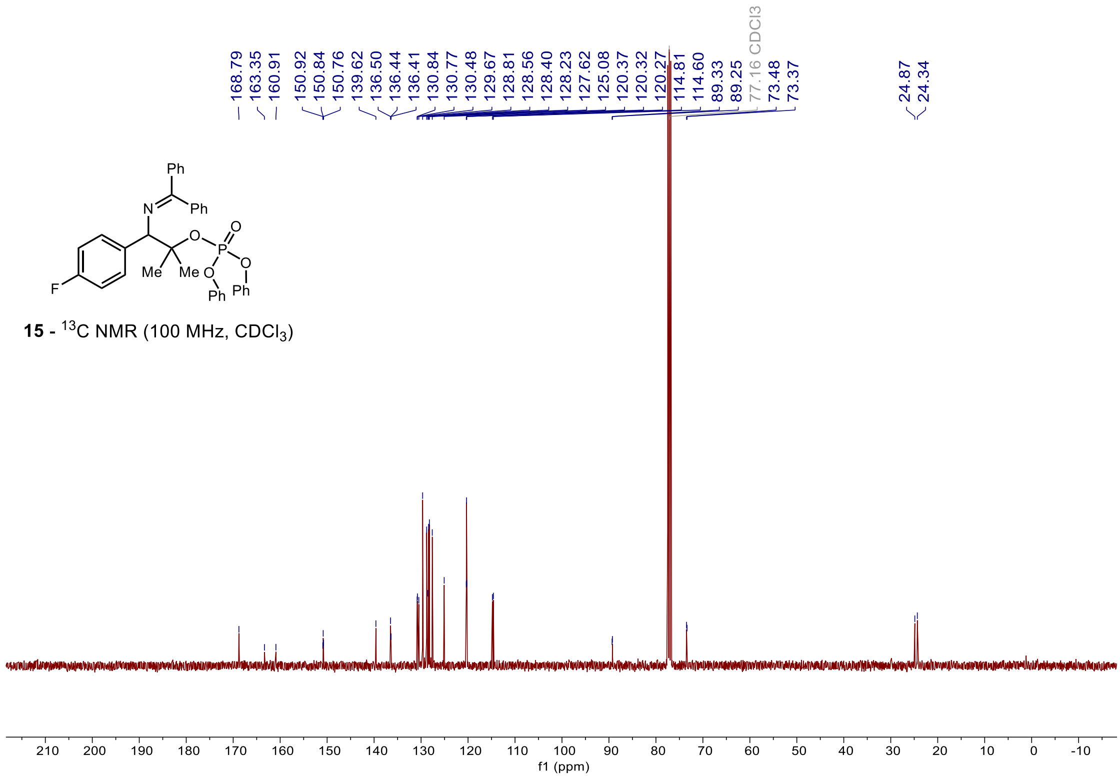

15 - ^{13}C NMR (100 MHz, $CDCl_3$)

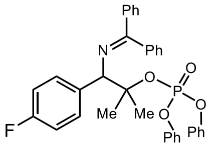

15 - ^{31}P NMR (160 MHz, $CDCl_3$)

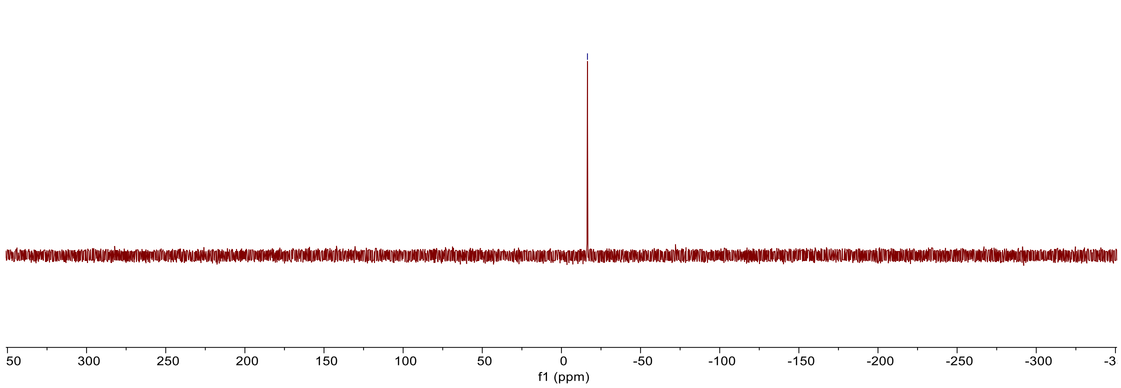

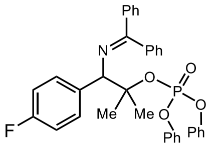

15 - ^{19}F NMR (376 MHz, $CDCl_3$)

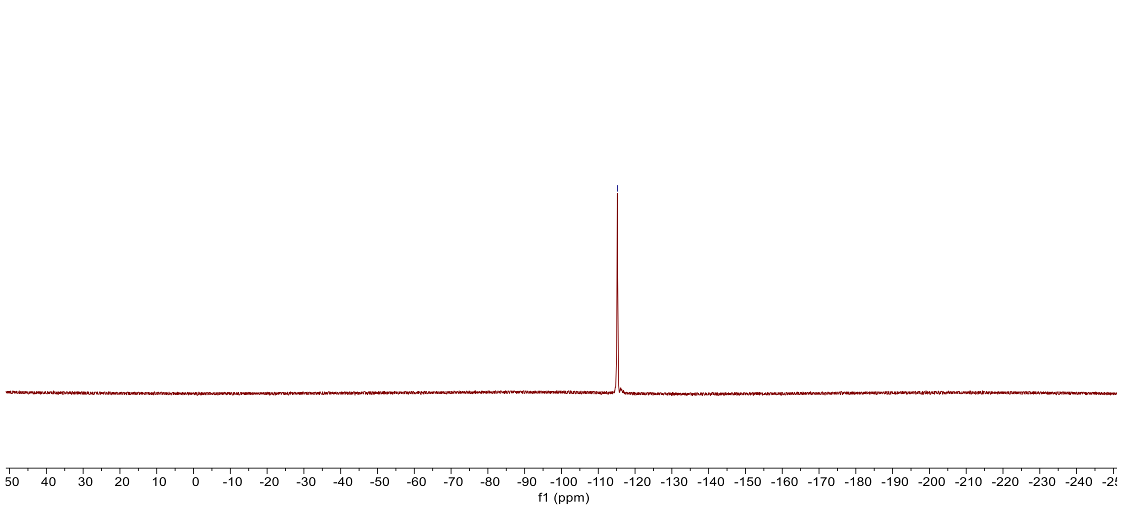

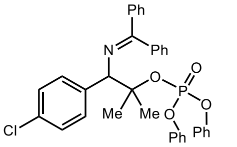

16 - ^{1}H NMR (400 MHz, CD_2Cl_2)

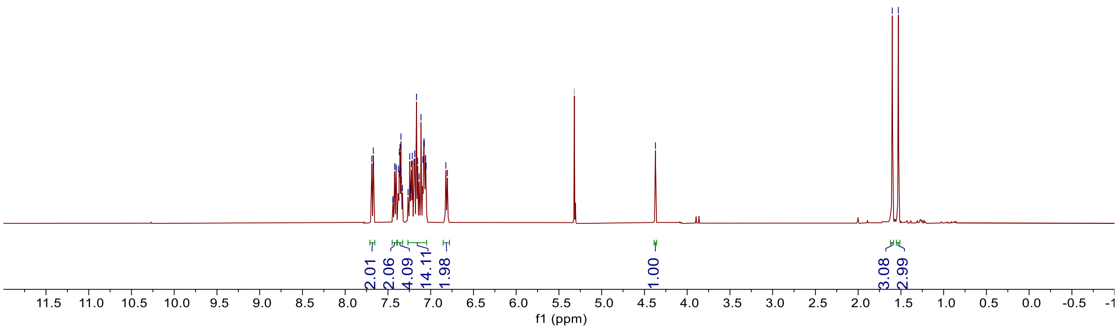

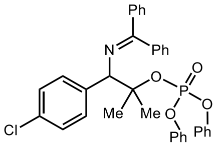

16 - ^{13}C NMR (100 MHz, CD_2Cl_2)

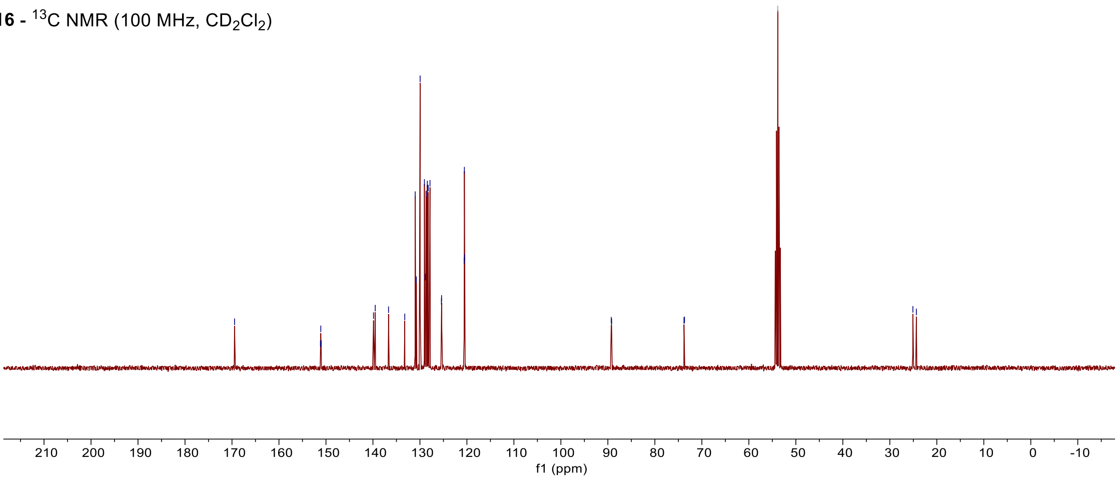

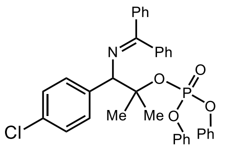

16 - ^{31}P NMR (160 MHz, CD_2Cl_2)

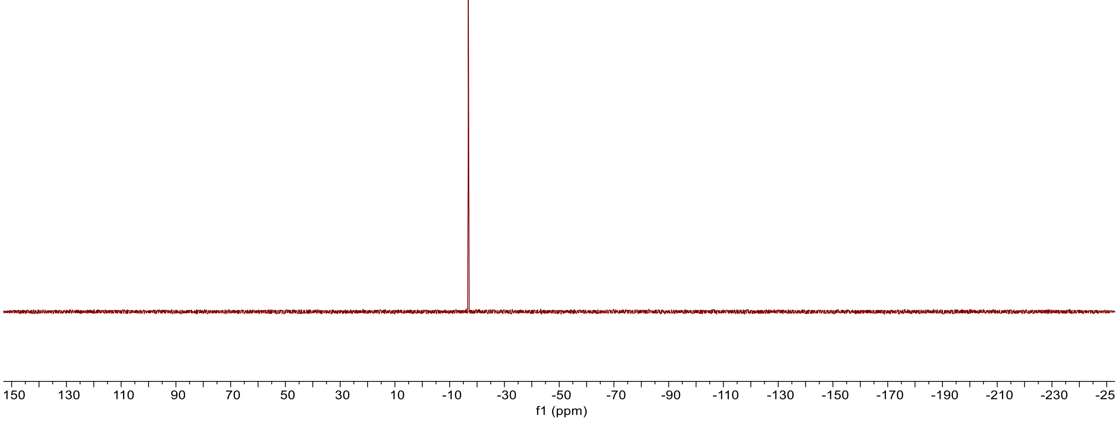

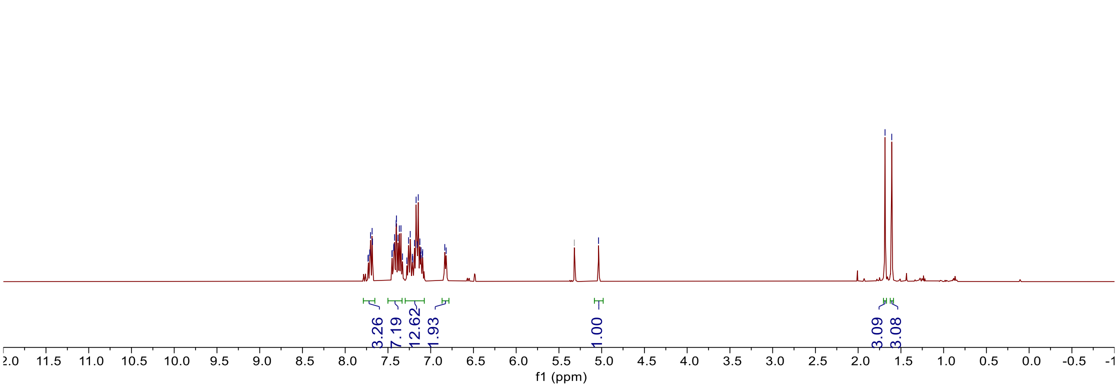

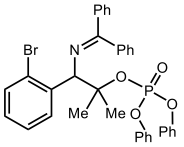

17 - ^{1}H NMR (400 MHz, CD_2Cl_2)

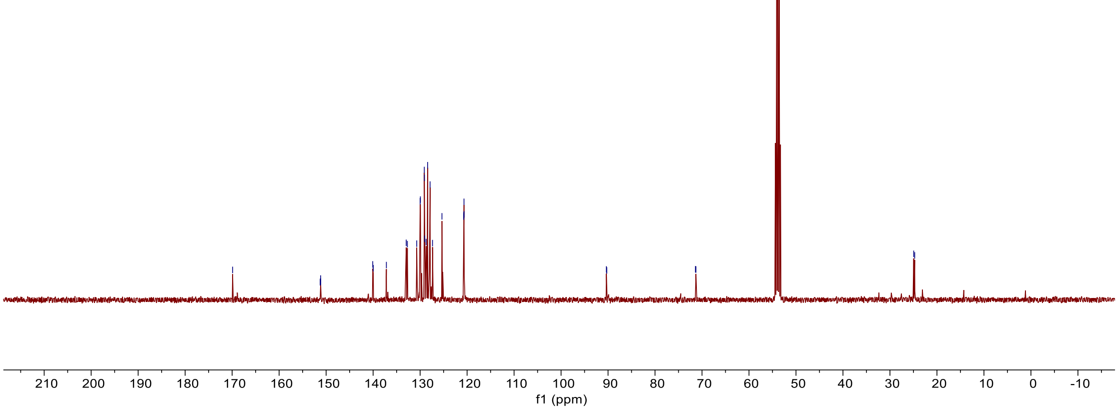

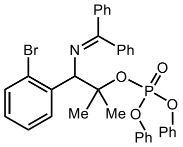

17 - ^{13}C NMR (100 MHz, CD_2Cl_2)

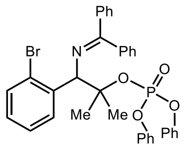

17 - ^{31}P NMR (160 MHz, CD_2Cl_2)

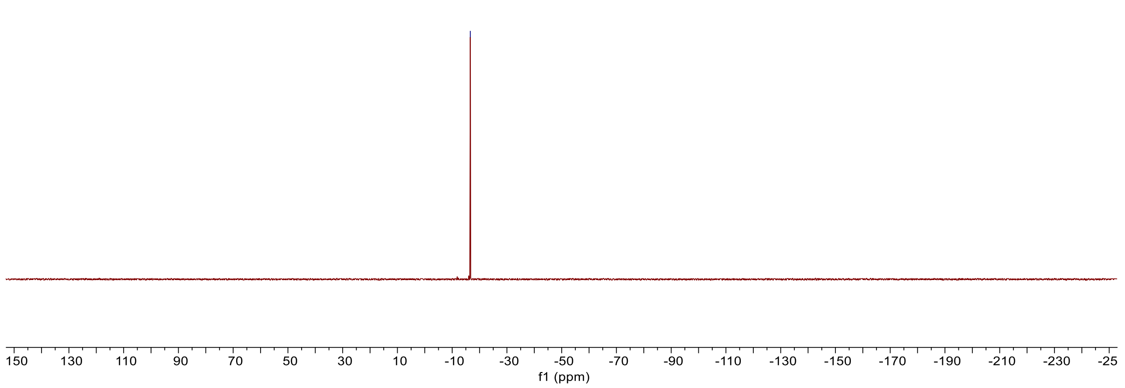

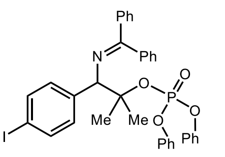

18 - ^{1}H NMR (400 MHz, CD_2Cl_2)

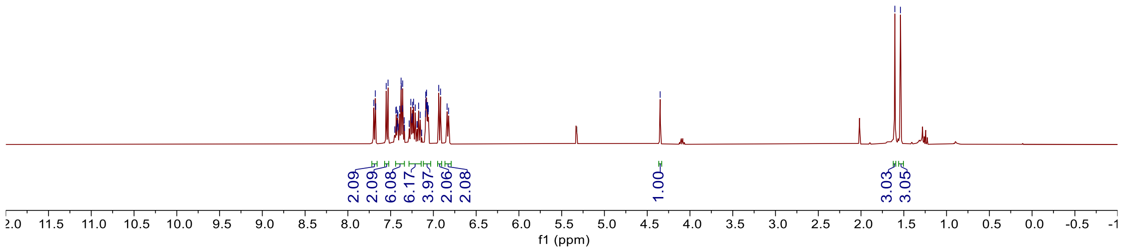

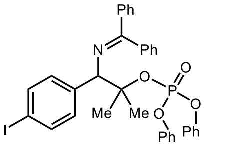

18 - ^{13}C NMR (100 MHz, CD_2Cl_2)

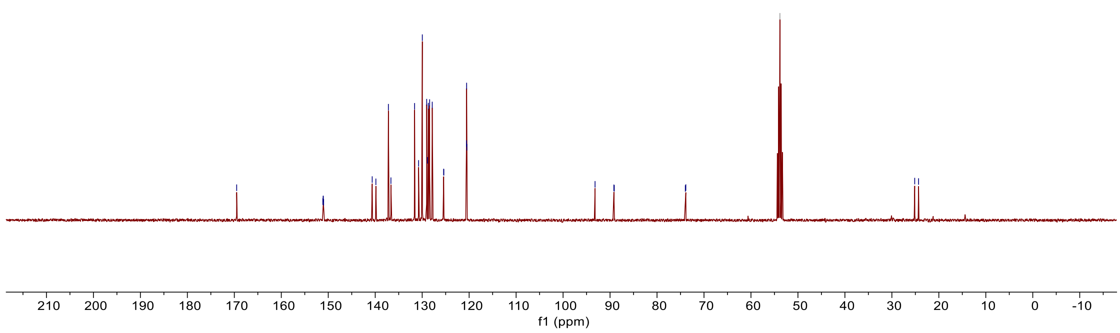

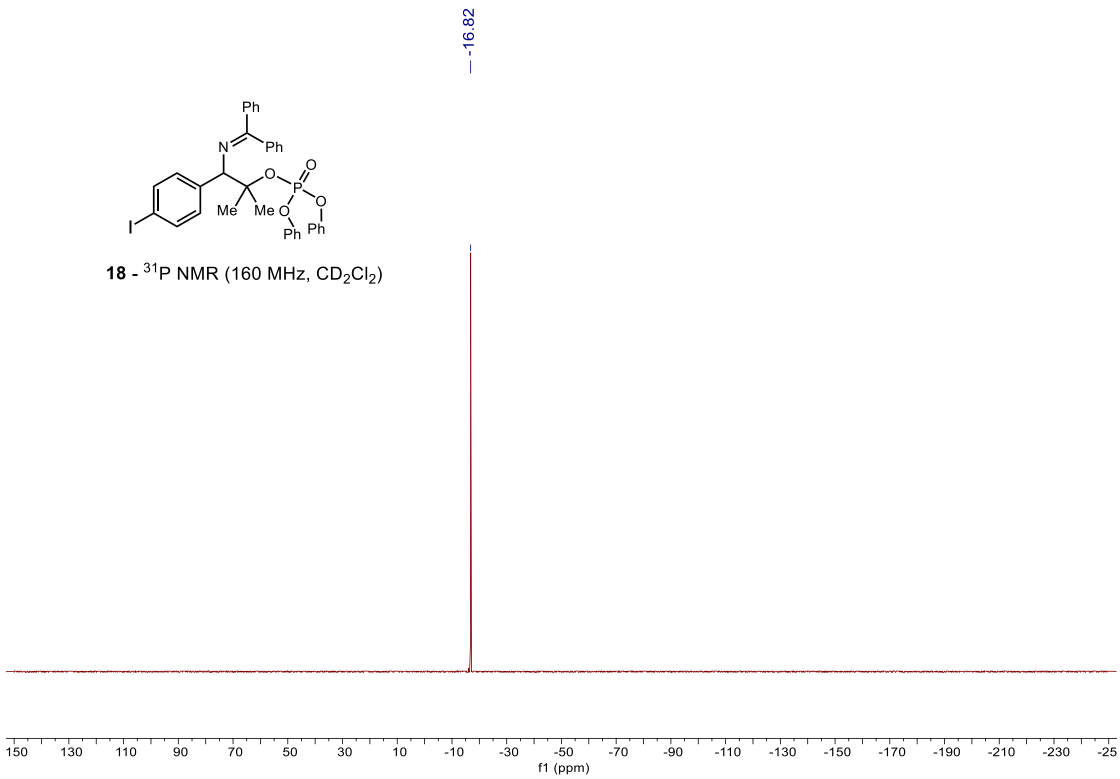

18 - ^{31}P NMR (160 MHz, CD_2Cl_2)

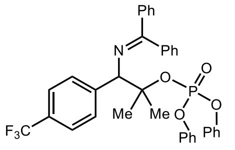

19 - ^{1}H NMR (400 MHz, CD_2Cl_2)

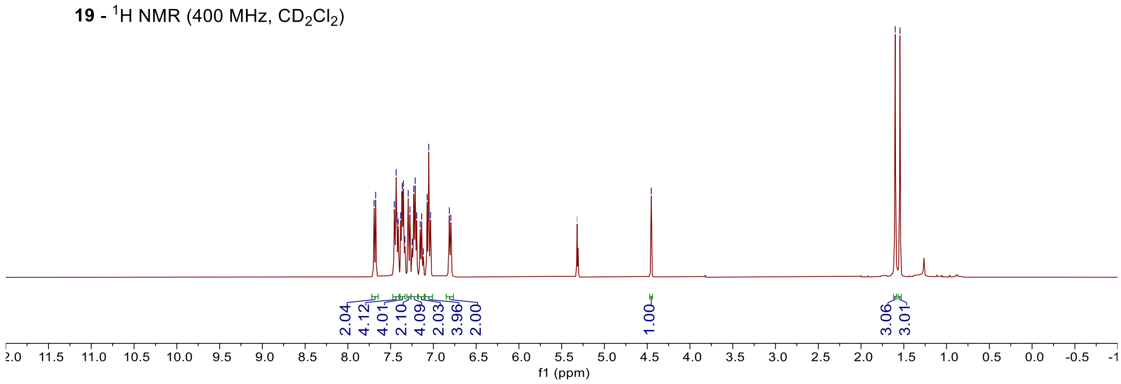

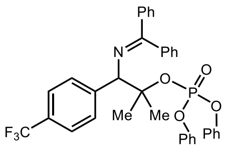

19 - ^{13}C NMR (100 MHz, CD_2Cl_2)

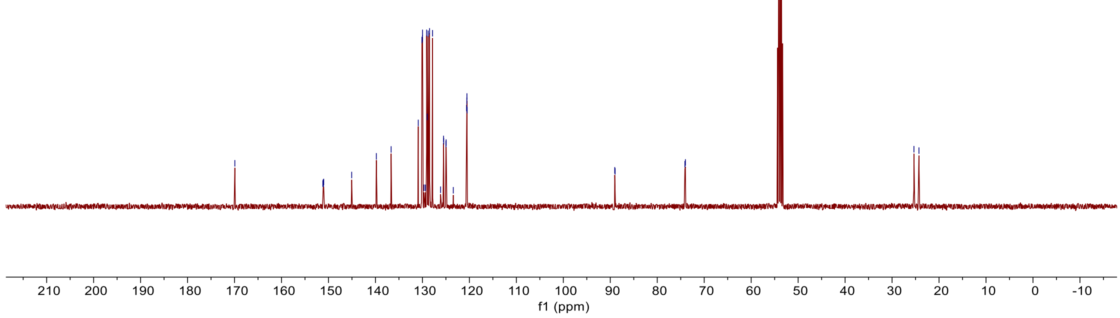

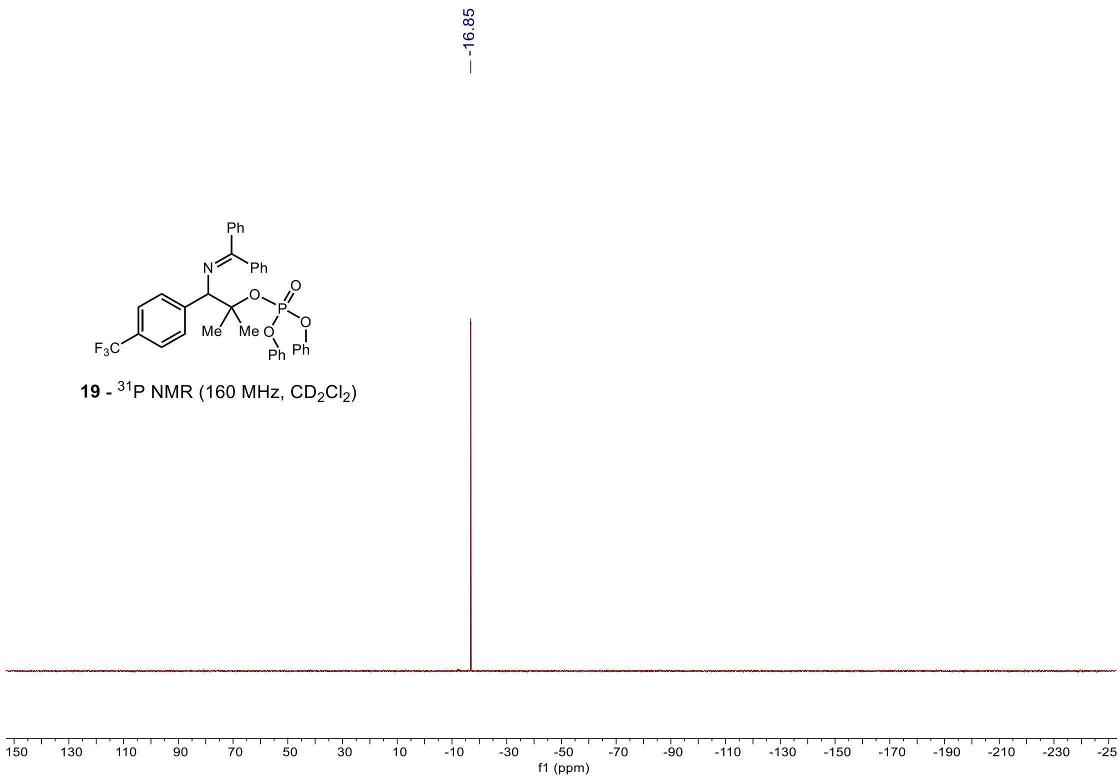

19 - ^{31}P NMR (160 MHz, CD_2Cl_2)

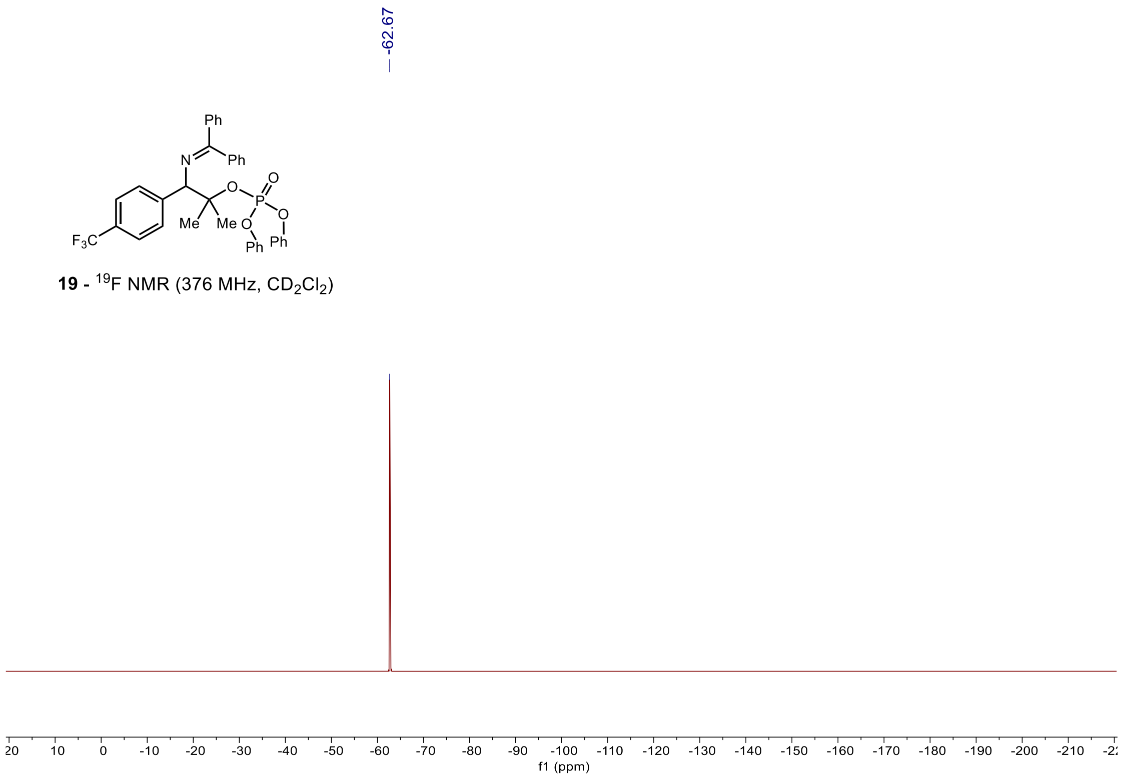

19 - ^{19}F NMR (376 MHz, CD_2Cl_2)

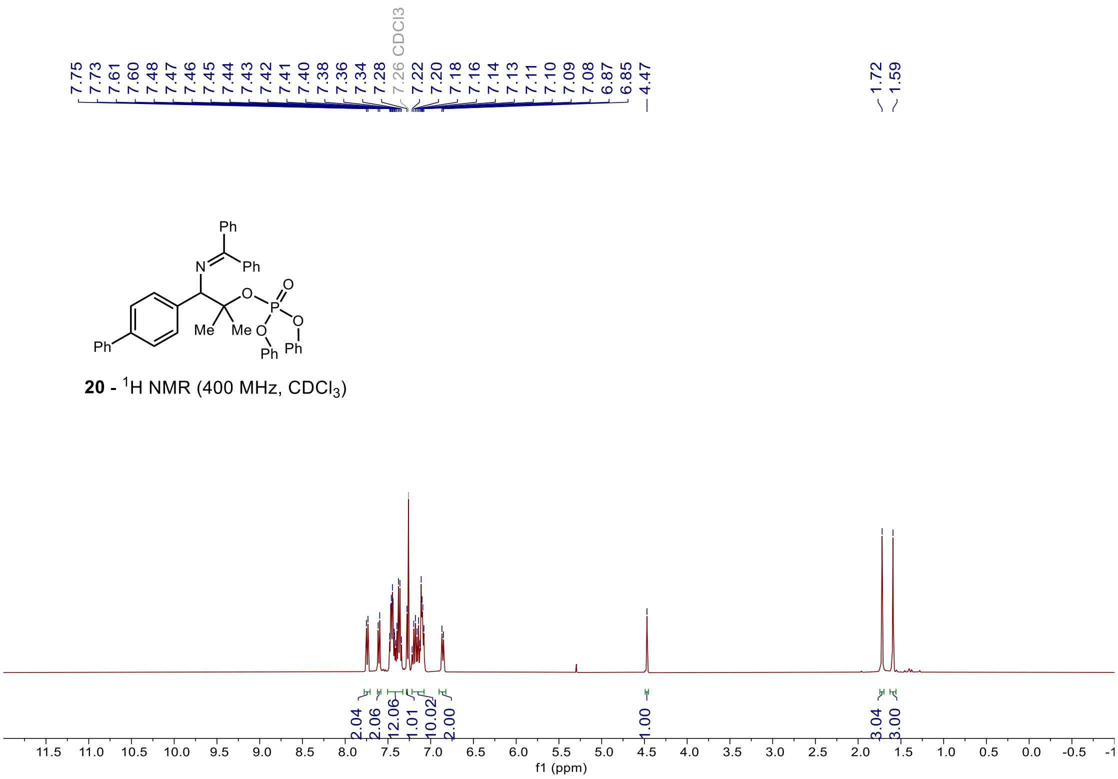

20 - ^{1}H NMR (400 MHz, $CDCl_3$)

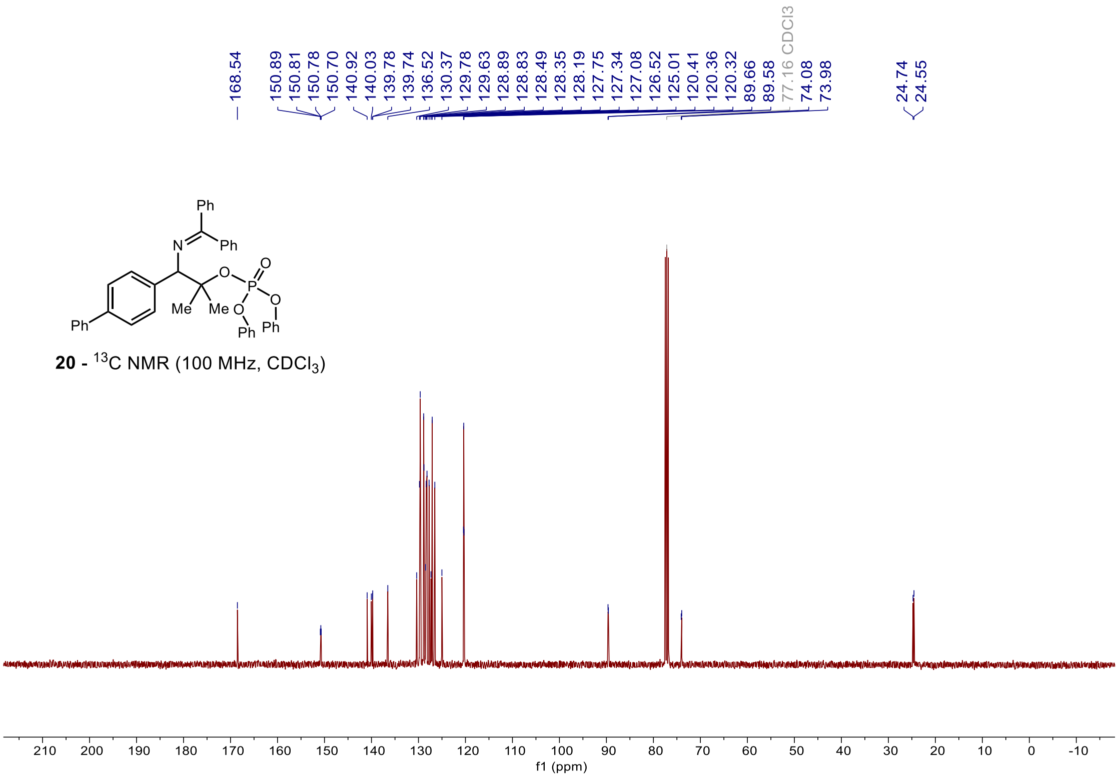

20 - ^{13}C NMR (100 MHz, $CDCl_3$)

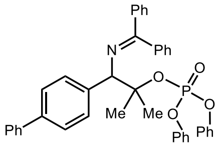

20 - ^{31}P NMR (160 MHz, $CDCl_3$)

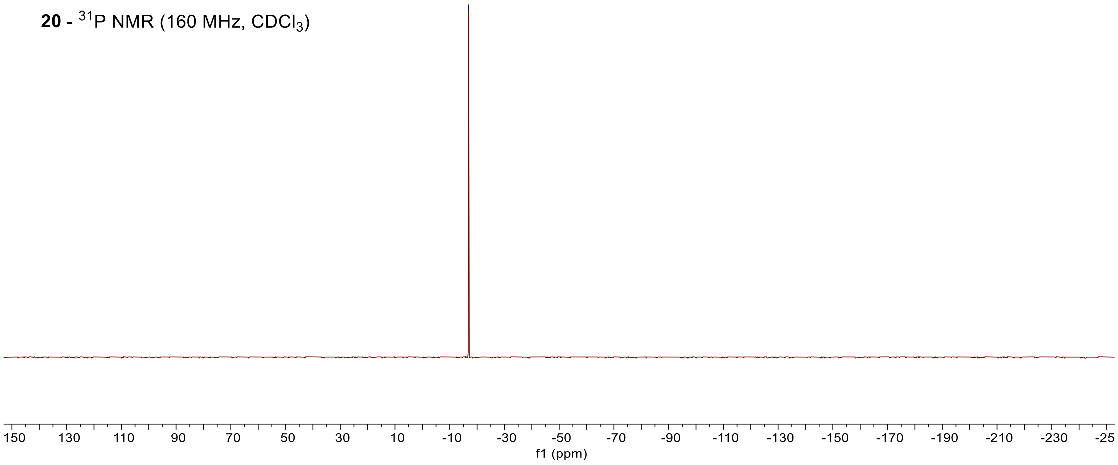

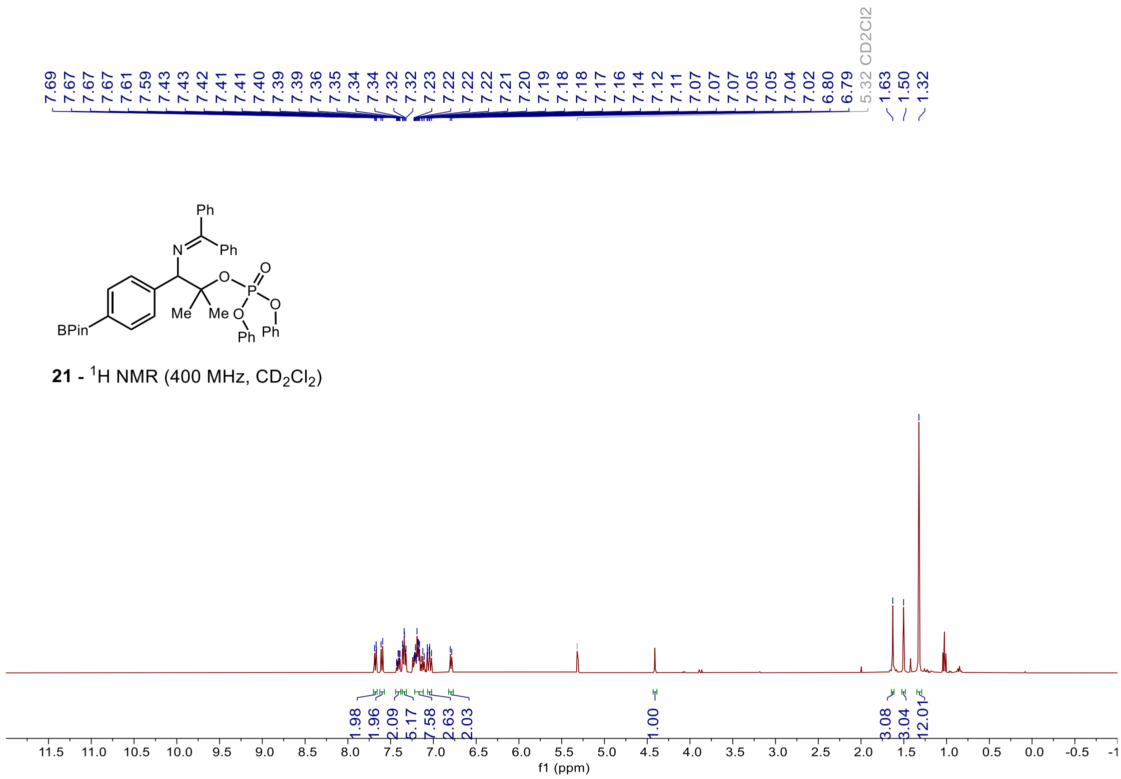

21 - ^{1}H NMR (400 MHz, CD_2Cl_2)

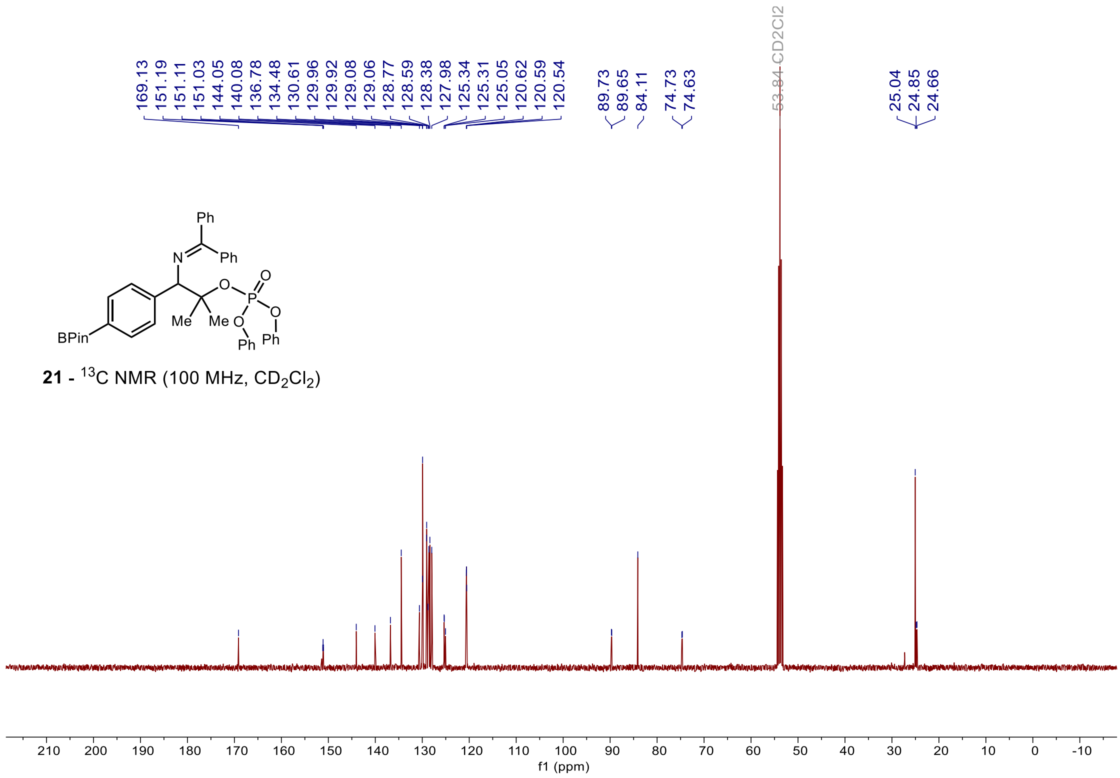

21 - ^{13}C NMR (100 MHz, CD_2Cl_2)

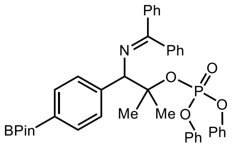

21 - ^{31}P NMR (160 MHz, CD_2Cl_2)

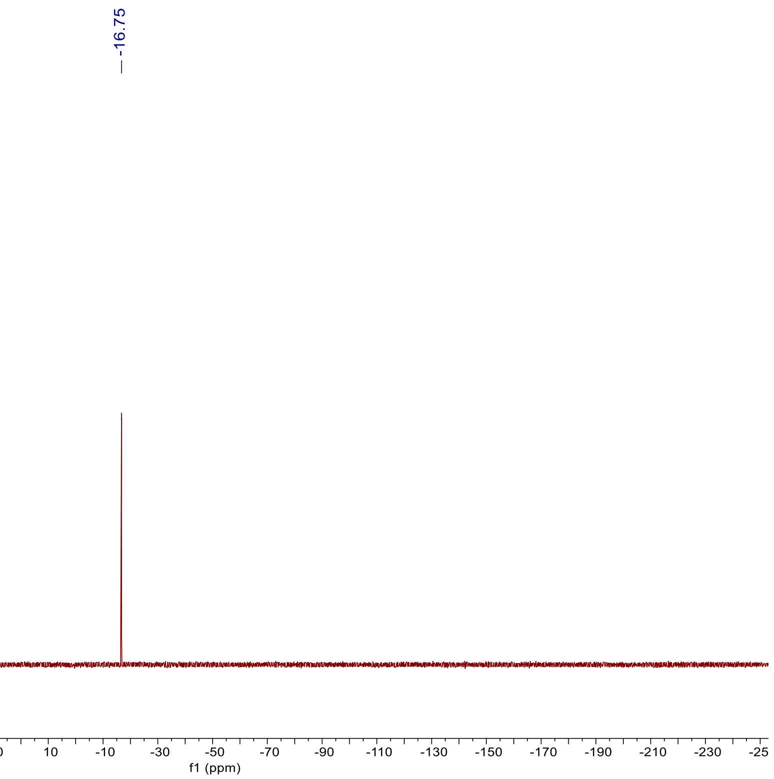

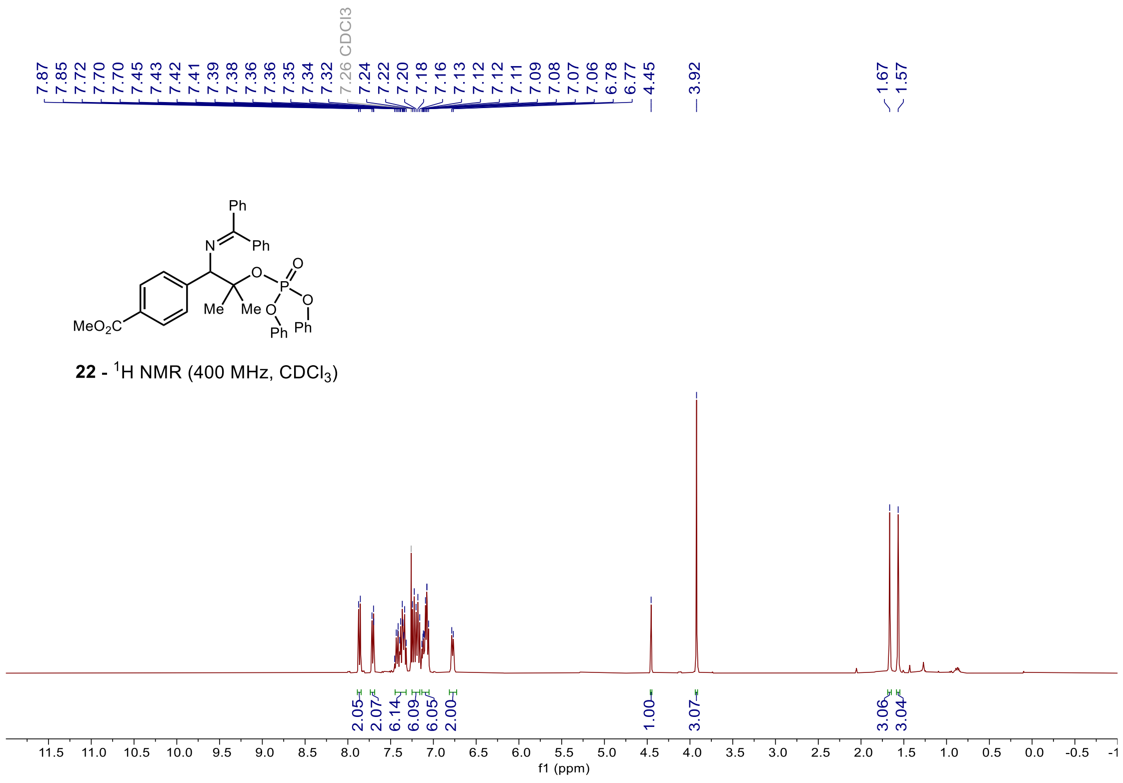

22 - ^{1}H NMR (400 MHz, $CDCl_3$)

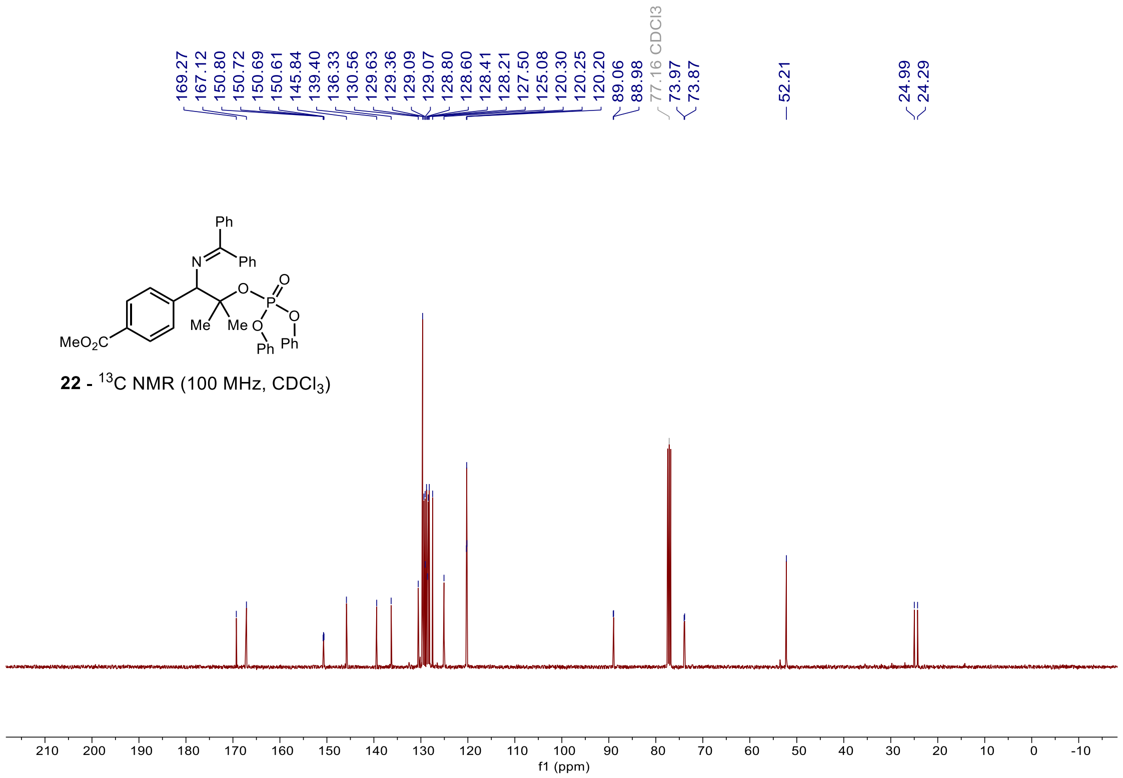

22 - ^{13}C NMR (100 MHz, $CDCl_3$)

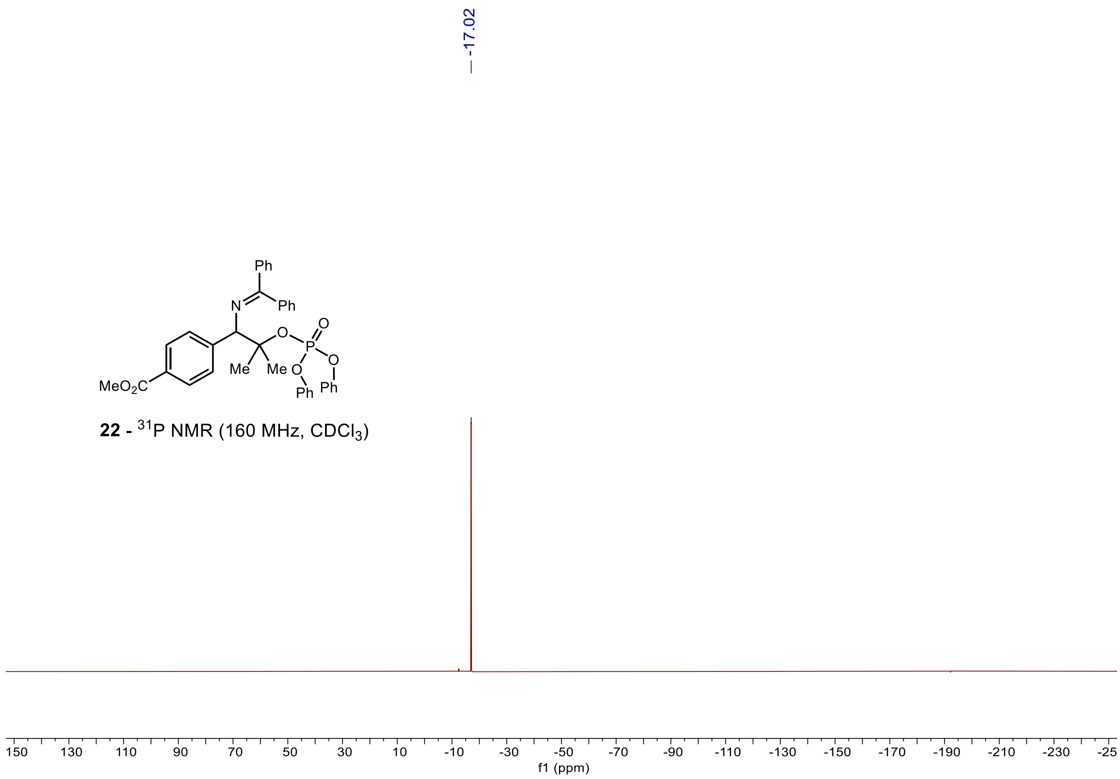

22 - ^{31}P NMR (160 MHz, $CDCl_3$)

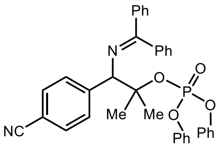

23 - ^{1}H NMR (400 MHz, $CDCl_3$)

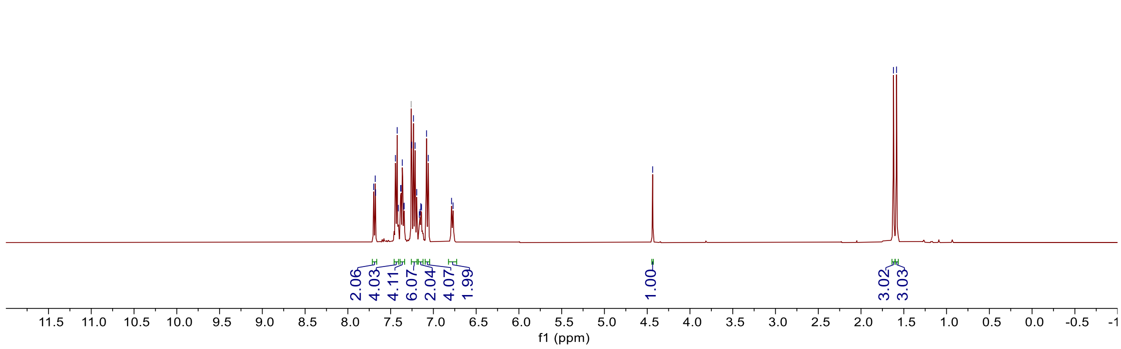

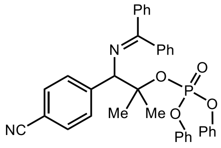

23 - ^{13}C NMR (100 MHz, $CDCl_3$)

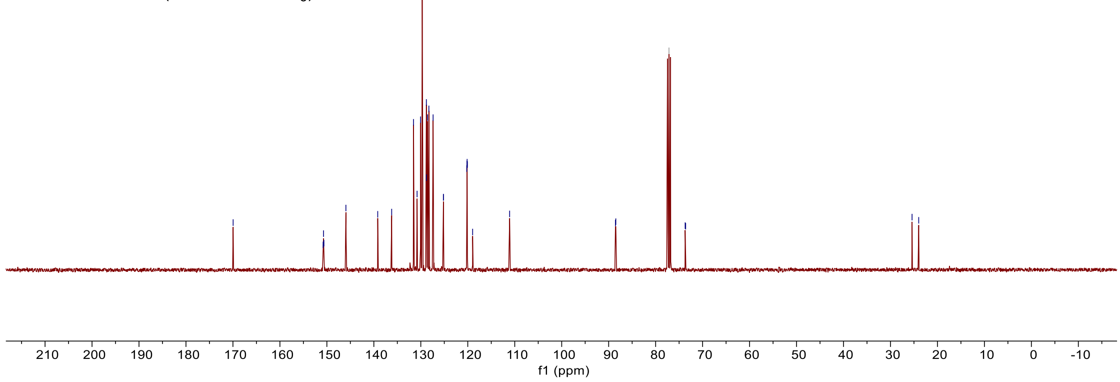

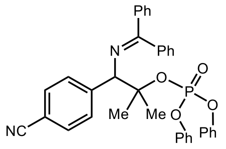

23 - ^{31}P NMR (160 MHz, $CDCl_3$)

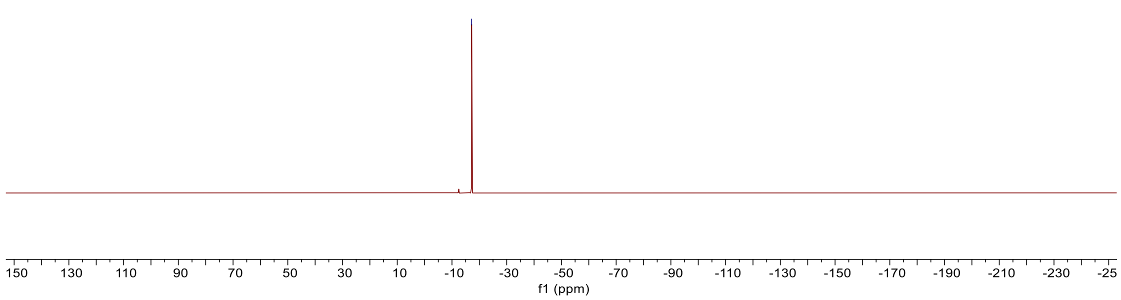

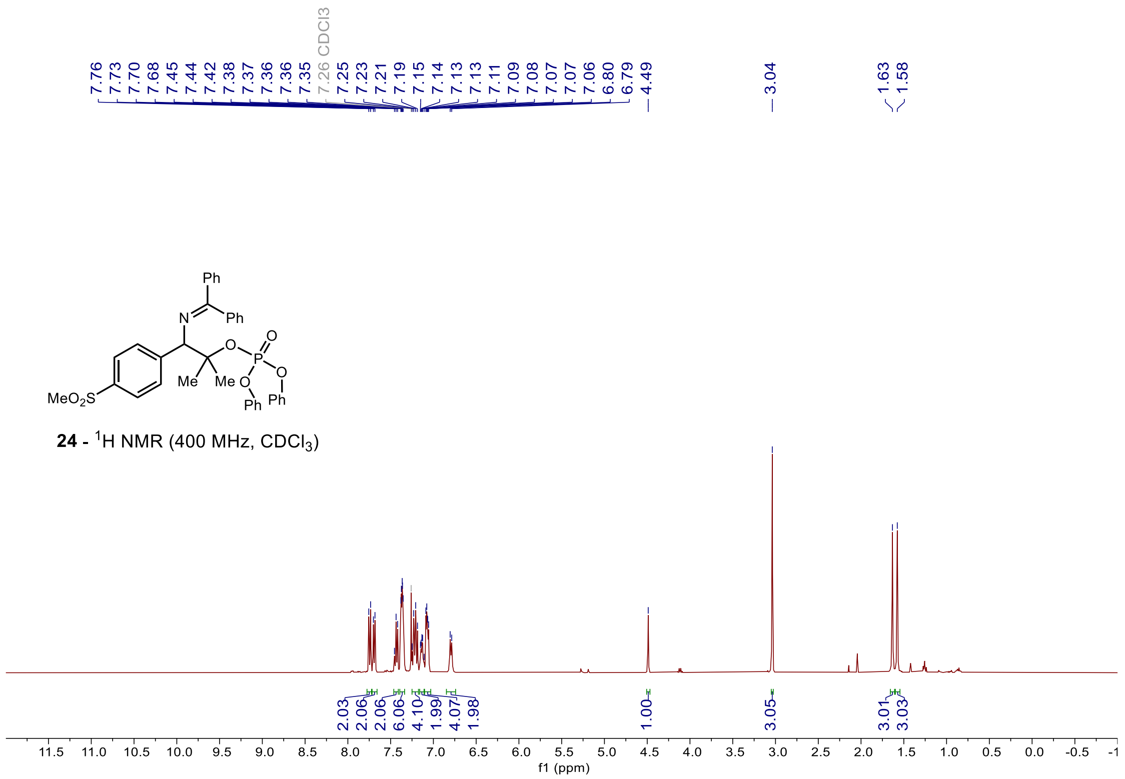

24 - ^{1}H NMR (400 MHz, $CDCl_3$)

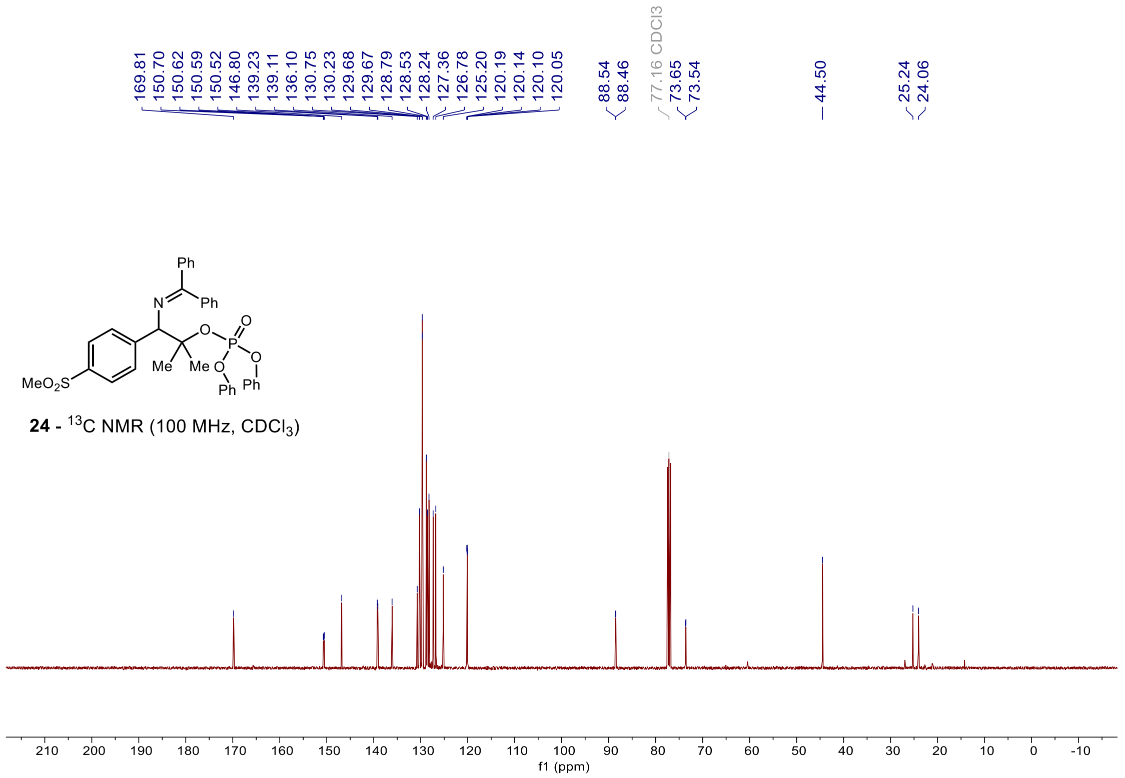

24 - ^{13}C NMR (100 MHz, $CDCl_3$)

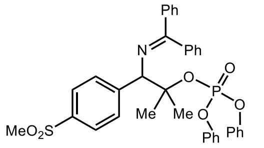

24 - ^{31}P NMR (160 MHz, $CDCl_3$)

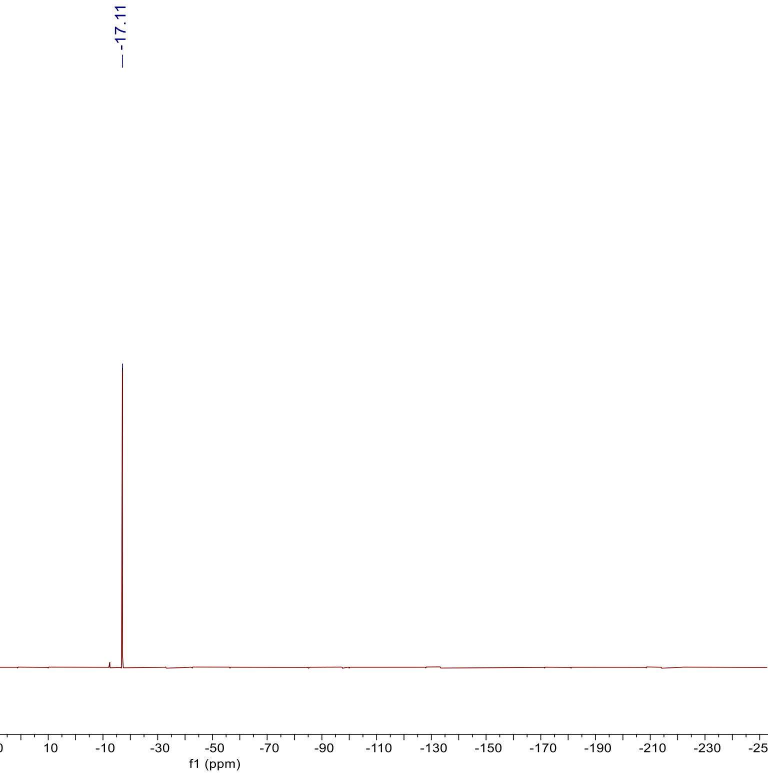

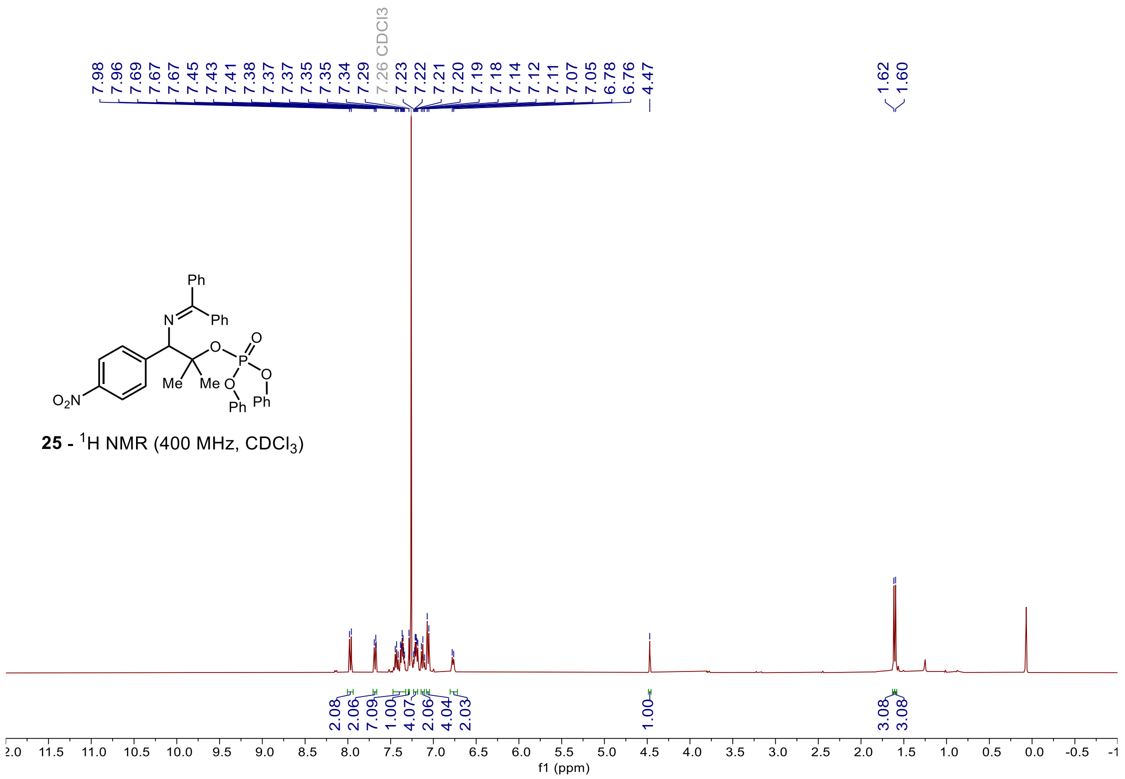

25 - ^{1}H NMR (400 MHz, $CDCl_3$)

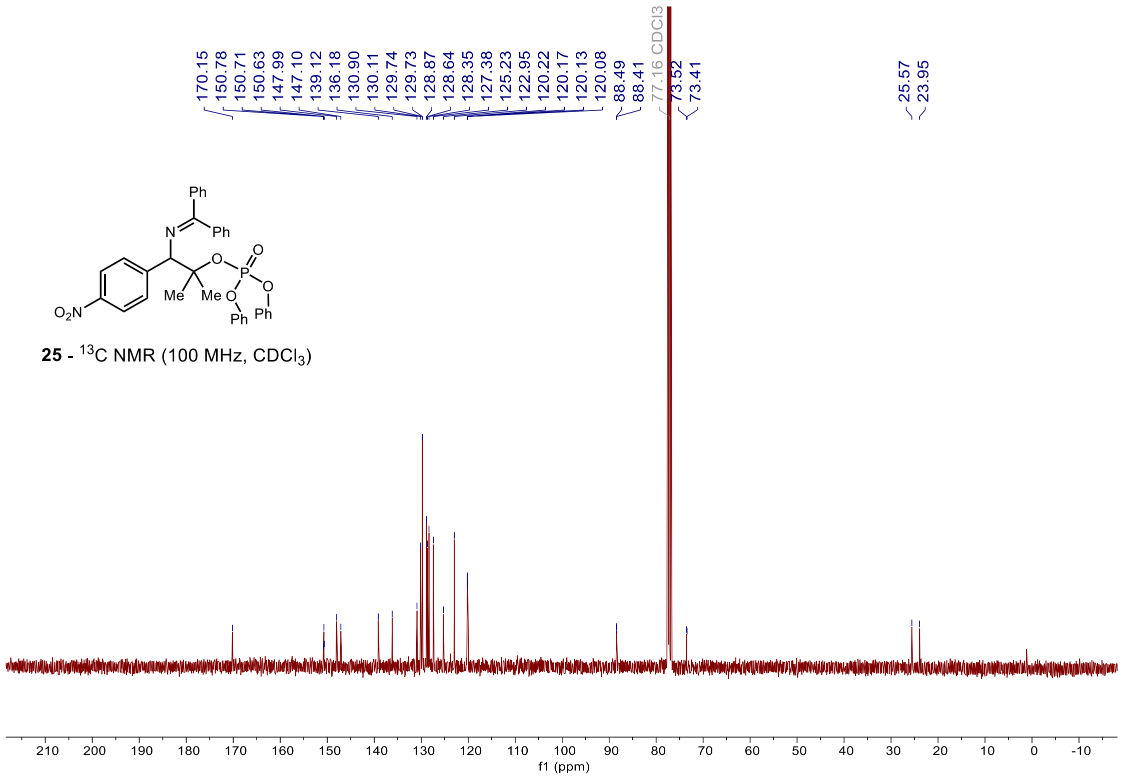

25 - ^{13}C NMR (100 MHz, $CDCl_3$)

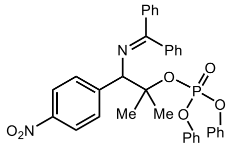

25 - ^{31}P NMR (160 MHz, $CDCl_3$)

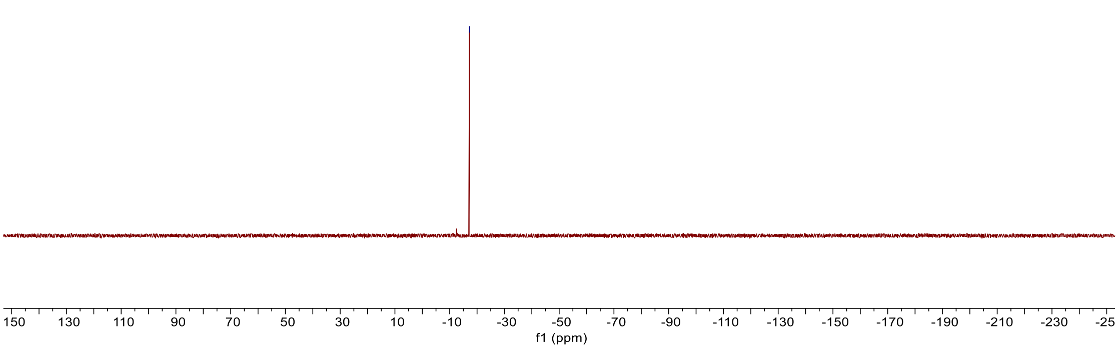

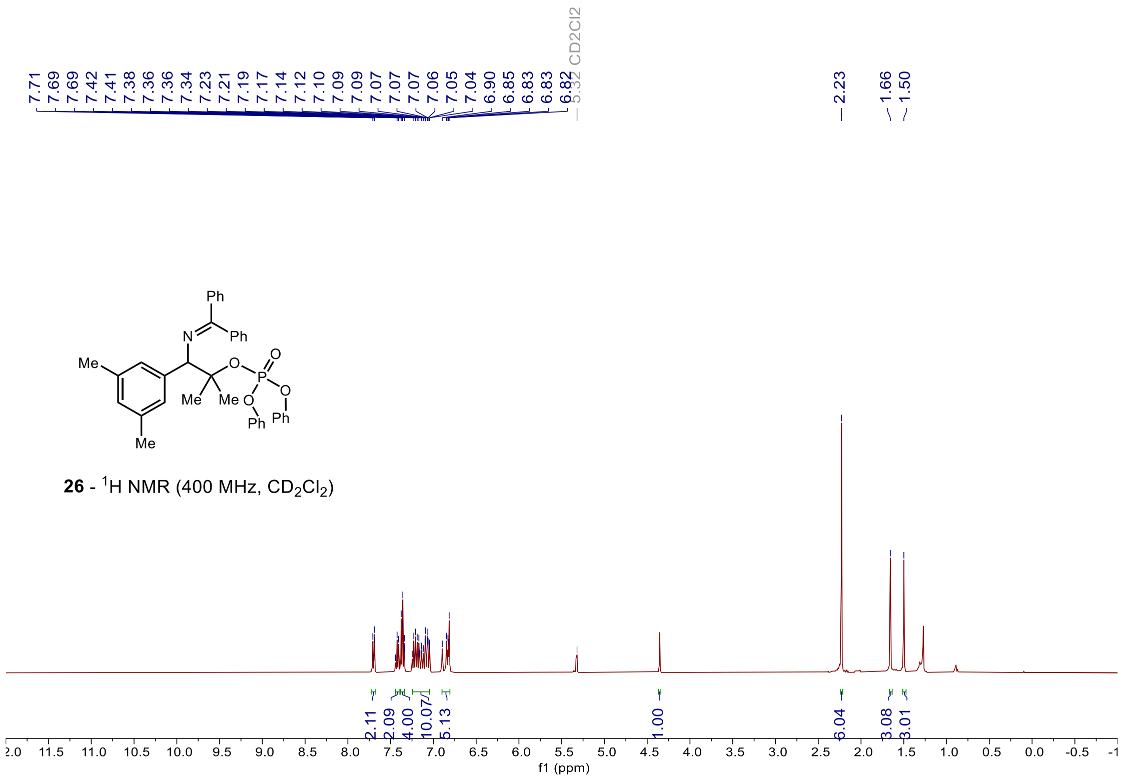

26 - ^{1}H NMR (400 MHz, CD_2Cl_2)

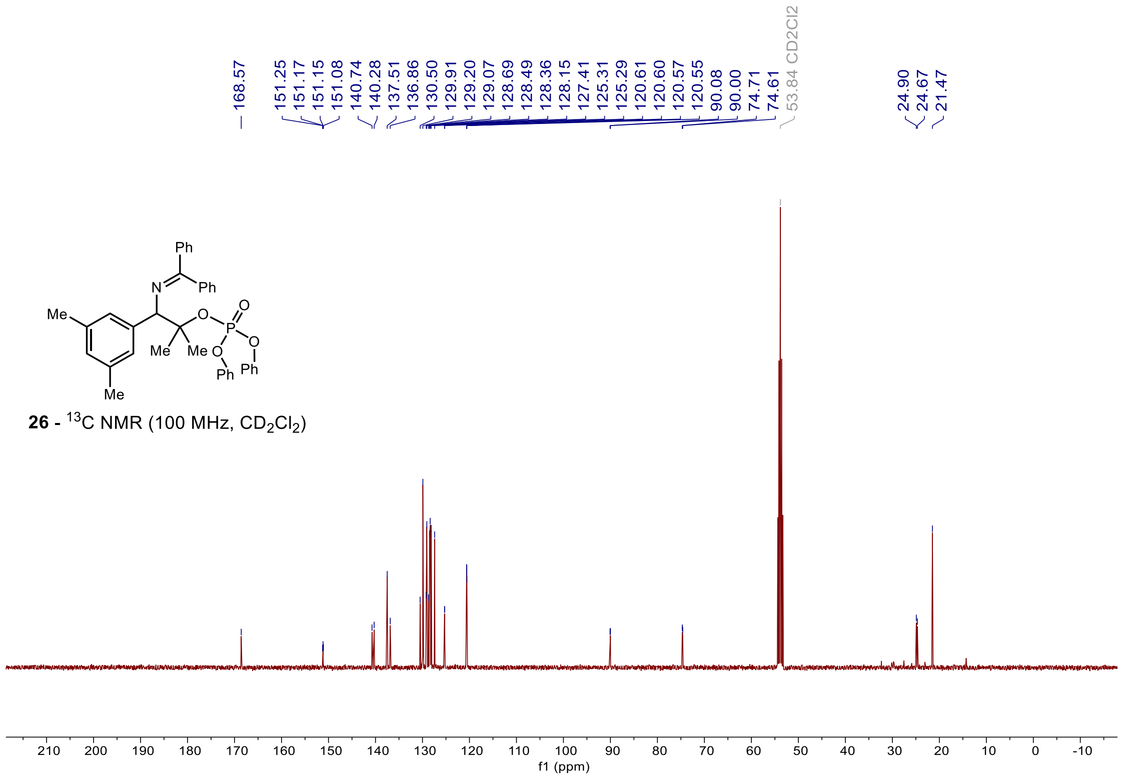

26 - ^{13}C NMR (100 MHz, CD_2Cl_2)

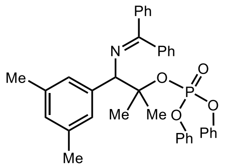

26 - ^{31}P NMR (160 MHz, CD_2Cl_2)

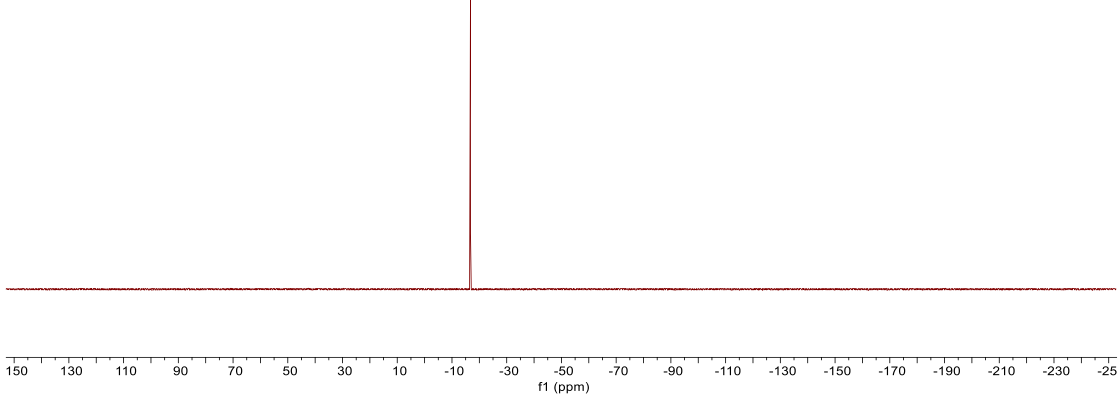

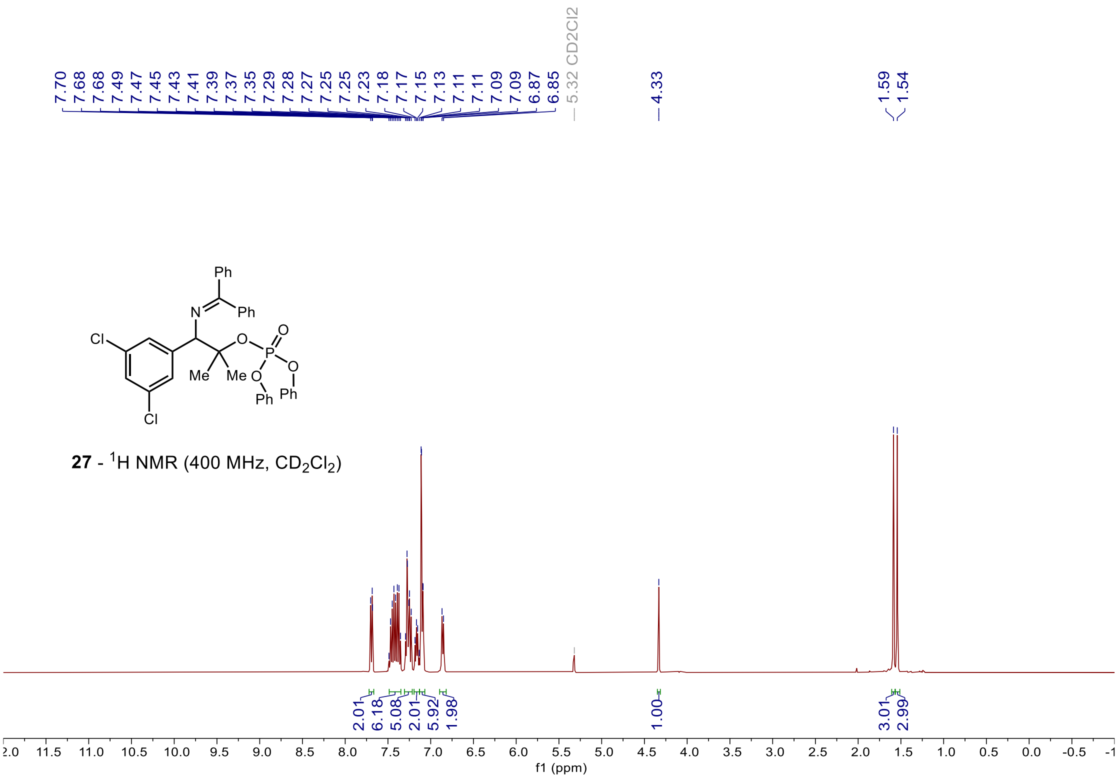

27 - ^{1}H NMR (400 MHz, CD_2Cl_2)

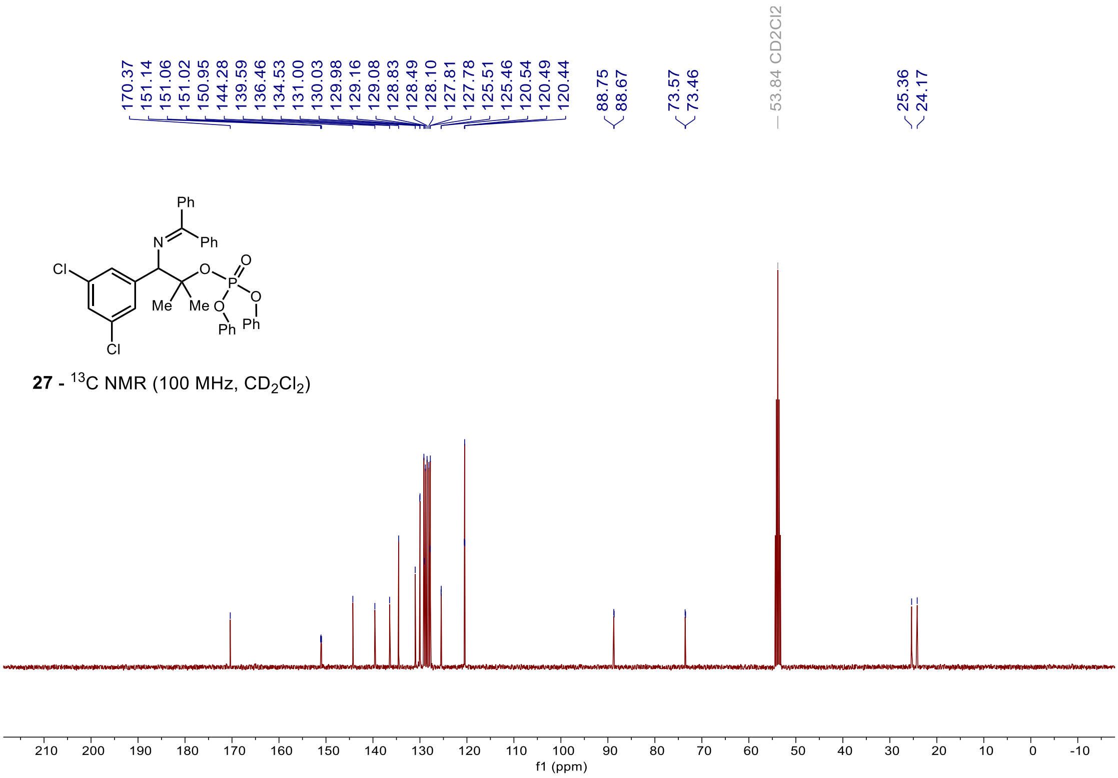

27 - ^{13}C NMR (100 MHz, CD_2Cl_2)

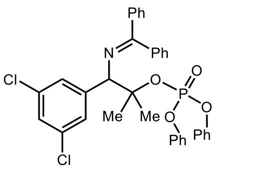

27 - ^{31}P NMR (160 MHz, $CDCl_3$)

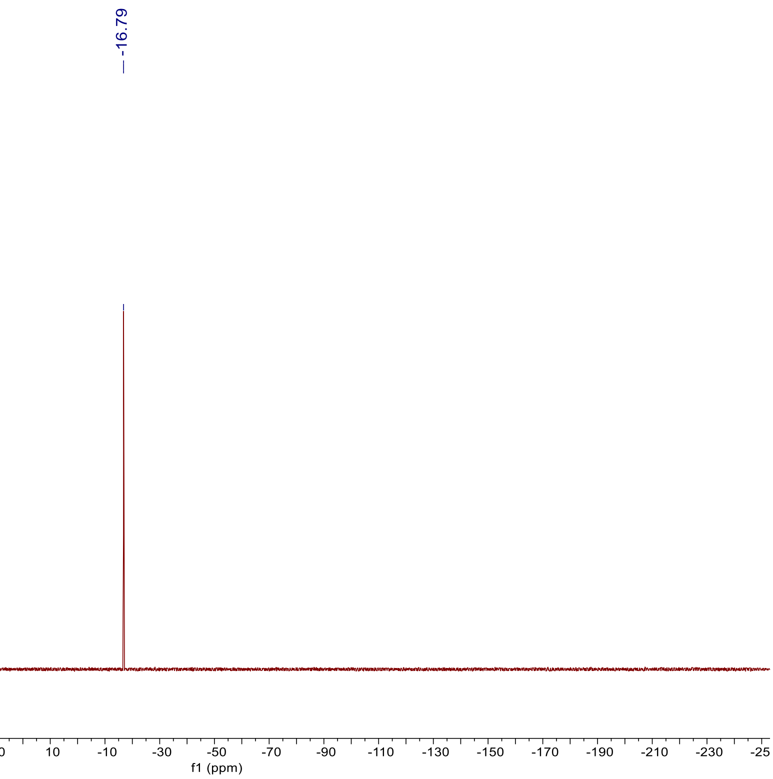

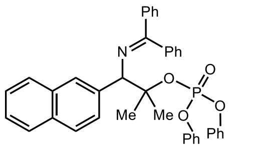

28 - ^{1}H NMR (400 MHz, $CDCl_3$)

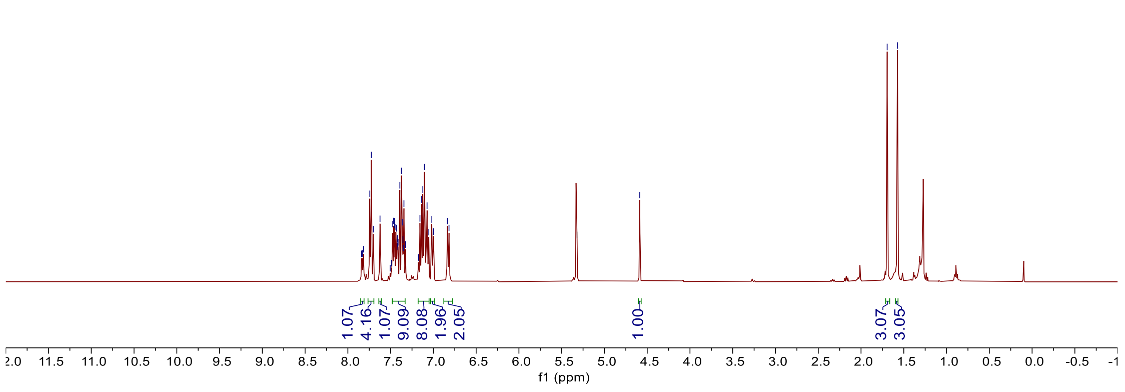

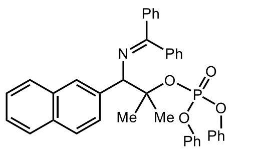

28 - ^{13}C NMR (100 MHz, $CDCl_3$)

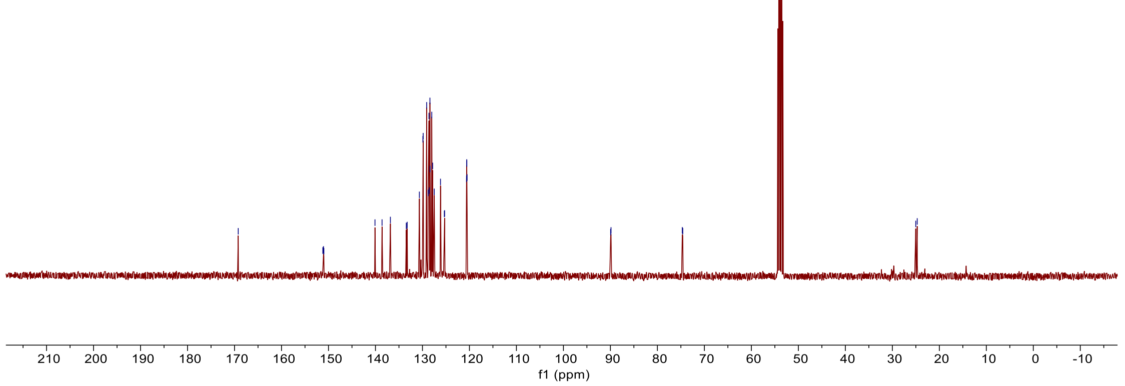

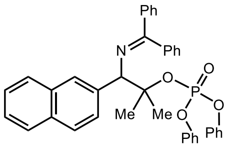

28 - ^{31}P NMR (160 MHz, $CDCl_3$)

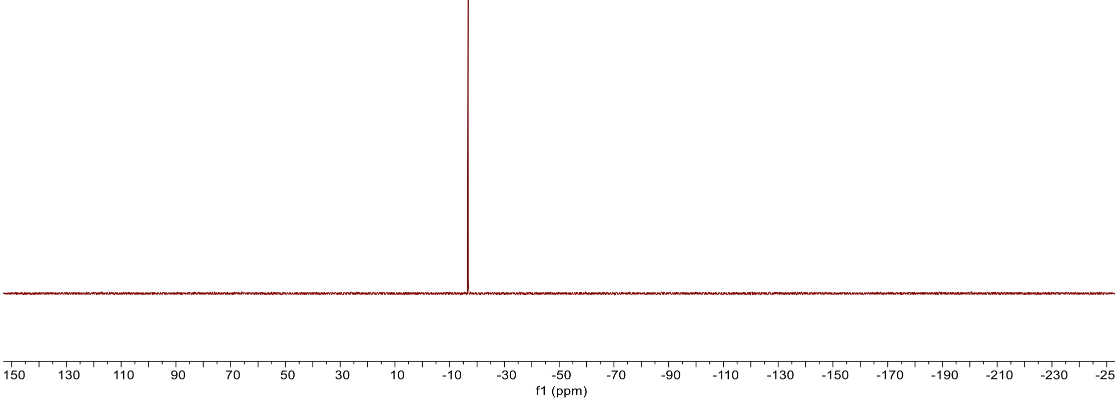

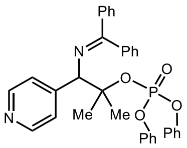

29 - ^{1}H NMR (400 MHz, CD_2Cl_2)

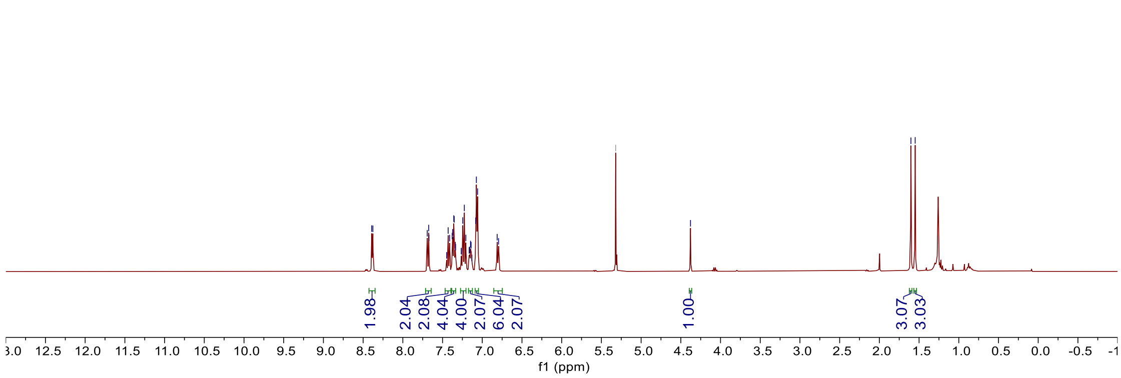

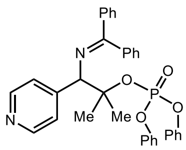

29 - ^{13}C NMR (100 MHz, CD_2Cl_2)

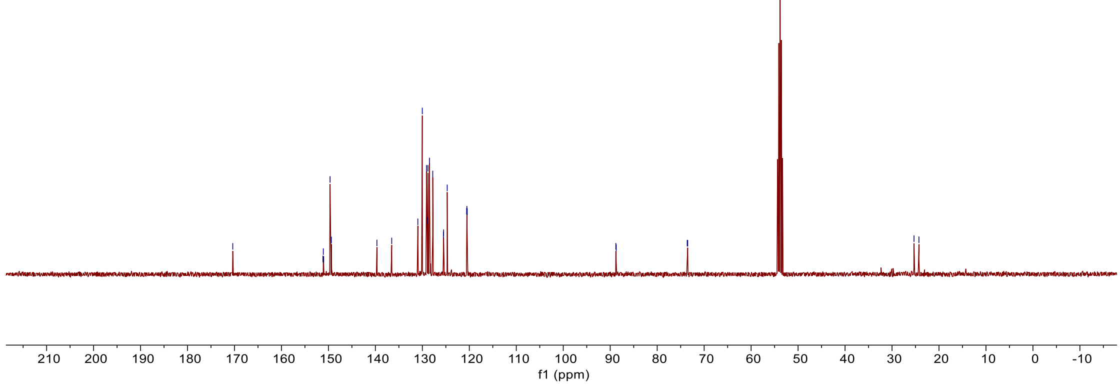

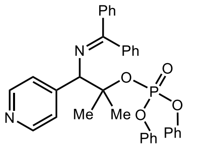

29 - ^{31}P NMR (160 MHz, CD_2Cl_2)

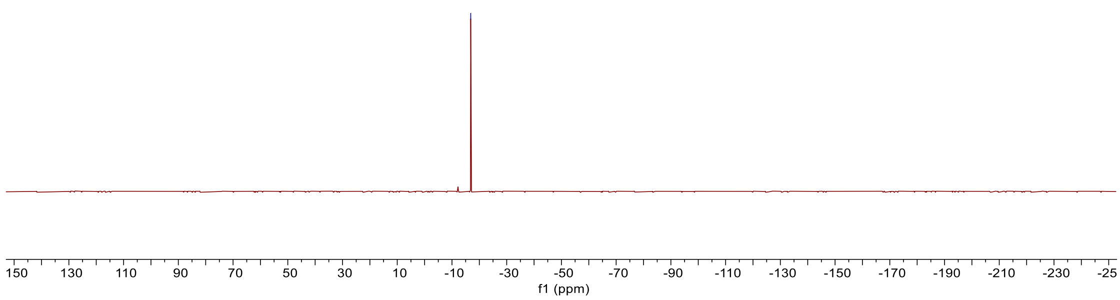

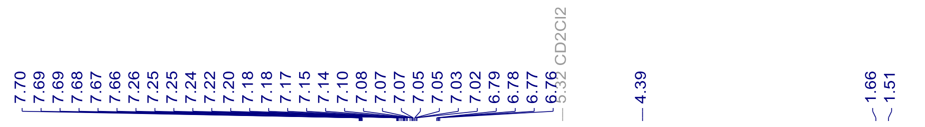

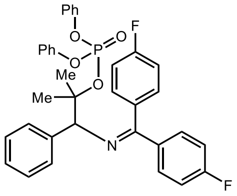

30 - ^{1}H NMR (400 MHz, CD_2Cl_2)

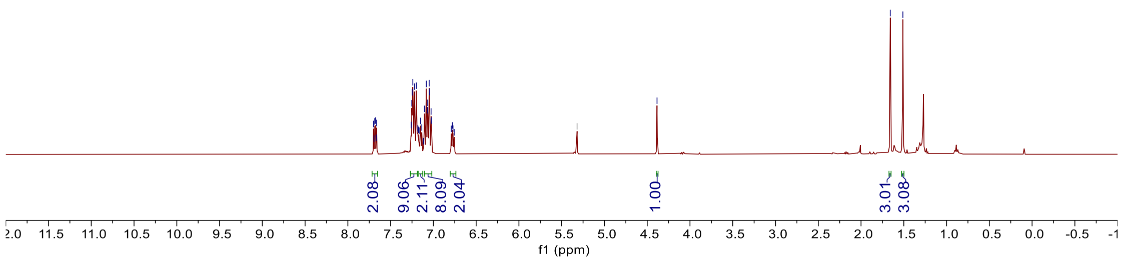

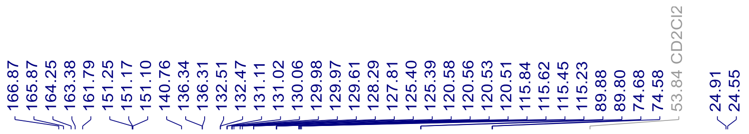

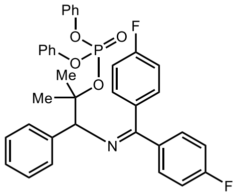

30 - ^{13}C NMR (100 MHz, CD_2Cl_2)

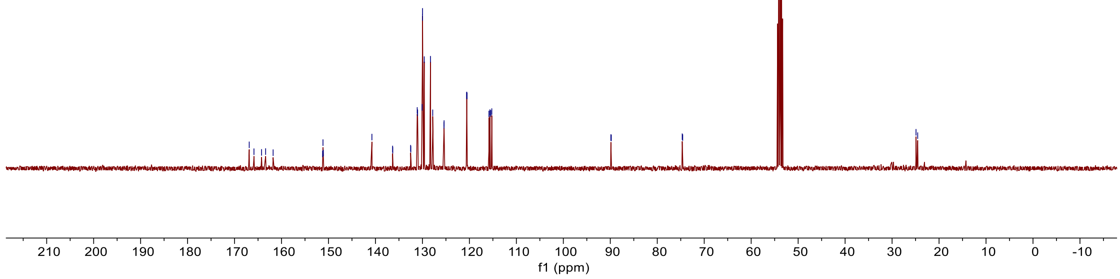

30 - ^{31}P NMR (160 MHz, CD_2Cl_2)

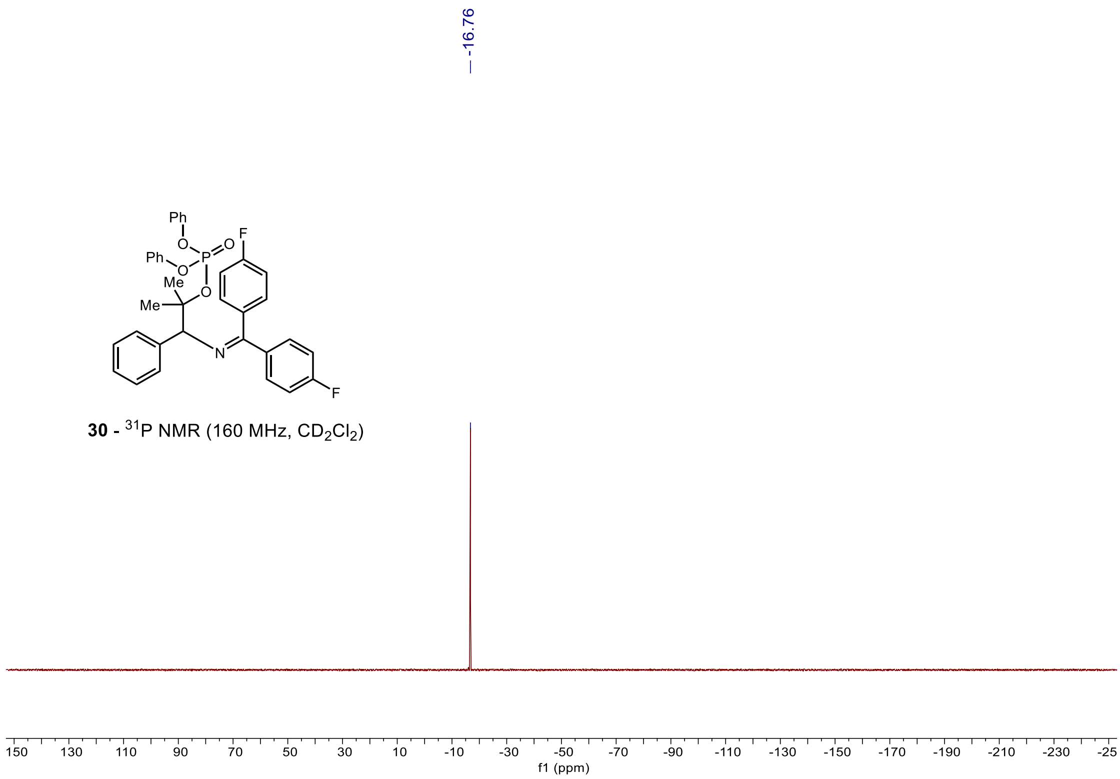

30 - ^{19}F NMR (376 MHz, CD_2Cl_2)

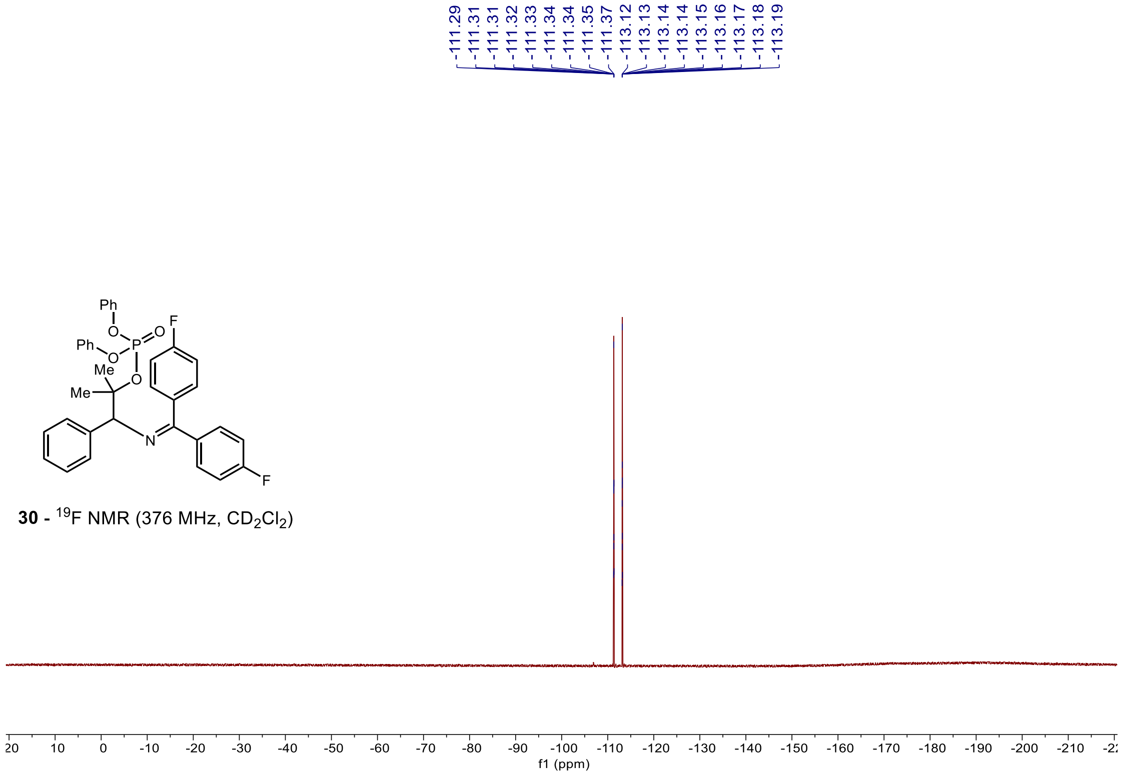

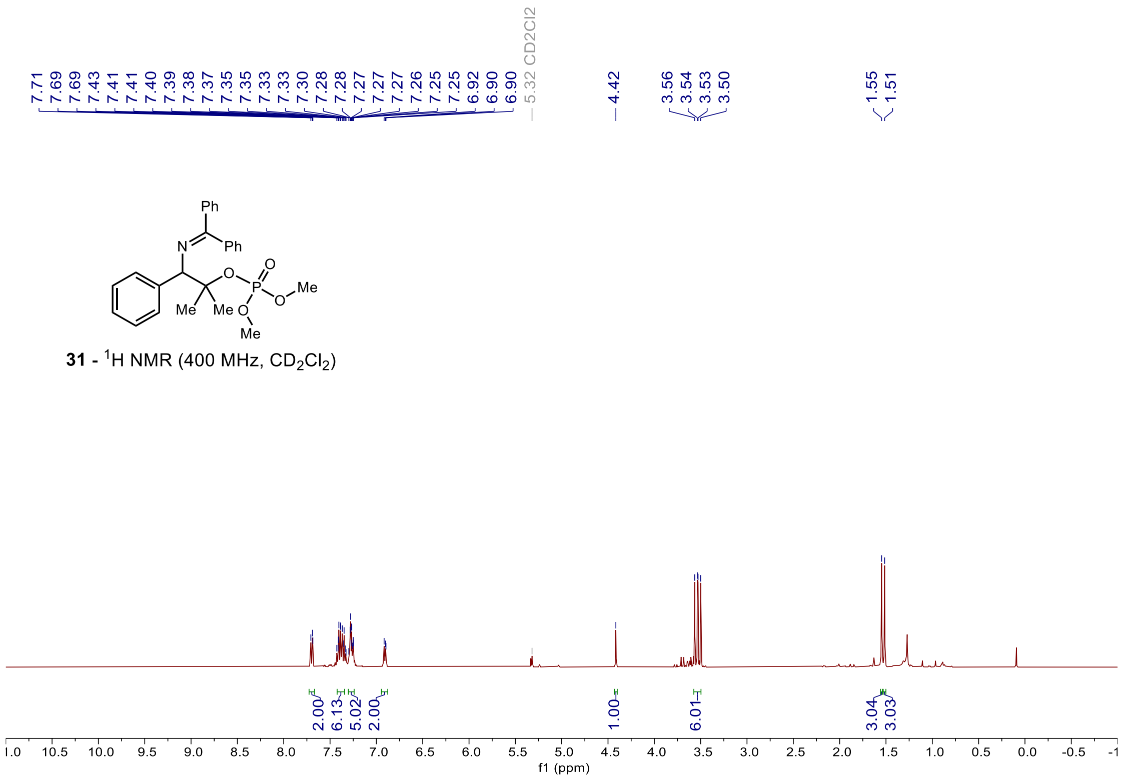

31 - ^{1}H NMR (400 MHz, CD_2Cl_2)

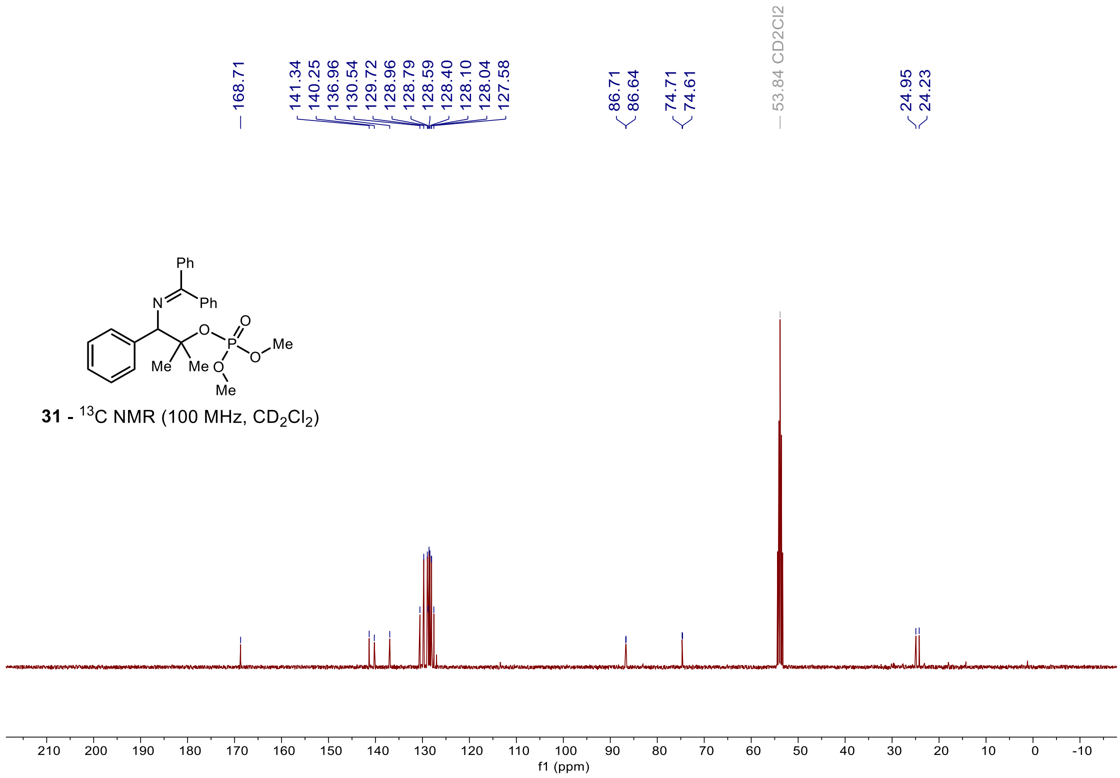

31 - ^{13}C NMR (100 MHz, CD_2Cl_2)

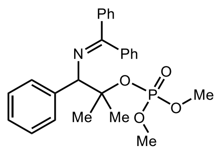

31 - ^{31}P NMR (160 MHz, CD_2Cl_2)

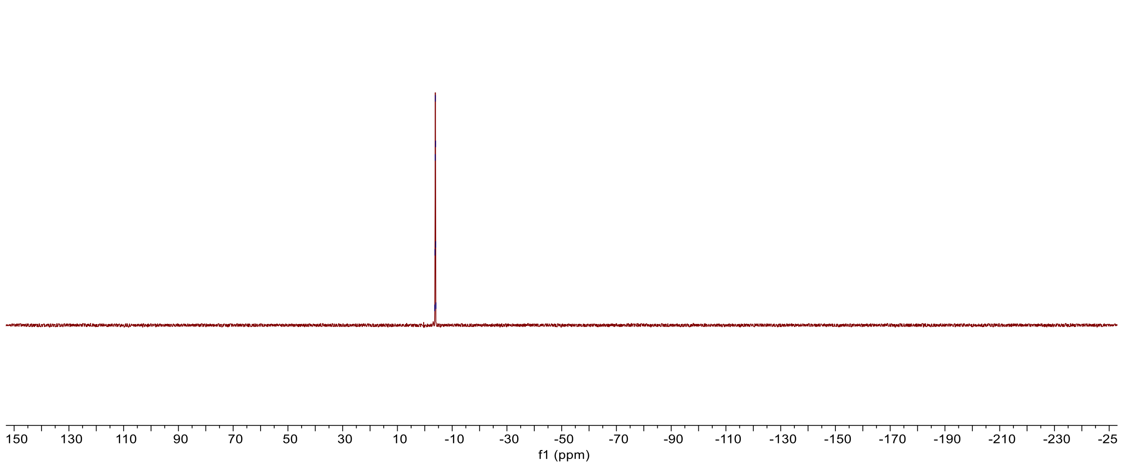

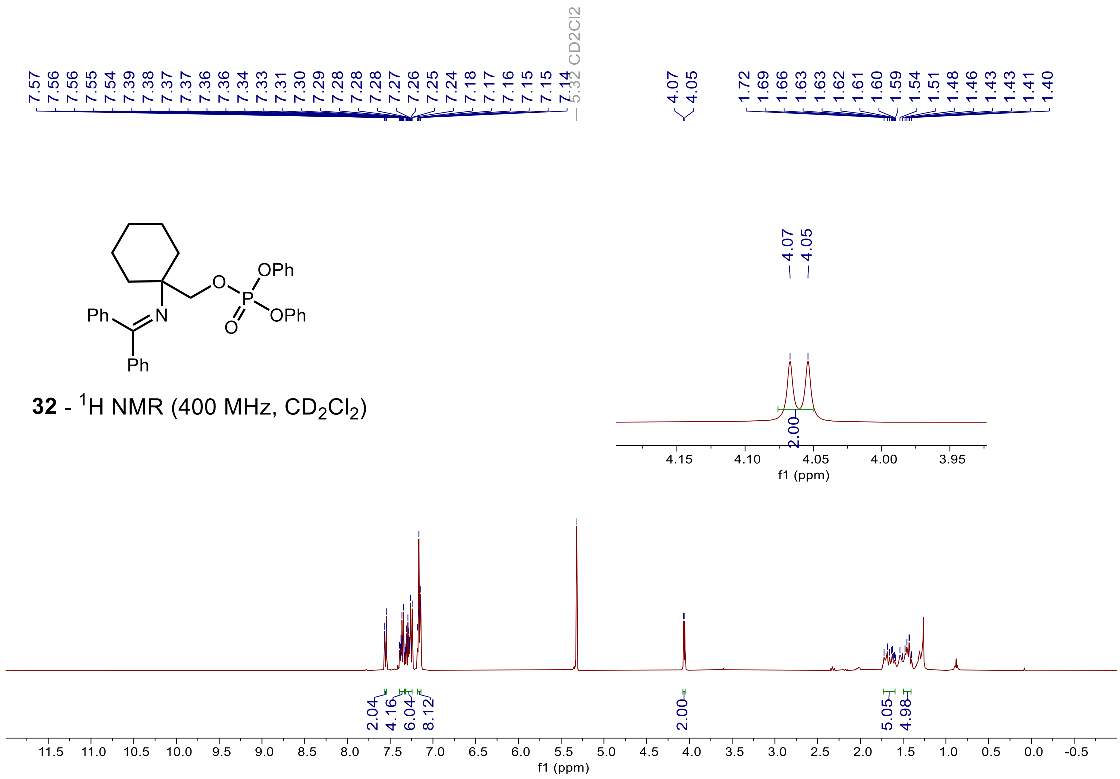

32 - ^{1}H NMR (400 MHz, CD_2Cl_2)

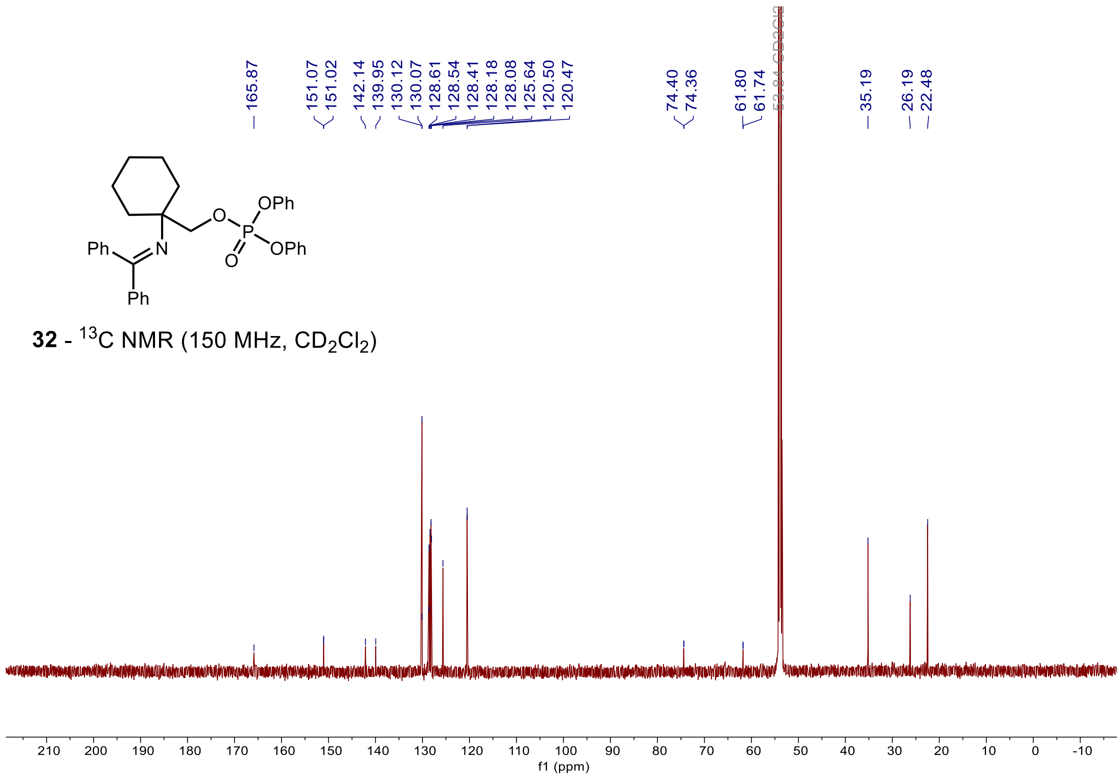

32 - ^{13}C NMR (150 MHz, CD_2Cl_2)

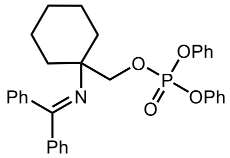

32 - ^{31}P NMR (160 MHz, CD_2Cl_2)

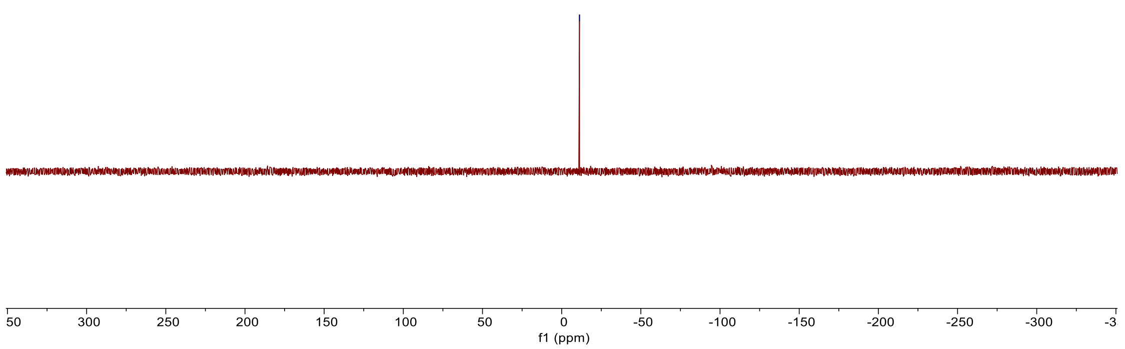

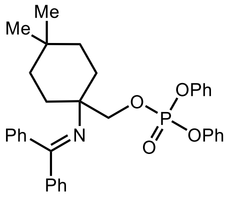

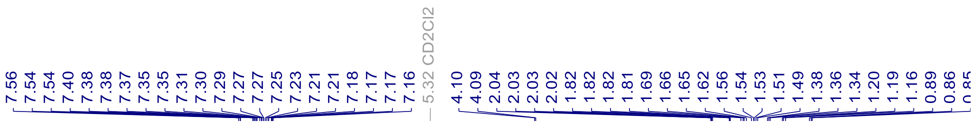
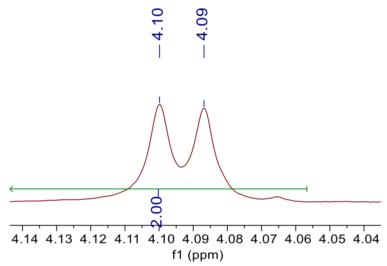
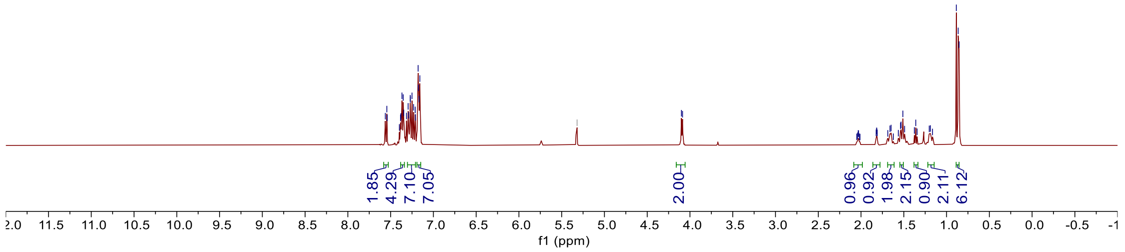

33 - ^{1}H NMR (400 MHz, CD_2Cl_2)

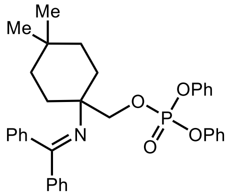

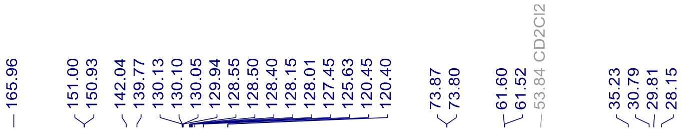
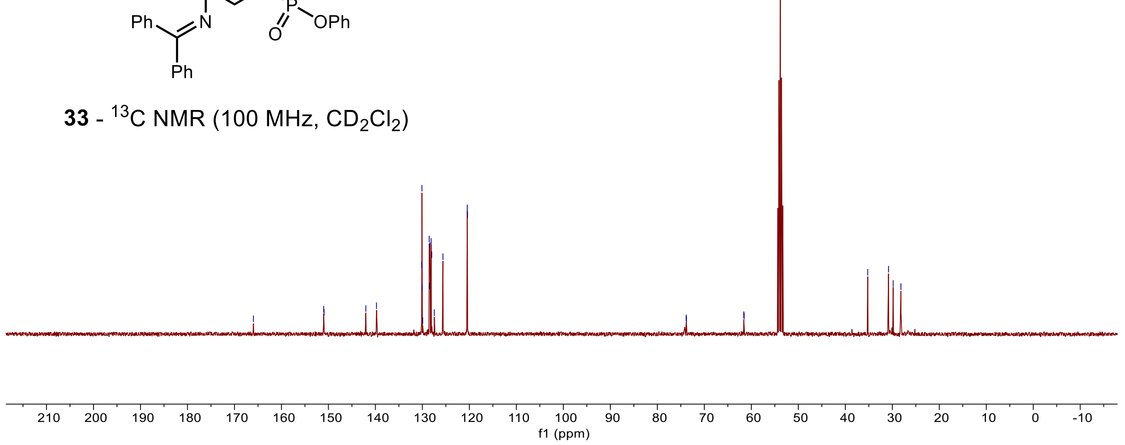

33 - ^{13}C NMR (100 MHz, CD_2Cl_2)

-11.81

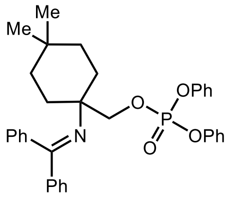

33 - ^{31}P NMR (160 MHz, CD_2Cl_2)

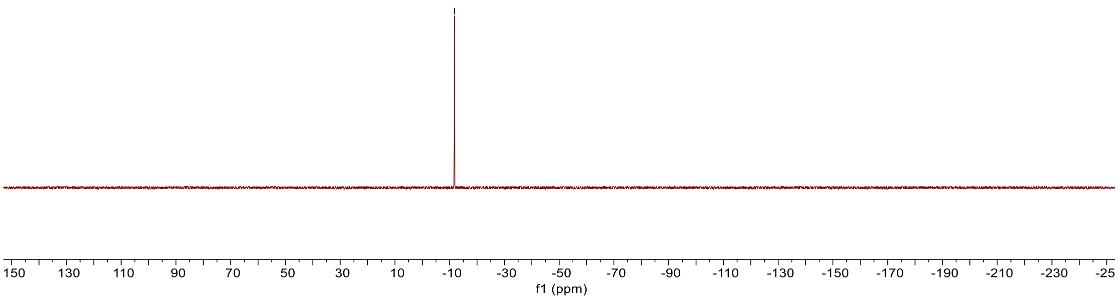

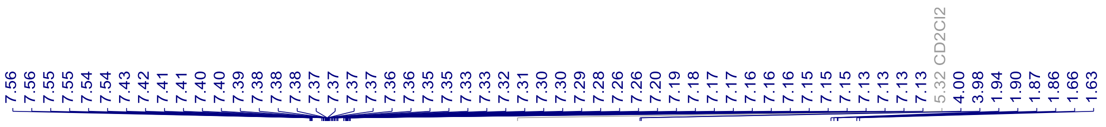

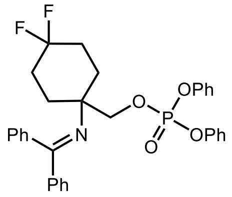

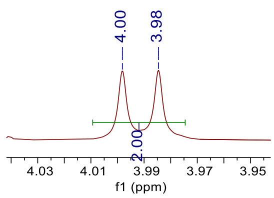

34 - ^{1}H NMR (400 MHz, CD_2Cl_2)

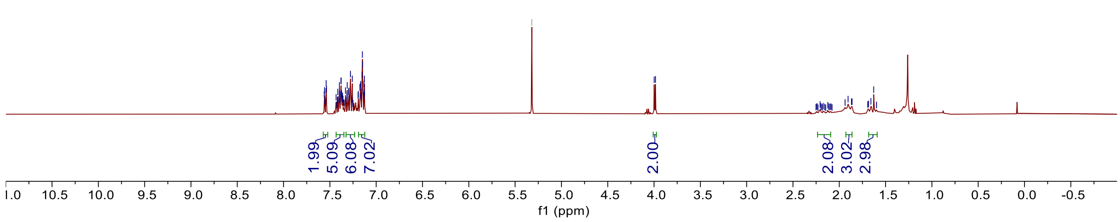

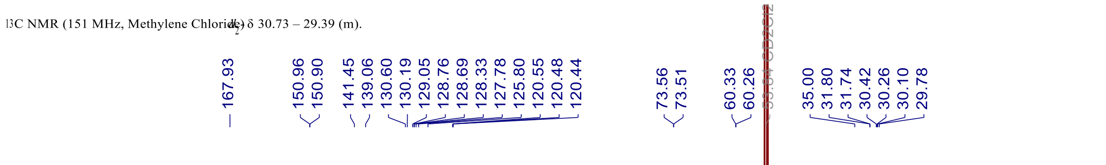

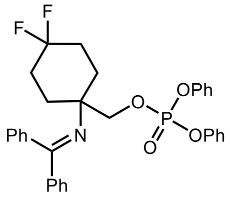

34 - ^{13}C NMR (150 MHz, CD_2Cl_2)

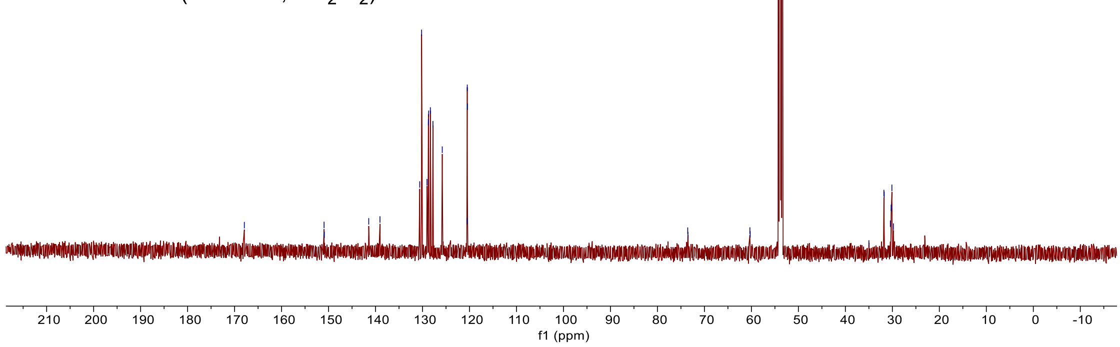

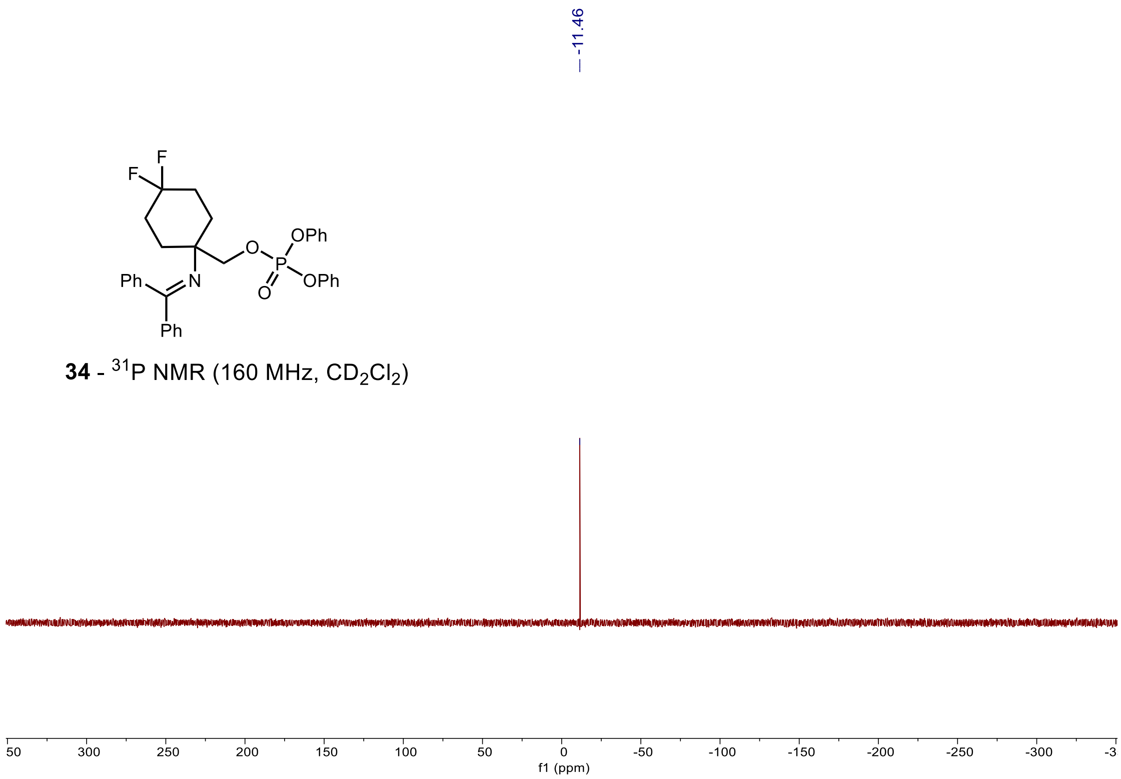

34 - ^{31}P NMR (160 MHz, CD_2Cl_2)

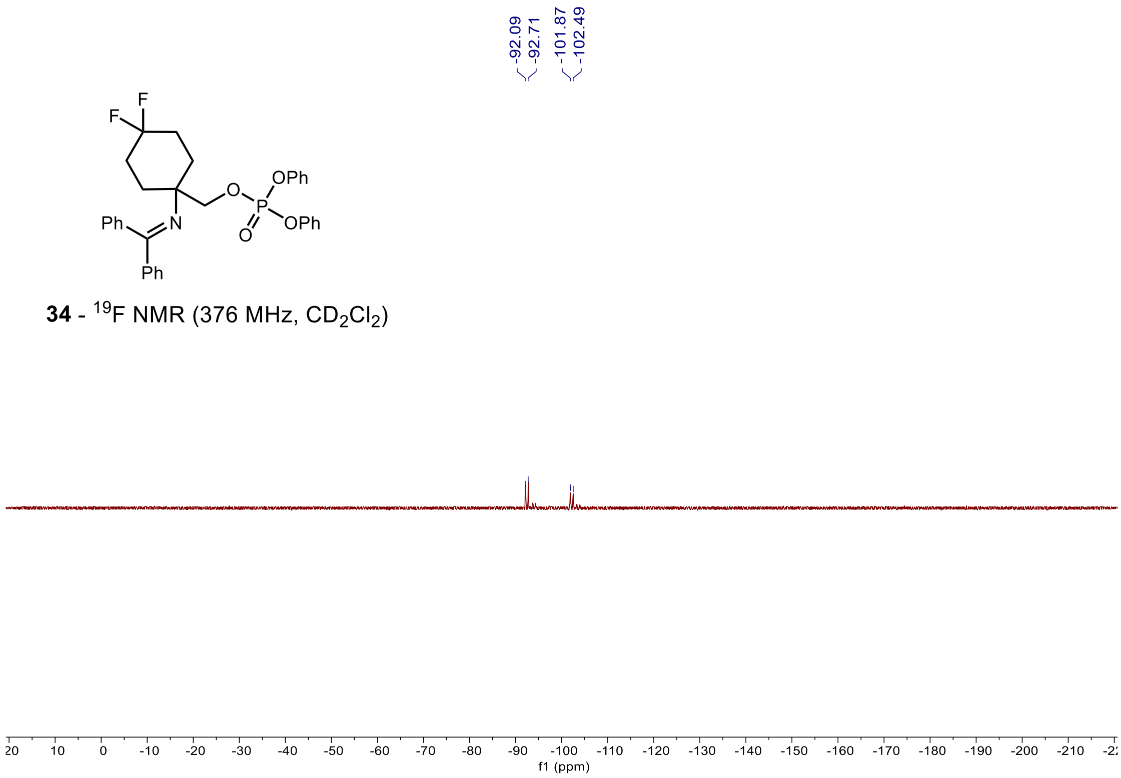

34 - ^{19}F NMR (376 MHz, CD_2Cl_2)

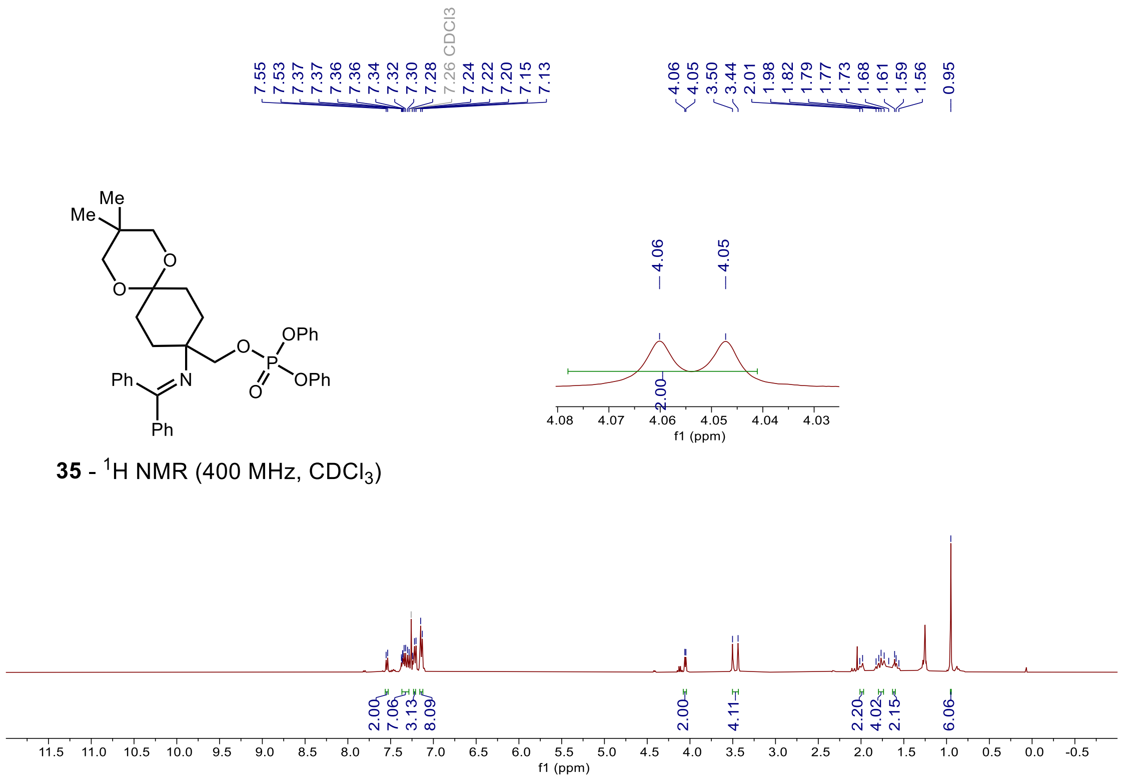

35 - ^{1}H NMR (400 MHz, $CDCl_3$)

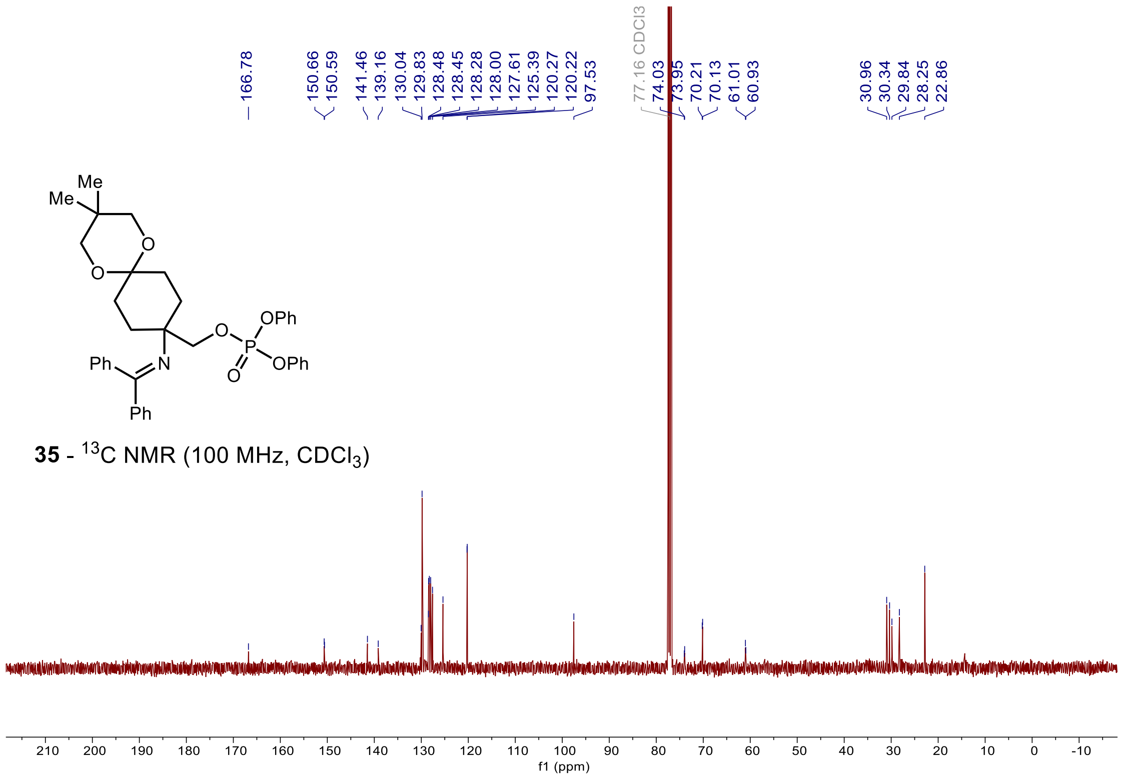

35 - ^{13}C NMR (100 MHz, $CDCl_3$)

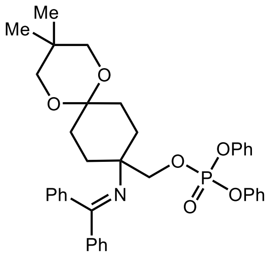

35 - ^{31}P NMR (400 MHz, $CDCl_3$)

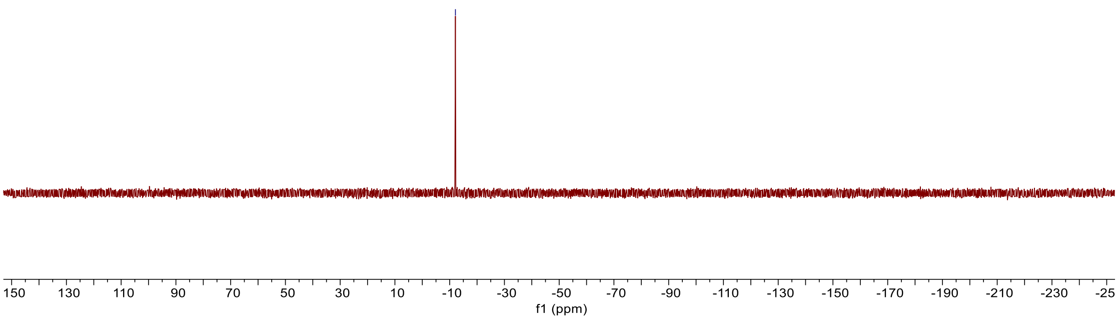

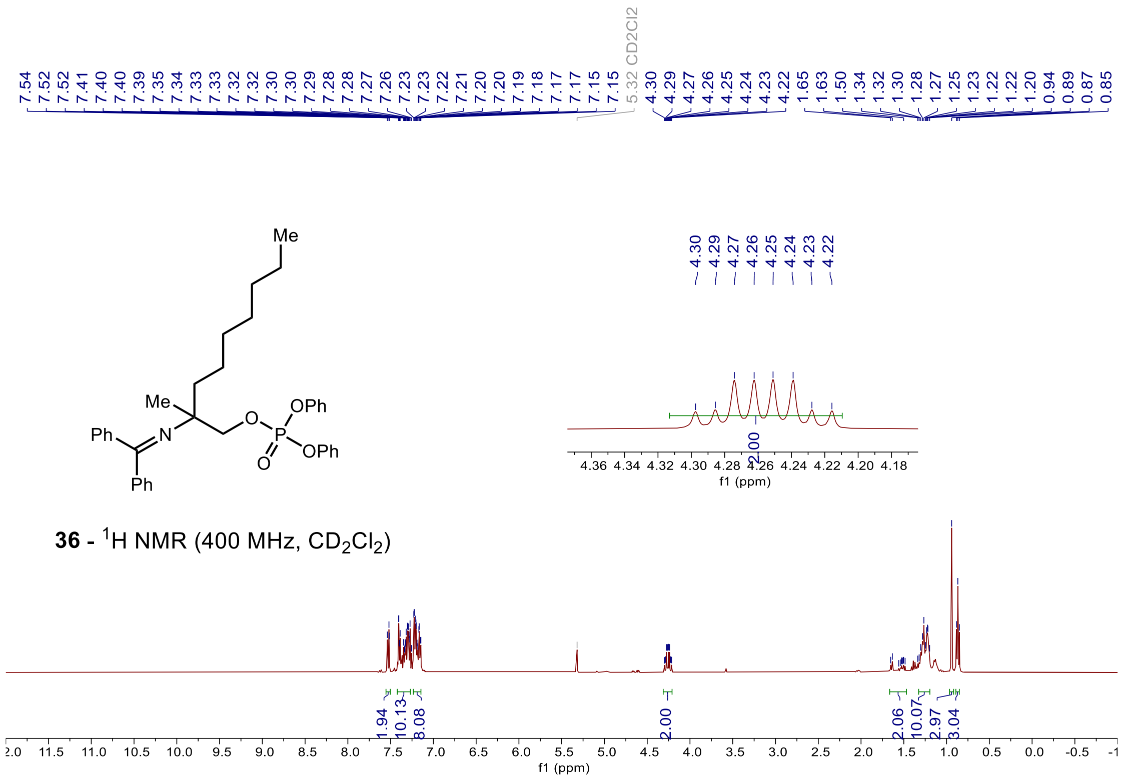

36 - ^{1}H NMR (400 MHz, CD_2Cl_2)

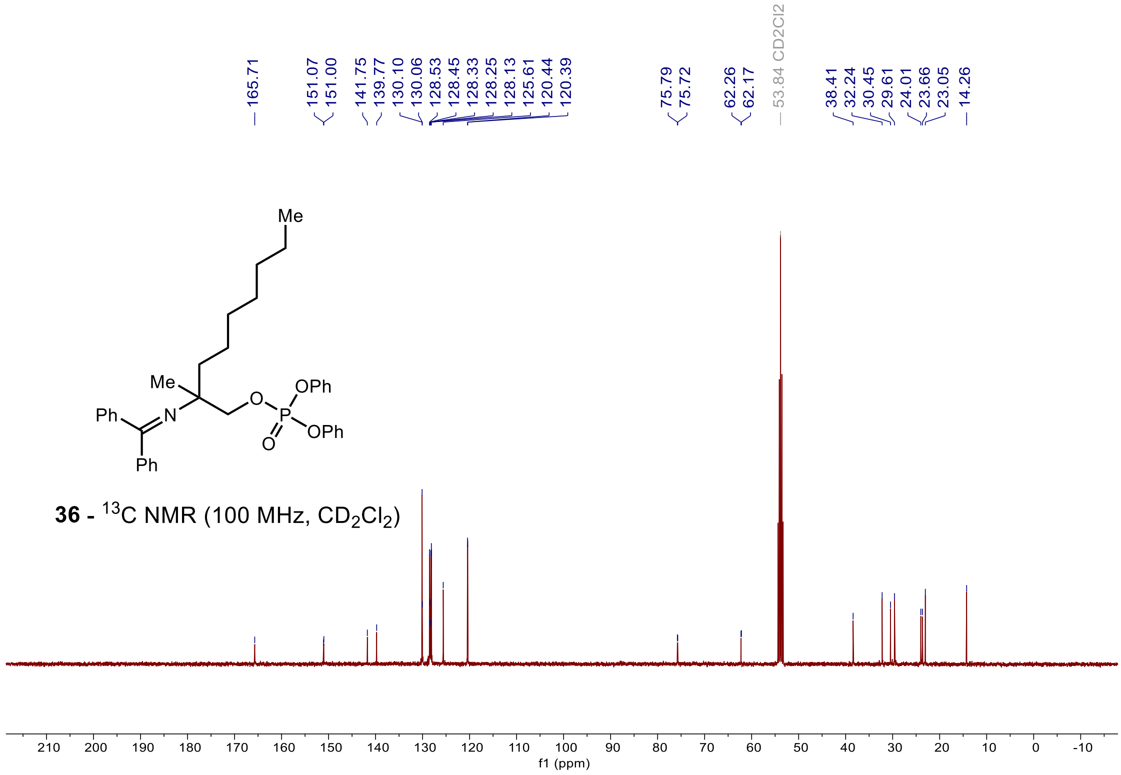

36 - ^{13}C NMR (100 MHz, CD_2Cl_2)

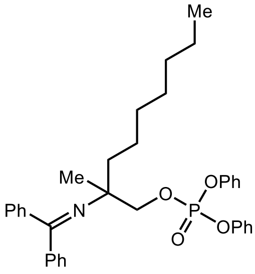

36 - ^{31}P NMR (160 MHz, CD_2Cl_2)

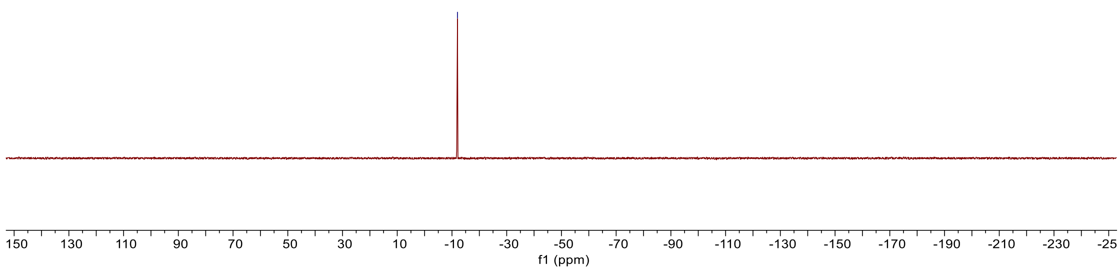

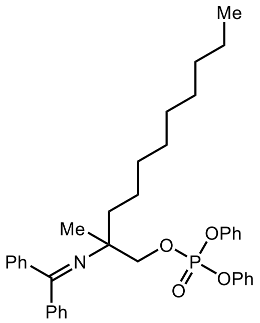

37 - ^{1}H NMR (400 MHz, CD_2Cl_2)

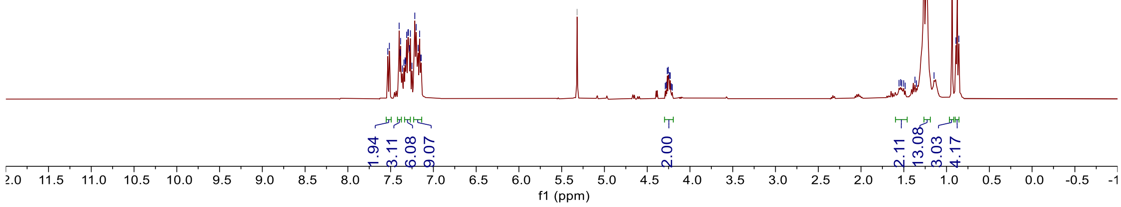

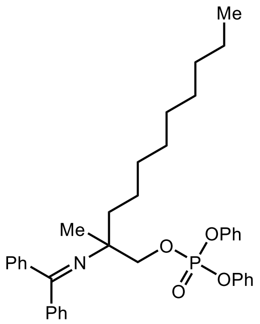

37 - ^{13}C NMR (150 MHz, CD_2Cl_2)

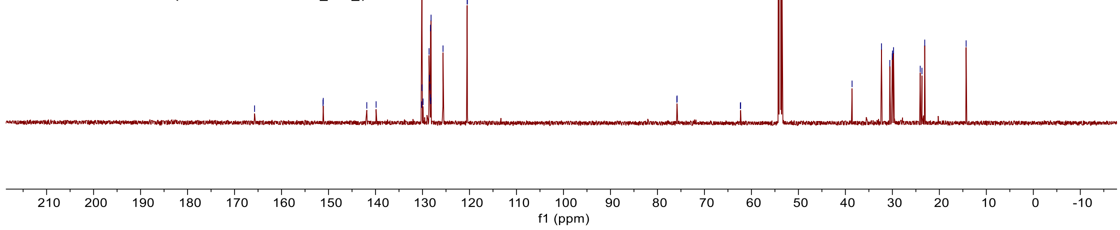

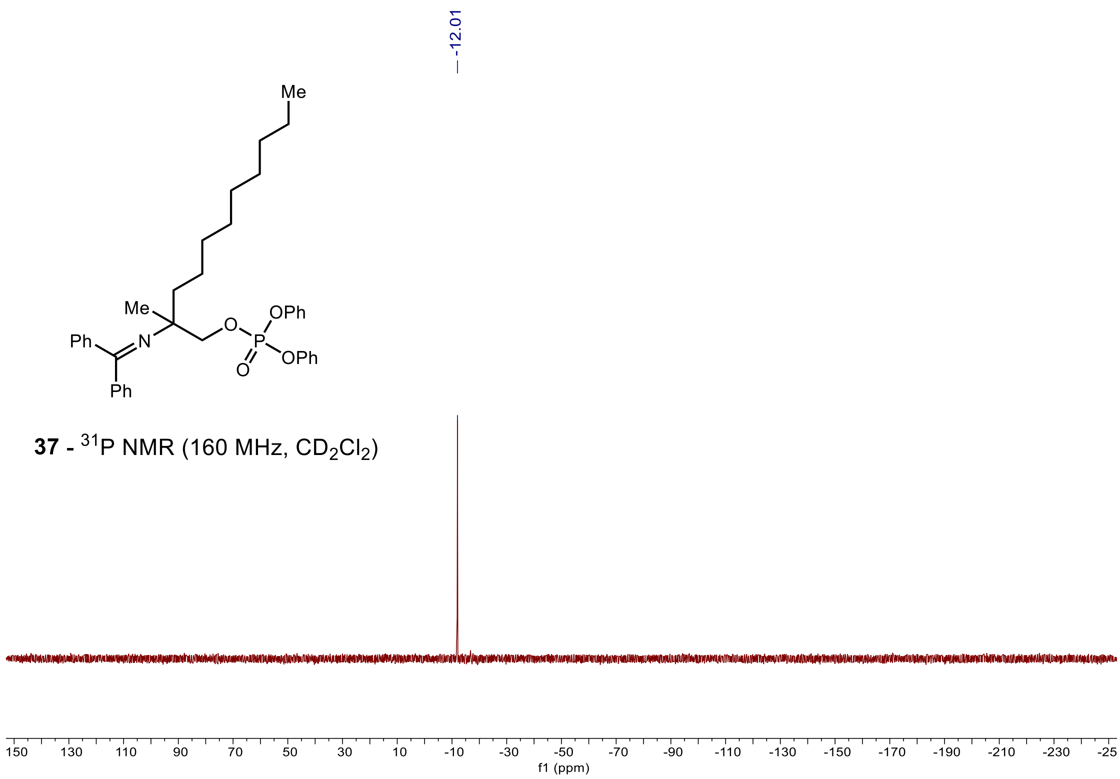
-12.01
Me
Me
Ph
N
Ph
O
OPh
P
O
OPh
37 - 31P NMR (160 MHz, CD2Cl2)
150
130
110
90
70
50
30
10
-10
-30
-50
-70
-90
-110
-130
-150
-170
-190
-210
-230
-25
f1 (ppm)

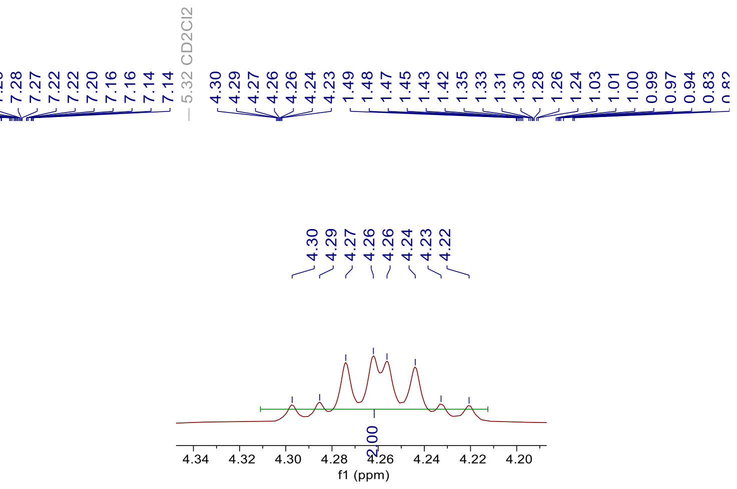

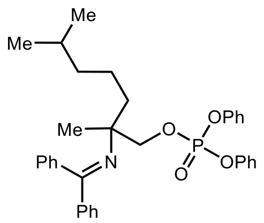

38 - ^{1}H NMR (400 MHz, CD_2Cl_2)

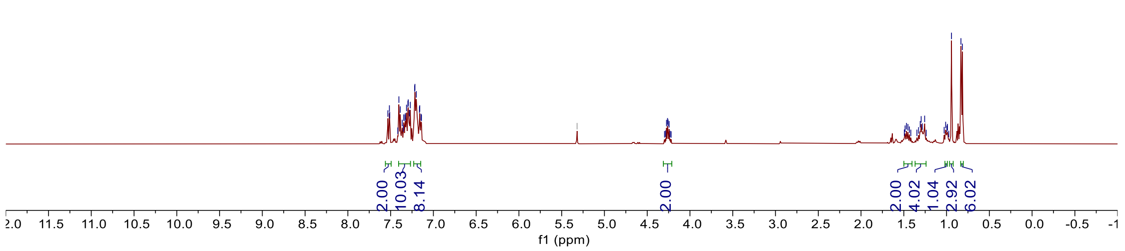

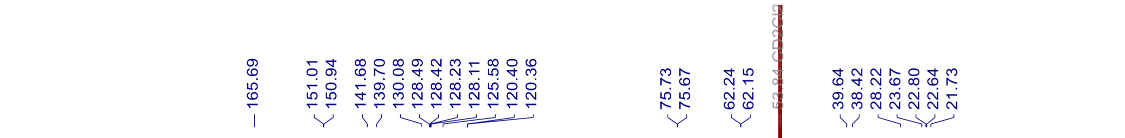

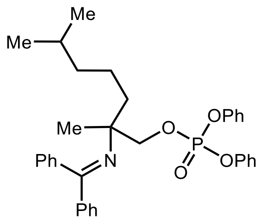

38 - ^{13}C NMR (100 MHz, CD_2Cl_2)

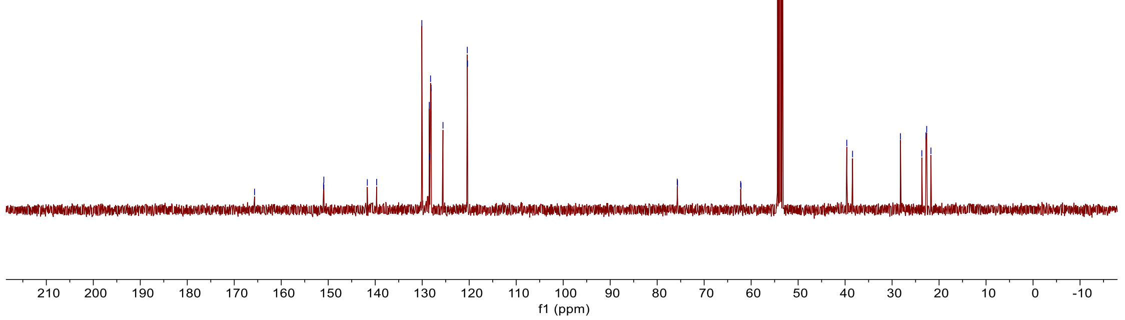

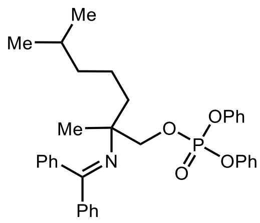

38 - ^{31}P NMR (160 MHz, CD_2Cl_2)

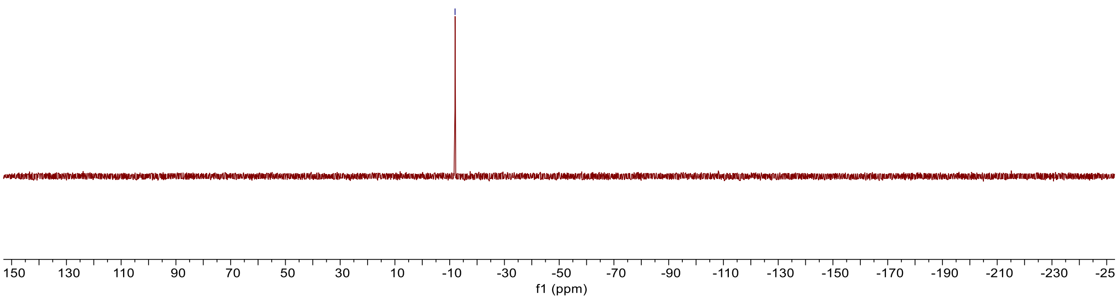

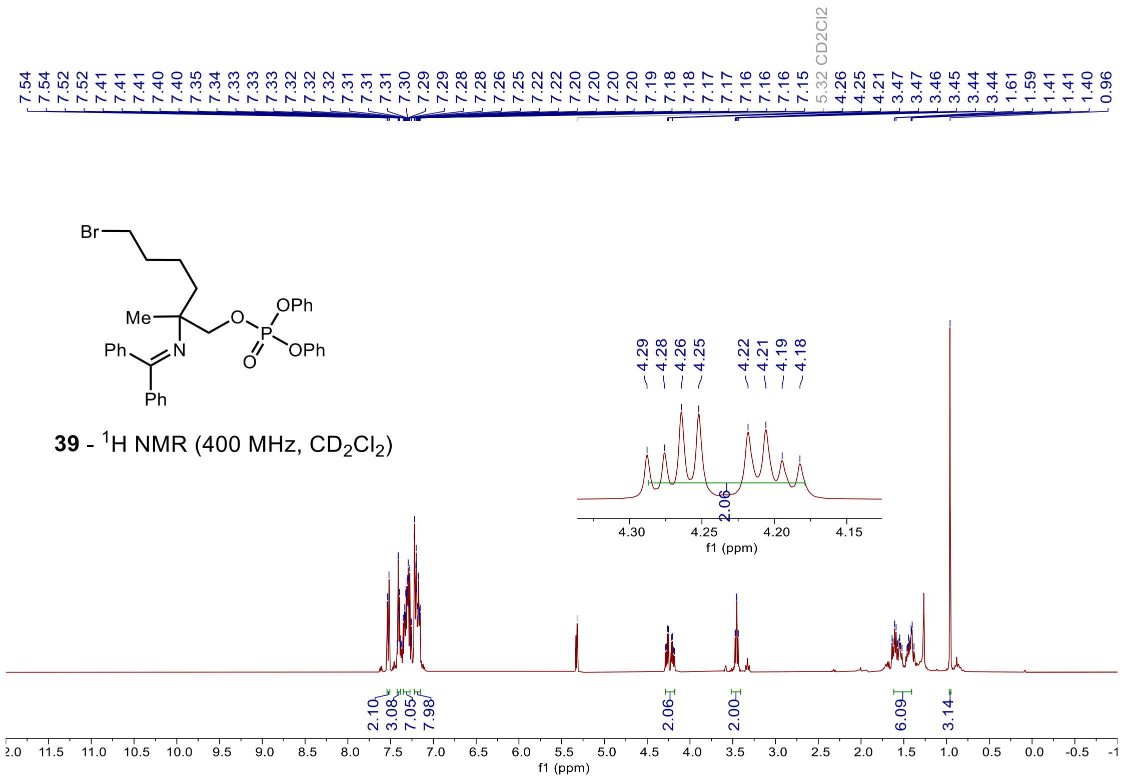

39 - ^{1}H NMR (400 MHz, CD_2Cl_2)

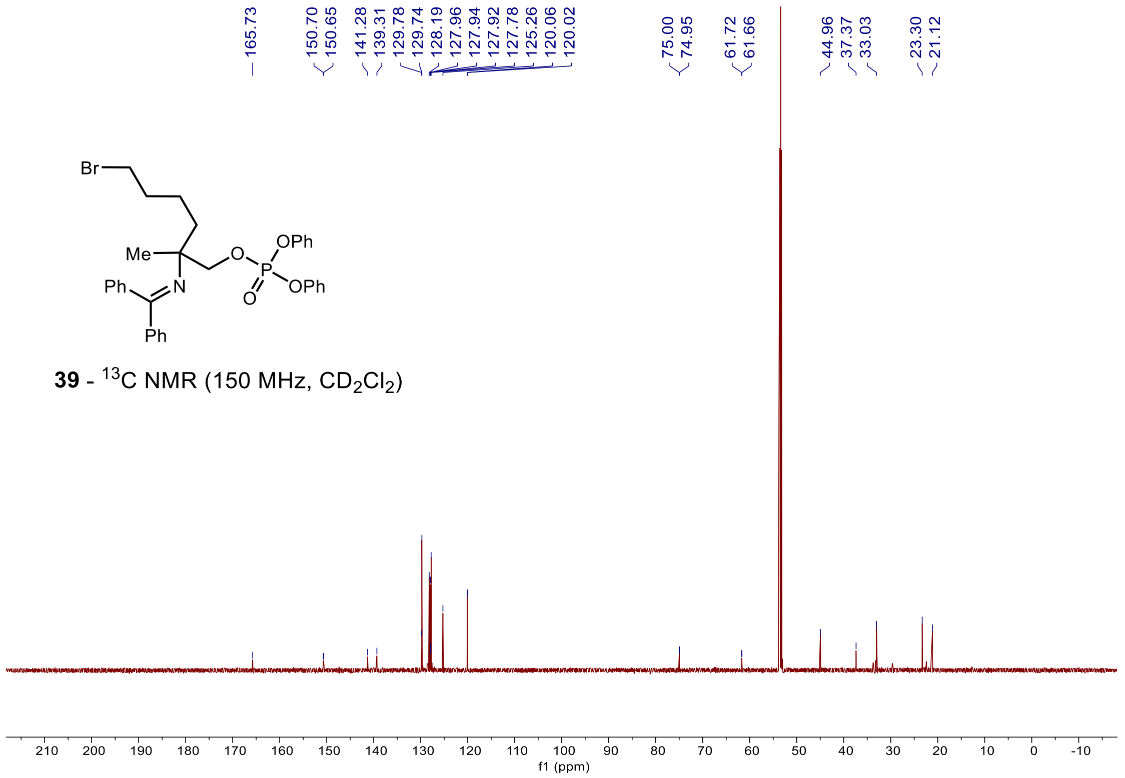

39 - ^{13}C NMR (150 MHz, CD_2Cl_2)

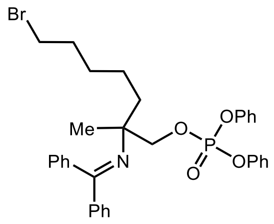

39 - ^{31}P NMR (160 MHz, CD_2Cl_2)

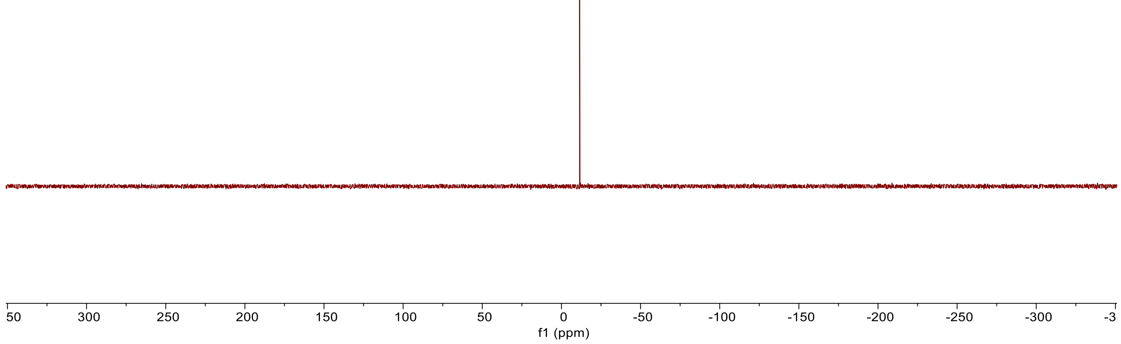

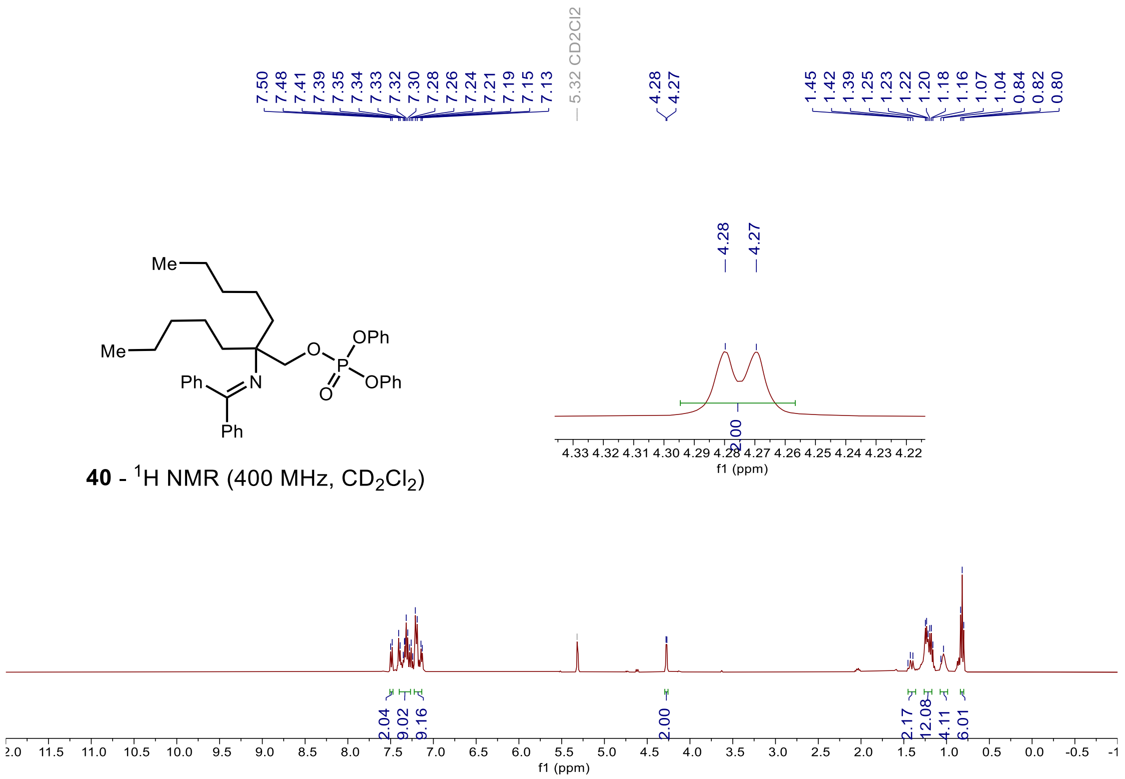

40 - ^{1}H NMR (400 MHz, CD_2Cl_2)

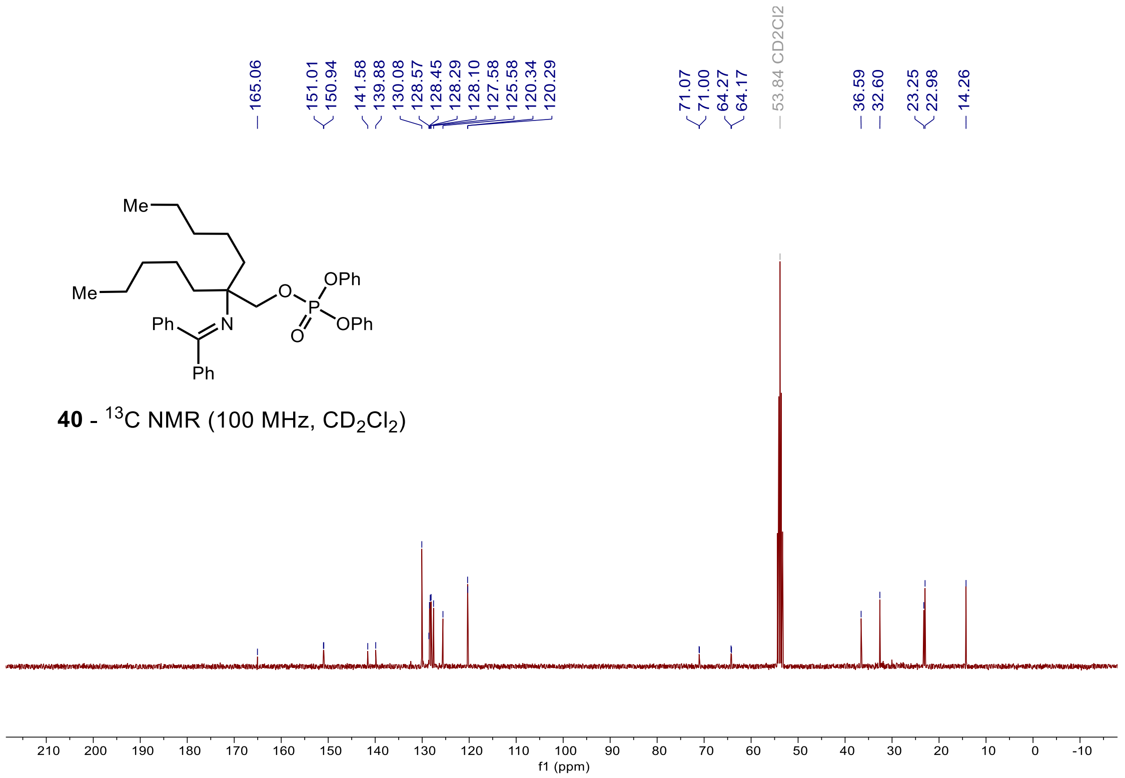

40 - ^{13}C NMR (100 MHz, CD_2Cl_2)

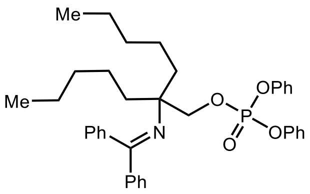

40 - ^{31}P NMR (160 MHz, CD_2Cl_2)

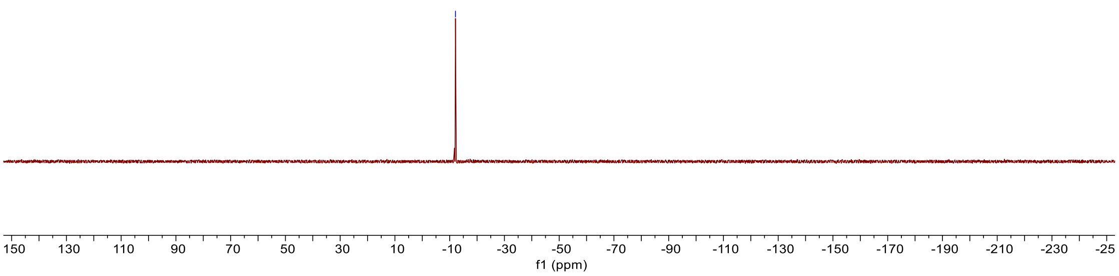

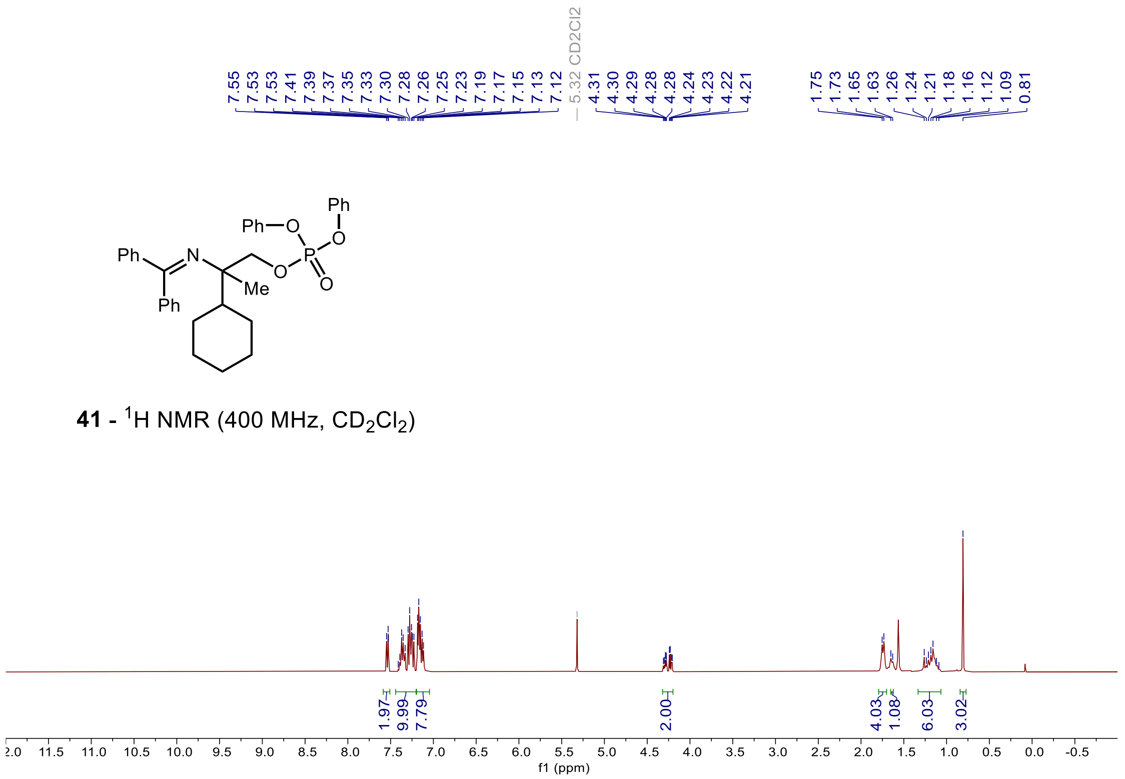

41 - ^{1}H NMR (400 MHz, CD_2Cl_2)

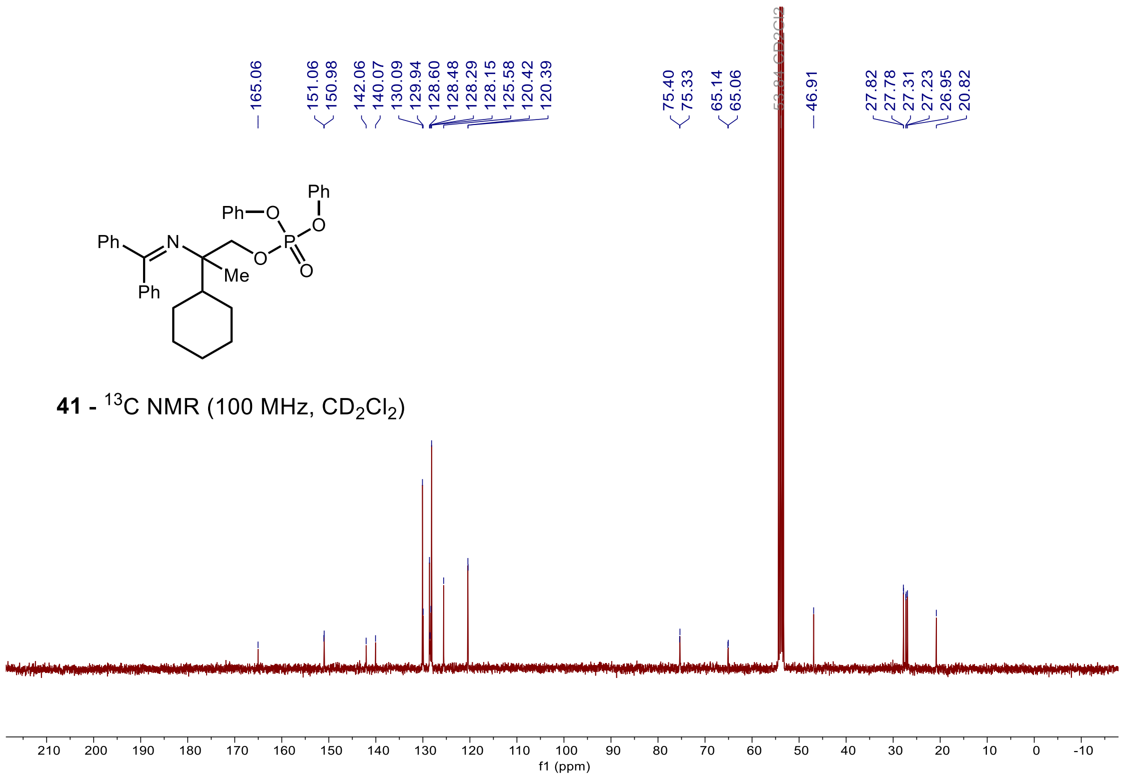

41 - ^{13}C NMR (100 MHz, CD_2Cl_2)

-11.87

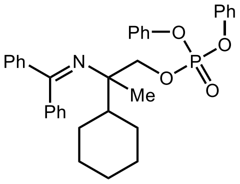

41 - ^{31}P NMR (160 MHz, CD_2Cl_2)

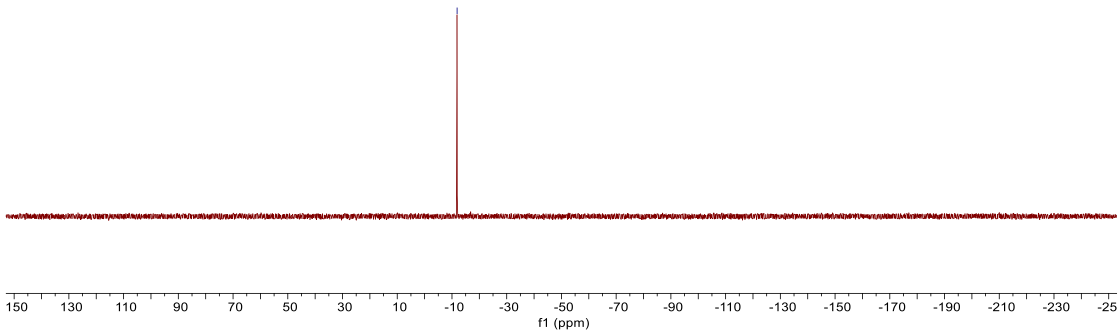

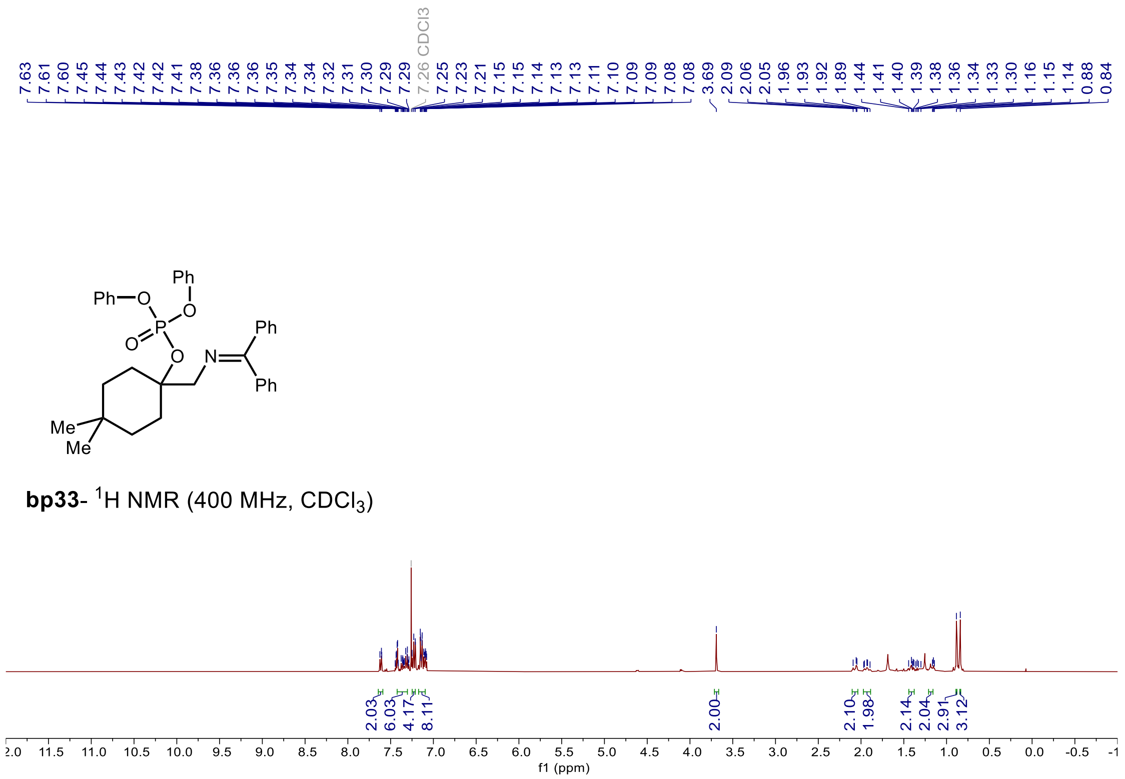

bp33- ^{1}H NMR (400 MHz, $CDCl_3$)

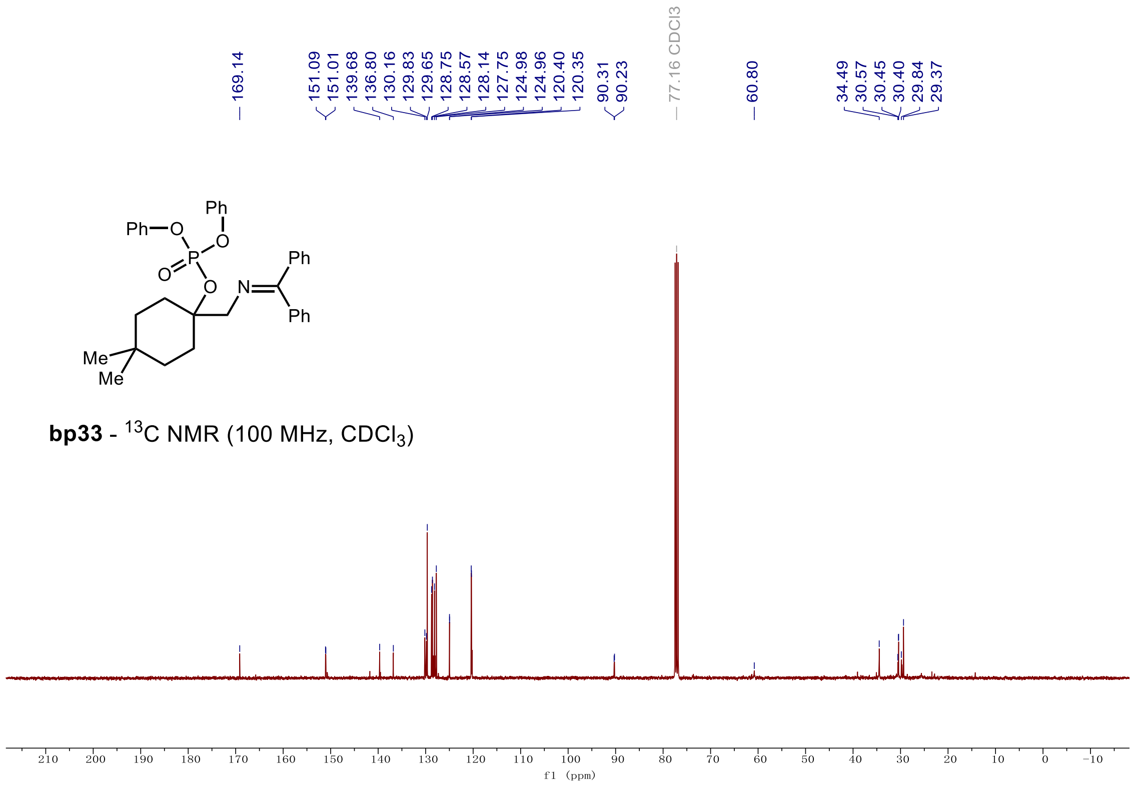

bp33 - ^{13}C NMR (100 MHz, $CDCl_3$)

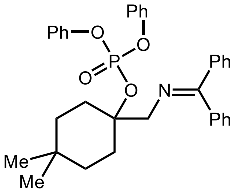

bp33 - ^{31}P NMR (160 MHz, $CDCl_3$)

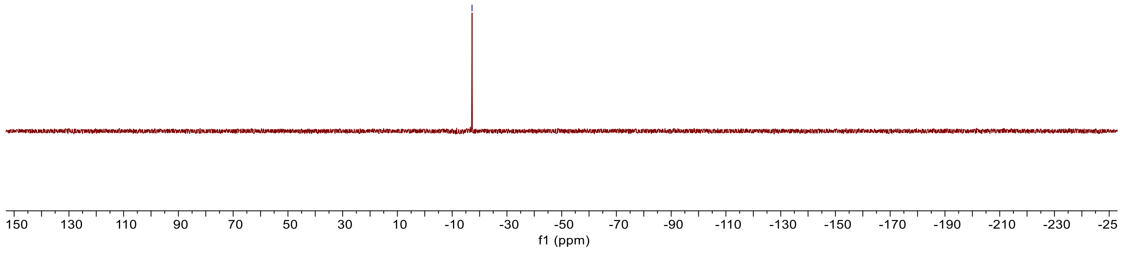

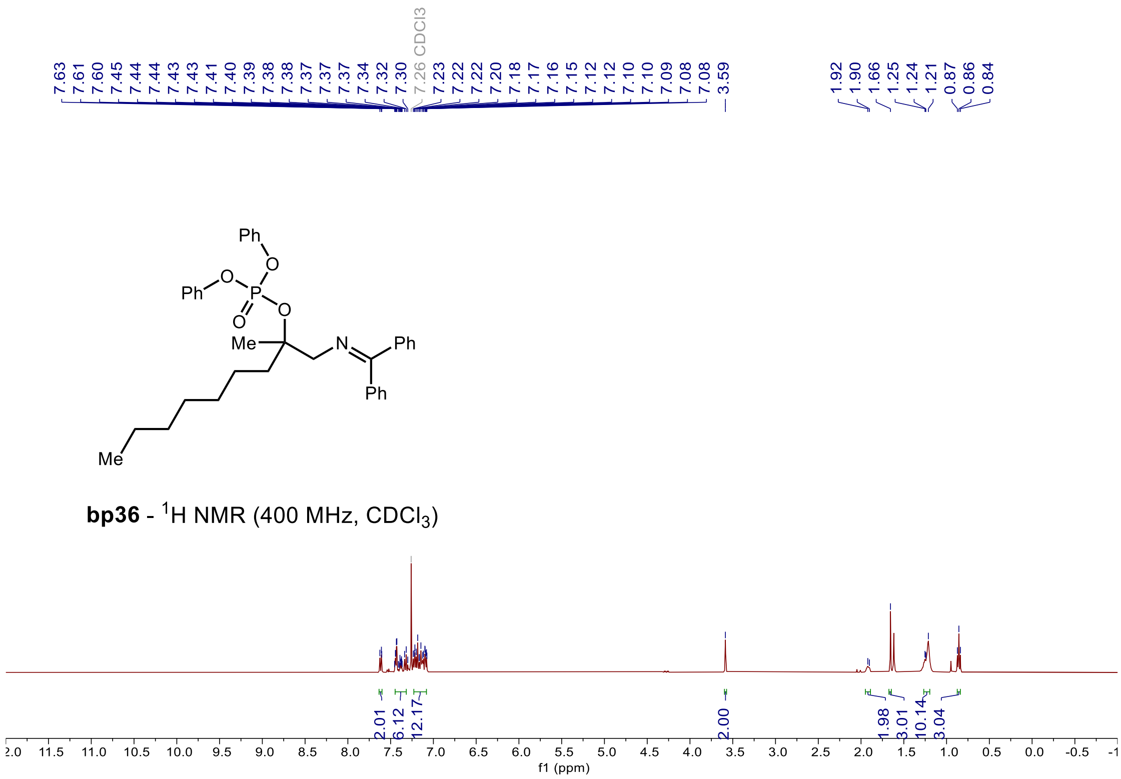

bp36 - ^{1}H NMR (400 MHz, $CDCl_3$)

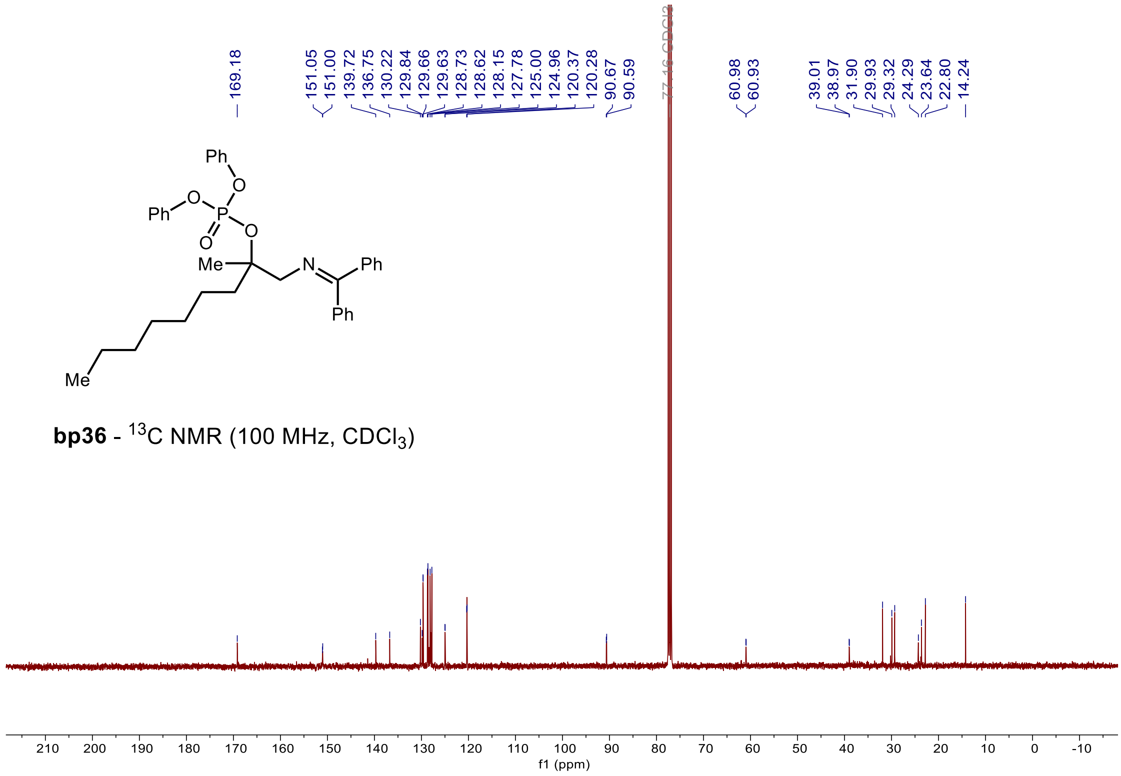

bp36 - ^{13}C NMR (100 MHz, $CDCl_3$)

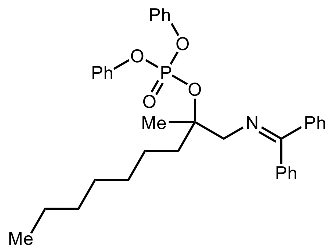

bp36 - ^{31}P NMR (160 MHz, $CDCl_3$)

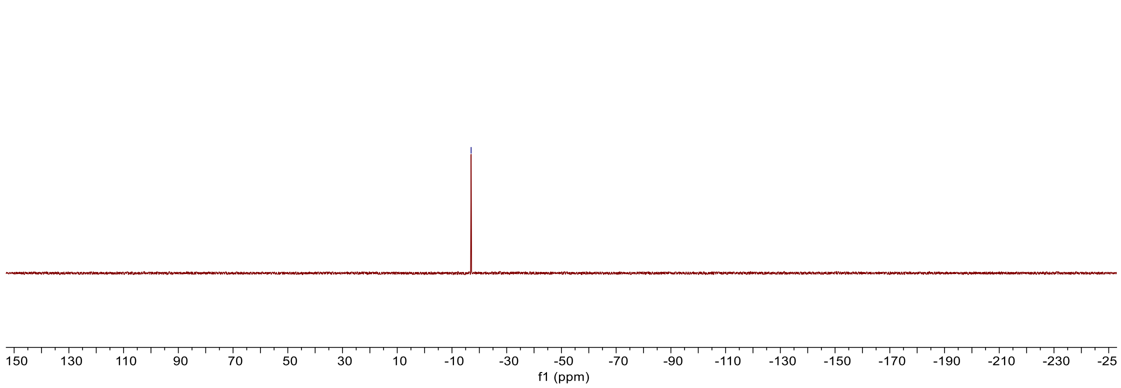

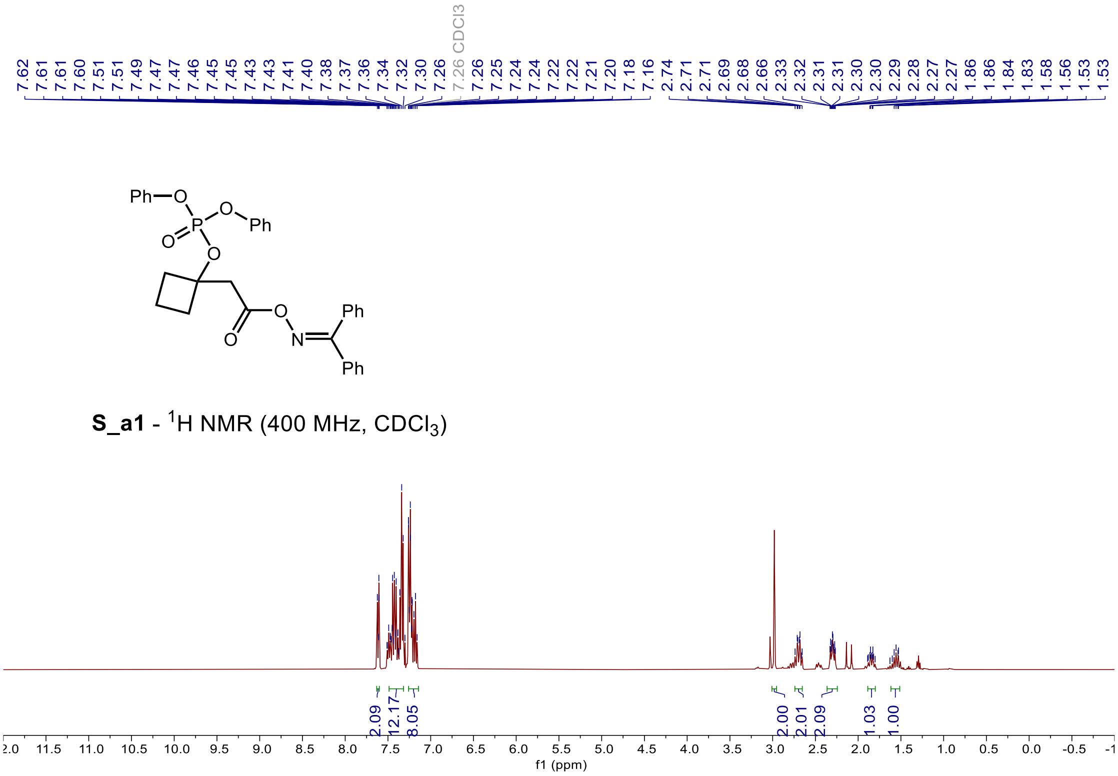

S_a1 - ^{1}H NMR (400 MHz, $CDCl_3$)

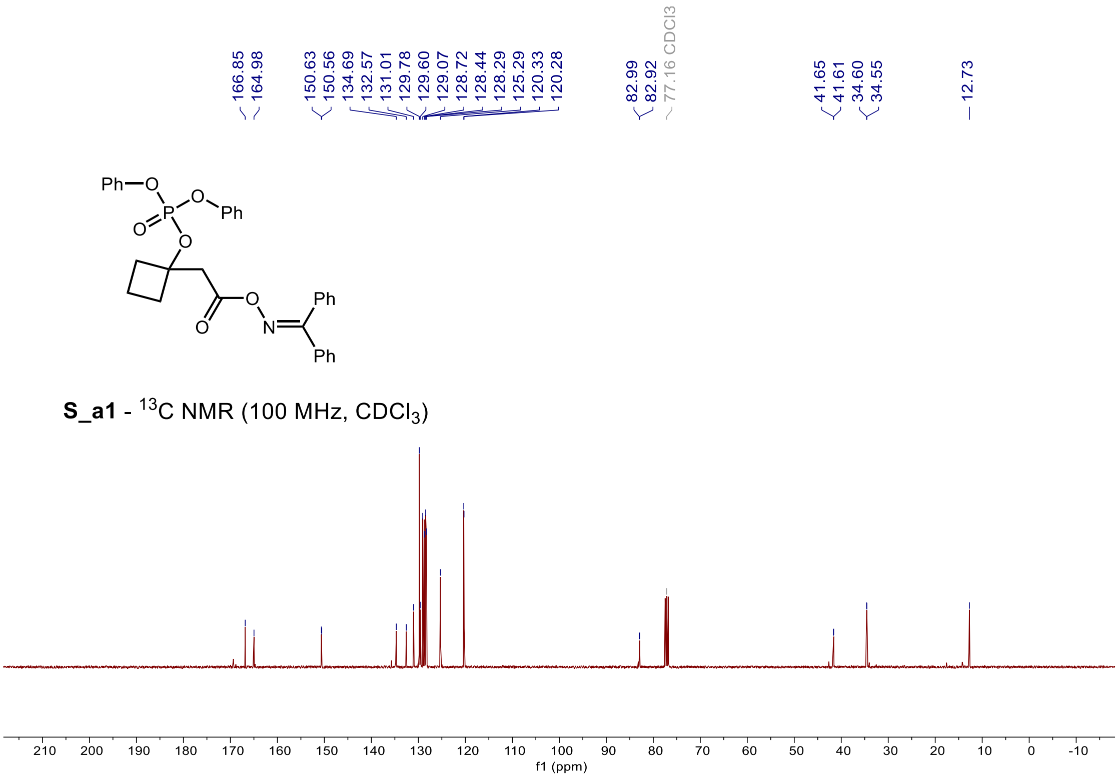

S_a1 - ^{13}C NMR (100 MHz, $CDCl_3$)

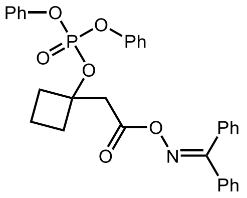

S_a1 - ^{31}P NMR (160 MHz, $CDCl_3$)

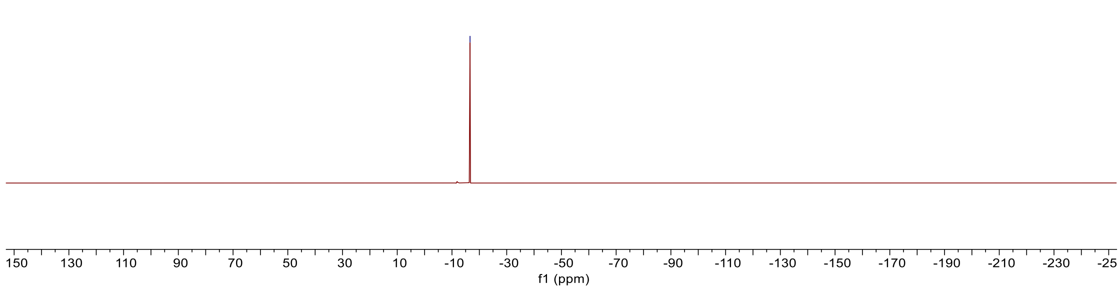

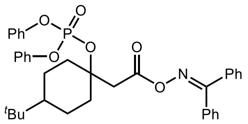

S_a2 - ^{1}H NMR (400 MHz, CD_2Cl_2)

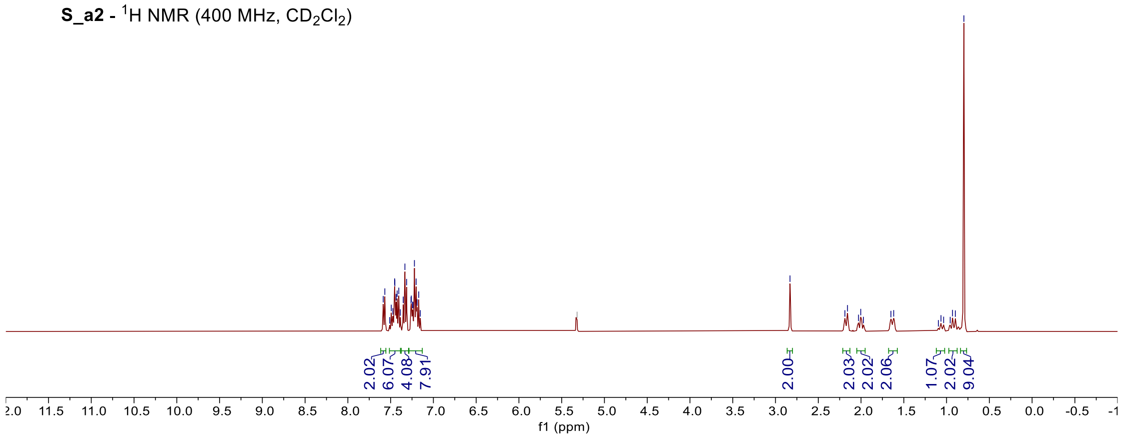

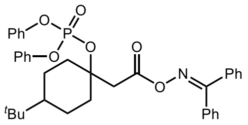

S_a2 - ^{13}C NMR (100 MHz, CD_2Cl_2)

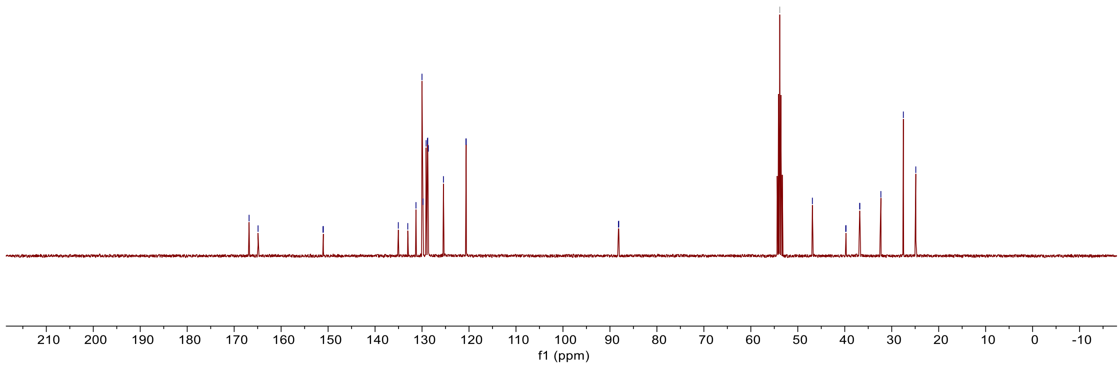

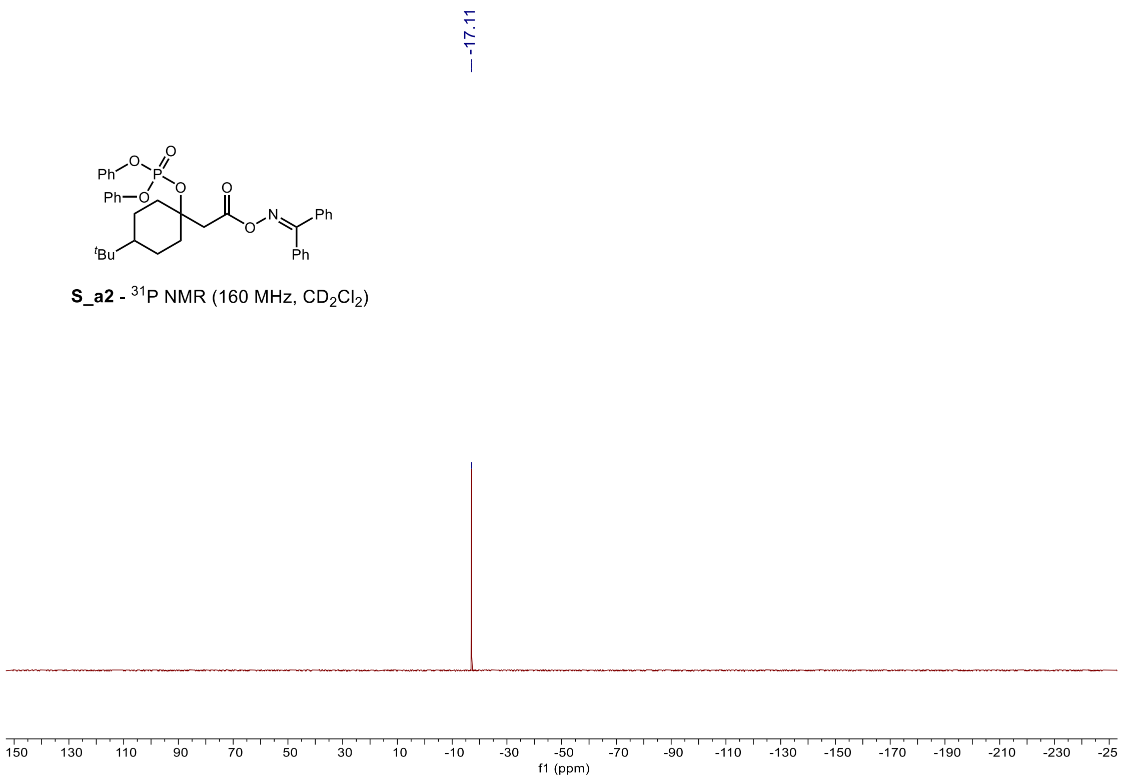

S_a2 - ^{31}P NMR (160 MHz, CD_2Cl_2)

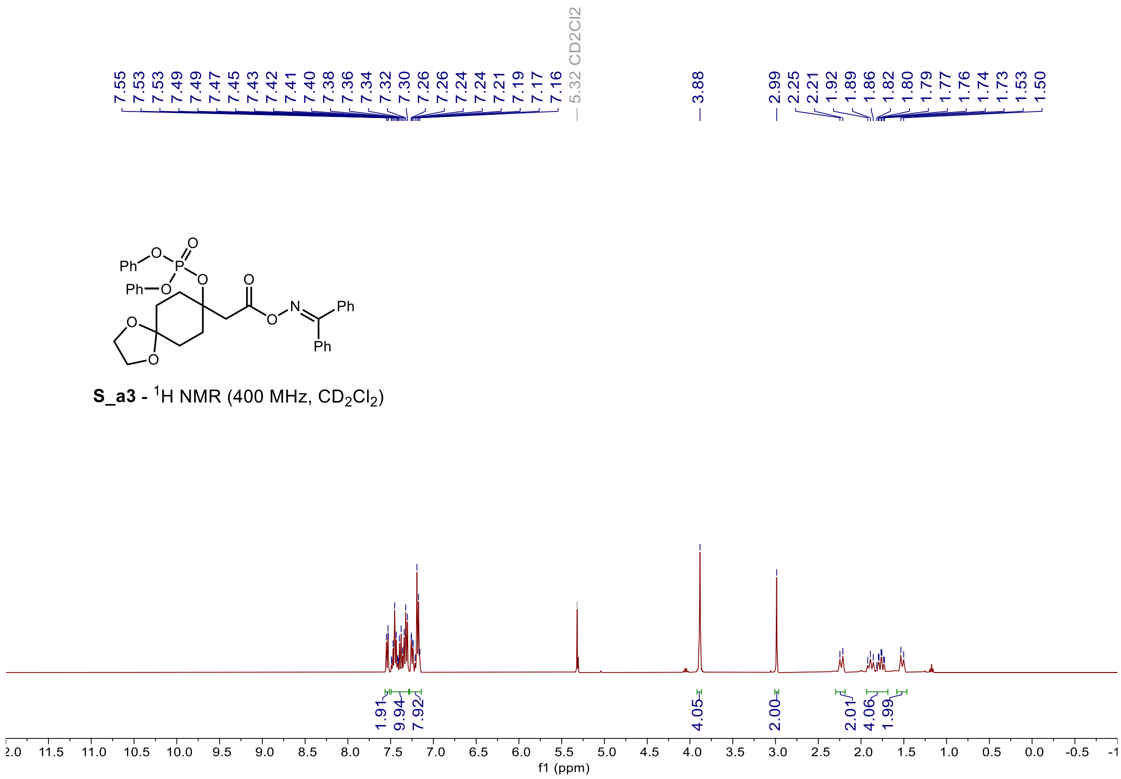

S_a3 - ^{1}H NMR (400 MHz, CD_2Cl_2)

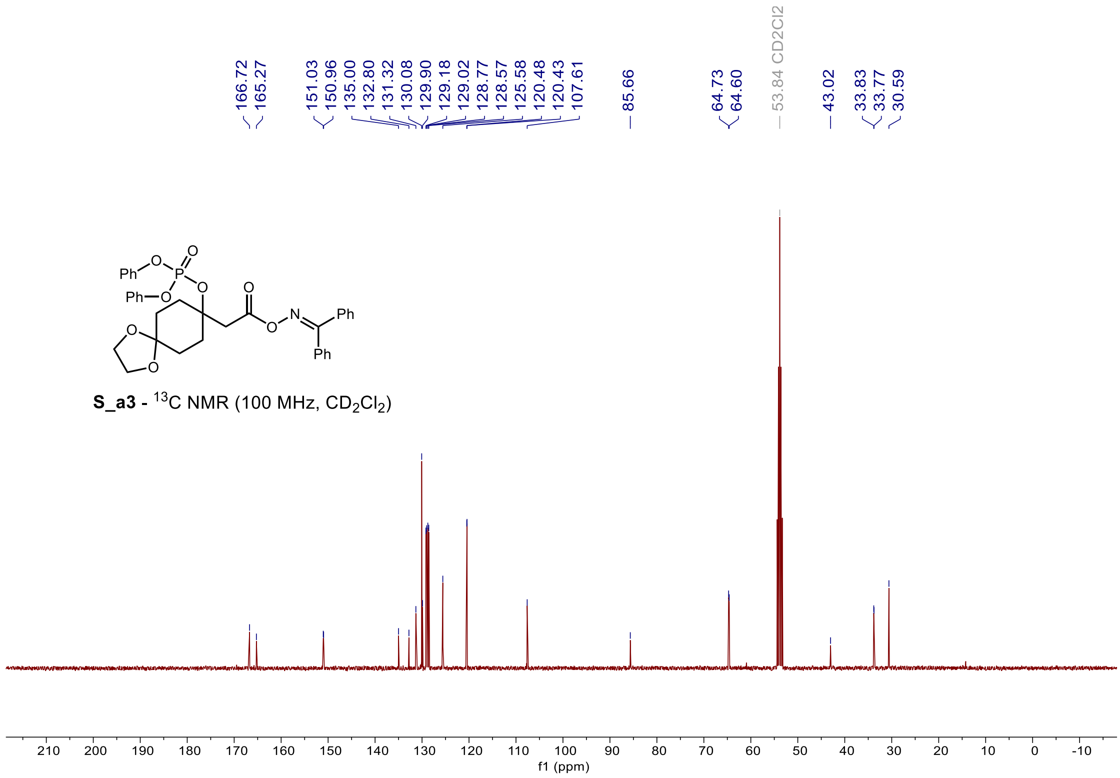

S_a3 - ^{13}C NMR (100 MHz, CD_2Cl_2)

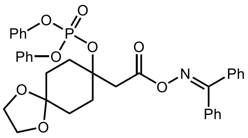

S_a3 - ^{31}P NMR (160 MHz, CD_2Cl_2)

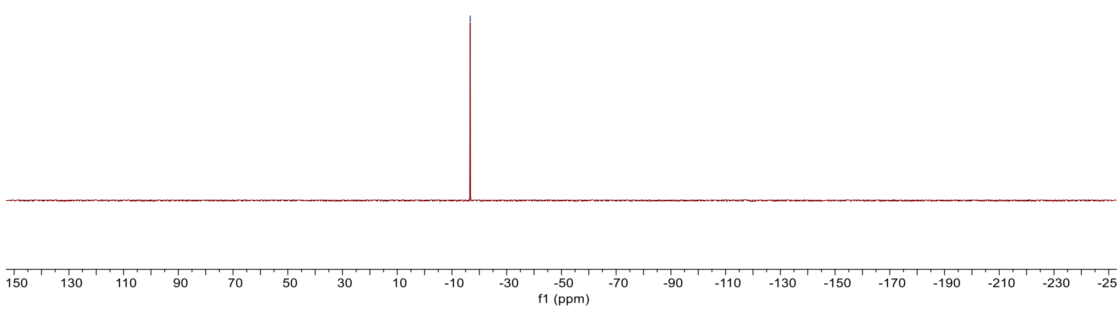

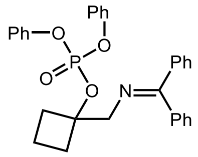

bp_a1 - ^{1}H NMR (400 MHz, $CDCl_3$)

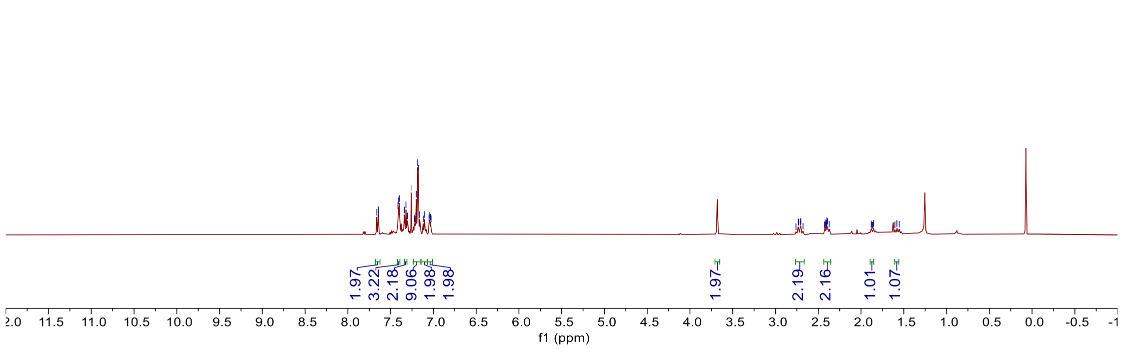

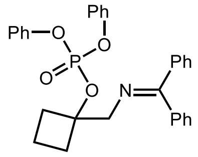

bp_a1 - ^{13}C NMR (100 MHz, $CDCl_3$)

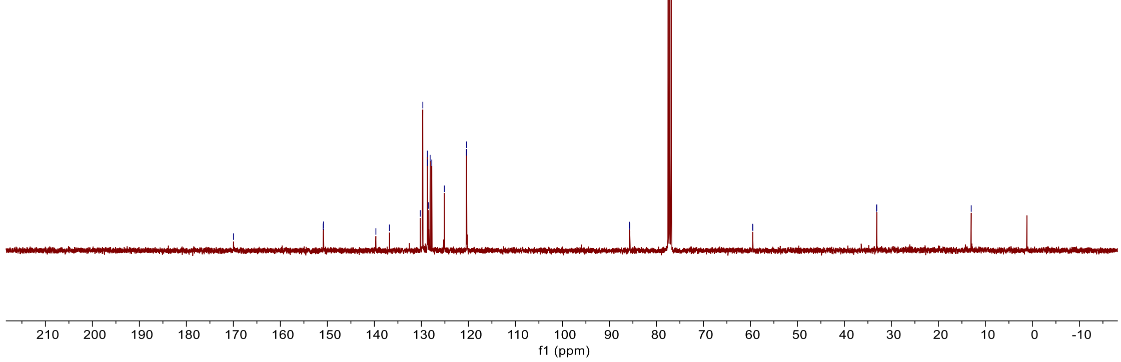

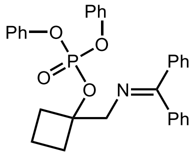

bp_a1 - ^{31}P NMR (160 MHz, $CDCl_3$)

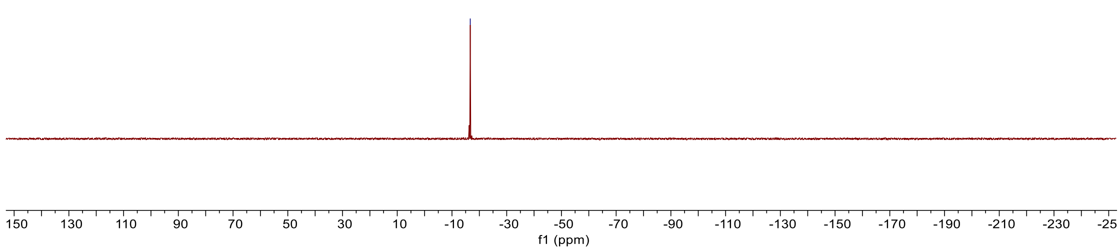

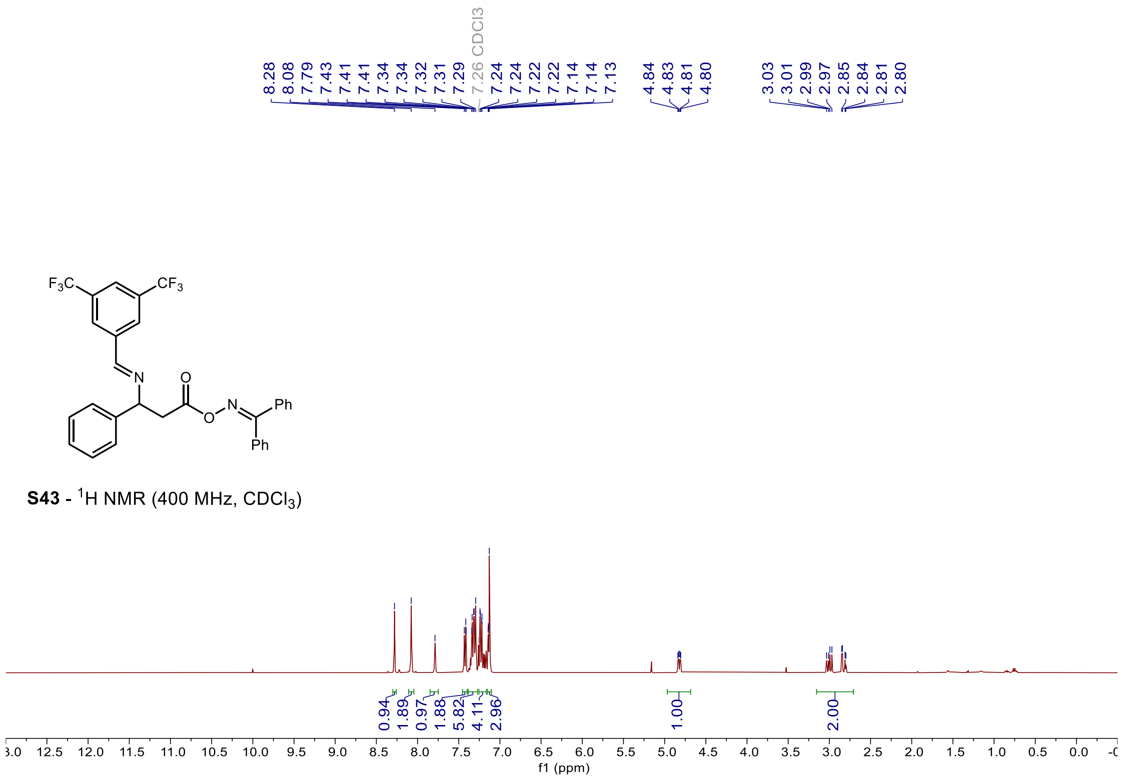

S43 - ^{1}H NMR (400 MHz, $CDCl_3$)

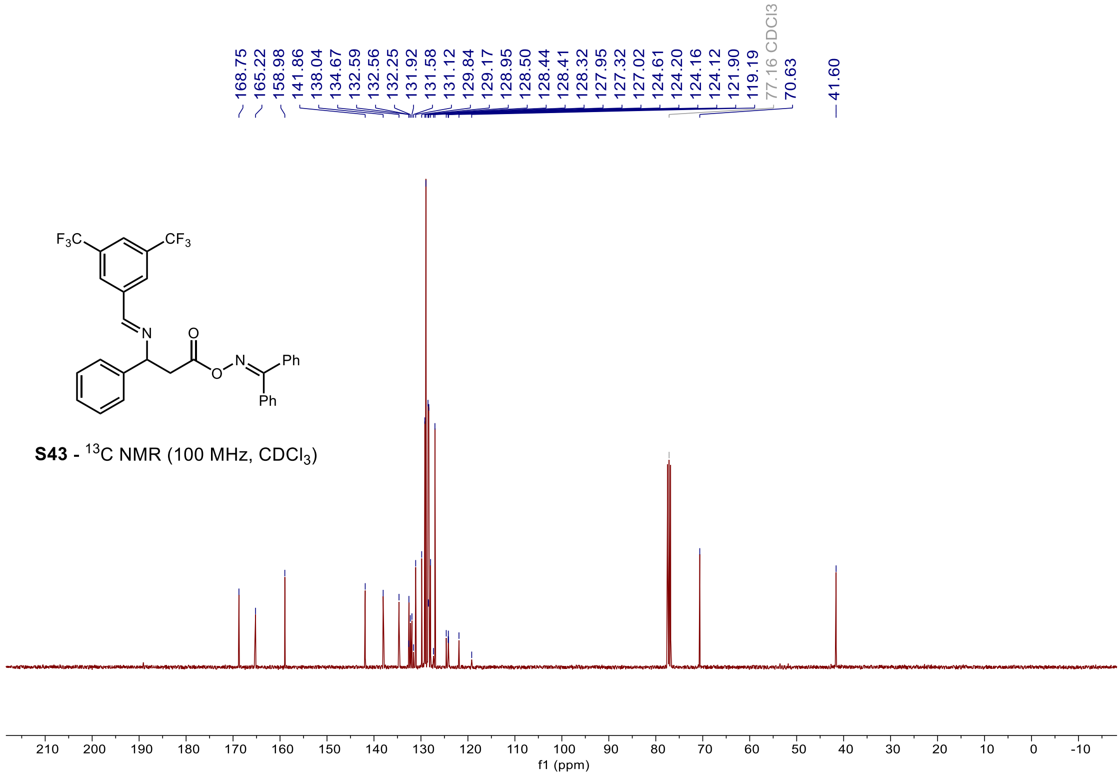

S43 - ^{13}C NMR (100 MHz, $CDCl_3$)

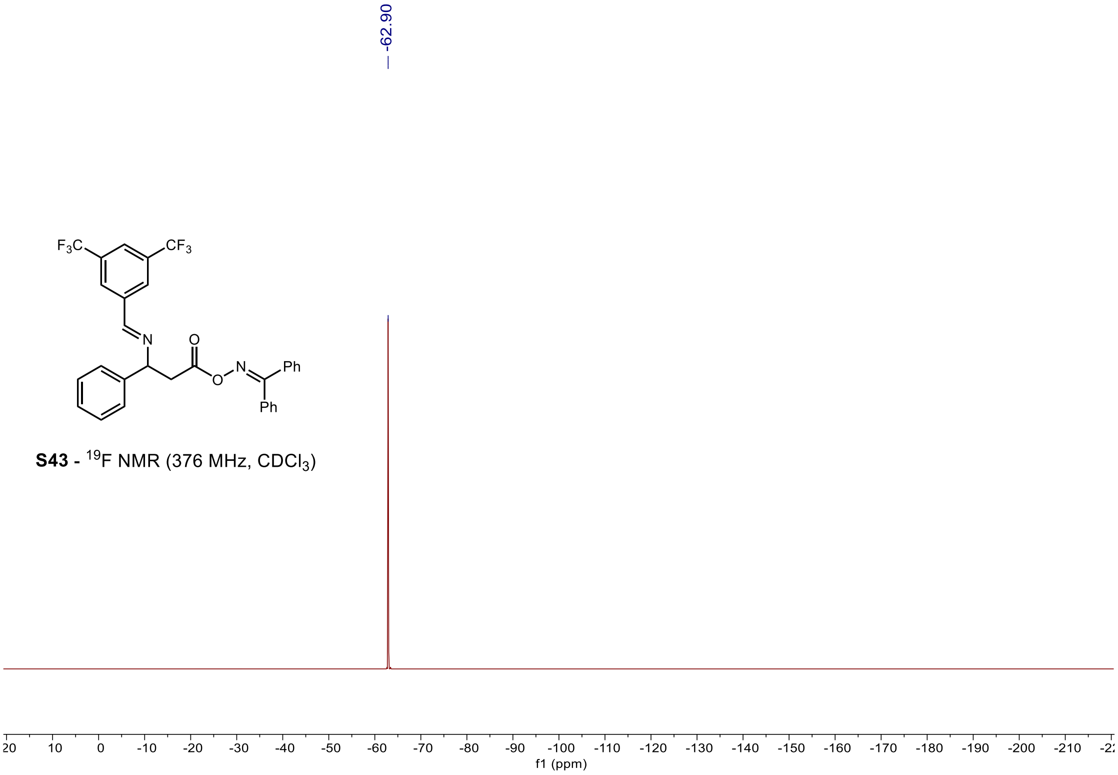

S43 - ^{19}F NMR (376 MHz, $CDCl_3$)

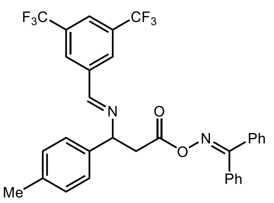

S44 - ^{1}H NMR (400 MHz, $CDCl_3$)

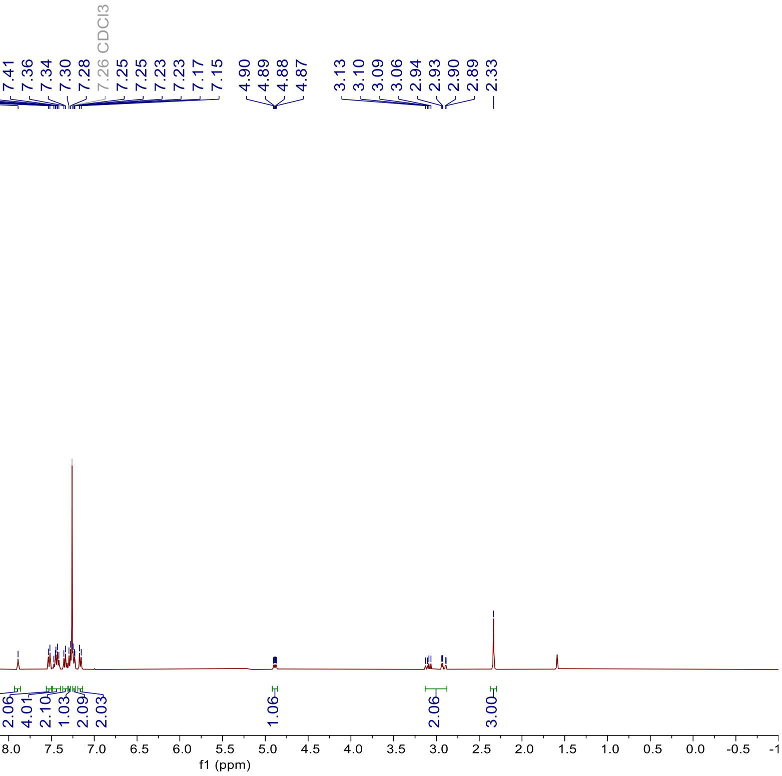

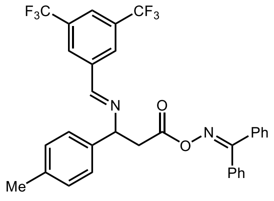

S44 - ^{13}C NMR (100 MHz, $CDCl_3$)

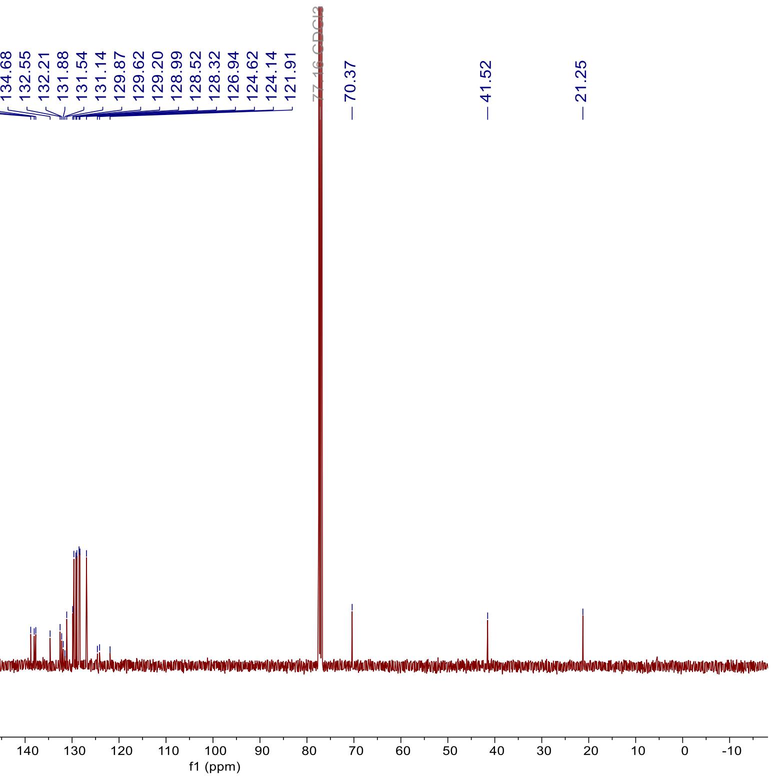

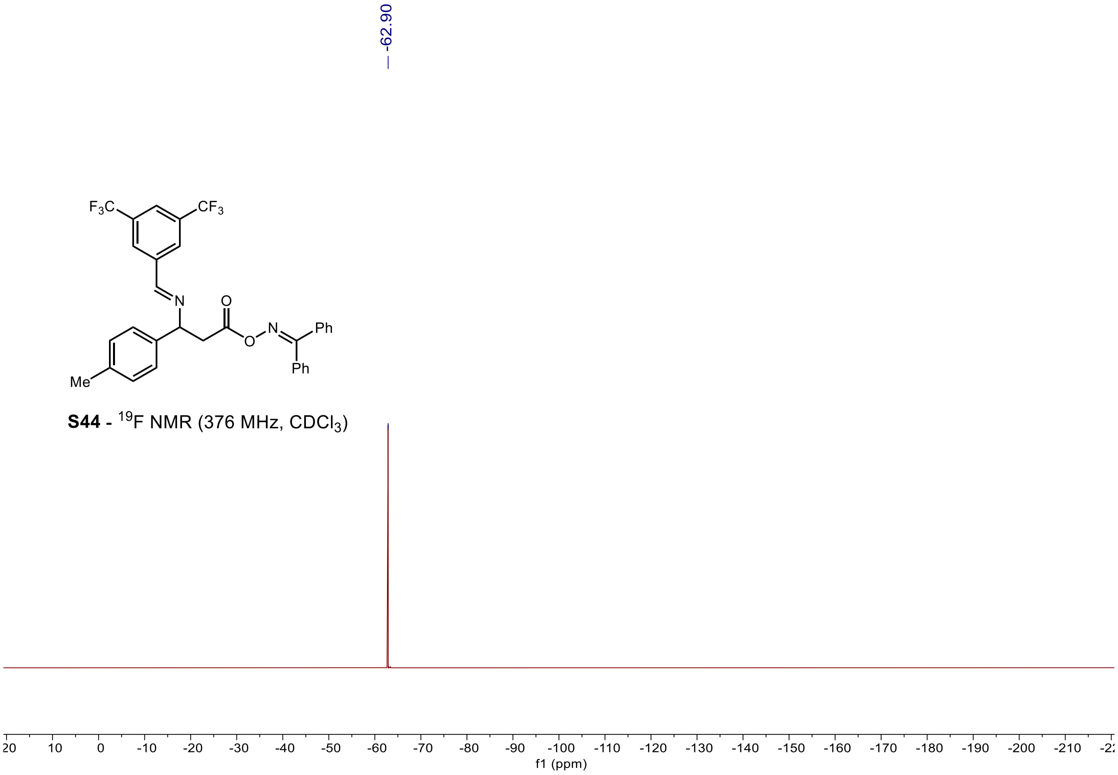

S44 - ^{19}F NMR (376 MHz, $CDCl_3$)

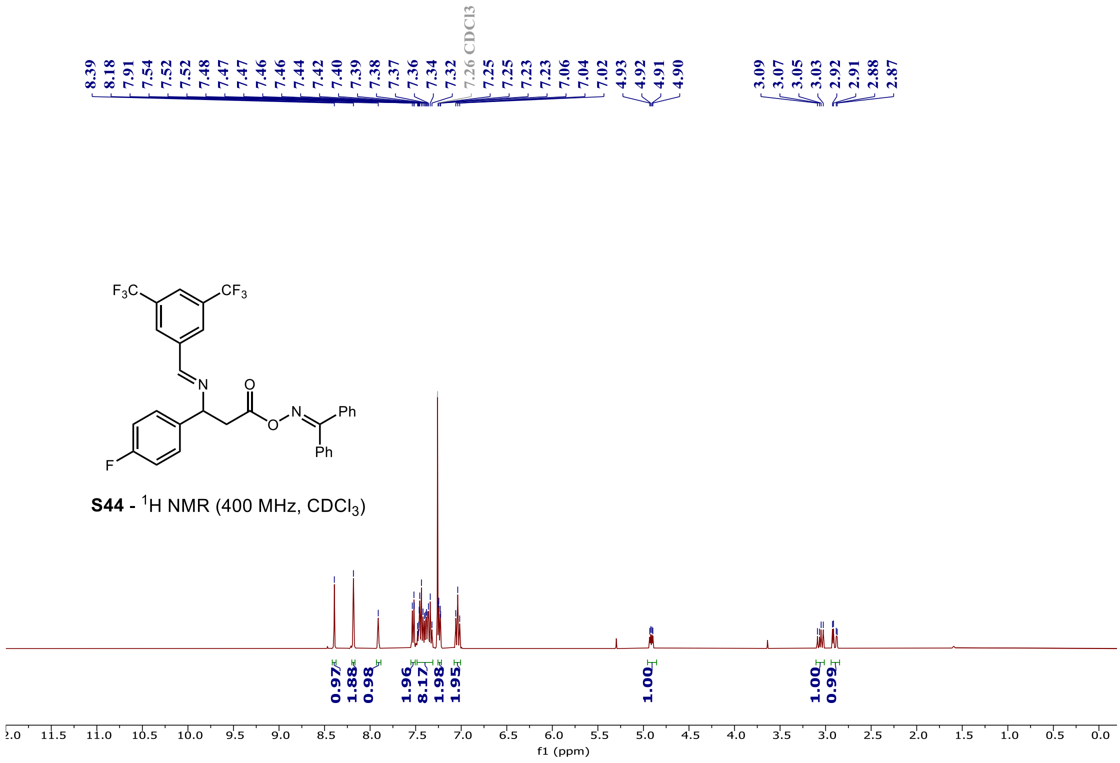

S44 - ^{1}H NMR (400 MHz, $CDCl_3$)

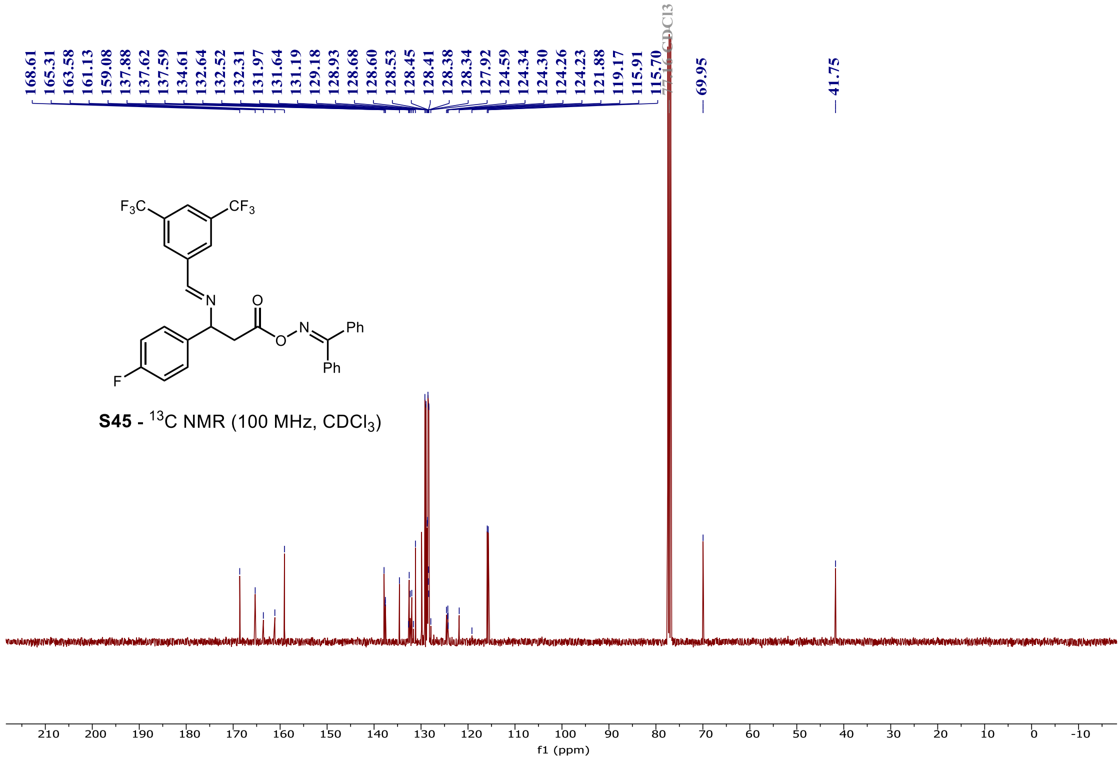

S45 - ^{13}C NMR (100 MHz, $CDCl_3$)

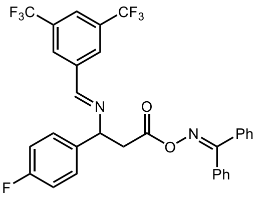

S45 - ^{19}F NMR (376 MHz, $CDCl_3$)

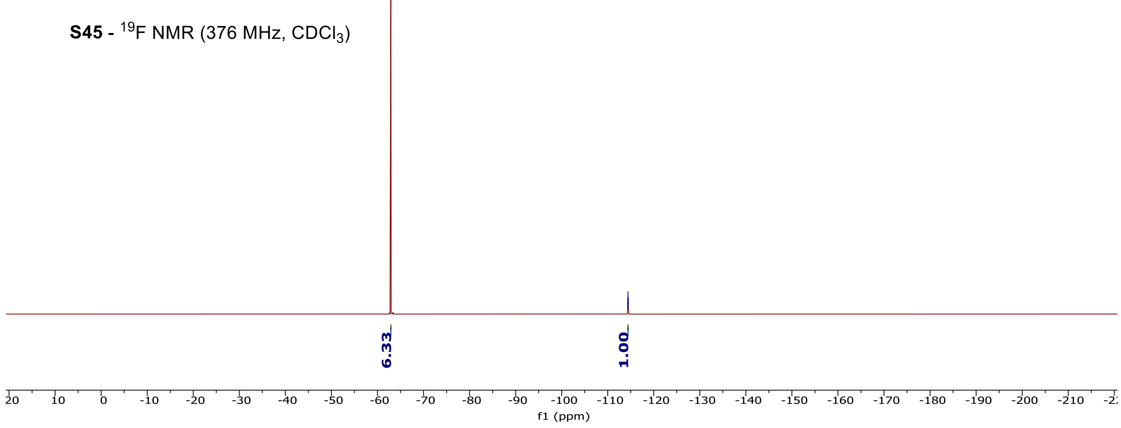

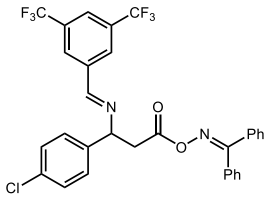

S46 - ^{1}H NMR (400 MHz, CD_2Cl_2)

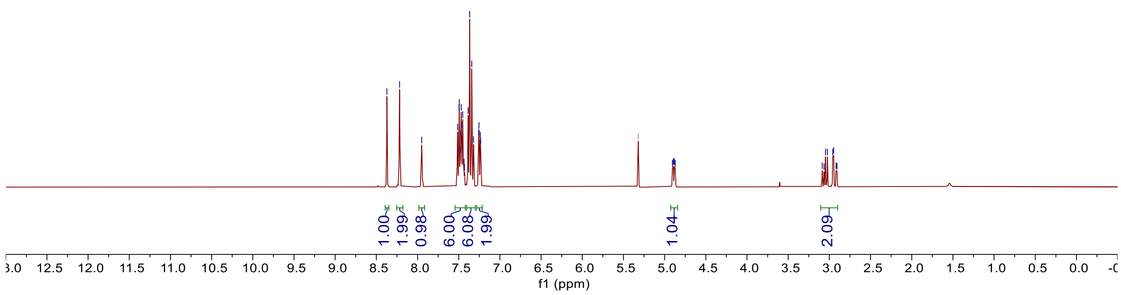

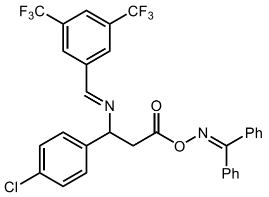

S46 - ^{13}C NMR (100 MHz, CD_2Cl_2)

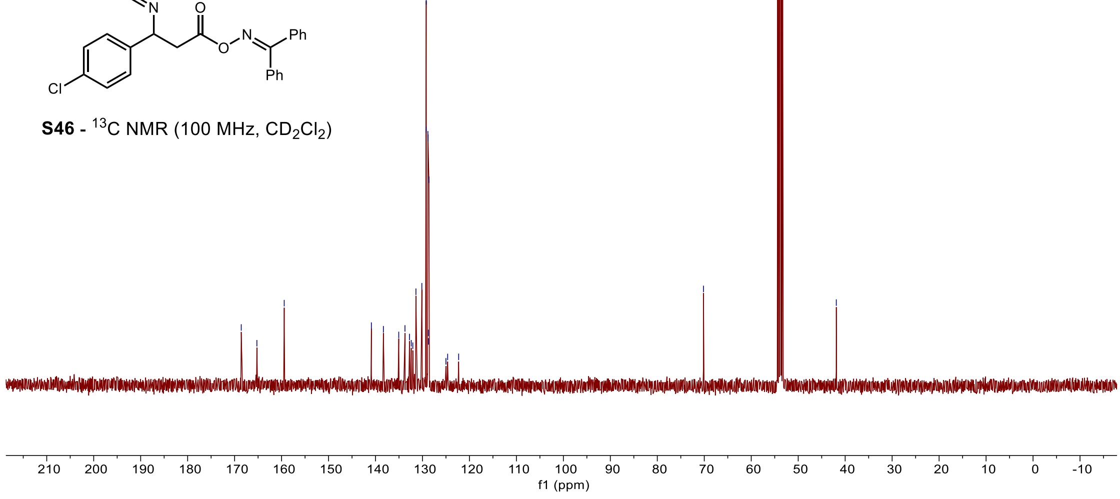

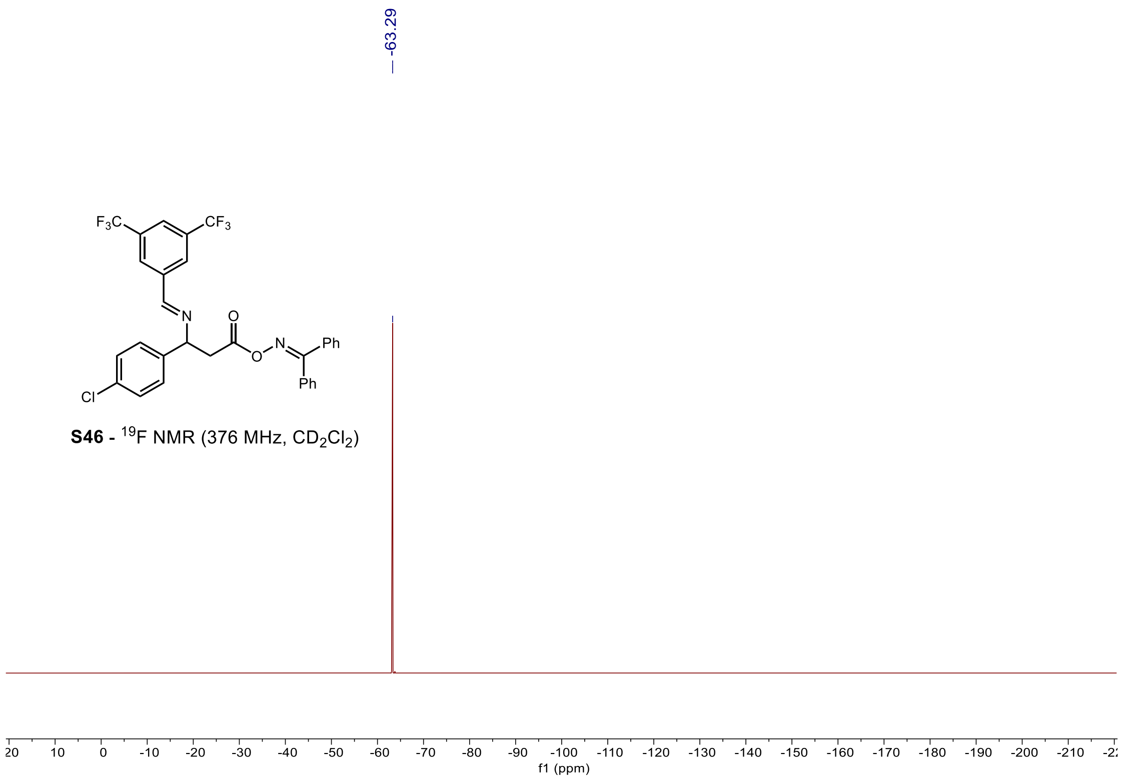

S46 - ^{19}F NMR (376 MHz, CD_2Cl_2)

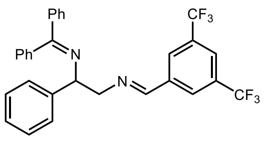

43 - ^{1}H NMR (400 MHz, C_6D_6)

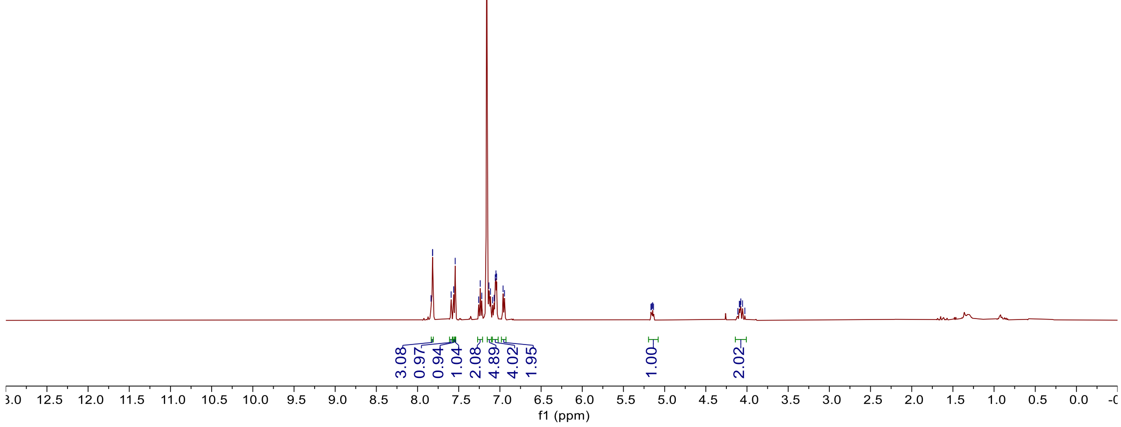

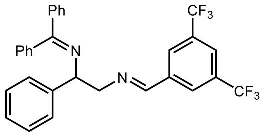

43 - ^{13}C NMR (150 MHz, C_6D_6)

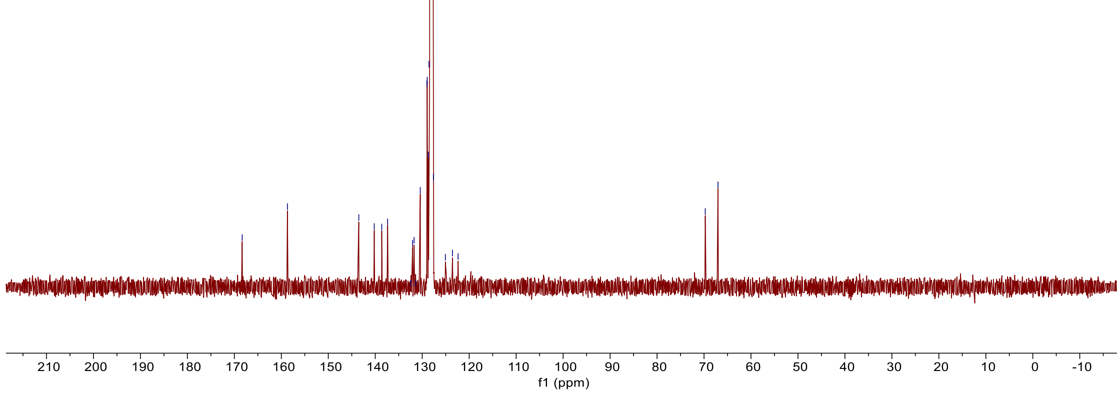

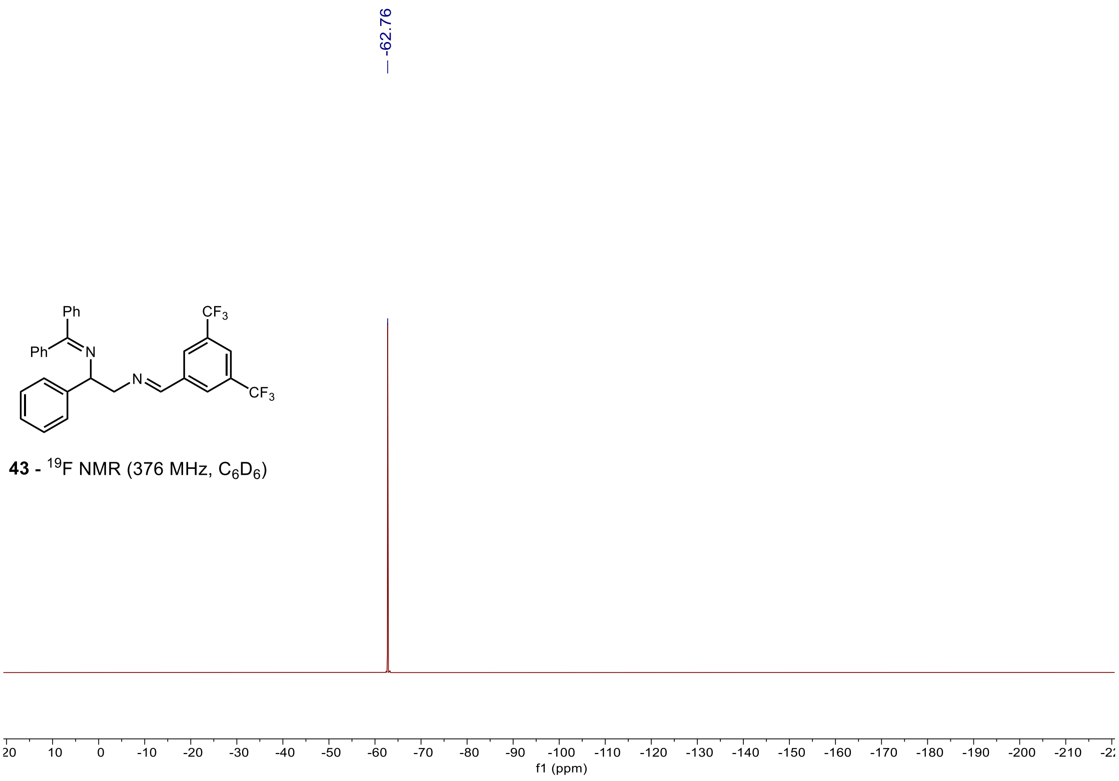

43 - ^{19}F NMR (376 MHz, C_6D_6)

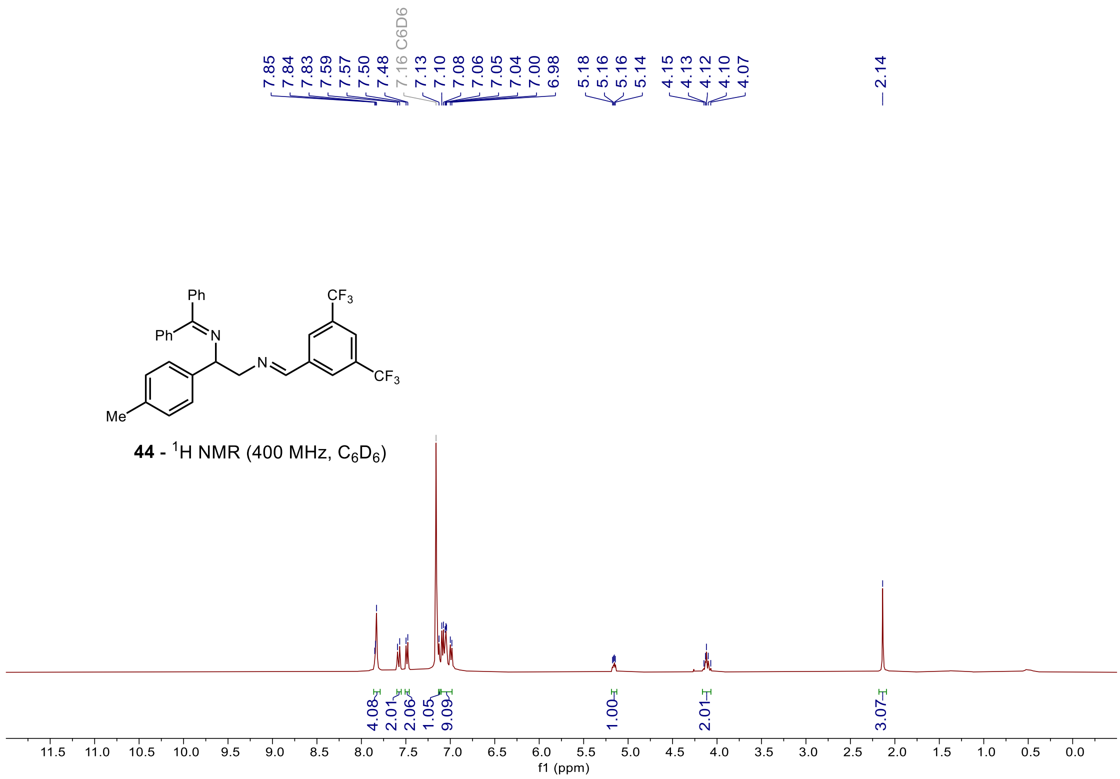
7.85
7.84
7.83
7.59
7.57
7.50
7.48
7.16 C6D6
7.13
7.10
7.08
7.06
7.05
7.04
7.00
6.98
5.18
5.16
5.16
5.14
4.15
4.13
4.12
4.10
4.07
2.14
Ph
Ph
N
N
CF3
CF3
Me
44 - 1H NMR (400 MHz, C6D6)
4.08
2.01
2.06
1.05
9.09
1.00
2.01
3.07
f1 (ppm)

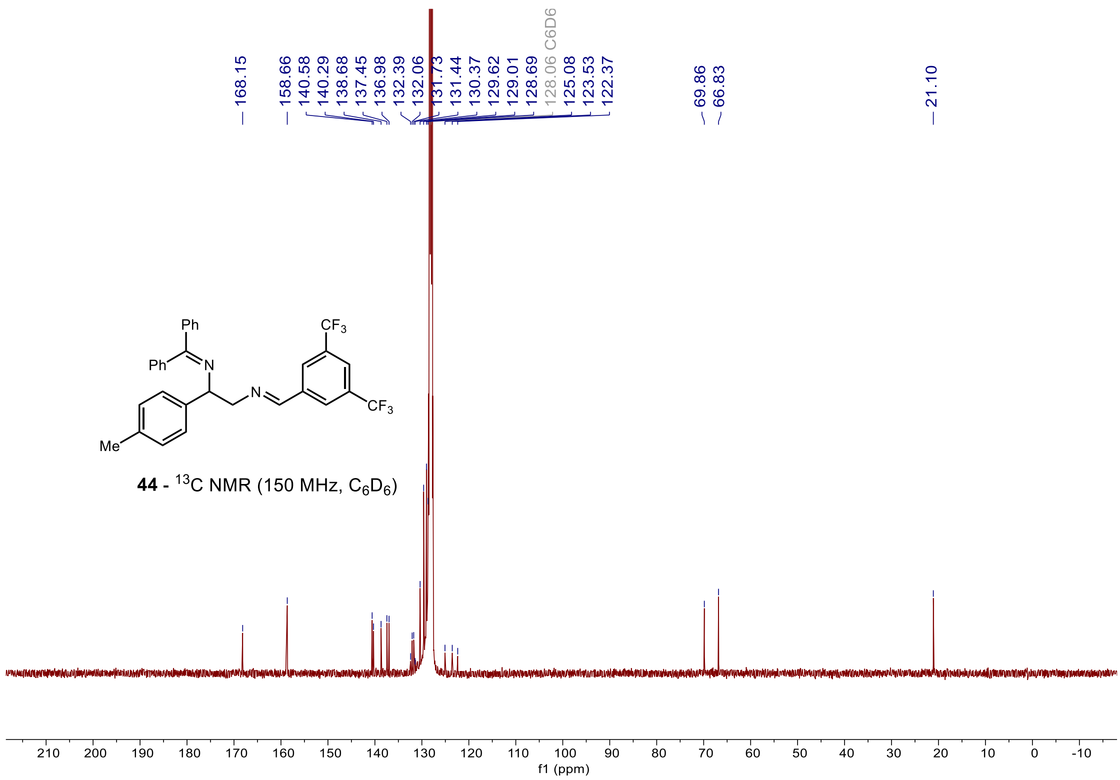
168.15
158.66
140.58
140.29
138.68
137.45
136.98
132.39
132.06
131.73
131.44
130.37
129.62
129.01
128.69
128.06 C6D6
125.08
123.53
122.37
69.86
66.83
21.10
Ph
Ph
N
N
CF3
CF3
Me
44 - 13C NMR (150 MHz, C6D6)
f1 (ppm)

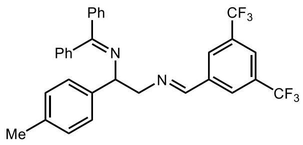

44 - ^{19}F NMR (376 MHz, C_6D_6)

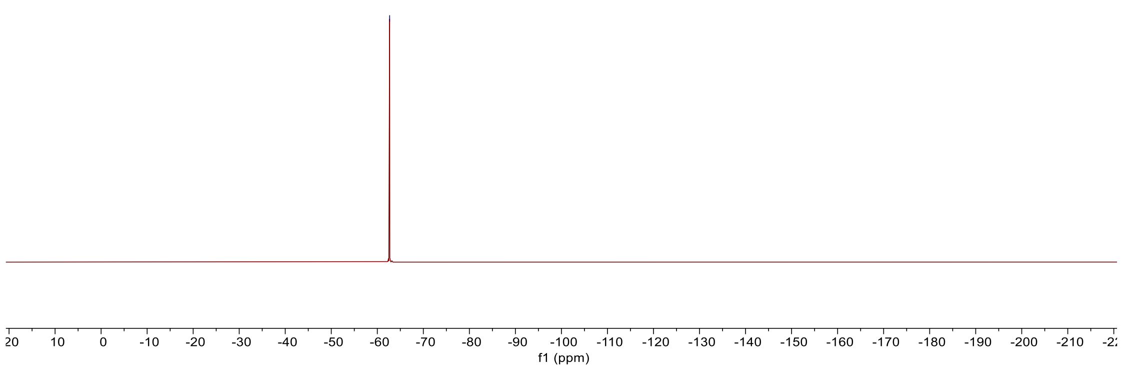

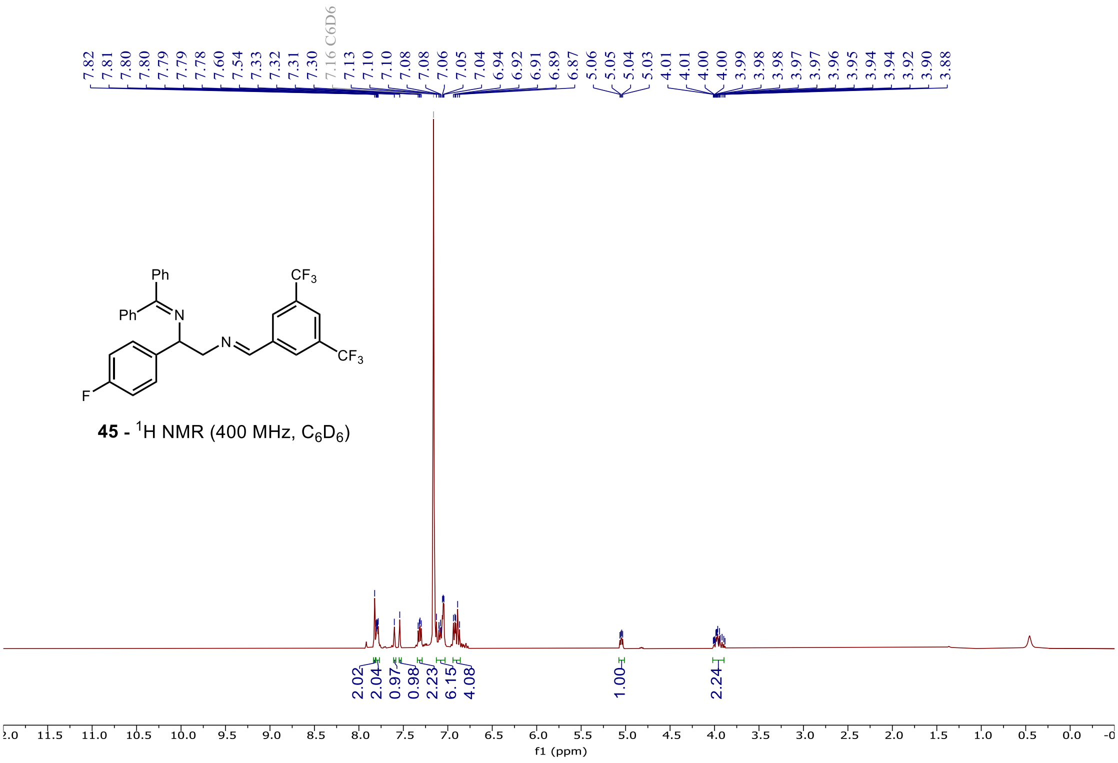

45 - ^{1}H NMR (400 MHz, C_6D_6)

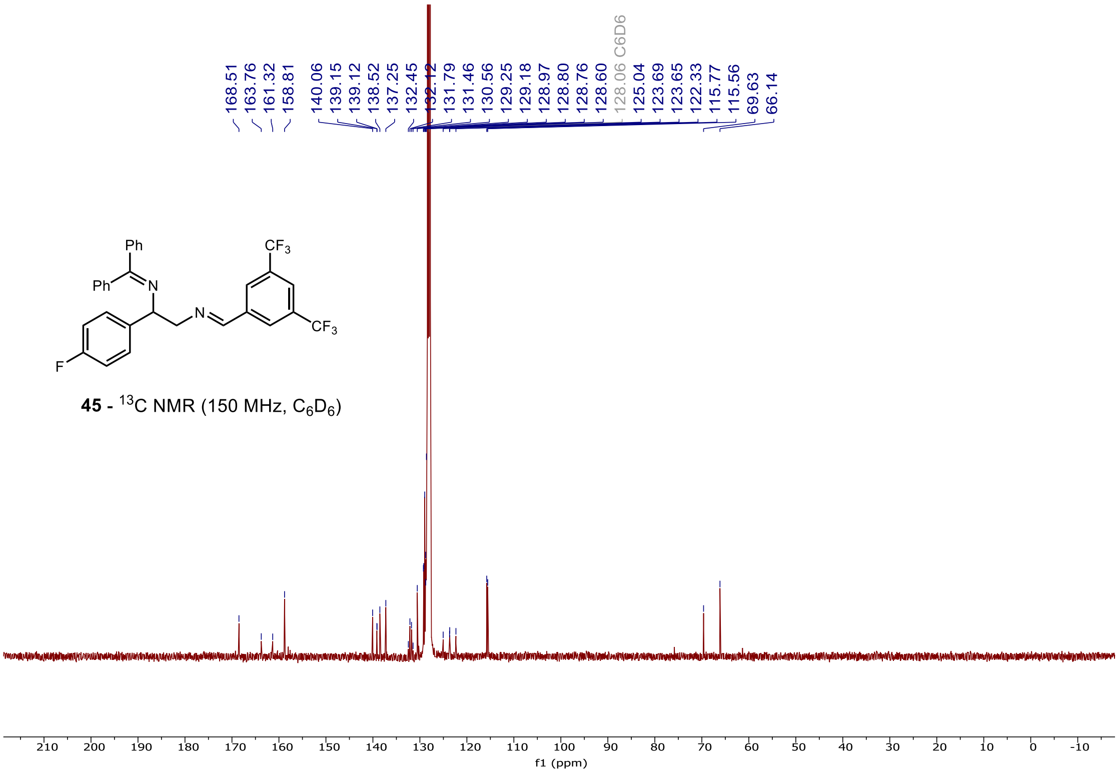

45 - ^{13}C NMR (150 MHz, C_6D_6)

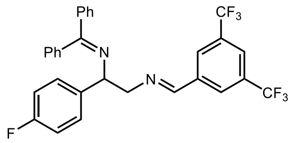

45 - ^{19}F NMR (376 MHz, C_6D_6)

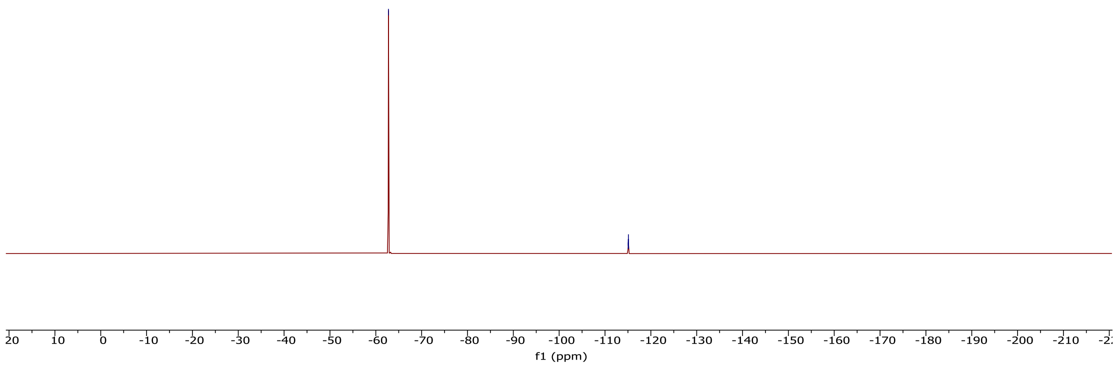

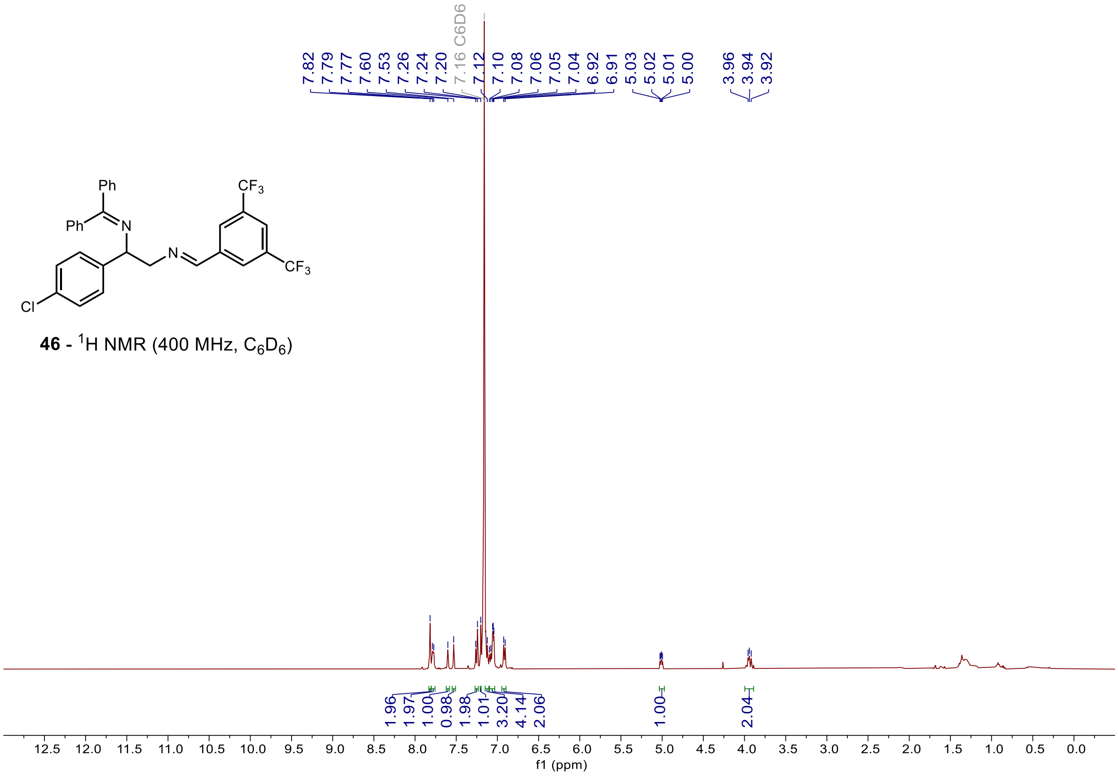

46 - ^{1}H NMR (400 MHz, C_6D_6)

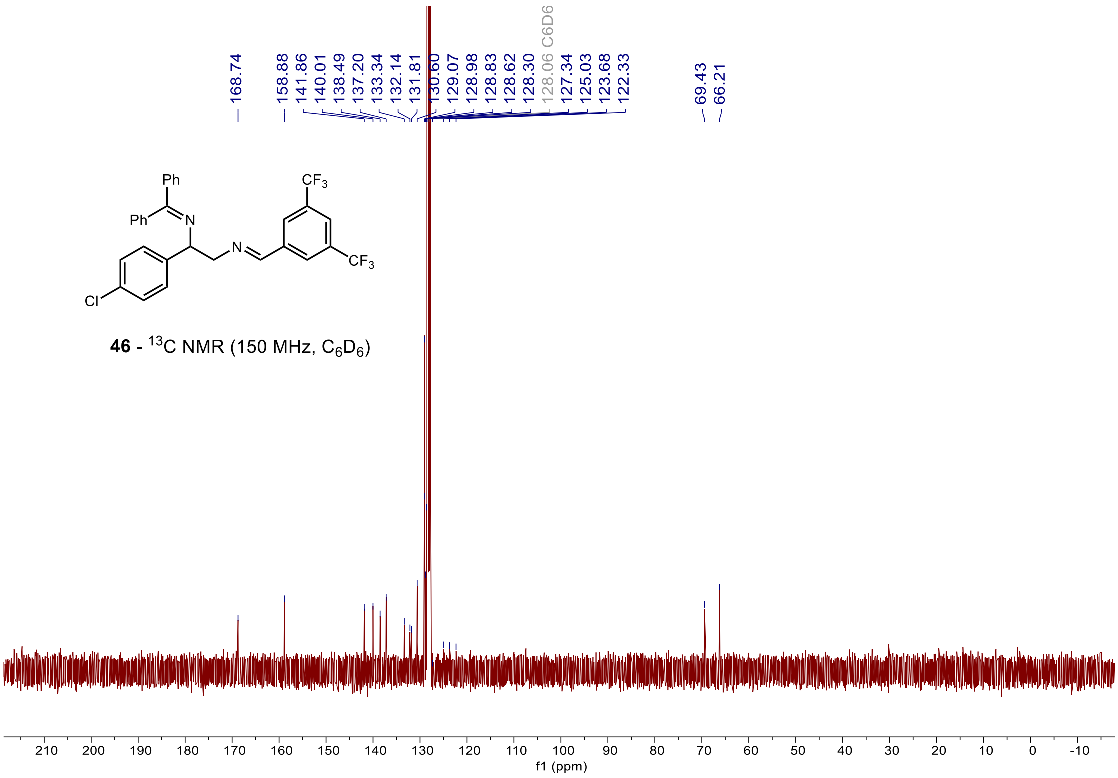

46 - ^{13}C NMR (150 MHz, C_6D_6)

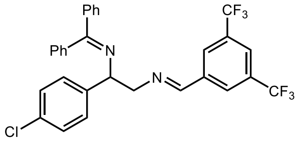

46 - ^{19}F NMR (376 MHz, C_6D_6)

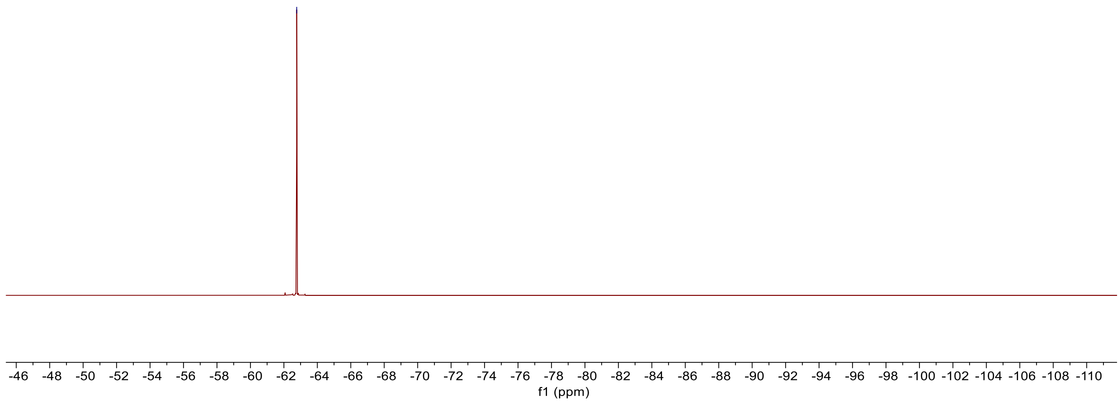

Supplement: Supplementary file 1 — Supporting File: advs76772‐sup‐0001‐SuppMat.pdf. [file ADVS-9999-e76772-s001.pdf]
